# A Zintl Cluster for Transition Metal-Free Catalysis: C=O Bond Reductions

**DOI:** 10.1021/jacs.2c08559

**Published:** 2022-11-09

**Authors:** Bono van IJzendoorn, Saad F. Albawardi, Inigo J. Vitorica-Yrezabal, George F. S. Whitehead, John E. McGrady, Meera Mehta

**Affiliations:** † Department of Chemistry, University of Manchester, Oxford Road, Manchester, M13 9PL, U.K.; ‡ Inorganic Chemistry Laboratory, Department of Chemistry, University of Oxford, Mansfield Road, Oxford, OX1 3QR, U.K.; § X-ray Diffraction Facility, University of Manchester, Oxford Road, Manchester, M13 9PL, U.K.

## Abstract

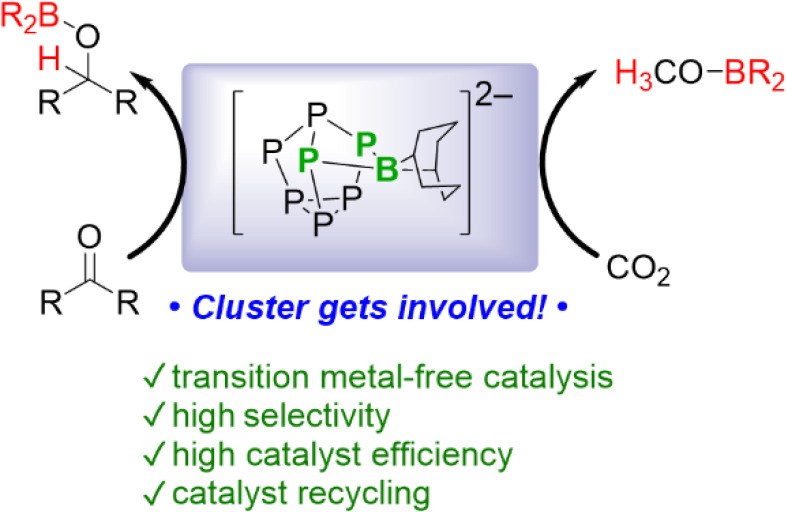

The first fully characterized boron-functionalized heptaphosphide Zintl cluster, [(BBN)P_7_]^2–^ ([**1**]^2–^), is synthesized by dehydrocoupling [HP_7_]^2–^. Dehydrocoupling is a previously unprecedented reaction pathway to functionalize Zintl clusters. [Na(18-c-6)]_2_[**1**] was employed as a transition metal-free catalyst for the hydroboration of aldehydes and ketones. Moreover, the greenhouse gas carbon dioxide (CO_2_) was efficiently and selectively reduced to methoxyborane. This work represents the first examples of Zintl catalysis where the transformation is transition metal-free and where the cluster is noninnocent.

## Introduction

Methanol (CH_3_OH) is a clean fuel and a highly important raw material for chemical industries.^1−3^ Over half of the world’s methanol is upcycled into everyday products, including pharmaceuticals, adhesives, agrochemicals, and paints/coatings. Producing methanol from carbon dioxide (CO_2_) has attracted global attention, because it converts a greenhouse gas into a resource that can re-enter the energy cycle. This approach is “two birds one stone” in contributing to global climate control and sustainable energy efforts.^4,5^ On an industrial scale, the conversion of atmospheric CO_2_ into methanol was first realized in 2012 by the George Olah Plant (Iceland), which produces up to 4500 m^3^ of methanol per year.^6^ The intrinsic stability of CO_2_ means that catalysts are essential for efficient reduction, and these are often based on expensive metals.^7^ The large-scale development and utilization of CO_2_ reduction necessarily requires any process to be sustainable and cost-effective, and so identifying less expensive and sustainable alternatives to these metals is an important target.

Heterogeneous phosphorus-containing materials represent one possible alternative to transition metals and are currently being explored for CO_2_ reduction.^8,9^ The synthesis of well-defined molecular analogues of these phosphorus materials offers the opportunity for mechanistic investigation, and molecular clusters offer an important middle ground between molecules and bulk solids. Zintl clusters, in particular, can be thought of as molecular mimics for heterogeneous materials:^10^ for example, the structure of [P_7_]^3–^ can be viewed as a fragment of red phosphorus,^11−14^ which is an inexpensive and abundant material, albeit a challenging one to study because of its poor solubility. In contrast, [P_7_] cages, especially those that have been functionalized, are soluble in common laboratory solvents, offering a broader range of handles for *in situ* investigation. An improved understanding of reactivity patterns for [P_7_] could be extended to corresponding reactions with materials based on the many allotropes of phosphorus.

**Figure 1:**
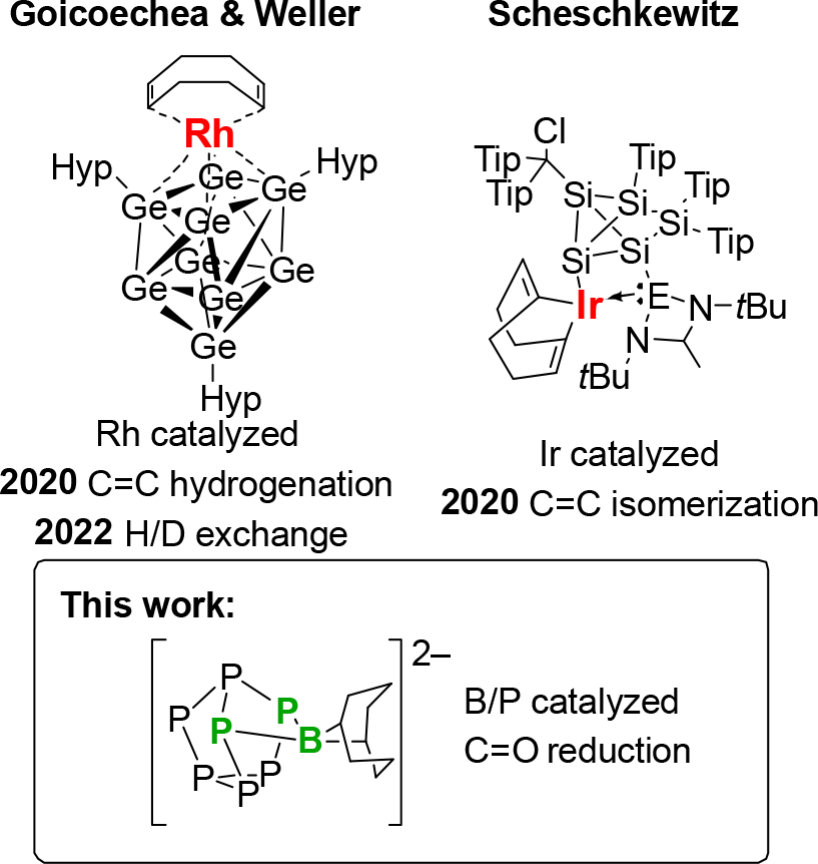
Zintl cluster catalysts. Hyp = Si(SiMe_3_)_3_, Tip = 2,4,6-triisopropylphenyl, E = Si, Ge, or Sn.

Only a small number of catalytic applications involving Zintl-derived clusters have been reported, where the cluster typically acts as a spectator ligand, supporting active rhodium or iridium centers^10,15,16^ (Figure 1) Catalysis of the reverse water–gas shift reaction by a Ru/Sn Zintl cluster, [Ru@Sn_9_]^6–^, has been reported in the recent literature, although the degree to which the cluster remains intact when deposited on a CeO_2_ surface remains to be established.^17^

The reaction chemistry of clusters based on the [P_7_] framework is an emerging field,^11,12,18^ but salt metathesis with group 14 electrophiles has already proven to be a powerful route to functionalizing the cluster. Further, in 2012, Goicoechea and co-workers reported the use of the protonated heptapnictide clusters [HPn_7_]^2–^ (Pn = P, As) in hydropnictination reactions with carbodiimides and isocyanates.^19−21^ They also reported that the [Pn_7_]^3–^ (Pn = P, As) cluster reacted with alkynes to afford 1,2,3-tripnictolides,^22,23^ while reaction with carbon monoxide afforded the [PCO]^−^ anion.^24^ In 2021, we reported that the trisilylated derivatives (R_3_Si)_3_P_7_ (R = Me, Ph) captured 3 equiv of heteroallene and underwent subsequent small-molecule exchange reactions.^25^

Herein we report that Zintl clusters based on the [P_7_] architecture are catalytically competent for borohydride reductions. Specifically, we prepare the transition metal-free functionalized heptaphosphide Zintl cluster shown in Figure 1 and establish its ability to catalyze the reduction of C=O bonds. Our initial focus is on the reduction of organic aldehydes and ketones, where the progress of reactions can readily be monitored via NMR spectroscopy, and then move on to heteroallenes where two double bonds are present. This then leads naturally to a survey of CO_2_ hydroboration, where complete selectivity to methoxyborane is observed. This Zintl catalyst displays turnover numbers, turnover frequencies, recyclability, and selectivity that are competitive with main group catalysts reported in the literature, under mild conditions.

## Results and Discussion

### Synthesis of Catalyst and Stoichiometric Studies

**Scheme 1:**
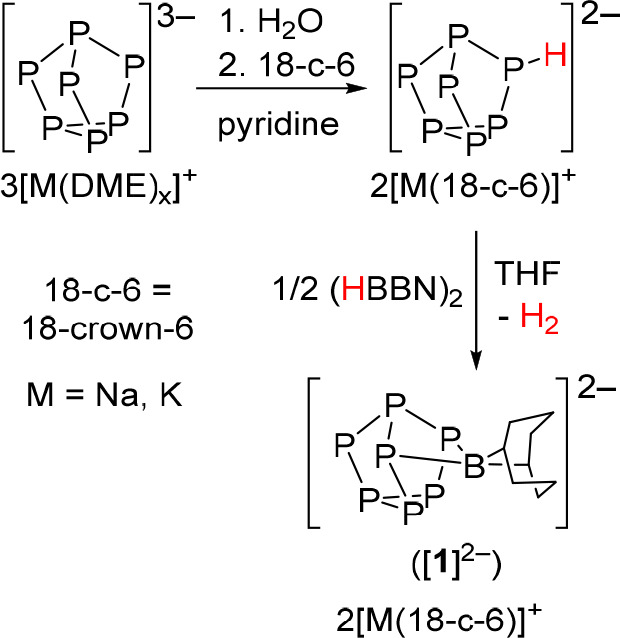
Synthesis of [M(18-c-6)]_2_[**1**]

Inspired by advances made in molecular frustrated Lewis pair (FLP) chemistry,^26−33^ the synthesis of boron-functionalized group 15 Zintl clusters capable of C=O bond reductions was targeted. First, using literature protocols the [P_7_]^3–^ salt^34^ and [HP_7_]^2–^ salt^19^ were prepared. The [HP_7_]^2–^ anion was then reacted with the 9-borabicyclo[3.3.1]nonane dimer (HBBN dimer) to give [M(18-c-6)]_2_[(BBN)P_7_] ([M(18-c-6)]_2_[**1**]) as either the sodium or potassium salt, shown in Scheme 1, along with the elimination of H_2_: gas formation could be observed during these reactions. This dehydrocoupling chemistry has no precedent in current synthetic strategies toward functionalizing Zintl clusters. Salt metathesis reactions using BBNOTf (OTf = triflate), Cy_2_BI, or Cy_2_BOTf with [P_7_]^3–^ did not result in functionalization of the cluster, but instead gave NMR spectra consistent with decomposition of the cluster.

**Figure 2:**
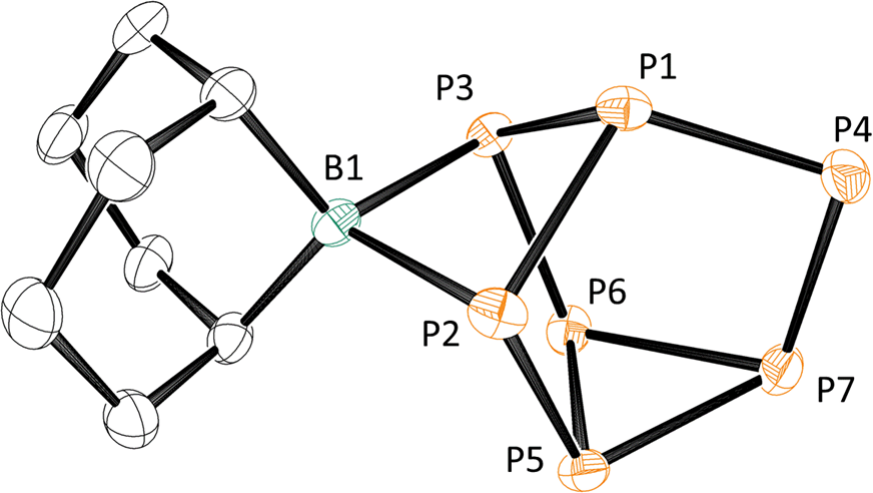
Molecular structure of [(BBN)P_7_]^2–^ ([**1**]^2–^) in the [Na(18-c-6)]_2_[(BBN)P_7_] salt. Anisotropic displacement ellipsoids pictured at 50% probability. Hydrogen atoms, THF solvent molecules, and [Na(18-c-6)]^+^ countercations omitted for clarity. Phosphorus: orange; boron: green; carbon: white. Selected bond length [Å]: B1–P2 2.052(8), B1–P3 2.082(7), P1–P2 2.213(2), P1–P3 2.216(2), P1–P4 2.128(3), P2–P5 2.182(2), P3–P6 2.185(2), P4–P7 2.136(2), P5–P6 2.235(2), P5–P7 2.236(3), P6–P7 2.218(2); selected bond angles [deg]: P2–B1–P3 93.3(3).

Nuclear magnetic resonance (NMR) spectroscopy studies of [M(18-c-6)]_2_[**1**] revealed five resonances in the ^31^P NMR spectrum, each exhibiting extensive P–P coupling, along with a single relatively sharp resonance in the ^11^B NMR spectrum at 11.14 ppm (Supporting Information, Figures S2 and S4). These spectroscopic features are consistent with the [P_7_] cage having a mirror plane and κ^2^-coordination of the BBN moiety to the cluster. Similar ^31^P NMR spectroscopic features were reported for the structurally related [(Ph_2_In)P_7_]^2–^ cluster.^35^ Single-crystal X-ray diffraction (XRD) studies were performed on both sodium and potassium salts [Na(18-c-6)]_2_[**1**] and [K(18-c-6)]_2_[**1**]. In both cases, consistent with the spectroscopic data, the BBN moiety in [**1**]^2–^ is coordinated to the [P_7_] fragment in a κ^2^-coordination mode (Figure 2). The average B–P bond length of 2.072 Å in[**1**]^2–^ is somewhat longer than B–P bonds in typical borylphosphines (R_2_P–BR_2_, 1.889–1.953 Å) and considerably longer than those in typical phosphinoborenes (R_2_P=BR_2_, 1.762–1.857 Å), where P=B π bonding is significant. The sum of the angles around phosphorus is 276°, also typical of borylphosphines (284–328°) rather than phosphinoborenes (359.8–328.3°).^36,37^

All efforts to react [Na(18-c-6)]_2_[**1**] directly with H_2_ were unsuccessful. However, stoichiometric reactions of [Na(18-c-6)]_2_[**1**] with carbonyls, including benzaldehyde and acetophenone, and heteroallenes such as phenyl isocyanate and CO_2_, resulted in an immediate color change from orange to red. Unfortunately, and despite multiple efforts, XRD diffraction quality crystals could not be obtained from any of these reactions, but *in situ*^31^P and ^11^B NMR spectroscopy confirmed the disappearance of [Na(18-c-6)]_2_[**1**], along with the formation of novel (asymmetric)^25^ functionalized clusters that would be consistent with formation of a carbonyl or heteroallene adduct (see Supporting Information, Figures S126–S138). We note that others have previously isolated and crystallographically characterized adducts of carbonyls or heteroallenes with FLPs where the C–O bond bridges the B···P gap.^38−41^ The clear color change and disappearance of [Na(18-c-6)]_2_[**1**] in these carbonyl and heteroallene reactions but not with H_2_ follows established reactivity patterns for borylphosphines^42^ rather than phosphinoborenes.^43^

### Catalytic Reduction of Carbonyls

[M(18-c-6)]_2_[**1**] salts (M = Na, K) were then explored for their potential as catalysts in the reduction of carbonyls, a transformation that is widely employed by the pharmaceutical, agrochemical, polymer, and fine chemical industries.^44^ Initially the reduction of benzaldehyde and benzophenone was targeted (Table 1). In tetrahydrofuran (THF) with 5 mol % [Na(18-c-6)]_2_[**1**] catalyst loading and the mild reductant pinacolborane (HBpin), the hydroboration of benzaldehyde (**2a**) and benzophenone (**3a**) was quickly achieved at room temperature (RT) to give the benzyloxyboranes **2b** and **3b** in high yields. Lowering the catalysts loading under the same conditions decreased the conversion, but it was found that a change of solvent to *ortho*-difluorobenzene (*o*DFB) improved the yield, giving near quantitative conversions even at 1 mol % catalyst loadings, under similar conditions. Hydrosilylation of the carbonyls using triethylsilane and triphenylsilane was also tested, but no reactions were observed. Changing the countercation from sodium to potassium did not affect catalyst performance. Control reactions confirmed that catalyst [M(18-c-6)]_2_[**1**] was necessary and also that the unfunctionalized K_3_P_7_ salt was completely inactive in this hydroboration. The [Na(18-c-6)]_2_[HP_7_] salt was also tested as a precatalyst and displayed lower catalytic performance compared to [M(18-c-6)]_2_[**1**].

**Table 1:**
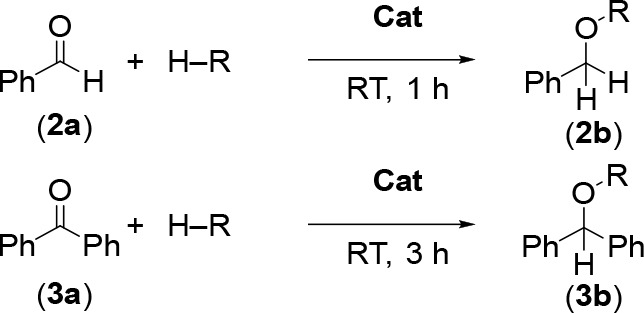
Reaction Condition Optimization for the Hydroboration of Carbonyls

catalyst (mol %)	*T*, °C	H–R	solvent	**2b** conv (%)^a^	**3b** conv (%)^a^
[Na(18-c-6)]_2_[**1**] (5)	RT	HBpin	THF	92	80
[Na(18-c-6)]_2_[**1**] (1)	RT	HBpin	THF	76	48
[Na(18-c-6)]_2_[**1**] (1)	RT	HBpin	*o*DFB	>99(94)	>99(96)
[Na(18-c-6)]_2_[**1**] (1)	50	Et_3_SiH	*o*DFB	0	0
[Na(18-c-6)]_2_[**1**] (1)	50	Ph_3_SiH	*o*DFB	0	0
[K(18-c-6)]_2_[**1**] (1)	RT	HBpin	THF	75	52
[K(18-c-6)]_2_[**1**] (1)	RT	HBpin	*o*DFB	>99	>99
[Na(18-c-6)]_2_[HP_7_] (5)^b^	RT	HBpin	*o*DFB	69	60
K_3_P_7_ (5)	RT	HBpin	THF	0	0
[K(18-c-6)]_3_[P_7_] (5)	RT	HBpin	THF	0	0
none	RT	HBpin	THF	0	0

a Determined by ^1^H NMR spectroscopy. Isolated yields are given in parentheses.

b Precatalyst.

Using 1 mol % [Na(18-c-6)]_2_[**1**] in *o*DFB, the scope of aldehyde hydroborations was expanded to include the substrates shown in Table 2. Similar to the hydroboration of benzaldehyde (**2a**), hydroboration of 2-formyl pyridine (**4a**) showed quantitative conversion to **4b** after 30 min. In contrast, introducing electron-withdrawing groups in the 4-position of the benzaldehyde resulted in longer reaction times. In the case of (1,1′-biphenyl)-4-carbaldehyde (**5a**), 4-(pyridin-4-yl)benzaldehyde (**6a**), 4-(trifluoromethyl)benzaldehyde (**7a**) and 4-bromobenzaldehyde (**8a**), complete conversion to the hydroborated products **5b**–**8b** was obtained after 5–10 h. Electron-donating groups on the 2-position of the benzaldehyde (aldehydes **9a** and **10a**) also required longer reaction times to give complete conversion, presumably due to increased steric crowding. Unsaturated aliphatic substituted aldehydes **11a** and **12a** showed selective hydroboration of the C=O bond and yielded products **11b** and **12b**, respectively. Hydroboration of acetylaldehyde (**13a**) was found to result in a mixture of paraldehyde and the desired hydroborated product **13b**.

**Table 2:**
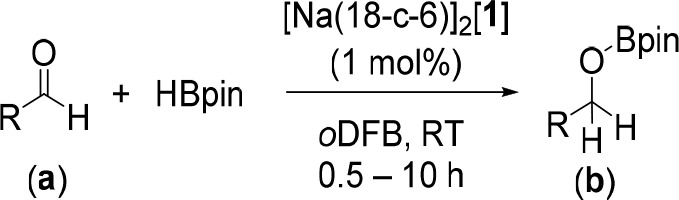

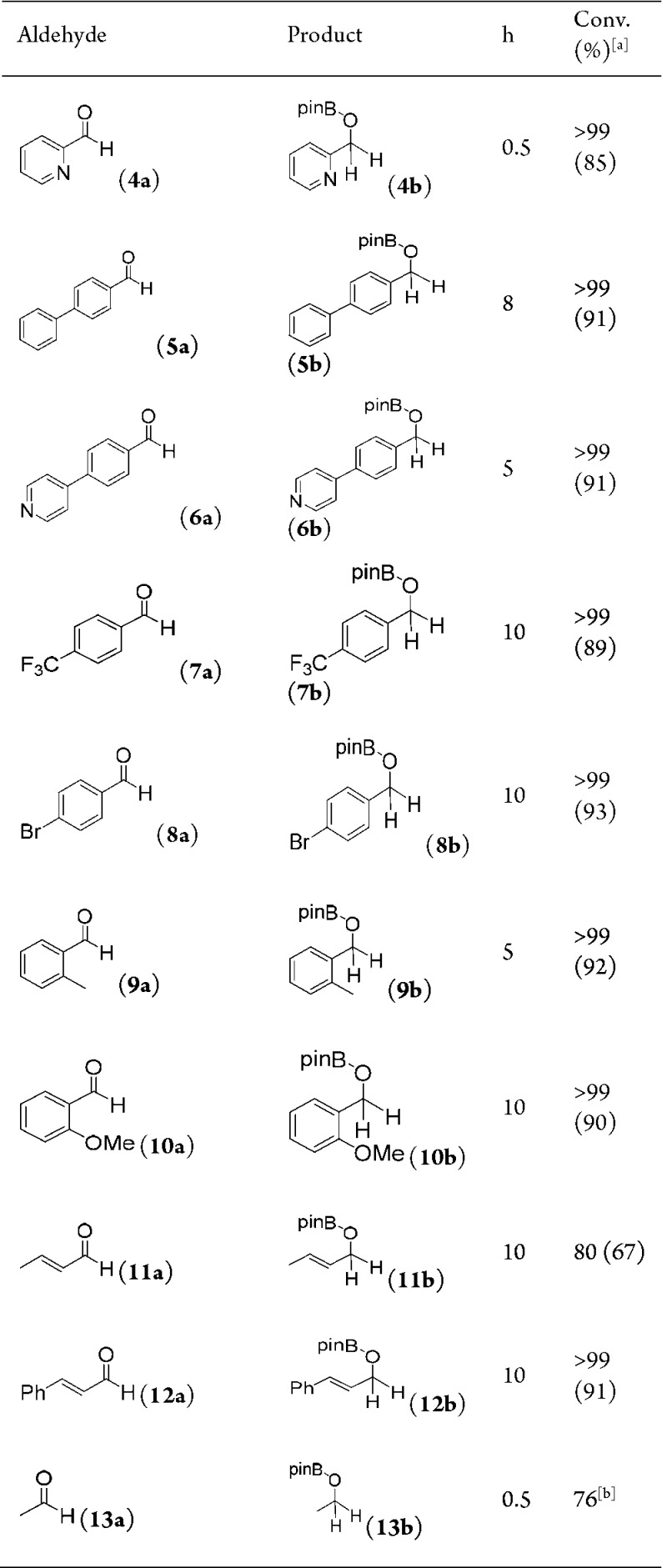
Catalytic Hydroboration of Aldehydes

a Determined by ^1^H NMR spectroscopy. Isolated yields are given in parentheses.

b 5 equiv of substrate was used because it quickly evaporates from the reaction mixture.

Next, the scope of ketone hydroborations was extended (Table 3). Substituting one phenyl group of benzophenone for a 2-pyridyl group (**3a** vs **14a**) leads to a significantly longer reaction time (3 vs 18 h, respectively). In contrast, the hydroboration of di(pyridyl) ketone (**15a**) only required 30 min to give complete conversion to **15b**. Coordination of the pyridyl group on the aldehyde to free HBpin could be a factor in determining the rate of these reactions; in support of this proposal, it was found that one of the pyridine nitrogens of **15b** coordinates to the Bpin boron center in its crystal structure (Supporting Information, Figure S66). Similar to the hydroboration of benzophenone (**3a**), acetophenone (**16a**) and thiophene-2-carboxaldhyde (**17a**) gave complete conversion to **16b** and **17b**, respectively, after 30 min.

**Table 3:**
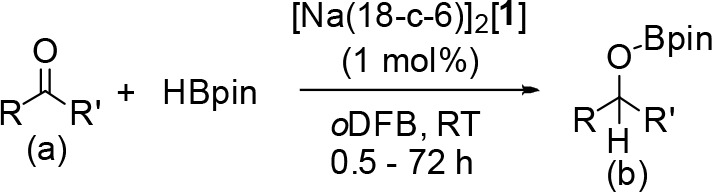

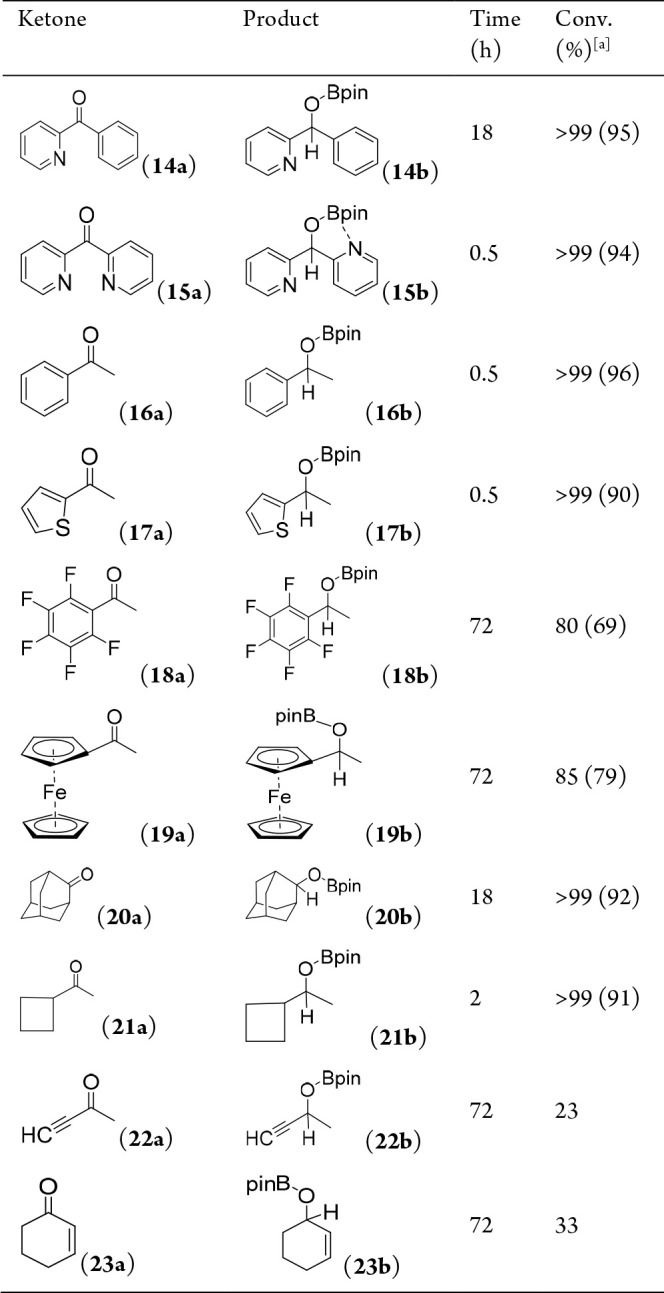
Catalytic Hydroboration of Ketones

a Determined by ^1^H NMR spectroscopy. Isolated yields are given in parentheses.

The incorporation of electron-withdrawing substituents resulted in longer reaction times, for example in the case of 1-(perfluorophenyl)ethan-1-one (**18a**) and acetylferrocene (**19a**), which required 72 h to give high conversions to **18b** and **19b**, respectively. The sterically bulky adamantanone (**20a**) gave **20b** after 18 h. The alkyl-substituted ketone cyclobutyl methyl ketone (**21a**) resulted in **21b** after 2 h. Introduction of ethynyl and alkenyl groups on the ketone (**22a** and **23a**) resulted in the formation of byproducts, decreasing the conversion to the desired hydroborated product.

**Scheme 2:**
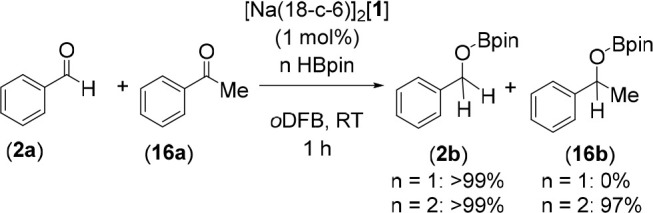
Competition Reaction between Benzaldehyde (**2a**) and Acetophenone (**16a**)

To investigate the chemoselectivity of carbonyl hydroborations, a competition reaction was carried out between benzaldehyde, **2a**, and acetophenone, **16a**, with 1 equiv of HBpin (Scheme 2). Exclusive hydroboration of benzaldehyde was observed under these conditions, but addition of a second equivalent of HBpin resulted in hydroboration of the acetophenone as well. Neither **2b** or **16b** was found to undergo deoxygenation in the presence of an excess of HBpin. The selectivity of reduction toward aldehydes in the presence of ketones is consistent with literature precedent^45−47^ and with the data in Table 1, which shows higher degrees of conversion for **2a** compared to **3a**.

### Catalytic Reduction of Heteroallenes

Encouraged by the catalytic hydroboration of carbonyls, we next extended the scope to heteroallenes, including both carbodiimides and isocyanates (Table 4). Three equivalents of HBpin was used in these transformations to probe the potential for deoxygenation or denitrogenation. Hydroboration of carbodiimide (^i^PrN)_2_C (**24a**) gave exclusively the mono-hydroborated product ^i^Pr(Bpin)NCHN^i^Pr (**24b**), with no evidence for bis-hydroborated or denitrogenated products even over prolonged reaction times and at elevated temperatures (50 °C). The same reaction with (CyN)_2_C (**25a**) also gave the mono-hydroborated product, (Cy(Bpin)N)CHNCy (**25b**), whereas the hydroboration of isocyanates PhNCO (**26a**) and CyNCO (**27a**) gave detectable mixtures of the mono-hydroborated (**26b** and **27b**), bis-hydroborated (**26c** and **27c**), and deoxygenated (**26d** and **27d**) products. Longer reaction times favored the deoxygenated products **26d** and **27d**, with 54–72% conversion obtained after 7 days.

**Table 4:**
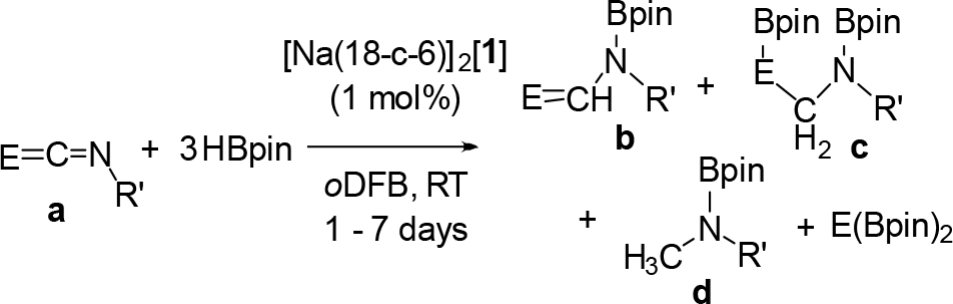

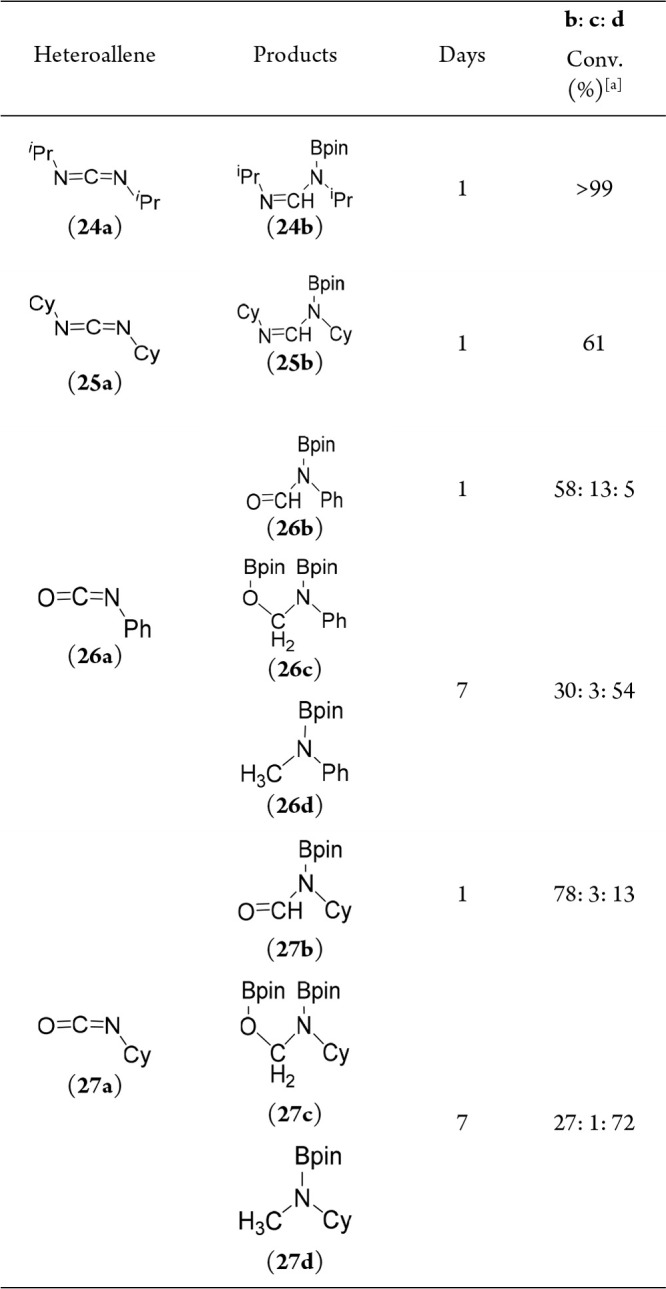
Hydroboration of Heteroallenes

a Determined by ^1^H NMR spectroscopy, based on C–H bond formation.

### Catalytic Reduction of CO_2_

Isocyanates (RN=C=O) are, of course, structurally related to the greenhouse gas CO_2_ (O=C=O), and so the successful deoxygenation of isocyanates to methyl amines motivated further investigations into the catalytic reduction of CO_2_. Molecular main group catalysts to convert CO_2_ to products such as formic acid (HCOOH), methanol (CH_3_OH), methane (CH_4_), and carbon monoxide (CO) have been previously reported,^48^ perhaps the best known of which are the FLP catalysts used to hydroborate or hydrosilylate CO_2_.^29,49^

Triethylsilane and triphenylsilane were tested in the catalytic reduction of CO_2_ and gave no conversion (Table 5), confirming that, as was the case for the carbonyl compounds, silanes are not suitable reducing agents to drive the reduction of CO_2_ using[Na(18-c-6)]_2_[**1**] as a catalyst. Next, 3 equiv of HBpin with 3.33 mol % [Na(18-c-6)]_2_[**1**] was pressurized with 1 atm of CO_2_ at RT and found to give a mixture of products. In good agreement with literature reported chemical shifts,^50^ the formic acid (Table 5, **b**), acetal (Table 5, **c**), methanol (Table 5, **d**), and methane (Table 5, **e**) oxidation levels could be identified in the reaction mixture. Using 3.33 mol % [Na(18-c-6)]_2_[**1**], common borane reductants and solvents were screened (see Supporting Information Section 5.1). Catecholborane (HBcat) and borane dimethylsulfide (BH_3_·SMe_2_) did not result in detectable amounts of product, but when the HBBN dimer, which has pinned back aliphatic groups and an accessible hydride, is used as the reductant, the conversion is 95% with a product distribution of 1:9 formylborane (**28b**):methoxyborane (**28d**). Control experiments confirmed that the naked [P_7_]^3–^ clusters (as K or K(18-c-6) salts) or HBBN dimer as catalyst is not independently catalytically active (Table 5, entries 7–9). The tris-functionalized P_7_ cluster (Me_3_Si)_3_P_7_ was also prepared^34^ and found to be catalytically inactive (Table 5, entry 10). These controls confirm that the BBN moiety and P_7_ cluster of [**1**]^2–^ cooperate to enable catalytic activity.

**Table 5:**
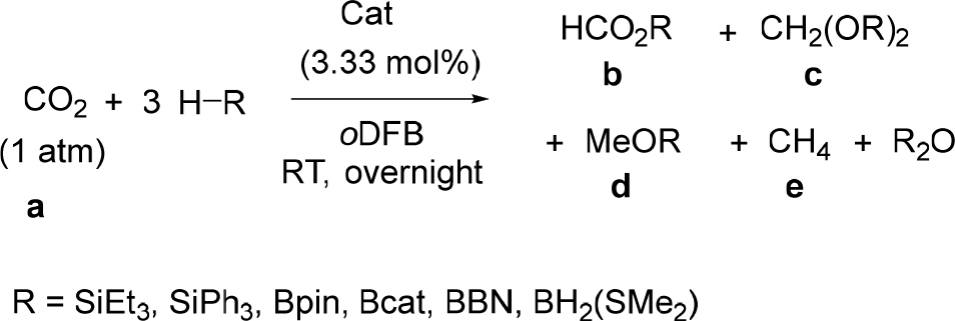
Screening Conditions for CO_2_ Reduction

entry	catalyst	H–R	**b** conv (%)^a^	**c** conv (%)^a^	**d** conv (%)^a^	**e** conv (%)^a^
1	[Na(18-c-6)]_2_[**1**]	Et_3_SiH	0	0	0	0
2	[Na(18-c-6)]_2_[**1**]	Ph_3_SiH	0	0	0	0
3	[Na(18-c-6)]_2_[**1**]	HBpin	3	1	31	10
4	[Na(18-c-6)]_2_[**1**]	HBcat	0	0	0	0
5	[Na(18-c-6)]_2_[**1**]	BH_3_·SMe_2_	0	0	0	0
6	[Na(18-c-6)]_2_[**1**]	(HBBN)_2_	11	0	84	0
7	none	(HBBN)_2_	0	0	0	0
8	K_3_P_7_	(HBBN)_2_	0	0	0	0
9	[K(18-c-6)]_3_P_7_	(HBBN)_2_	0	0	0	0
10	(Me_3_Si)_3_P_7_	(HBBN)_2_	0	0	0	0

a Determined by ^1^H NMR spectroscopy, based on C–H bond formation.

The effect of varying reaction conditions with the HBBN dimer as the reductant is summarized in Table 6. Reducing the catalyst loading from 3.33 mol % to 0.33 mol % increased the selectivity toward methoxyborane (MeOBBN, **28d**) to >99%, while the introduction of toluene as a cosolvent improved solubility of (HBBN)_2_ and thus increased the turnover frequency (TOF). Increasing the temperature from RT to 50 °C further increased the TOF to 300 while lowering the catalyst loading to 0.01 mol %, giving the maximum turnover number (TON) of 9800. Cluster decomposition is observed above 50 °C, limiting catalyst screening conditions at high temperatures. The maximum TON and TOF (Table 6, entries 3 and 5) are high compared to previously reported main group catalysts for this transformation (see Supporting Information Table S7).

**Table 6:**
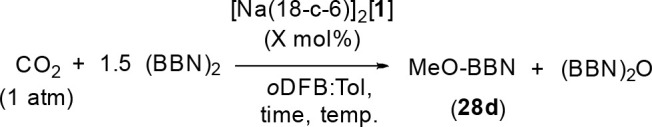
Catalytic Hydroboration of CO_2_ to MeOBBN

entry	[Na(18-c-6)]_2_[**1**] (X mol %)^a^	time (h)	*T* (°C)	**28d** conv (%)^b^	TON^b^	TOF (h^–1^)^b^
1^c^	0.33	12	RT	>99	300	25
2	0.33	10	RT	>99	300	30
3	0.33	1	50	>99	300	300
4	0.1	32	RT	>99	1000	31
5	0.01	480	RT	98	9800	20
6	0.01	40	50	95	9476	237

a Relative to B–H bonds.

b Determined by ^1^H NMR spectroscopy, based on C–H bond formation.

c Alternative conditions: only *o*DFB as solvent.

Boron–phosphorus FLP systems reported by Fontaine and Stephan also show excellent activity with TONs between 2950 and 5556 and TOFs between 176 and 853 h^–1^.^51,52^ However, these catalysts required more forcing conditions (higher temperature and/or pressure) than the ones reported here. Under the mild conditions described here TOFs in the range 2.6–50 h^–1^ and TONs between 99 and 648 have been reported by Goicoechea, Datta and Mandal, Cantat, Song, and Ramos.^53−57^ Meaningful comparisons can be made between [Na(18-c-6)]_2_[**1**] and Cantat’s P(MeNCH_2_CH_2_)_3_N catalyst.^58^ Both show similar maximum TOFs ([Na(18-c-6)]_2_[**1**]: 237 h^–1^; P(MeNCH_2_CH_2_)_3_N: 287 h^–1^), but significantly higher maximum TONs can be achieved with catalyst [Na(18-c-6)]_2_[**1**] ([Na(18-c-6)]_2_[**1**]: 9800; P(MeNCH_2_CH_2_)_3_N: 6043).

In addition to the excellent TON observed, catalyst [Na(18-c-6)]_2_[**1**] was found to be surprisingly robust: after complete catalytic hydroboration of CO_2_ to methoxyborane (**28d**) no decomposition of [Na(18-c-6)]_2_[**1**] was detected by NMR spectroscopy. To investigate whether catalyst [Na(18-c-6)]_2_[**1**] could be recycled, the reaction sample was reloaded with 3 equiv of HBBN and 1 atm of CO_2_. Again, at RT, complete conversion to **28d** was observed overnight. Catalyst [Na(18-c-6)]_2_[**1**] was recycled a total of seven times with no loss of catalyst activity, consistent with living catalysis. After these cycles, water hydrolysis of methoxyborane gave methanol in complete conversion: 0.62 mmol of methanol was produced using 0.9 μmol of catalyst (a 689-fold excess of methanol).

**Figure 3:**
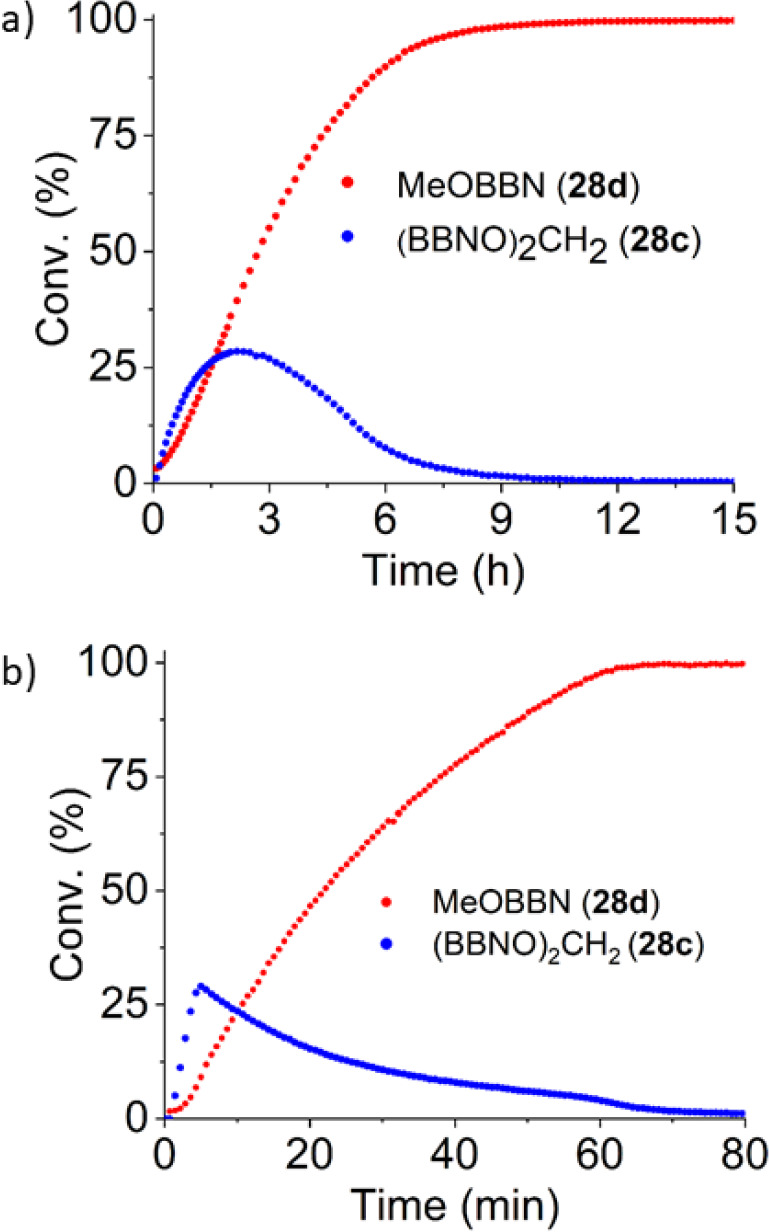
Tracked reaction: (a) reaction at RT; (b) reaction at 50 °C.

The CO_2_ hydroboration with HBBN dimer was monitored by ^1^H NMR spectroscopy with 0.33 mol % [Na(18-c-6)]_2_[**1**] at room temperature and 50 °C (Table 6, entries 2, 3). In both cases, intermediate CH_2_(OBBN)_2_ (**28c**) quickly formed and then was consumed (Figure 3). Meanwhile **28d** is formed gradually throughout the reaction. In line with literature reports, the intermediate formyl-BBN (**28b**) was not detected under these conditions and is presumed to be significantly more reactive toward hydroboration compared to CO_2_, **28c**, and **28d**.

Stoichiometric reduction of CO_2_ using equimolar [Na(18-c-6)]_2_[**1**] and HBBN exclusively gave HC(O)OBBN (**28b**). Further, reaction of [Na(18-c-6)]_2_[**1**] with 2 equiv of HBBN under a CO_2_ atmosphere again exclusively gave **28b**, indicating that CO_2_ is not trapped and reduced to **28b** then further reduced to **28c** and **28d** while intact on the catalyst. Rather, the evidence suggests that **28b** is released after one addition of borane but then re-enters the catalytic cycle to form **28c** and **28d**.

Mimicking catalytic conditions, **28b** could be independently prepared *in situ* by addition of an excess of HBBN to formic acid in oDFB/toluene over 20 h. After generation of **28b**, there is no further reaction with excess HBBN. However, when [**1**]^2–^ was added to the reaction mixture, formation of **28c** and **28d** was observed. This observation is consistent with (1) the need for [**1**]^2–^ in the reduction of **28b** and (2) **28b** being an intermediate in CO_2_ hydroboration and not a side product.

### Mechanistic Investigations

**Figure 4:**
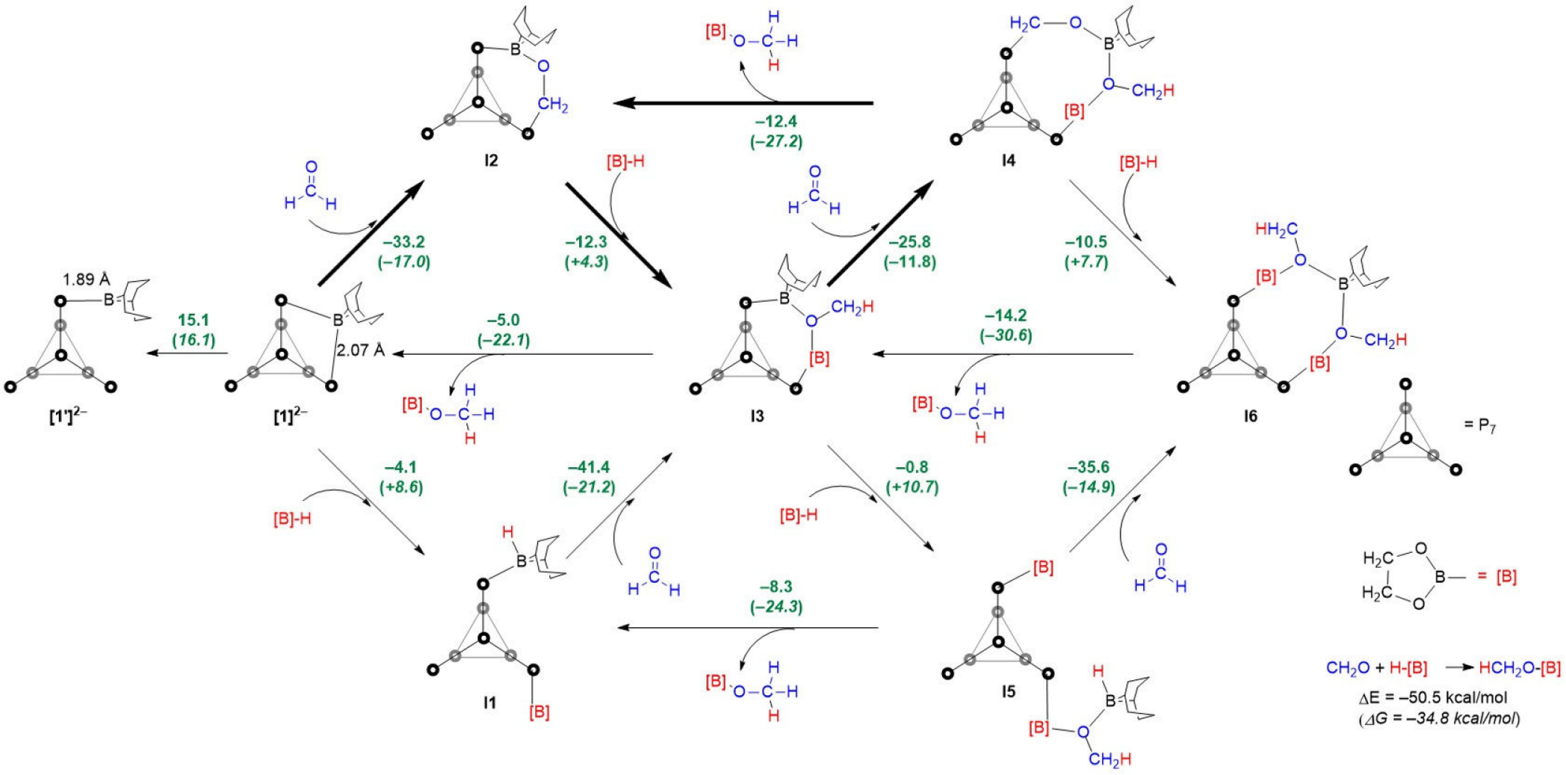
Energies and zero-point-corrected free energies (in parentheses) of possible steps on the [**1**]^2–^-catalyzed reduction pathway of H_2_C=O with HBpin. All calculations were performed at the wB97XD, def2-TZVP level. All energies are given in kcal/mol. The energies in each of the six triangles sum to the overall energy of CH_2_O + H–[B] → HCH_2_O–[B], and each therefore constitutes a viable catalytic cycle. Based on the experimental evidence supporting the presence of **I2** and **I4**, we favor the cycle highlighted with bold arrows as the dominant one (see text for more detailed discussion).

In order to probe the mechanistic landscape for the reduction of C=O functional groups, we have performed DFT calculations on the simplest model substrate, formaldehyde (Figure 4). The optimized structure of [**1**]^2–^ reproduces the crystallographic data with good accuracy: the optimized B–P bond lengths are 2.07 Å (vs 2.052(8) and 2.082(7) Å in Figure 2), while P1–P2 and P1–P3 are 2.21 Å vs crystallographic values of 2.213(2) and 2.216(2) Å, respectively. In its equilibrium structure, the boron center in [**1**]^2–^ is saturated by two B–P bonds, but a wider survey of the potential energy surface reveals a second shallow local minimum only 15.1 kcal/mol above the equilibrium structure, where the BBN fragment is coordinated to only one of the two phosphorus centers (isomer [**1′**]^2–^, Figure 4). In this case the single B–P bond length is 1.89 Å, precisely in the range for borylphosphines.

In the mechanistic scheme shown in Figure 4, a number of possible intermediates that are related by the addition of [B]H or formaldehyde or by the release of the product MeO[B] are identified. The changes in energy and zero-point-corrected free energy for the overall reaction H_2_CO + [B]H → MeO[B] are −50.5 and −34.8 kcal/mol, respectively, and the energies around each of the six triangles shown in Figure 4 sum to exactly these values. The cycle in the top left of the figure involves exothermic addition of H_2_CO to [**1**]^2–^ to form **I2**, followed by marginally exothermic addition of H[B] to form the methoxy derivate, **I3**, where the OMe group bridges the two boron centers. Loss of the product to regenerate [**1**]^2–^ is then moderately exothermic (−5.0 kcal/mol) but strongly favored on the free energy scale (−22.1 kcal/mol). The alternative pathway via initial activation of the borohydride (lower left triangle in Figure 4) proceeds via a marginally exothermic first step followed by a very exothermic binding and reduction of formaldehyde (Δ*E* = −41.4 kcal/mol). Both cycles, [**1**]^2–^ + CH_2_O + [B]H → **I1** or **I2** → **I3** → [**1**]^2–^ + MeO[B], therefore present a plausible cascade leading from reactants to products. We note, however, that only one of the two B–P bonds has been activated in **I3**, leaving open the possibility of a further series of reactions involving reaction of **I3** with second equivalents of formaldehyde and borohydride to form **I6** (via **I4** or **I5** depending on the order of addition) followed by release of MeO[B] to regenerate **I3**. The energies of the various steps in the cycle **I3** + CH_2_O + [B]H → **I4** or **I5** → **I6** → **I3** + MeO[B] are strikingly similar to those for the original cycle ([**1**]^2–^ + CH_2_O + [B]H → **I1** or **I2** → **I3** → [**1**]^2–^ + MeO[B]), other than a marginal decrease in exothermicity of the steps that involve binding of substrate and an increased exothermicity of the final release of product, both of which reflect the greater steric crowding as more molecules are assembled around the catalyst. Nevertheless, it is clear that the second B–P bond in **I3** remains capable of binding further substrate molecules.

**Figure 5:**
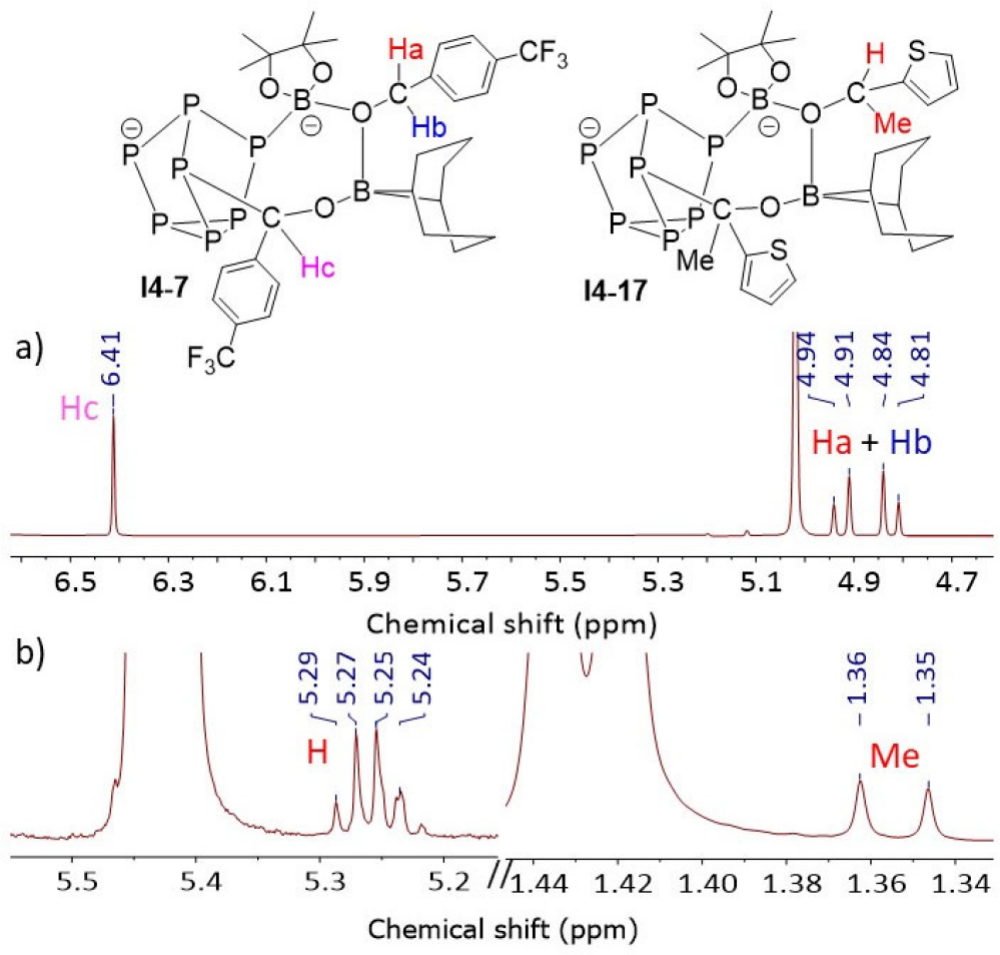
^1^H NMR spectroscopic monitoring of hydroboration of (a) **7a** and (b) **17a**.

Some support for the presence of **I4** in solution comes from monitoring of the hydroboration of **5a**, **7a**, **11a**, **14a**, and **17a** (see Supporting Information, Section 6.4). ^1^H NMR studies on the hydroboration of **5a**, **7a**, and **11a** reveal the presence of a second-order AB spin system with similar chemical shifts and coupling as the hydroborated products (selected spectra, Figure 5a). The AB spin system is consistent with the presence of a pair of enantiotopic protons displaying a 10–12 Hz geminal coupling, an assertion that is further supported by ^1^H correlated NMR spectroscopy (COSY) studies (Supporting Information, Figure S142). ^1^H diffusion-ordered NMR spectroscopy (DOSY) studies on the hydroboration of **7a** (Supporting Information, Figure S143) confirmed that the species giving rise to the AB spin system has the lowest diffusion coefficient, indicating that it is the largest species present in the reaction mixture, in line with the proposed structure for **I4** given in Figure 4. In contrast, the analogues of **I4** detected from the hydroboration of the ketones **14a** and **17a**, where only a single proton is present, display similar resonances but without further geminal coupling (selected spectra, Figure 5b). The characteristic resonances for **I4** are observed only during the reaction and disappear when the reaction is complete. Moreover, addition of 500 equiv of **7b** to *in situ* generated **I2** did not result in formation of **I4**, confirming the irreversibility of the **I4** → **I2** step.

In contrast, a direct spectroscopic signature of **I2** could not be identified in any of the reactions with aldehydes or ketones. However, we note that insertion of carbonyl groups into P–B bonds has precedent in the work by Fontaine and Ramos, where C–O bonds in the crystallographically characterized products are typically in the range 1.39–1.40 Å.^57^ The optimized structure of **I2** (Supporting Information, Figure S165) shows a similarly activated C–O bond (C–O = 1.37 Å, B–O = 1.47 Å), along with a short P–C bond length of 1.89 Å. Moreover, during stoichiometric CO_2_ capture experiments with [Na(18-c-6)]_2_[**1**] using ^13^C-labeled CO_2_, we observe a doublet at 194.13 ppm with ^1^*J*_CP_ = 49 Hz in the ^13^C{^1^H} NMR spectrum. A C–P coupling constant of this magnitude is qualitatively consistent with the presence of a direct C–P bond as in **I2** (or indeed **I4**), and the DFT-computed value (wB97XD/def2-TZVP) in the analogue of **I2** with CO_2_ (see Supporting Information, Figure S170) rather than formaldehyde as substrate is 41 Hz.

The presence of significant concentrations of **I4** would, in principle, also opens up the possibility of a third cycle, **I2** + CH_2_O + [B]H → **I3** → **I4** → **I2** + MeO[B] (Figure 4, top, center), where the total exothermicity of −50.4 kcal/mol is distributed evenly across the borohydride binding, formaldehyde binding, and product release steps (−12.3, −25.8, and −12.4 kcal/mol, respectively). In their recent study of CO_2_ reduction using Ph_2_PCH_2_CH_2_BBN, Ramos et al. have argued that trapping of CO_2_ by the B/P FLP leads to formation of a formaldehyde adduct analogous to **I2**,^57^ which, in fact, acts as the active catalyst in the dominant CO_2_ reduction cycle. By analogy, [**1**]^2–^ would then be a precursor, lying outside the main cycle, while **I2** is the active catalyst. Musgrave and co-workers have highlighted the “anticatalytic” role of the Lewis base in frustrated Lewis pairs:^59^ the Lewis base, in binding to the nucleophilic site (the carbonyl carbon here), reduces its susceptibility to subsequent attack by the reducing agent. In the present context, the very strong binding of formaldehyde to [**1**]^2–^ (−33.2 kcal/mol) will reduce its susceptibility to attack by borohydride, and so the somewhat weaker binding of the second aldehyde (**I3** + H_2_CO → **I4**, −25.8 kcal/mol) may accelerate its subsequent reduction by borohydride. The computed energetics, combined with the spectroscopic evidence for the presence of **I2** and **I4** in solution and the literature precedent for species like **I2** to act as catalysts, leads us to propose that this cycle is likely to dominate the reaction. At this point, however, we offer an important caveat, that the binding of the carbonyl group is strongly dependent on the size of the R and R′ groups: the −33.2 kcal/mol computed for the binding of H_2_CO to [**1**]^2–^ is reduced to −25.2 kcal/mol for benzaldehyde (PhCHO). The energetics in Figure 4 for the simplest model aldehyde, formaldehyde, therefore offer an overview of potential intermediates on the mechanistic landscape, but should not be taken to apply quantitatively to any of the experimental data summarized in Tables 1–3.

**Scheme 3:**
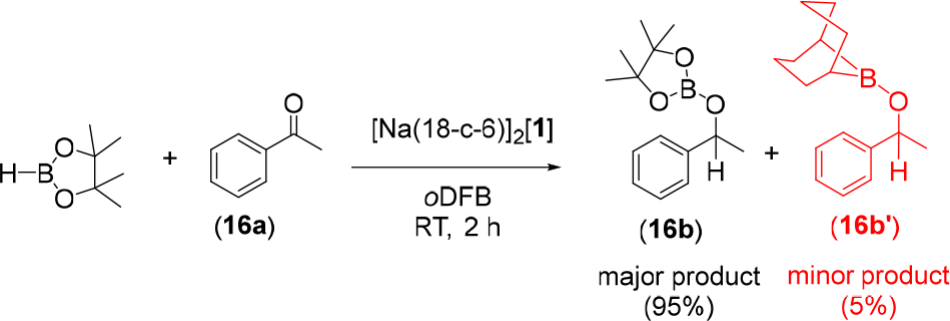
Stoichiometric Hydroboration of Acetophenone

Stoichiometric studies between [Na(18-c-6)]_2_[**1**], acetophenone, and HBpin confirmed (Scheme 3) compound **16b** to be the major product, but its BBN analogue **16b′** was also detected as a minor product in a 95:5 ratio. One possible source of the minor isomer is the methoxy-bridged intermediate, **I3** in Figure 4, where the oxygen is almost symmetrically bonded to both boron centers (B–O = 1.59 and 1.60 Å to the BBN and [B] groups, respectively). Cleavage of the two almost equivalent B–O bonds could then lead to either **16b** or **16b′**. At the wB97XD/def2-TZVP level, decomposition to give **16b′** (+ [B]–P_7_) was found to be less favorable by 15.1 kcal/mol (Δ*G* = −16.2 kcal/mol) than to **16b** (+ [**1**]^2–^), qualitatively consistent with the observed product distribution. The formation of **16b′** in the experiment does, however, indicate a 5% degradation of the catalyst under these conditions. Of course, a cluster functionalized with Bpin ([(Bpin)P_7_]^2–^) could also be catalytically active, following equivalent pathways to those shown in Figure 4, and a catalytic role for [(Bpin)P_7_]^2–^ would be consistent with our description of the [Na(18-c-6)]_2_[HP_7_] salt as a precatalyst in Table 1. We have not, however, been able to isolate or independently synthesize this species and test its catalytic performance, despite multiple attempts to do so. It is noteworthy that catalyst degradation via this route is not an issue when the HBBN dimer is used as a reducing agent, as it is in our experiments on CO_2_ hydroboration, simply because the analogue of **I3** is then symmetric.

The mechanism of the CO_2_ and heteroallene reduction reactions will be the subject of a further study, but it seems likely that, in their initial stages, at least, they will share many common features with the formaldehyde reaction shown in Figure 4. The geometries of the analogues of **I2** with CO_2_, HNCNH, and HC≡CCHO shown in Supporting Information, Figure S170, certainly show no significant differences in the binding mode of the substrate to [**1**]^2–^.

## Conclusion

In conclusion, a boron-functionalized group 15 Zintl cluster, [(BBN)P_7_]^2–^ ([**1**]^2–^), is prepared and found to be competent in the catalytic hydroboration of C=O bonds. Aldehydes, ketones, carbodiimides, isocyanates, and CO_2_ are all reduced, and, in the case of CO_2_ hydroboration, based on catalyst performance alone, [Na(18-c-6)]_2_[**1**] is competitive with other main group catalysts. Further, high selectivity to methoxyborane under mild conditions and catalyst recycling is established. This work represents the first application of a Zintl cluster in transition metal-free catalysis and establishes clearly that the cluster itself can play an active role in the catalytic cycle beyond that of a spectator ligand.

## Supporting Information Available

The general information, experimental procedures, characterization data, and computational details (PDF)

## Abbreviations

THF: tetrahydrofuran
RT: room temperature
HBpin: pinacolborane
HBBN: 9-borabicyclo(3.3.1)nonane
*o*DFB: *ortho*-difluorobenzene
XRD: X-ray diffraction
NMR: nuclear magnetic resonance
OTf: triflate
DFT: density functional theory
FLP: frustrated Lewis pair
COSY: correlation spectroscopy
DOSY: diffusion-ordered spectroscopy
