# Supplementary material for: A Zintl Cluster for Transition Metal-Free Catalysis: C=O Bond Reductions
Source: J Am Chem Soc. 2022 Nov 9;144(46):21213–23. doi: 10.1021/jacs.2c08559 (PMC9706568; doi:10.1021/jacs.2c08559)
Supplement: Supplementary file 1 — ja2c08559_si_001.pdf [file ja2c08559_si_001.pdf]

A Zintl Cluster for Transition Metal-Free Catalysis: C=O Bond Reductions

Bono van IJzendoorn,[a] Saad F. Albawardi,[b] Inigo J. Vitorica-Yrezabal,[c] George F. S. Whitehead,[c] John E. McGrady,*[b] Meera Mehta*[a]

a. Department of Chemistry, University of Manchester, Oxford Road, Manchester, M13 9PL, UK. E-mail: meera.mehta@manchester.ac.uk

b. Inorganic Chemistry Laboratory, Department of Chemistry, University of Oxford, Mansfield Rd, OX1 3QR, UK. E-mail: john.mcgrady@chem.ox.ac.uk

c. X-ray Diffraction Facility, University of Manchester, Oxford Road, Manchester, M13 9PL, UK.

Table of Contents

1. Methods and Materials 4
1.1. Experimental Considerations 4
1.2. Analytical Considerations 5
1.3. X-ray Diffraction Studies 6
1.4. NMR Spectra Simulations 7
2. Synthesis and Characterization of Catalysts 8
2.1. Synthesis [Na(18-c-6)]$_2$[BBN-P$_7$], [Na(18-c-6)]$_2$[1] 8
2.2. Synthesis [K(18-c-6)]$_2$[BBN-P$_7$], [K(18-c-6)]$_2$[1] 12
3. Catalytic Hydroboration of Ketones/Aldehydes 14
3.1. Reaction Conditions Screening 14
3.2. General Procedure for Hydroboration of Ketones/Aldehydes 15
3.2.1. Characterization Data Hydroboration of Aldehydes 18
3.2.2. Characterization Data Hydroboration of Ketones 35
3.3. Chemoselectivity Hydroboration of Aldehydes Versus Ketones 51
4. Catalytic Hydroboration of Carbodiimides and Isocyanates 53
4.1. General Procedure for Hydroboration of Carbodiimides and Isocyanates 53
4.1.1. Hydroboration of Diisopropyl Carbodiimide 53
4.1.2. Hydroboration of Dicyclohexyl Carbodiimide 54
4.1.3. Hydroboration of Phenyl Isocyanate 55
4.1.4. Hydroboration of Cyclohexyl Isocyanate 56
5. Catalytic Hydroboration of Carbon Dioxide 57
5.1. Solvent and Reductant Screening 57
5.2. Catalyst Loading 58
5.3. Catalyst Control Reactions 60
5.4. Isotopic Labelled Studies 61
5.5. Tracked Catalytic Hydroboration of CO_2 62
5.5.1. Tracked Reaction at 25 °C 62
5.5.2. Tracked Reaction at 50 °C 64
5.6. Catalyst Recycling 65
5.7. Hydrolysis Methoxyborane (28d) to Methanol 66
5.8. Comparison to Literature Metal-free Catalysts for CO_2 Hydroboration 67
6. Experimental Mechanistic Investigations 68
6.1. Addition H–[B] to [Na(18-c-6)]$_2$[1] 68
6.1.1. Addition (HBBN)$_2$ to [Na(18-c-6)]$_2$[1] 68
6.1.2. Addition HBpin to [Na(18-c-6)]$_2$[1] 72

6.2. Addition of Carbonyls to [Na(18-c-6)]$_2$[1] 73
6.2.1. Addition Benzaldehyde to [Na(18-c-6)]$_2$[1] 73
6.2.2. Addition Acetophenone to [Na(18-c-6)]$_2$[1] 74
6.3. Addition of Heteroallenes to [Na(18-c-6)]$_2$[1] 76
6.3.1. Addition phenyl isocyanate to [Na(18-c-6)]$_2$[1] 76
6.3.2. Addition CO_2 to [Na(18-c-6)]$_2$[1] 78
6.4. Selected NMR Hydroboration Aldehydes/Ketones 80
6.5. Stoichiometric Hydroboration of Acetophenone 85
6.6. Stoichiometric Hydroboration of CO_2 87
6.7. Hydroboration of formyl-BBN (28b) 89
7. Crystallography Tables 91
8. Density Functional Theory 93
8.1. Computational methods 93
8.2. Total energies (E and G) and optimized cartesian coordinates (Å) for all stationary points reported in the text. 93
9. References 111

1. Methods and Materials

1.1. Experimental Considerations

All manipulations were performed under an inert atmosphere using standard Schlenk-line, and glovebox techniques. Glassware was flame dried prior to use.

Dry THF, diethyl ether, toluene, and pentane were obtained using Innovative Technologies anhydrous engineering solvent purification systems and subsequently degassed. DME, pyridine, and hexane were dried over Na or K, purified by distillation. *o*DFB was dried over CaH_2 and purified by distillation. Pyr-d_5, THF-d_8, C_6D_6, were dried over activated 3 Å molecular sieves. All solvents were stored over activated 3 Å molecular sieves.

Red phosphorus, naphthalene, trimethylsilyl chloride, catechol borane, pinacol borane, HBBN dimer, borane dimethyl sulfide, triethyl silane, triphenyl silane, 18-crown-6, benzaldehyde, 2-pyridine-carboxaldehyde, 4-phenyl benzaldehyde, 4-(4-pyridyl) benzaldehyde, 4-trifluoromethyl benzaldehyde, 4-bromo benzaldehyde, 2-methyl benzaldehyde, 4-methoxy benzaldehyde, crotonaldehyde, cinnamaldehyde, acetylaldhyde, benzophenone, 2-pyridyl phenone, di(2-pyridyl)ketone, acetophenone, 2-acetylthiophene, 2-acetylpentafluorophenyl, 1-(pentafluorophenyl)ethanone, acetylferrocene, 2-adamantanone, 1-(cyclobutyl)ethanone, 1-(ethynyl)ethanone, cyclohex-3-enone, diisopropyl carbodiimide, dicyclohexyl carbodiimide, phenyl isocyanate, cyclohexyl isocyanate were purchased from a commercial source (Sigma-Aldrich, Alfa Aesar, Fuorochem, Tokyo Chemical Industry, Thermo Fisher Scientific, and Acros Organics) and used without purification. Elemental sodium and elemental potassium were cleaned by removal of the oxide layers and washing with toluene and hexane. $[Na(DME)_x]_3P_7$, K_3P_7, $[Na(18\text{-}c\text{-}6)]_2[HP_7]$ and $[K(18\text{-}c\text{-}6)]_2[HP_7]$ were synthesized using modified literature procedures.[1, 2] Carbon dioxide (cp grade, 99.95%) was purchased from BOC Ltd. Carbon-13 labelled carbon dioxide (99.0 atom% ^{13}C) was purchased from Sigma-Aldrich and was used using standard Schlenk-line techniques.

1.2. Analytical Considerations

NMR Spectroscopy. ^{1}H, ^{1}H COSY, ^{1}H DOSY, ^{11}B, $^{11}B\{^{1}H\}$, $^{13}C\{^{1}H\}$, $^{19}F\{^{1}H\}$, ^{31}P NMR and ^{31}P COSY spectra were recorded on a Bruker AVIII 400 spectrometer (operating frequencies: 399.78 MHz, 128.36 MHz, 100.53 MHz, 376.17 MHz, and 161.83 MHz for ^{1}H, ^{11}B, ^{13}C, ^{19}F, and ^{31}P, respectively). Variable temperature ^{31}P NMR spectra were recorded on a Bruker AVII 500 spectrometer (operating frequency: 202.46 MHz). Solid Sate ^{11}B NMR spectra were recorded on a Bruker AVIII 400 solid state spectrometer (operating frequencies: 128.36 MHz for ^{11}B). ^{1}H and $^{13}C\{^{1}H\}$ NMR chemical shifts were internally referenced to the residual solvent resonances (C_6D_6 (benzene-d_6): ^{1}H δ = 7.16 ppm, $^{13}C\{^{1}H\}$ δ = 128.02 ppm, THF-d_8 (tetrahydrofuran-d_8): ^{1}H δ = 3.58, 1.73 ppm, $^{13}C\{^{1}H\}$ δ = 67.57, 25.37 ppm, Pyr-d_5 (pyridine-d_5): ^{1}H δ = 8.74, 7.58, 7.22 ppm, $^{13}C\{^{1}H\}$ δ = 150.35, 135.91, 123.87 ppm. ^{11}B, ^{19}F, ^{31}P chemical shifts were externally referenced to $BF_3{\cdot}Et_2O$ $CFCl_3$, H_3PO_4, respectively. Solution phase NMR samples were prepared under an inert atmosphere in 5 mm J Young NMR tubes. Solid state NMR sample were prepared under an inert atmosphere in 4 mm rotors. 1D and COSY NMR data was analyzed using MestReNova V14.0.0 software or Topspin V3.6.1 software. DOSY NMR data was analysed using General NMR Analysis Toolbox (GNAT) V1.3.2 software.

Elemental Analysis. Elemental analysis was carried out by the microanalysis service of the University of Manchester using a Flash 2000 elemental analyser. Samples were prepared under a nitrogen atmosphere.

Mass spectrometry. Mass spectrometry (MS) samples were measured by the mass spectrometry service of the University of Manchester using an electrospray ionization (ESI) or atmospheric pressure chemical ionization (APCI) equipped Thermo Orbitrap Executive Plus Extended Mass Range mass spectrometer. Gas chromatography (GC) MS were measured using an Agilent GCMS system, 7890A series GC attached to 5975C electron ionisation (EI) quadrupole mass spectrometer. Samples were prepared under a nitrogen atmosphere and directly injected into the ionization source of the mass spectrometer unless otherwise stated.

Ultraviolet–visible spectroscopy. Ultraviolet–visible (UV-Vis) electronic absorption spectra were recorded on a Mettler Toledo UV5Bio spectrophotometer using 10 mm path length quartz cuvettes.

1.3. X-ray Diffraction Studies

Data collection: X-ray diffraction data were collected for compounds [Na(18-c-6)]$_2$[**1**], **15b**, and **28d'** at 100 K dual source Rigaku FR-X rotating anode diffractometer with a Hypix-6000HE detector and an Oxford Cryosystems nitrogen flow gas system. Data were measured with CrisAlisPro software. X-ray diffraction data for compound [K(18-c-6)]$_2$[**1**] was collected at 150 K using an Oxford Diffraction Supernova dual-source diffractometer equipped with a 135 mm Atlas CCD area detector. Crystals were quench-cooled using an Oxford Cryosystems open flow N_2 cooling device. All data were collected using Cu Kα radiation (λ = 1.5418 Å).

Crystal structure determination and refinements: X-ray data were processed and reduced using CrysAlisPro. Absorption correction was performed using empirical methods (SCALE3 ABSPACK) based upon symmetry-equivalent reflections combined with measurements at different azimuthal angles. The crystal structure was solved and refined against all F^2 values using the SHELX and Olex2 suite of programmes.[3, 4] All atoms were refined anisotropically. Hydrogen atoms were placed in calculated positions and refined using idealized geometries and assigned fixed isotropic displacement parameters. Atomic displacement parameters were restrained using a rigid body approach by applying SHELX RIGU commands and to be similar using SHELX SIMU commands. Disordered THF, toluene and 18-c-6 (18-c-6 = 18-crown-6) molecules in [Na(18-c-6)]$_2$[**1**] and [K(18-c-6)]$_2$[**1**] were modelled and refined over two positions.

Crystallographic data have been deposited with the CCDC (CCDC 2195882-2195885).

1.4. NMR Spectra Simulations

NMR spectra simulations were performed with the gNMR software package (version 5.06). Experimental NMR spectra were imported into the gNMR package using the gCVT and gSPG applications. Initial values for the chemical shift, linewidth, and coupling constant parameters for the fitting procedure were taken from the experimental data and modified manually until the calculated spectrum closely resembled the experimental spectrum. Simulated spectra were refined using the iterate function in gNMR. Some $^{2}J_{PP}$ coupling constants were set to 0 Hz and excluded from the iteration process as their inclusion in the fitting process did not make a clear change to the observed calculated band shapes.

2. Synthesis and Characterization of Catalysts

2.1. Synthesis $[Na(18\text{-c-}6)]_2[BBN\text{-}P_7]$, $[Na(18\text{-c-}6)]_2[1]$

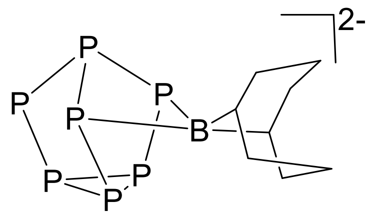

2 $[Na(18\text{-c-}6)]^+$

To a Schlenk flask charged with a stir bar and $[Na(18\text{-c-}6)]_2[HP_7]$ (1.005 g, 1.27 mmol, 1.0 eq.), HBBN dimer (464 mg, 1.90 mmol, 1.5 eq.), THF (40 mL) was added. Gas evolution was observed. The reaction mixture was allowed to stir until no gas evolution was observed, usually around 1h, and a dark orange solution was observed. The mixture was filtered yielding a clear dark orange solution. The solvent was removed under reduced pressure and the residue was washed with toluene (5 x 20mL). The residue was dissolved in THF (5 mL) and filtered yielding a clear dark orange solution. The solution was filtered and removal of volatiles under reduced pressure yielded glassy orange solids. Crystals suitable for X-ray diffraction analysis were obtained through slow diffusion of hexane into a concentrated THF solution.

Isolated Yield: 0.817 g, 70% yield.

^{1}H NMR (400 MHz, 298 K, Pyr-d_5/THF): δ = 3.44 (s, 48 H, *18-crown-6*), 2.30 – 1.70 (m, 14 H, *BBN*) ppm.

^{11}B NMR (128 MHz, 298 K, Pyr-d_5/THF): δ = 11.14 (s) ppm.

^{13}C{^{1}H} NMR (101 MHz, 298 K, Pyr-d_5/THF): δ = 69.56 (s, *18-crown-6*), 34.67 (t, $^2J_{CP}$ = 8.3 Hz, $P_2B\mathit{C}HCH_2CH_2$), 32.60 (t, $^3J_{CP}$ = 4.4 Hz, $P_2BCH\mathit{C}H_2CH_2$), 15.14 (s, $P_2BCHCH_2\mathit{C}H_2$) ppm.

^{31}P NMR (162 MHz, 298 K, Pyr-d_5/THF): δ = 76.70 (ddd, $^1J_{PP}$ = 345.9, 212.1, $^2J_{PP}$ = 61.1 Hz, 2P, basal), 45.88 (dtd, $^1J_{PP}$ = 377.3, 233.1, $^2J_{PP}$ = 55.3 Hz, 1P, apical), -31.88 (dd (pseudo triplet), $^1J_{PP}$ = 369.3 Hz, 1P, bridging), –56.44 – –63.25 (m, 1P, basal), –153.26 – –159.94 (m, 2P, bridging) ppm.

Elemental analysis for $C_{32}H_{62}BNa_2O_{12}P_7$: calcd.: C 42.12, H 6.85, N 0.0; found C 42.17, H 7.12, N 0.0

Note: The cluster was not observed by mass spectrometry, possibly due to its high air and mositure sensitivity resulting in cluster decomposition.

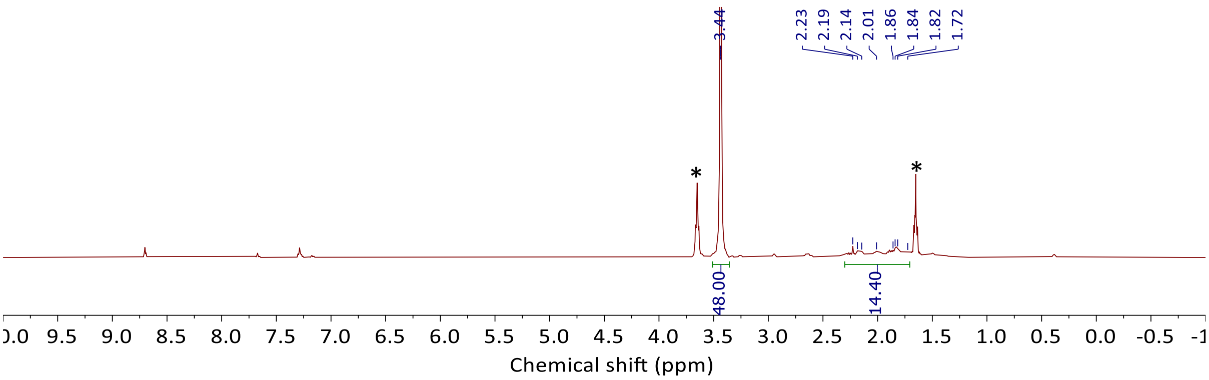

Figure S1. ^{1}H NMR spectrum (Pyr-d_5/THF) of $[Na(18\text{-}c\text{-}6)]_2[\mathbf{1}]$. THF marked with *.

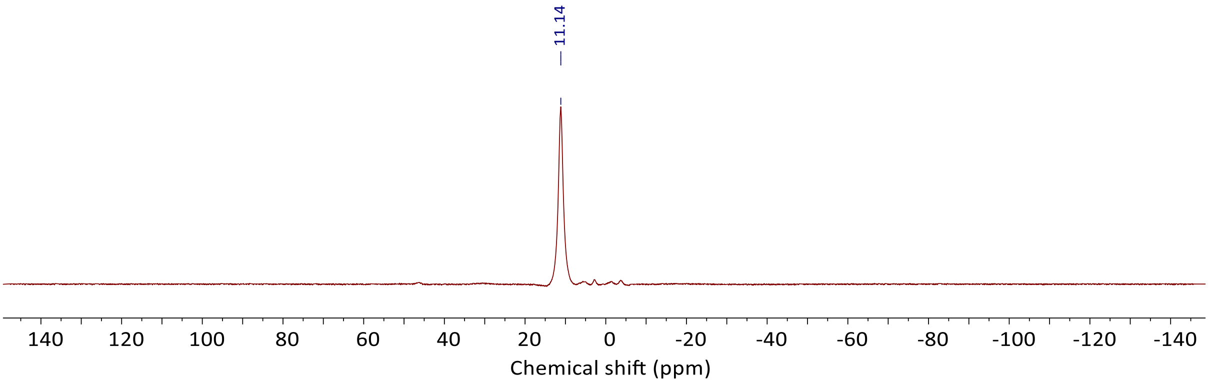

Figure S2. ^{11}B NMR spectrum (Pyr-d_5/THF) of $[Na(18\text{-}c\text{-}6)]_2[\mathbf{1}]$.

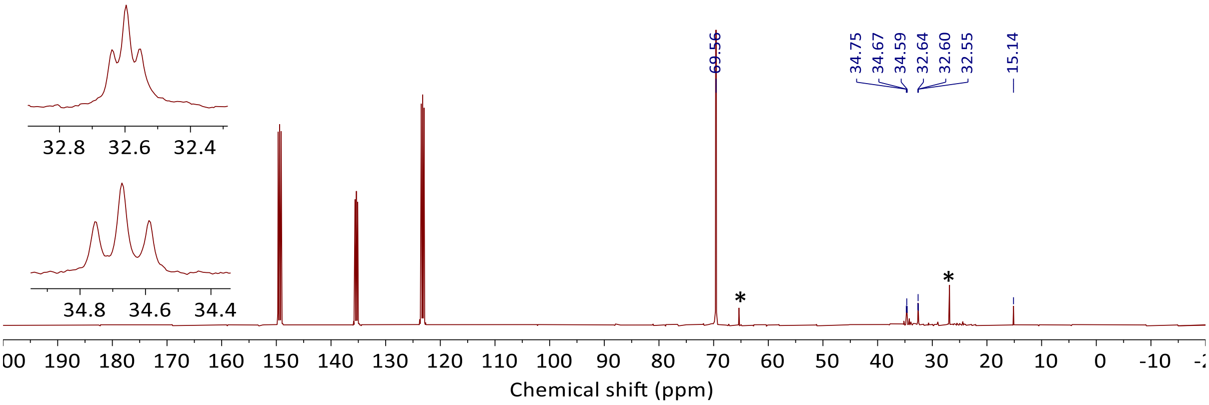

Figure S3. $^{13}C\{^{1}H\}$ NMR spectrum (Pyr-d_5/THF) of $[Na(18\text{-}c\text{-}6)]_2[\mathbf{1}]$. THF marked with *.

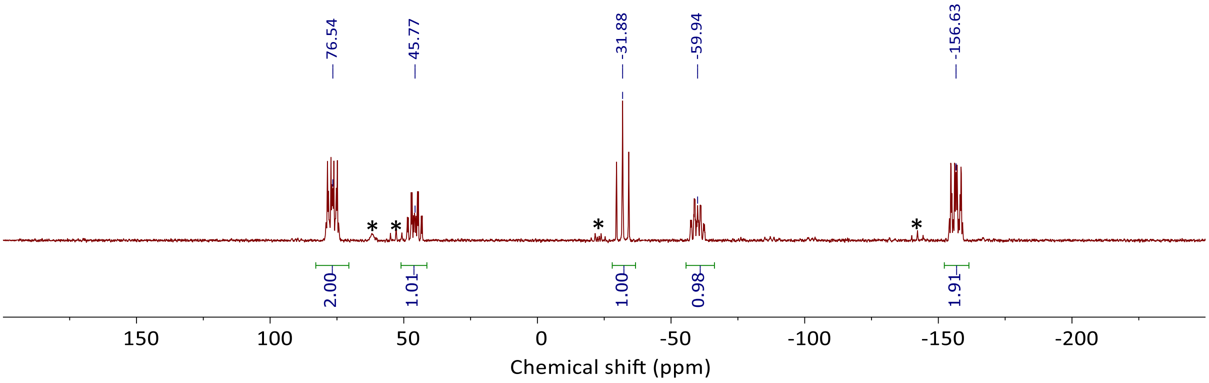

Figure S4. ^{31}P NMR spectrum (Pyr-d_5/THF) of $[Na(18\text{-}c\text{-}6)]_2[\mathbf{1}]$. Resonances marked with * are possibly from a small amount of $[\mathbf{1'}]^{2-}$ (<2%).

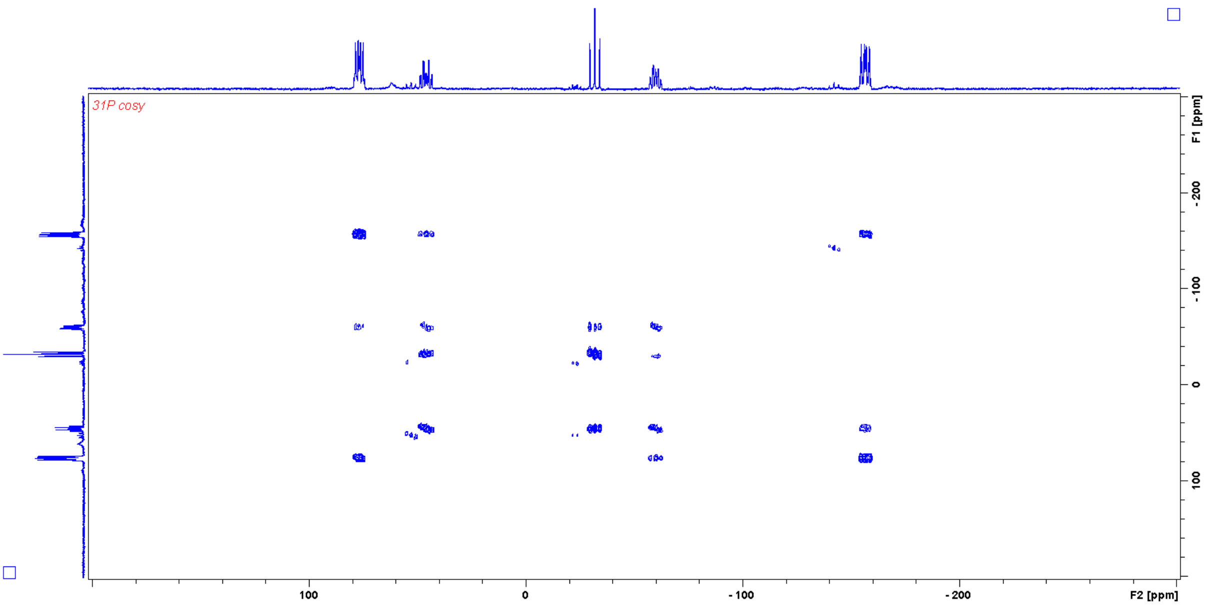

Figure S5. ^{31}P COSY NMR spectrum (Pyr-d_5/THF) of [Na(18-c-6)]$_2$[**1**].

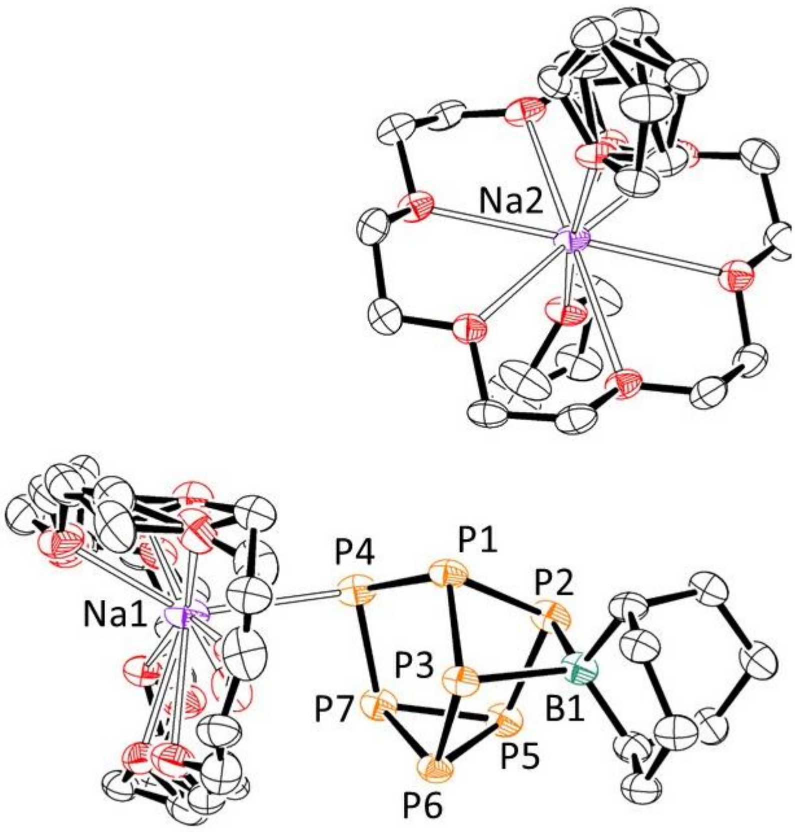

Figure S6. Molecular structure of [Na(18-c-6)]$_2$[**1**]. Anisotropic displacement ellipsoids pictured at 50% probability. Hydrogen atoms omitted for clarity. Phosphorus: Orange; Boron: Green; Carbon: White; Sodium: Purple.

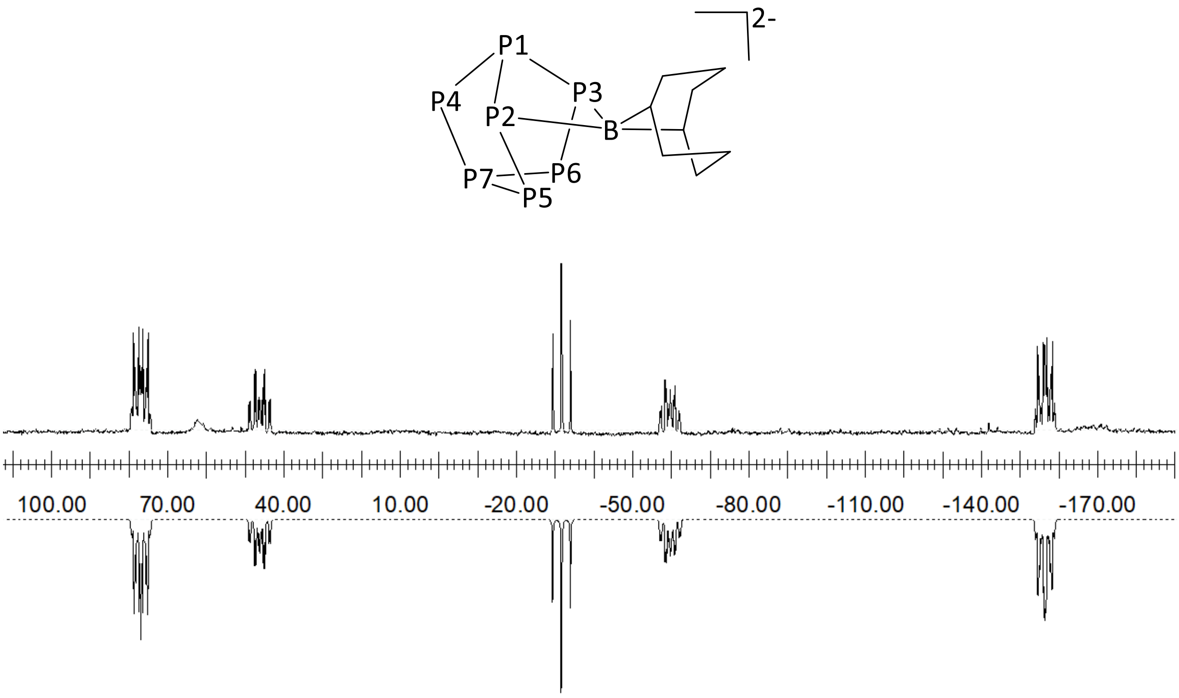

Figure S7. ^{31}P NMR (*o*DFB) spectrum of [Na(18-c-6)]$_2$[**1**].Top: Experimental NMR spectra; bottom: simulated NMR spectrum.

Table S1. Simulated NMR spectroscopic values

Nucleus	Chemical shift (ppm)	*J* coupling	coupling constant (Hz)
P1	–59.70	^1JP1–P2	212
P2, P3	76.96	^1JP1–P3	212
P4	–31.67	^1JP1–P4	370
P5, P6	–156.46	^2JP1–P5	28
P7	46.25	^2JP1–P6	28
		^2JP1–P7	61
		^1JP2–P5	346
		^1JP3–P6	346
		^1JP4–P7	370
		^1JP5–P6	200
		^1JP5–P7	233
		^1JP6–P7	233
		Final residual	1.96e+05

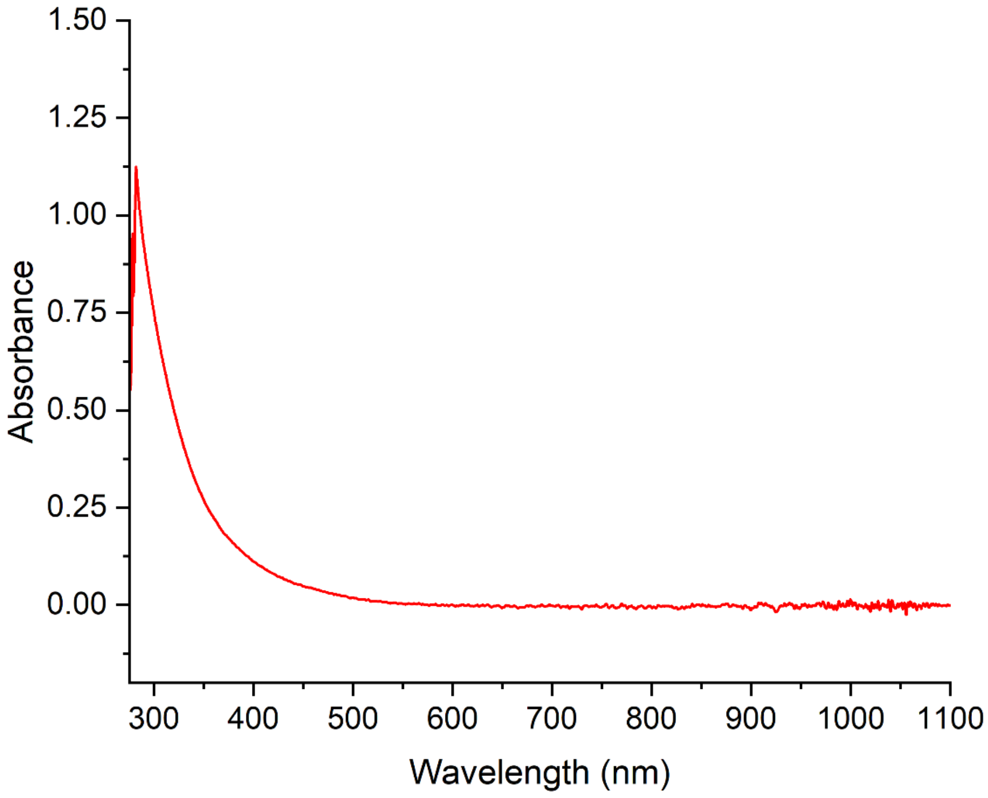

Figure S8. Uv-Vis spectrum of [**1**]$^{2–}$ (0.11 mM in *o*DFB).

2.2. Synthesis [K(18-c-6)]$_2$[BBN-P$_7$], [K(18-c-6)]$_2$[1]

Compound [K(18-c-6)]$_2$[**1**] was prepared anologuesly to compound [Na(18-c-6)]$_2$[**1**] by using [K(18-c-6)]$_2$[HP$_7$] instead. [K(18-c-6)]$_2$[**1**] was obtained in similar yields to [Na(18-c-6)]$_2$[**1**]. NMR spectroscopic data are idenitcal to [Na(18-c-6)]$_2$[**1**]. Crystals suitable for X-ray diffraction analysis were obtained through slow diffusion of toluene into a concentrated THF solution.

Elemental analysis for $C_{32}H_{62}BK_2O_{12}P_7$: calcd.: C 40.69, H 6.62, N 0.0; found C 40.20, H 6.87, N 0.0

Note: The cluster was not observed by mass spectrometry, possibly due to its high air and moisture sensitivity resulting in cluster decomposition.

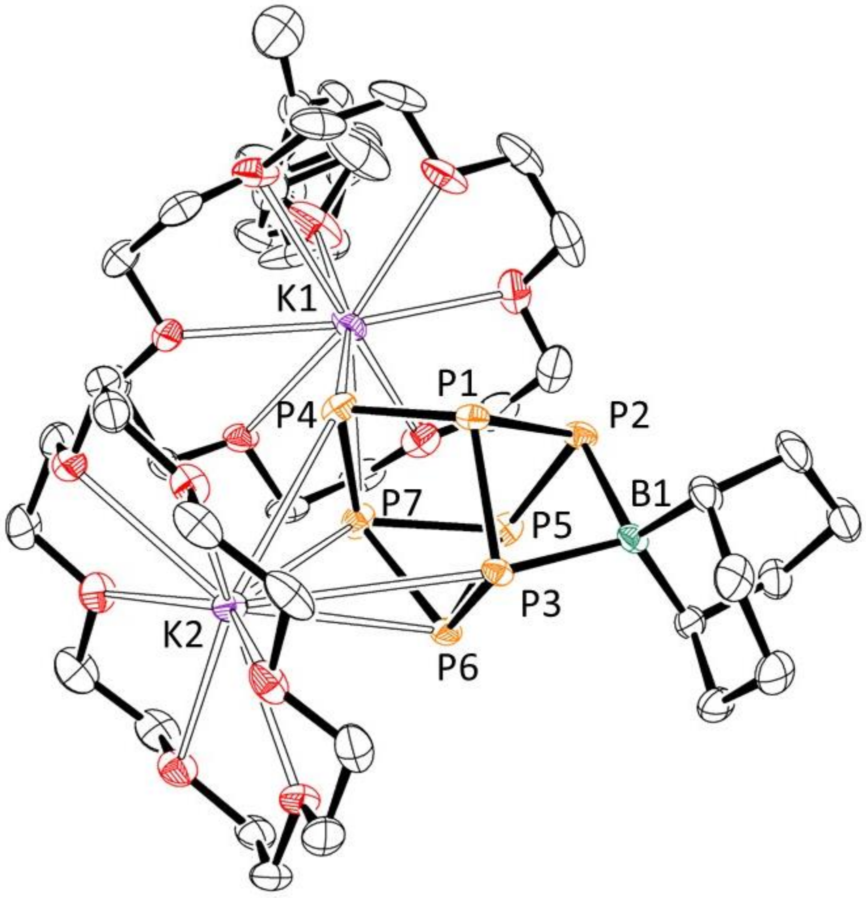

Figure S9. Molecular structure of [K(18-c-6)]$_2$[**1**]. Anisotropic displacement ellipsoids pictured at 50% probability. Hydrogen atoms and toluene solvent molecule ommited for clarity. Phosphorus: Orange; Boron: Green; Carbon: White; Potassium: Purple.

3. Catalytic Hydroboration of Ketones/Aldehydes

3.1. Reaction Conditions Screening

Aldehyde/ ketone (0.22 mmol, 1.0 eq.) and toluene (25 µL, 0.24 mmol) was added to a solution of catalyst (2.2 – 11.0 µmol, 0.01 – 0.05 eq.), reductant (0.22 mmol, 1.0 eq.) and solvent (0.5 mL) in a J Young NMR tube. The reaction was allowed to react for 3 h. The reaction was monitored by ^{1}H NMR, ^{11}B NMR and $^{11}B\{^{1}H\}$ NMR. Crude NMR conv. was determined by integration of the ^{1}H NMR spectrum using the toluene as internal standard (^{1}H δ = 2.31 ppm).

Table S2. Optimization Aldehyde and Ketone Hydroboration

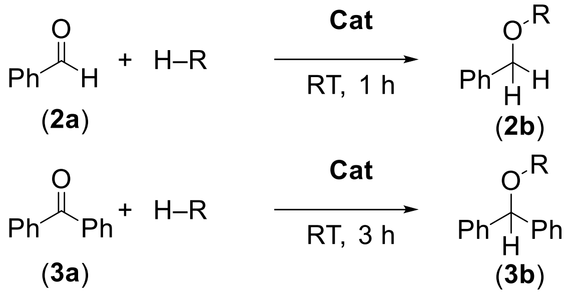

Catalysts (mol%)	Temp. (°C)	Reductant (H–R)	Solvent	**2b** conv. (%)[a]	**3b** conv. (%)[a]
-	RT	HBpin	THF	0	0
K_3P_7 (5)	RT	HBpin	THF	0	0
K_3P_7 + 18-c-6 (5)	RT	HBpin	THF	0	0
$[Na(18\text{-}c\text{-}6)]_2$[**1**] (5)	RT	HBpin	THF	92	80
$[Na(18\text{-}c\text{-}6)]_2$[**1**] (1)	RT	HBpin	THF	76	48
$[Na(18\text{-}c\text{-}6)]_2$[**1**] (1)	RT	HBpin	*o*DFB	>99	>99
$[Na(18\text{-}c\text{-}6)]_2$[**1**] (1)	50	Et_3SiH	*o*DFB	0	0
$[Na(18\text{-}c\text{-}6)]_2$[**1**] (1)	50	Ph_3SiH	*o*DFB	0	0
$[K(18\text{-}c\text{-}6)]_2$[**1**] (1)	RT	HBpin	THF	75	52
$[K(18\text{-}c\text{-}6)]_2$[**1**] (1)	RT	HBpin	*o*DFB	>99	>99
$[Na(18\text{-}c\text{-}6)]_2[HP_7]$ (5)[b]	RT	HBpin	*o*DFB	69	60

[a] Determined by ^{1}H NMR spectroscopy using toluene (0.235 mmol) as an internal standard. [b] Pre-catalyst.

3.2. General Procedure for Hydroboration of Ketones/Aldehydes

Ketone/ aldehyde (0.22 mmol, 1.0 eq.) and toluene (25 µL, 0.24 mmol) was added to a solution of $[Na(18\text{-}c\text{-}6)]_2$[**1**] (2 mg, 2.2 µmol, 0.01 eq.), pinacolborane (28 mg, 32 µL, 0.22 mmol, 1.0 eq.) and *o*DFB (0.5 mL) in a J Young NMR tube. The reaction was allowed to react for 0.5 – 72 h. The reaction was monitored by ^{1}H NMR, ^{11}B NMR and $^{11}B\{^{1}H\}$ NMR. NMR conv. was determined by integration of the crude ^{1}H NMR spectrum using the toluene as an internal standard (^{1}H δ = 2.31 ppm). The solvent was removed under reduced pressure and the residue was extracted with pentane or toluene. Removal of volatiles or crystallization allowed for the isolation of the hydroboration products.

Table S3. Catalytic hydroboration of aldehydes compounds.

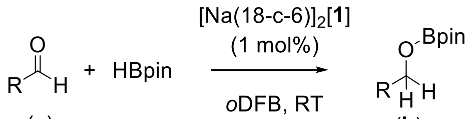

(a) (b)

Entry	Aldehyde	Product	Time (h)	Conv. (%)[a]	TOF (h^{-1})
1	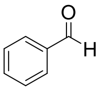 (2a)	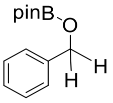 (2b)	0.5	>99 (94)	200
2	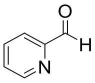 (4a)	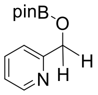 (4b)	0.5	>99 (85)	200
2	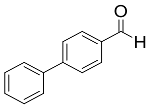 (5a)	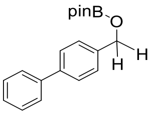 (5b)	8	>99 (91)	12.5
3	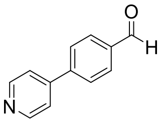 (6a)	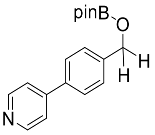 (6b)	5	>99 (91)	20
5	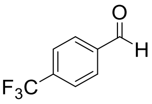 (7a)	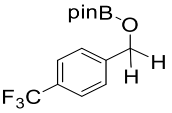 (7b)	10	>99 (89)	10
6	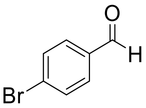 (8a)	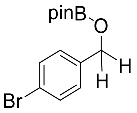 (8b)	10	>99 (93)	10
7	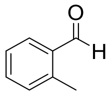 (9a)	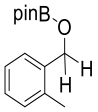 (9b)	5	>99 (92)	20
8	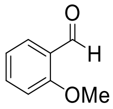 (10a)	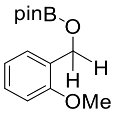 (10b)	10	>99 (90)	10
9	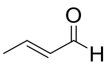 (11a)	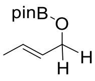 (11b)	10	80 (67)	8
10	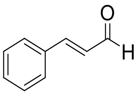 (12a)	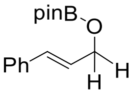 (12b)	10	>99 (91)	10
11[b]	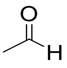 (13a)	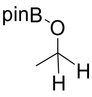 (13b)	0.5	76	152

[a] Determined by ^{1}H NMR spectroscopy using toluene (0.235 mmol) as an internal standard. Yields of the isolated product are given within parentheses. [b] 5.0 eq. of substrate was used.

Table S4. Catalytic Hydroboration of Ketones.

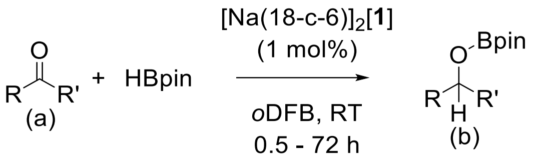

Entry	Ketone	Product	Time (h)	Conv. (%)[a]	TOF (h^{-1})
1	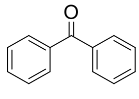 (**3a**)	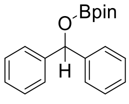 (**3b**)	3	>99 (96)	33
2	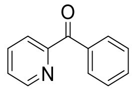 (**14a**)	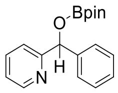 (**14b**)	18	>99 (95)	5.5
3	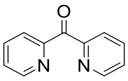 (**15a**)	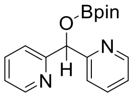 (**15b**)	0.5	>99 (94)	200
4	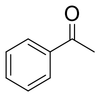 (**16a**)	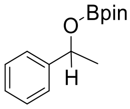 (**16b**)	0.5	>99 (96)	200
5	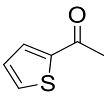 (**17a**)	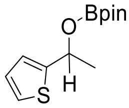 (**17b**)	0.5	>99 (90)	200
6	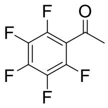 (**18a**)	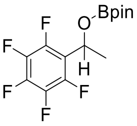 (**18b**)	72	80 (69)	1.1
7	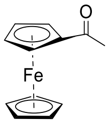 (**19a**)	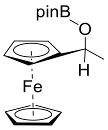 (**19b**)	72	85 (79)	1.2
8	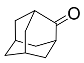 (**20a**)	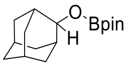 (**20b**)	18	>99 (92)	5.5
9	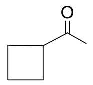 (**21a**)	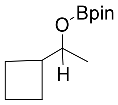 (**21b**)	2	>99 (91)	50
10	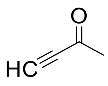 (**22a**)	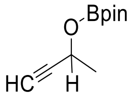 (**22b**)	72	23	0.3
11	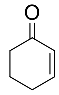 (**23a**)	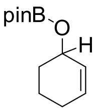 (**23b**)	72	33	0.5

[a] Determined by ^{1}H NMR spectroscopy using toluene (0.235 mmol) as an internal standard. Yields of the isolated product are given within parentheses.

3.2.1. Characterization Data Hydroboration of Aldehydes

3.2.1.1. 2-(benzyloxy)-4,4,5,5-tetramethyl-1,3,2-dioxaborolane

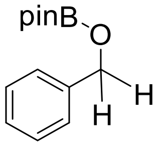

^{1}H NMR (400 MHz, 298 K, C_6D_6): δ = 7.30 (d, $^3J_{HH}$ = 7.5 Hz, 2H, *Ar*), 7.12 (dd, $^3J_{HH}$ = 7.6, 7.6 Hz, 2H, *Ar*), 7.04 (t, $^3J_{HH}$ = 7.3 Hz, 1H, *Ar*), 4.95 (s, 2H, O–C*H*$_2$), 1.03 (s, 12H) ppm.

^{11}B NMR (128 MHz, 298 K, C_6D_6): δ = 22.8 (s) ppm.

^{13}C{^{1}H} NMR (101 MHz, 298 K, C_6D_6): δ = 139.69 (s, *Ar*), 128.22 (s, *Ar*), 127.20 (s, *Ar*), 126.67 (s, *Ar*), 82.37 (s, *OBpin*), 66.58 (s, O–CH$_2$), 24.32 (s, *OBpin*) ppm.

Mass spectrometry (APCI): $C_{13}H_{20}B_1O_3$+Na ([M+Na]$^+$): calcd: 257.1325; found: 257.1325.

NMR conv.: >99%

Isolated Yield: 94%

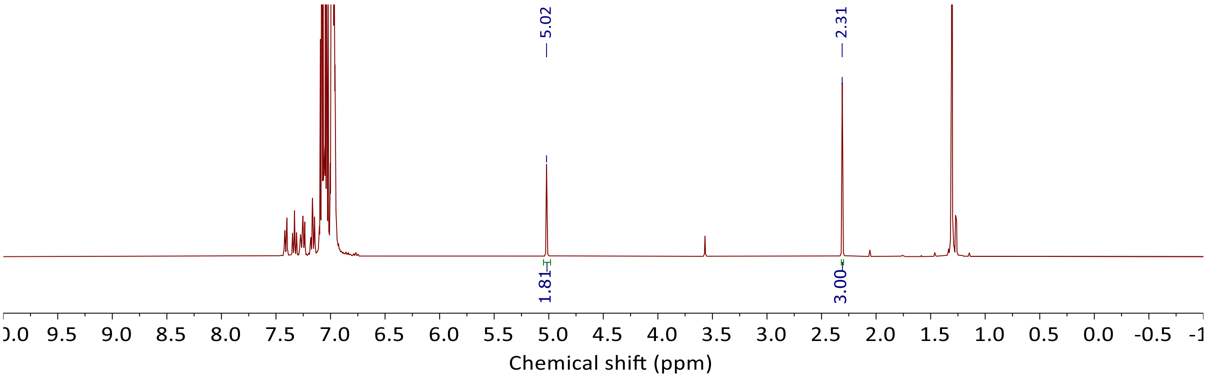

Figure S10. ^{1}H NMR spectrum (*o*DFB) of crude **2b**.

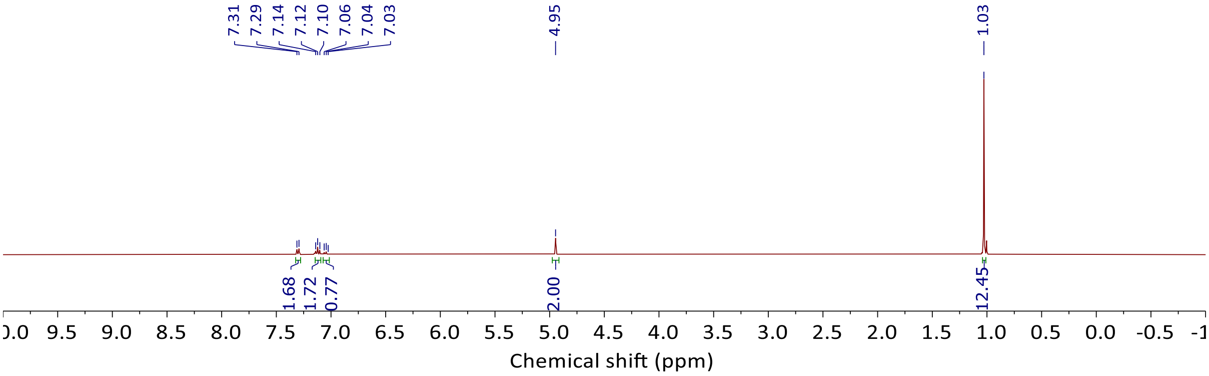

Figure S11. ^{1}H NMR spectrum (C_6D_6) of isolated **2b**.

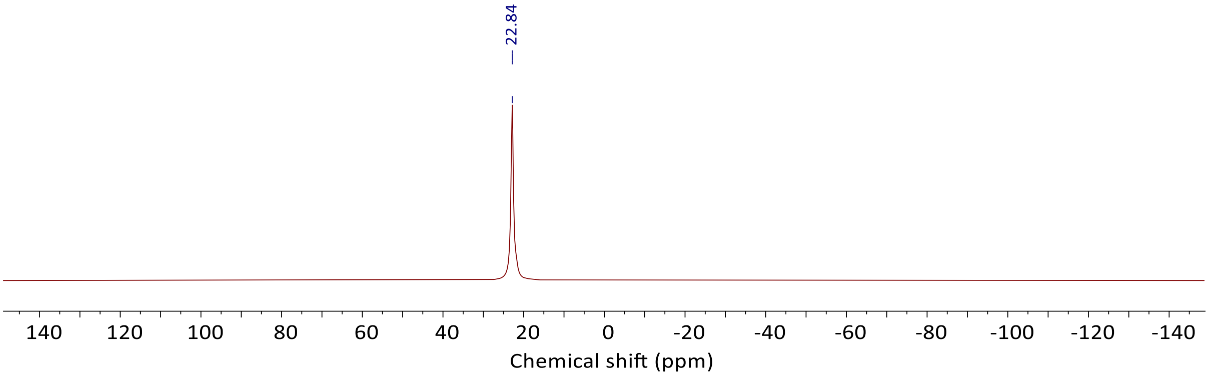

Figure S12. ^{11}B NMR spectrum (C_6D_6) of isolated **2b**.

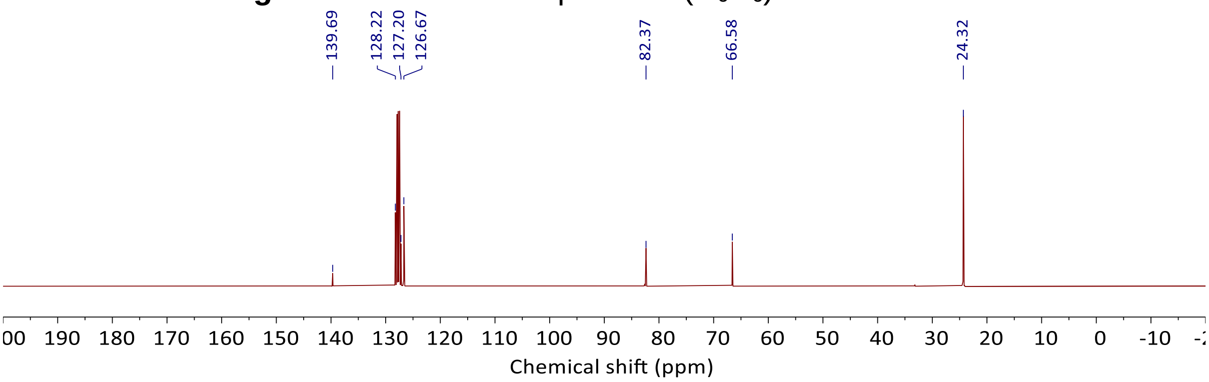

Figure S13. $^{13}C\{^1H\}$ NMR spectrum (C_6D_6) of isolated **2b**.

3.2.1.2. 2-{[(4,4,5,5-tetramethyl-1,3,2-dioxaborolan-2-yl)oxy]methyl}pyridine

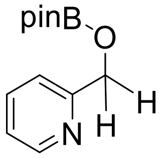

^1H NMR (400 MHz, 298 K, C_6D_6): δ = 8.39 (d, $^3J_{HH}$ = 5.8 Hz, 1H, *Ar*), 7.27 (d, $^3J_{HH}$ = 7.9 Hz, 1H, *Ar*), 7.06 (td, $^3J_{HH}$ = 7.7, $^4J_{HH}$ = 1.8 Hz, 1H, *Ar*), 6.58 (dd, $^3J_{HH}$ = 6.9, 5.5 Hz, 1H, *Ar*), 5.22 (s, 2H, O–C*H*$_2$), 1.06 (s, 12H, *OBpin*) ppm.

^{11}B NMR (128 MHz, 298 K, C_6D_6): δ = 22.3 (s) ppm.

$^{13}C\{^1H\}$ NMR (101 MHz, 298 K, C_6D_6): δ = 159.62 (s, *Ar*), 148.37 (s, *Ar*), 136.05 (s, *Ar*), 121.66 (s, *Ar*), 119.54 (s, *Ar*), 82.32 (s, *OBpin*), 67.54 (s, O–CH_2), 24.42 (s, *OBpin*) ppm.

Mass spectrometry (APCI): $(C_{12}H_{18}B_1O_3)_2$+Na ($[Mx2+Na]^+$): calcd: 493.2657; found: 493.2584.

NMR conv.: >99%

Isolated Yield: 85%

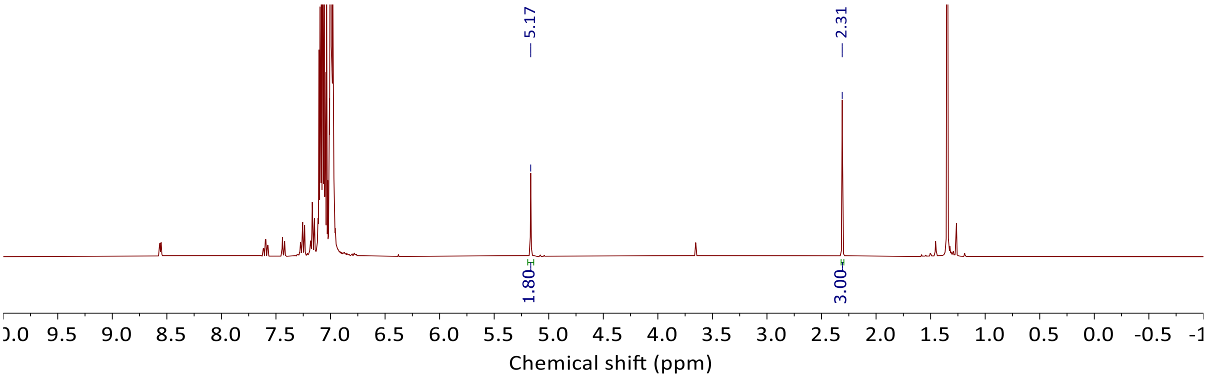

Figure S14. ^{1}H NMR spectrum (*o*DFB) of crude **4b**.

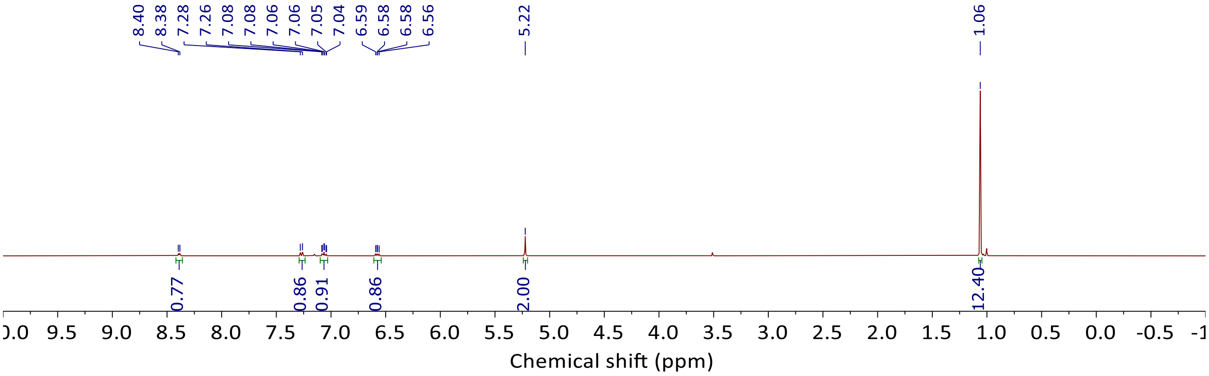

Figure S15. ^{1}H NMR spectrum (C_6D_6) of isolated **4b**.

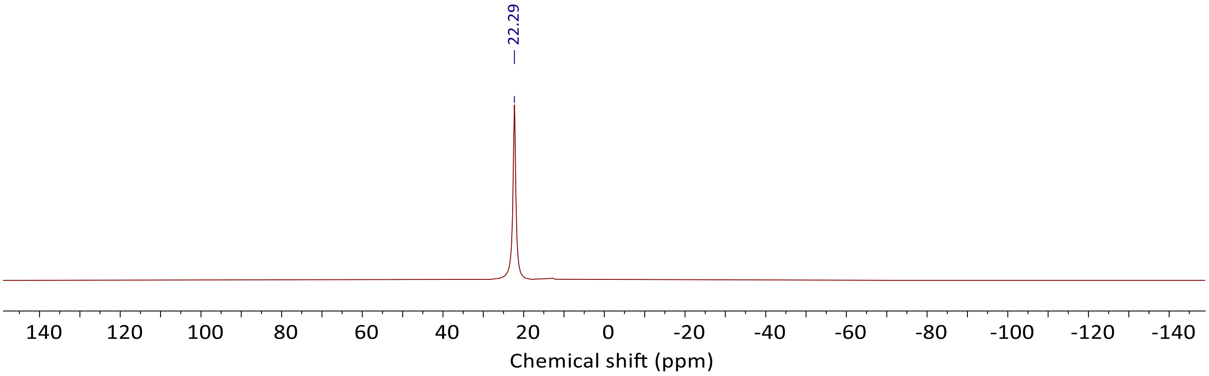

Figure S16. ^{11}B NMR spectrum (C_6D_6) of isolated **4b**.

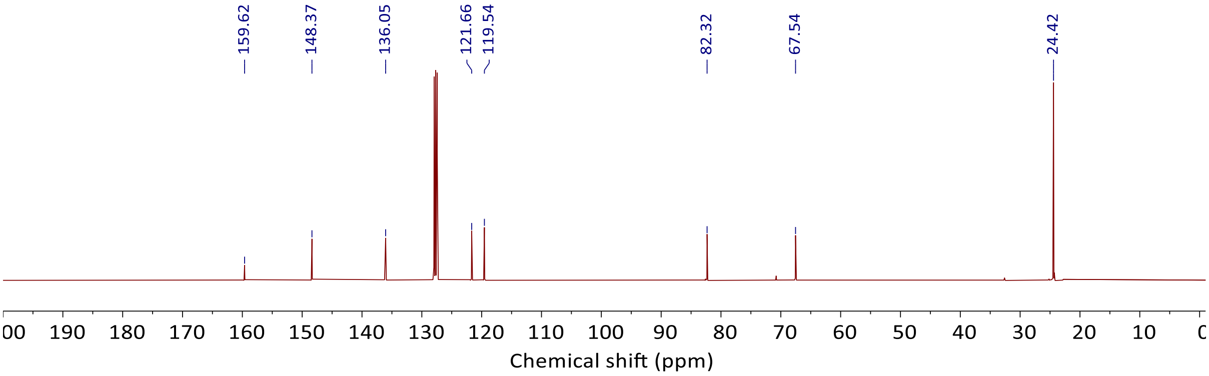

Figure S17. $^{13}C\{^{1}H\}$ NMR spectrum (C_6D_6) of isolated **4b**.

3.2.1.3. 2-(4-phenylbenzyloxy)-4,4,5,5-tetramethyl-1,3,2-dioxaborolane

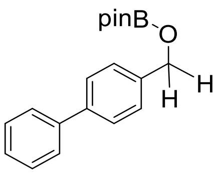

^{1}H NMR (400 MHz, 298 K, C_6D_6): δ = 7.44 – 7.40 (m, 4H, *Ar*), 7.37 – 7.33 (m, 2H, *Ar*), 7.20 (t, $^{3}J_{HH}$ = 7.4 Hz, 2H, *Ar*), 7.16 – 7.07 (m, 1H), 5.00 (s, 2H, O–C*H₂*), 1.06 (s, 12H, *OBpin*).

^{11}B NMR (128 MHz, 298 K, C_6D_6): δ = 22.9 (s) ppm.

^{13}C{^{1}H} NMR (101 MHz, 298 K, C_6D_6): δ = 141.45 (s, *Ar*), 140.74 (s, *Ar*), 139.07 (s, *Ar*), 129.02 (s, *Ar*), 127.59 (s, *Ar*), 127.45 (s, *Ar*), 127.40 (overlapping singlets, *Ar*), 82.81 (s, *OBpin*), 66.76 (s, O–C*H₂)*, 24.73 (s, *OBpin*) ppm.

Mass spectrometry (APCI): $C_{19}H_{23}B_1O_3$-H ($[M–H]^-$): calcd: 309.1656; found: 308.1660.

NMR conv.: >99%

Isolated Yield: 91%

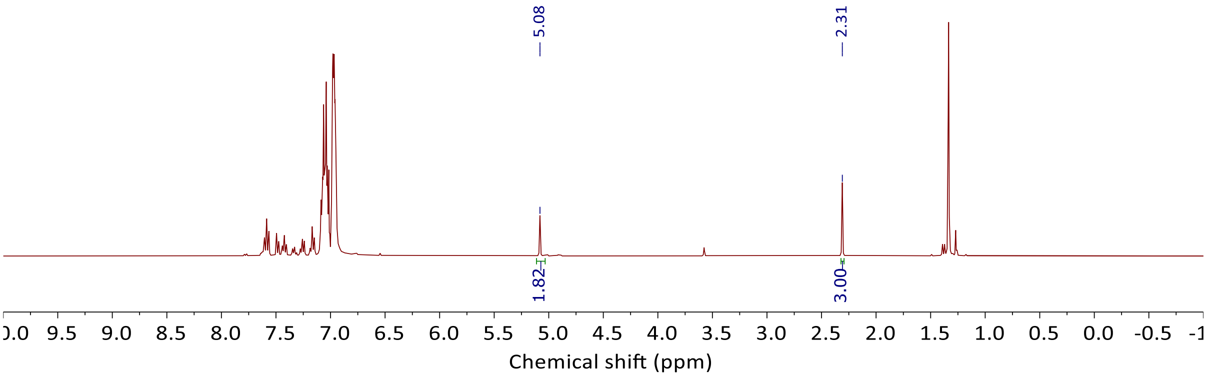

Figure S18. ^{1}H NMR spectrum (*o*DFB) of crude **5b**.

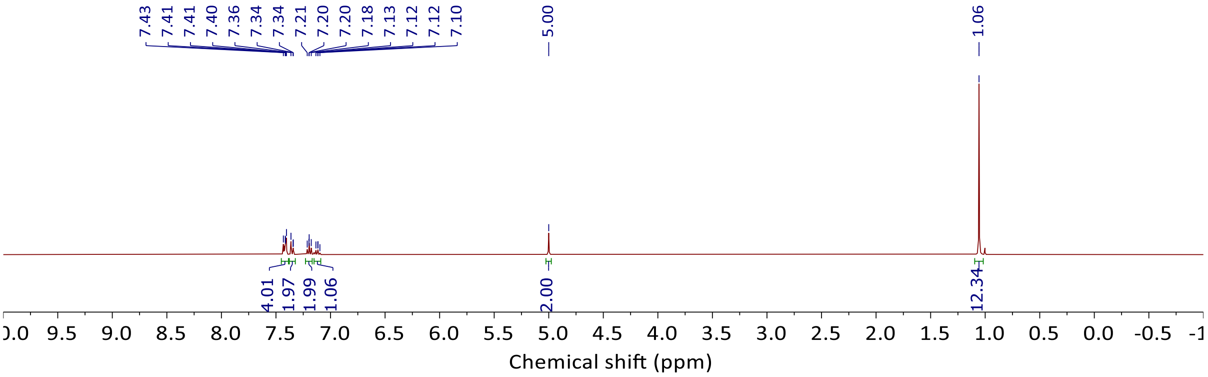

Figure S19. ^{1}H NMR spectrum (C_6D_6) of isolated **5b**.

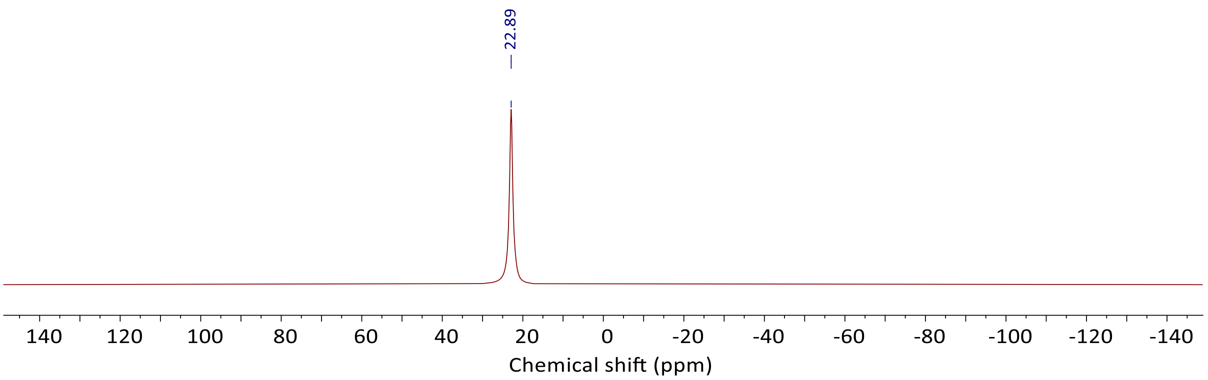

Figure S20. ^{11}B NMR spectrum (C_6D_6) of isolated **5b**.

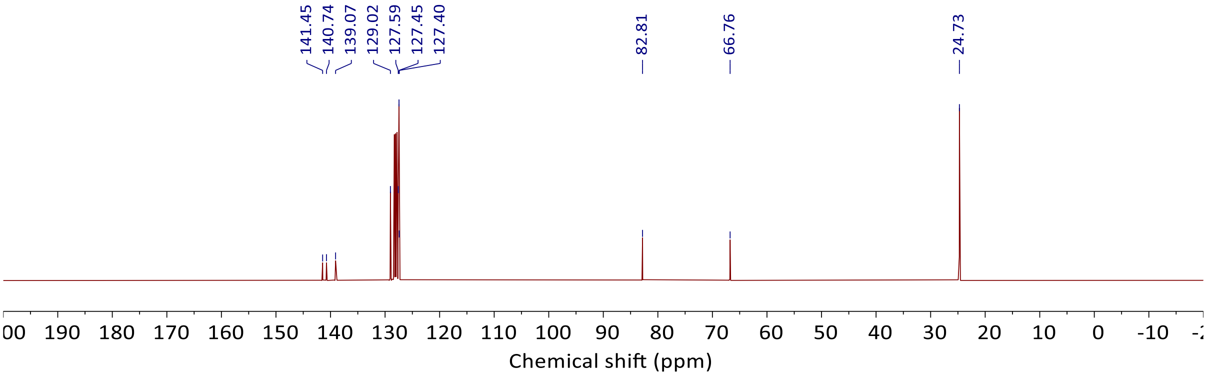

Figure S21. $^{13}C\{^1H\}$ NMR spectrum (C_6D_6) of isolated **5b**.

3.2.1.4. 4-{4-[((4,4,5,5-tetramethyl-1,3,2-dioxaborolan-2-yl)oxy)methyl]phenyl}pyridine

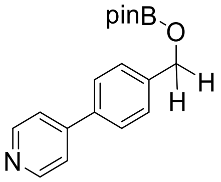

^1H NMR (400 MHz, 298 K, C_6D_6): δ = 8.61 – 8.56 (m, 2H, *Ar*), 7.33 – 7.23 (m, 4H, *Ar*), 7.05 – 6.99 (m, 2H, *Ar*), 4.97 (s, 2H, O–C*H*$_2$), 1.06 (s, 12H, *OBpin*) ppm.

^{11}B NMR (128 MHz, 298 K, C_6D_6): δ = 22.9 (s) ppm.

$^{13}C\{^1H\}$ NMR (101 MHz, 298 K, C_6D_6): δ = 150.44 (s, *Ar*), 147.28 (s, *Ar*), 140.45 (s, *Ar*), 137.22 (s, *Ar*), 127.15 (s, *Ar*), 126.87 (s, *Ar*), 121.15 (s, *Ar*), 82.56 (s, *OBpin*), 66.15 (s, O–CH_2), 24.36 (s, *OBpin*) ppm.

Mass spectrometry after hydrolysis (ESI): $C_{12}H_{11}N_1O_1$+H ($[M+H]^-$): calcd: 186.0913; found186.0908.

NMR conv.: >99%

Isolated Yield: 91%

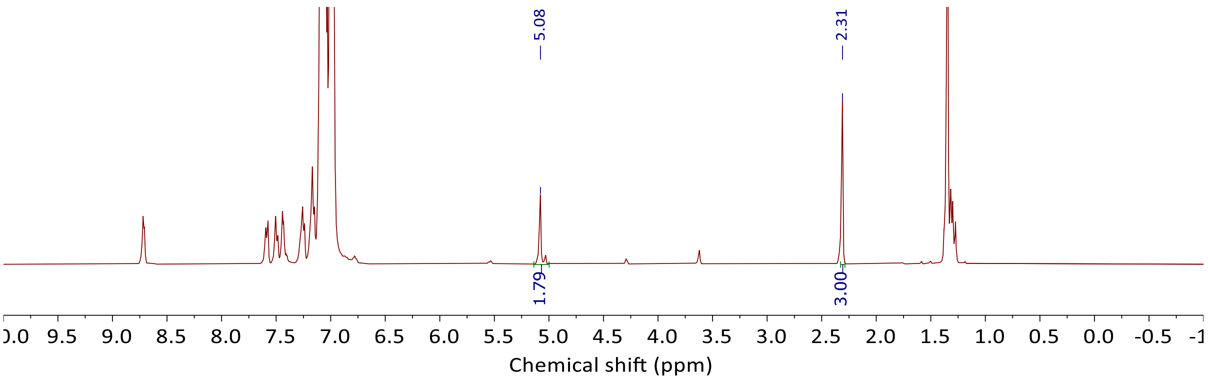

Figure S22. ^{1}H NMR spectrum (*o*DFB) of crude **6b**.

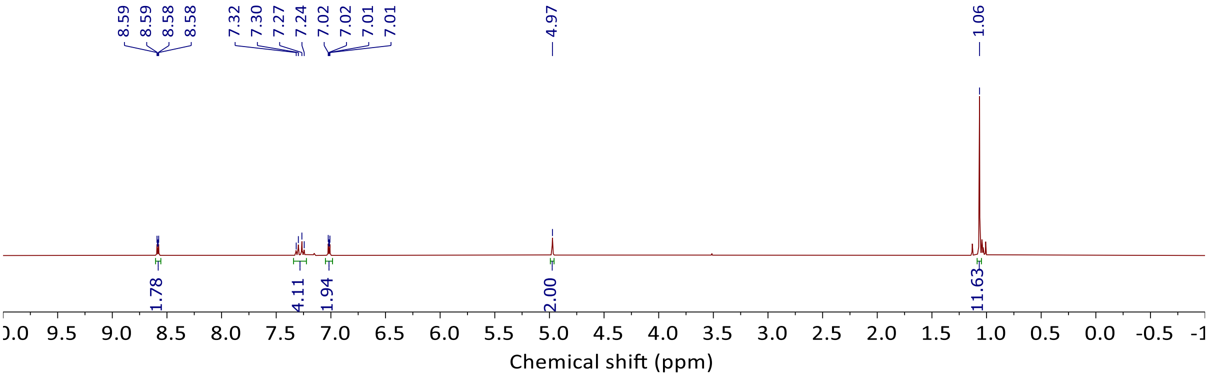

Figure S23. ^{1}H NMR spectrum (C_6D_6) of isolated **6b**.

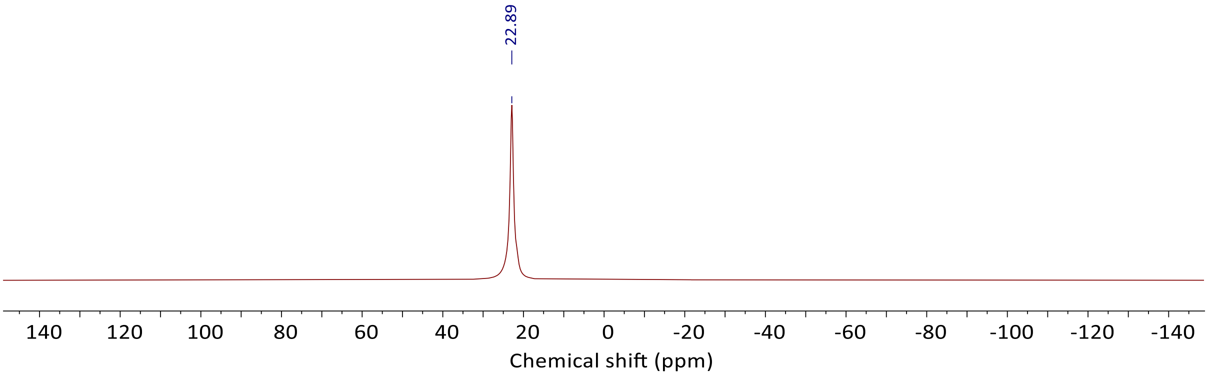

Figure S24. ^{11}B NMR spectrum (C_6D_6) of isolated **6b**.

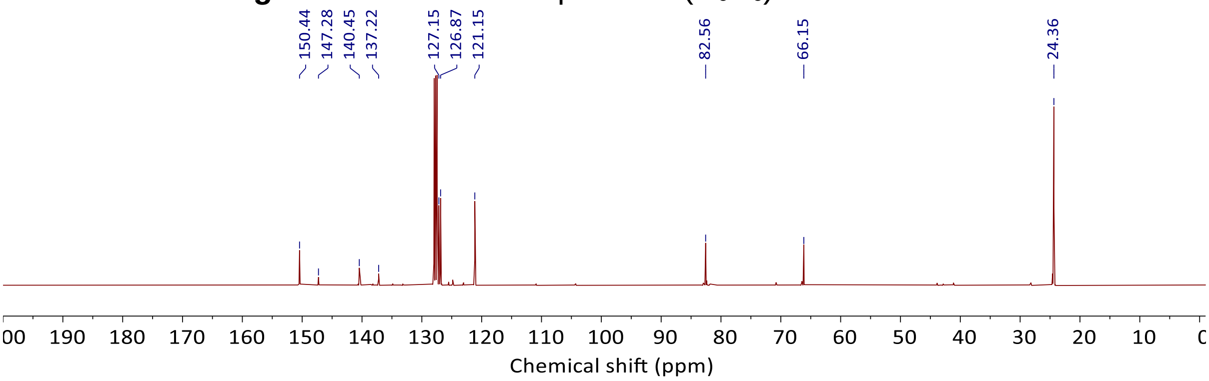

Figure S25. ^{13}C{^{1}H} NMR spectrum (C_6D_6) of isolated **6b**.

3.2.1.5. 2-(4-trifluoromethylbenzyloxy)-4,4,5,5-tetramethyl-1,3,2-dioxaborolane

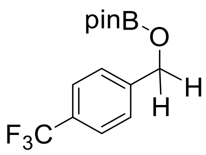

^{1}H NMR (400 MHz, 298 K, C_6D_6): δ = 7.29 (d, $^{3}J_{HH}$ = 8.8 Hz, 2H, *Ar*), 7.08 (d, $^{3}J_{HH}$ = 8.3 Hz, 2H, *Ar*), 4.78 (s, 2H, O–C*H*$_2$), 1.03 (s, 12H, *OBpin*) ppm.

^{11}B NMR (128 MHz, 298 K, C_6D_6): δ = 22.8 (s) ppm.

^{13}C{^{1}H} NMR (101 MHz, 298 K, C_6D_6): δ = 143.51 (s, *Ar*), 129.22 (q, $^{1}J_{CF}$ = 32.0 Hz, Ar–*C*F$_3$), 126.48 (s, *Ar*), 125.98 (s, *Ar*), 125.07 (q, $^{2}J_{CF}$ = 3.9 Hz, *Ar*–CF$_3$), 82.62 (s, *OBpin*), 65.63 (s, O–*C*H$_2$*)*, 24.25 (s, *OBpin*) ppm.

^{19}F{^{1}H} NMR (376 MHz, 298 K, C_6D_6): δ = –62.2 (s) ppm.

Mass spectrometry (APCI): $C_{14}H_{18}B_1O_3F_3$+H ([M+H]$^{+}$): calcd: 303.1376; found: 303.1377.

NMR conv.: >99%

Isolated Yield: 89%

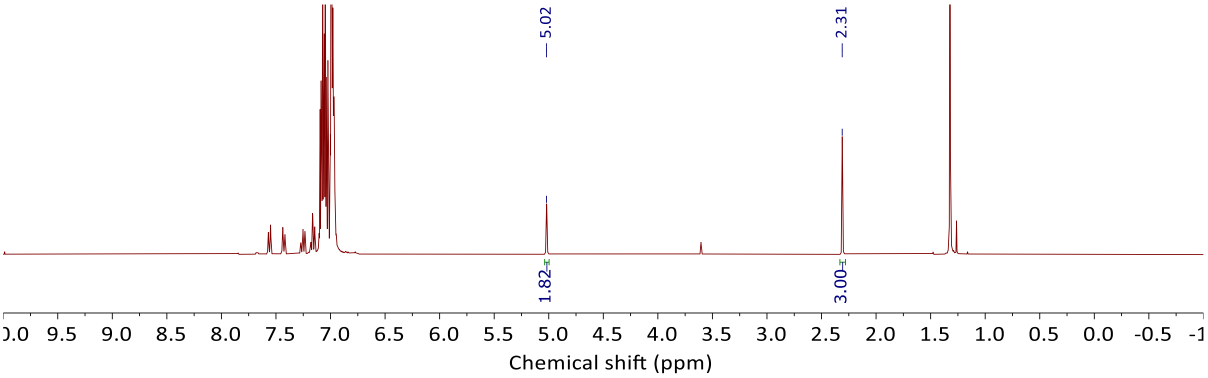

Figure S26. ^{1}H NMR spectrum (*o*DFB) of crude **7b**.

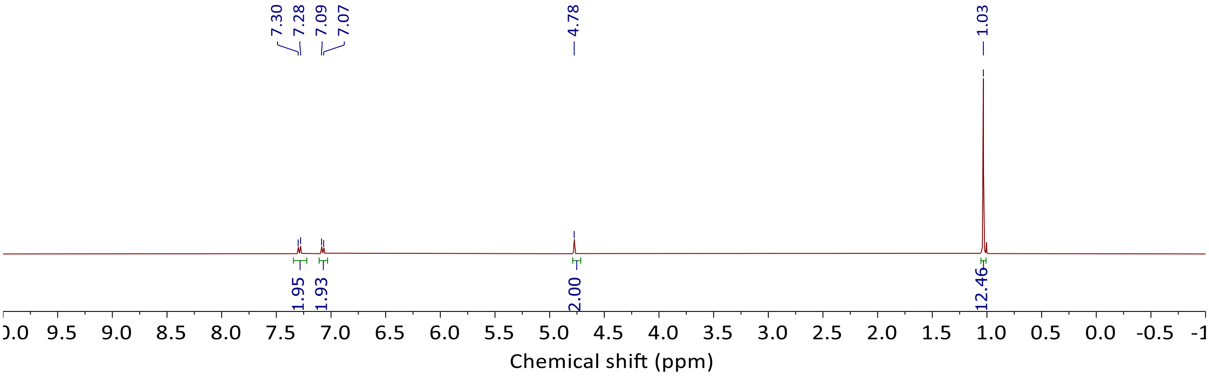

Figure S27. ^{1}H NMR spectrum (C_6D_6) of isolated **7b**.

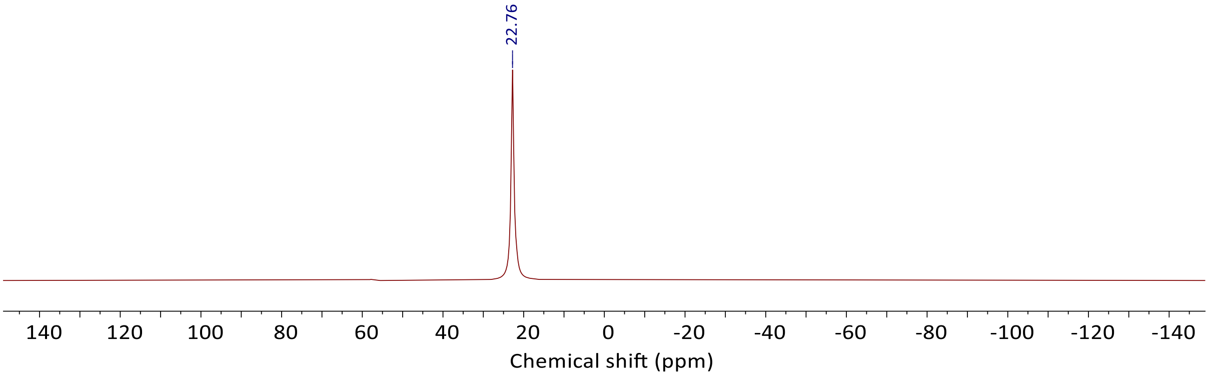

Figure S28. ^{11}B NMR spectrum (C_6D_6) of isolated **7b**.

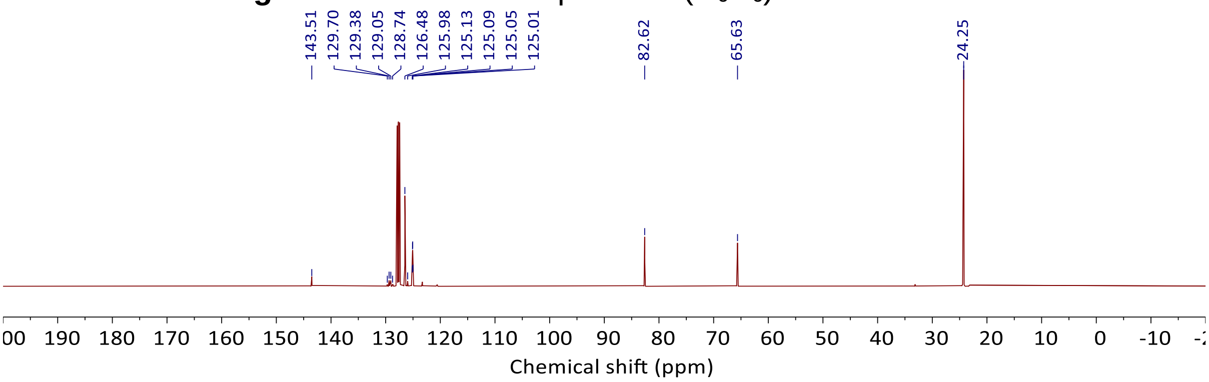

Figure S29. $^{13}C\{^{1}H\}$ NMR spectrum (C_6D_6) of isolated **7b**.

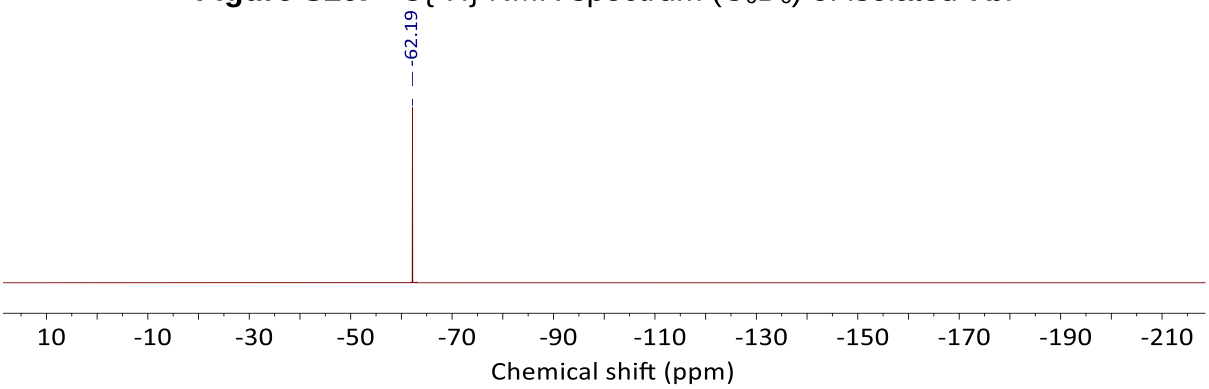

Figure S30. $^{19}F\{^{1}H\}$ NMR spectrum (C_6D_6) of isolated **7b**.

3.2.1.6. 2-((4-bromobenzyl)oxy)-4,4,5,5-tetramethyl-1,3,2-dioxaborolane

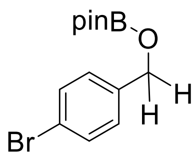

^{1}H NMR (400 MHz, 298 K, C_6D_6): δ = 7.20 (d, $^{3}J_{HH}$ = 8.4 Hz, 2H, *Ar*), 6.92 (d, $^{3}J_{HH}$ = 8.7 Hz, 2H, *Ar*), 4.72 (s, 2H, O–C*H*$_2$), 1.02 (s, 12H, *OBpin*) ppm.

^{11}B NMR (128 MHz, 298 K, C_6D_6): δ = 22.7 (s) ppm.

$^{13}C\{^{1}H\}$ NMR (101 MHz, 298 K, C_6D_6): δ = 138.54 (s, *Ar*), 131.31 (s, *Ar*), 128.33 (s, *Ar*), 121.13 (s, *Ar*), 82.50 (s, *OBpin*), 65.75 (s, O–CH_2), 24.30 (s, *OBpin*) ppm.

Mass spectrometry (APCI): $C_{13}H_{18}B_1O_3$+Na ($[M+Na]^+$): calcd: 335.0430; found: 335.0548.

NMR conv.: >99%

Isolated Yield: 93%

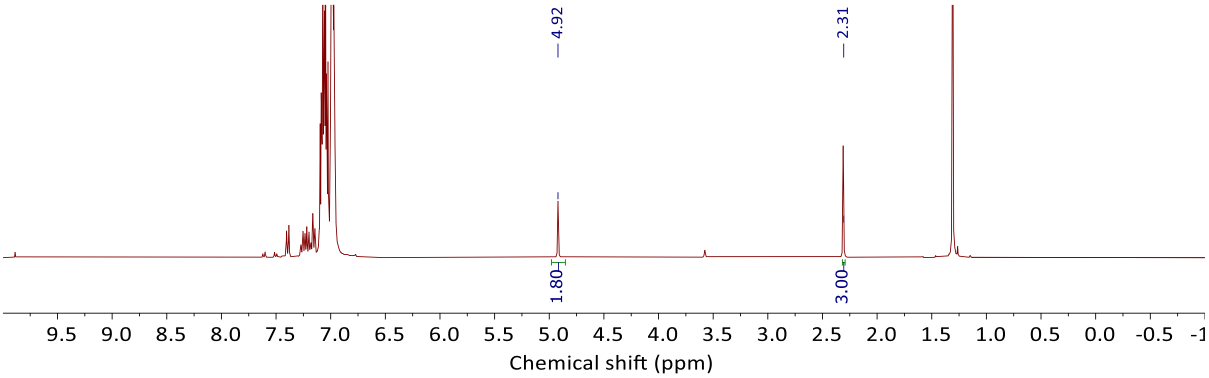

Figure S31. ^{1}H NMR spectrum (*o*DFB) of crude **8b**.

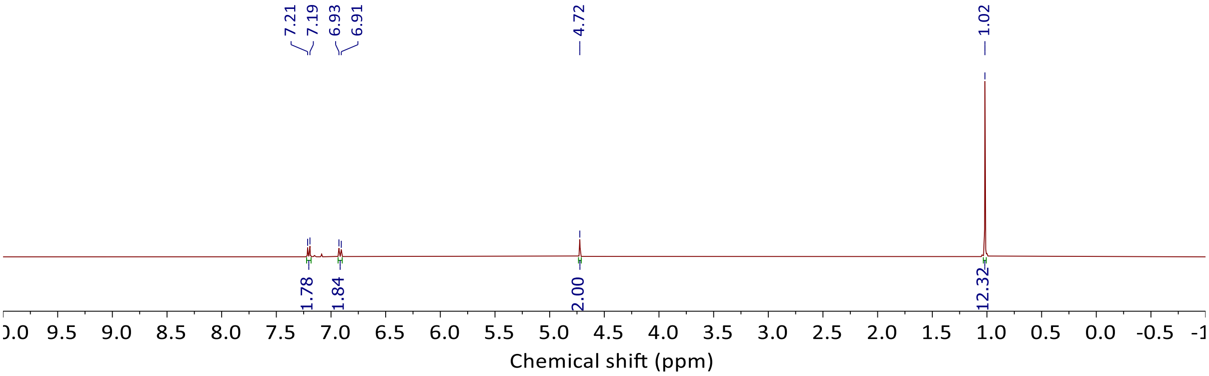

Figure S32. ^{1}H NMR spectrum (C_6D_6) of isolated **8b**.

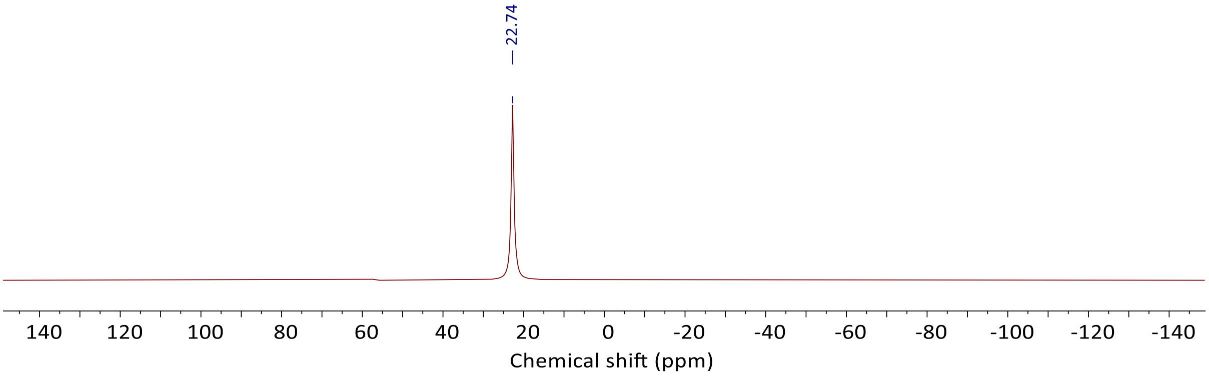

Figure S33. ^{11}B NMR spectrum (C_6D_6) of isolated **8b**.

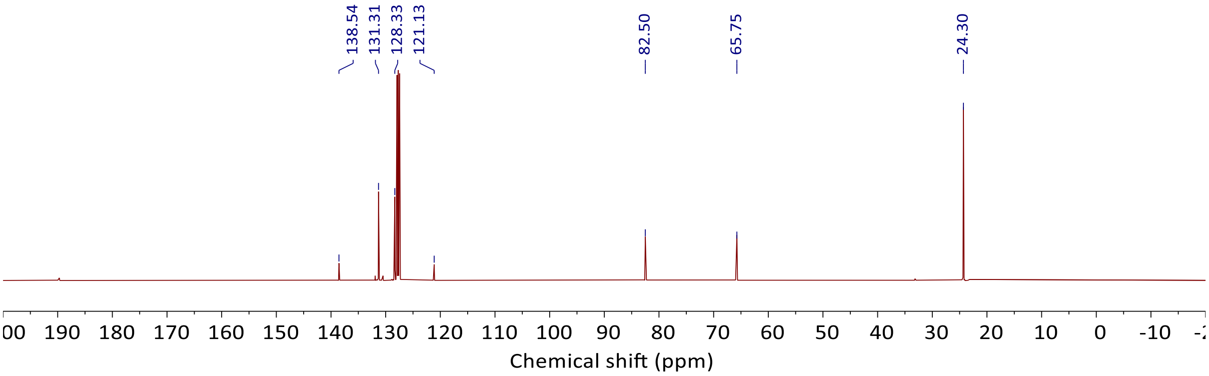

Figure S34. $^{13}C\{^{1}H\}$ NMR spectrum (C_6D_6) of isolated **8b**.

3.2.1.7. 2-((2-methylbenzyl)oxy)-4,4,5,5-tetramethyl-1,3,2-dioxaborolane

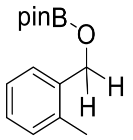

^{1}H NMR (400 MHz, 298 K, C_6D_6): δ = 7.53 (d, $^{3}J_{HH}$ = 5.5 Hz, 1H, *Ar*), 7.11 – 7.01 (m, 2H, *Ar*), 6.94 (d, $^{3}J_{HH}$ = 6.9 Hz, 1H, *Ar*), 4.96 (s, 2H, O–CH_2), 2.06 (s, 3H, Ar–*Me*), 1.04 (s, 12H, *OBpin*) ppm.

^{11}B NMR (128 MHz, 298 K, C_6D_6): δ = 22.8 (s) ppm.

$^{13}C\{^{1}H\}$ NMR (101 MHz, 298 K, C_6D_6): δ = 137.52 (s, *Ar*), 135.42 (s, *Ar*), 129.92 (s, *Ar*), 127.35 (s, *Ar*), 127.14 (s, *Ar*), 125.85 (s, *Ar*), 82.35 (s, *OBpin*), 64.96 (s, O–CH_2), 24.33 (s, *OBpin*), 18.20 (s, Ar–*Me*) ppm.

Mass spectrometry (APCI): $C_{14}H_{22}B_1O_3$+H ([M+H]$^+$): calcd: 249.1662; found: 249.1640.

NMR conv.: >99%

Isolated Yield: 92%

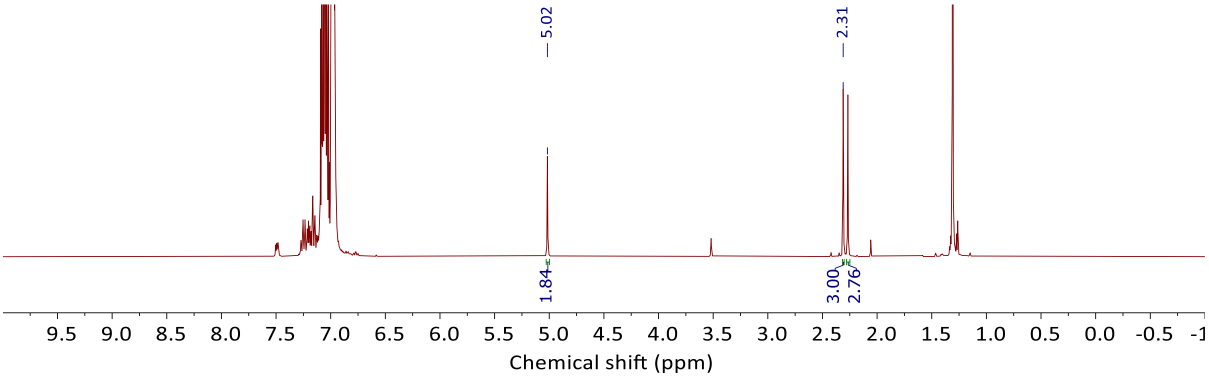

Figure S35. ^{1}H NMR spectrum (*o*DFB) of crude **9b**.

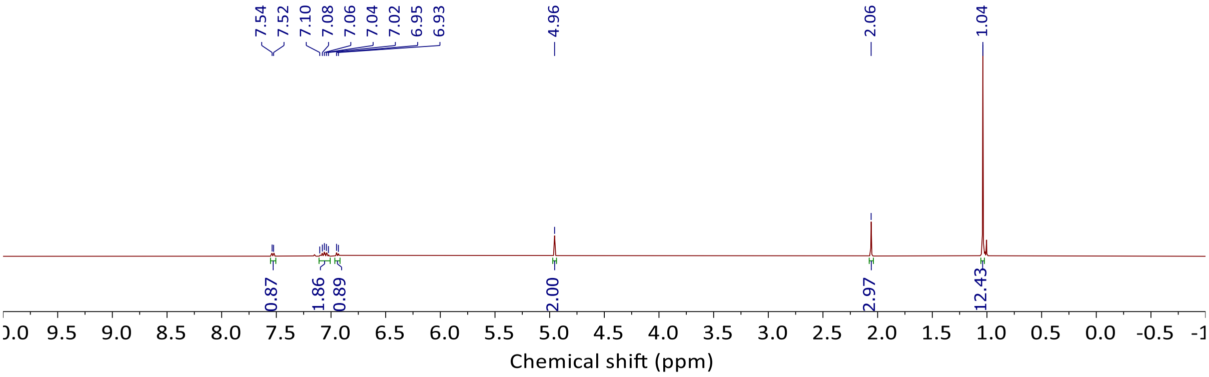

Figure S36. ^{1}H NMR spectrum (C_6D_6) of isolated **9b**.

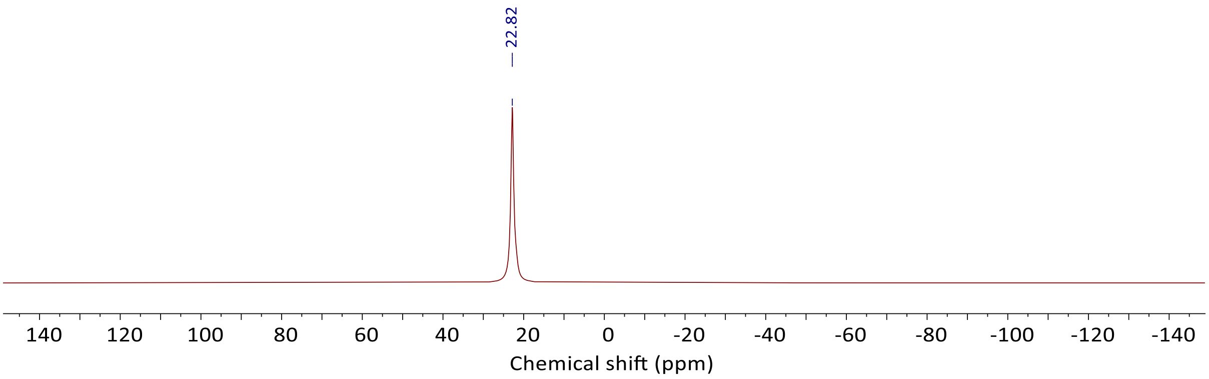

Figure S37. ^{11}B NMR spectrum (C_6D_6) of isolated **9b**.

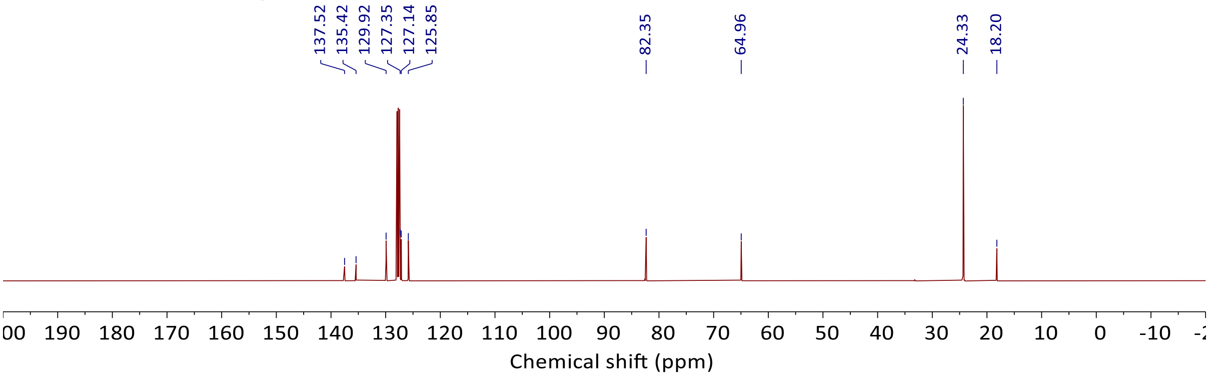

Figure S38. ^{13}C{^{1}H} NMR spectrum (C_6D_6) of isolated **9b**.

3.2.1.8. 2-((2-methoxybenzyl)oxy)-4,4,5,5-tetramethyl-1,3,2-dioxaborolane

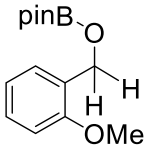

^{1}H NMR (400 MHz, 298 K, C_6D_6): δ = 7.67 (d, $^{3}J_{HH}$ = 7.4 Hz, 1H, *Ar*), 7.09 – 7.03 (m, 1H, *Ar*), 6.89 (t, $^{3}J_{HH}$ = 8.1 Hz, 1H, *Ar*), 6.46 (d, $^{3}J_{HH}$ = 6.9 Hz, 1H, *Ar*), 5.30 (s, 2H, O–C*H*$_2$), 3.22 (s, 3H, *OMe*), 1.04 (s, 12H, *OBpin*) ppm

^{11}B NMR (128 MHz, 298 K, C_6D_6): δ = 22.9 (s) ppm.

^{13}C{^{1}H} NMR (101 MHz, 298 K, C_6D_6): 156.44 (s, *Ar*), 128.19 (s, *Ar*), 127.98 (s, *Ar*), 127.12 (s, *Ar*), 120.42 (s, *Ar*), 109.69 (s, *Ar*), 82.29 (s, *OBpin*), 62.28 (s, O–*CH*$_2$), 54.31 (s, *OMe*), 24.34 (s, *OBpin*) ppm.

Mass spectrometry (APCI): $C_{14}H_{22}B_1O_4$+Na ([M+Na]$^+$): calcd: 287.1431; found: 287.1469.

NMR conv.: >99%

Isolated Yield: 90%

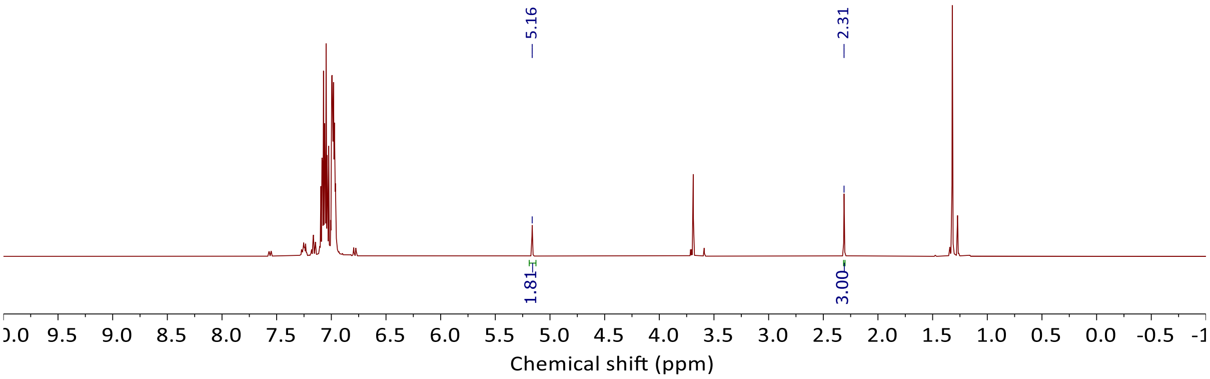

Figure S39. ^{1}H NMR spectrum (*o*DFB) of crude **10b**.

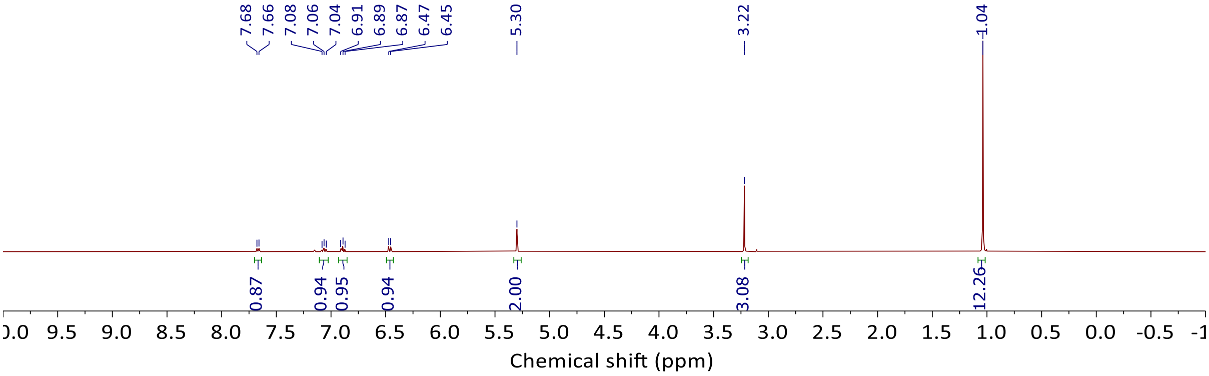

Figure S40. ^{1}H NMR spectrum (C_6D_6) of isolated **10b**.

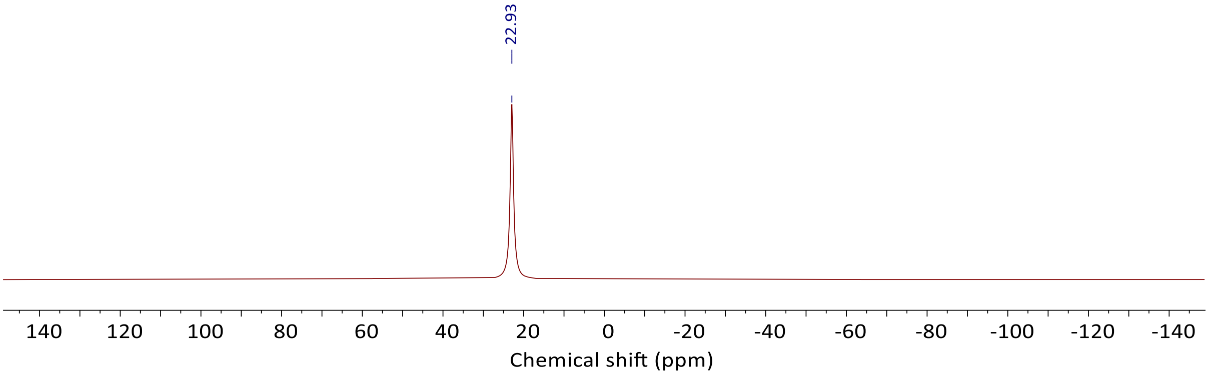

Figure S41. ^{11}B NMR spectrum (C_6D_6) of isolated **10b**.

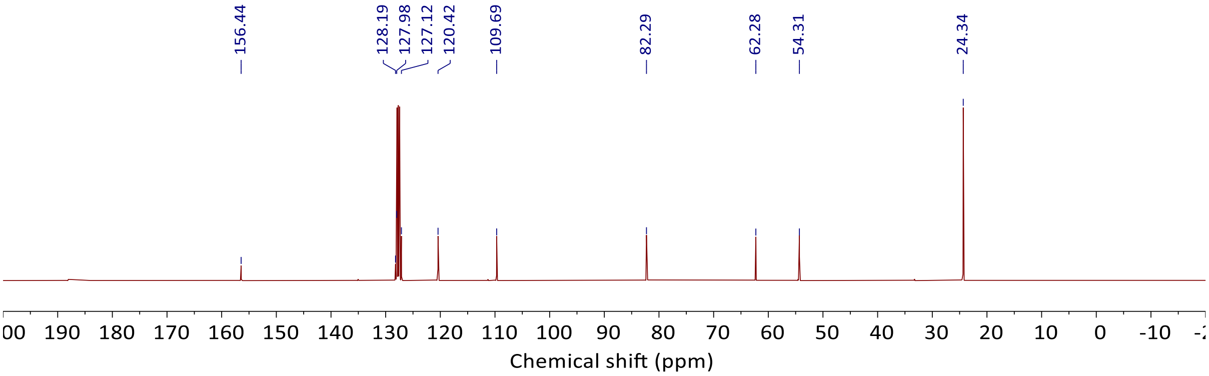

Figure S42. ^{13}C{^{1}H} NMR spectrum (C_6D_6) of isolated **10b**.

3.2.1.9. 2-(but-2-en-1-yloxy)-4,4,5,5-tetramethyl-1,3,2-dioxaborolane

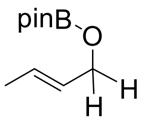

^{1}H NMR (400 MHz, 298 K, C_6D_6): δ = 5.66 – 5.52 (m, 2H, C*H*=C*H*–CH$_2$O), 4.43 – 4.37 (m, 2H, O–C*H$_2$*), 1.51 – 1.43 (m, 3H, *Me*CH=CH), 1.04 (s, 12H, *OBpin*).

^{11}B NMR (128 MHz, 298 K, C_6D_6): δ = 22.7 (s) ppm.

^{13}C{^{1}H} NMR (101 MHz, 298 K, C_6D_6): δ = 128.97 (s, MeCH=*C*H), 126.93 (s, Me*C*H=CH), 82.12 (s, *OBpin*), 65.29 (s, CH=CH–*C*H$_2$O), 24.36 (s, *OBpin*), 17.27 (s, *Me*CH=CH) ppm.

Mass spectrometry (APCI): $C_{15}H_{21}B_1O_3$+Na ([M–H]$^-$): calcd: 197.1349; found: 197.1342.

NMR conv.: 80%

Isolated Yield: 67%

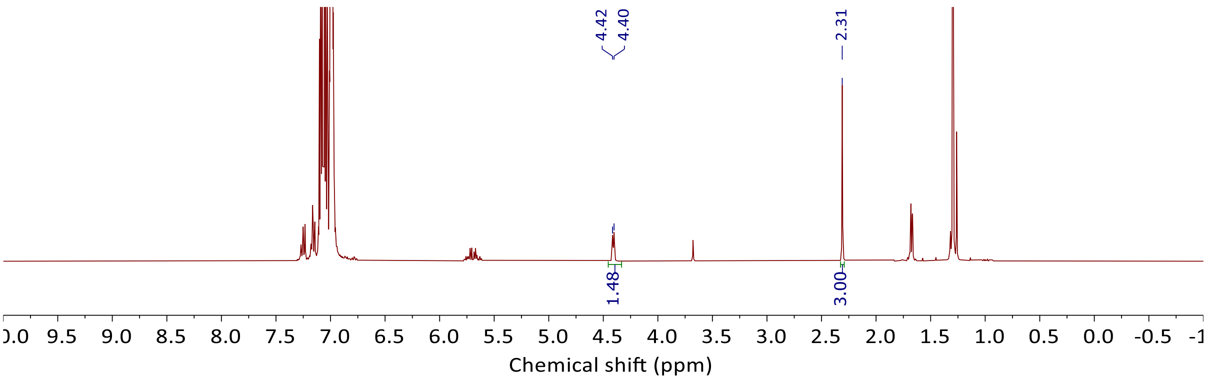

Figure S43. ^{1}H NMR spectrum (*o*DFB) of crude **11b**.

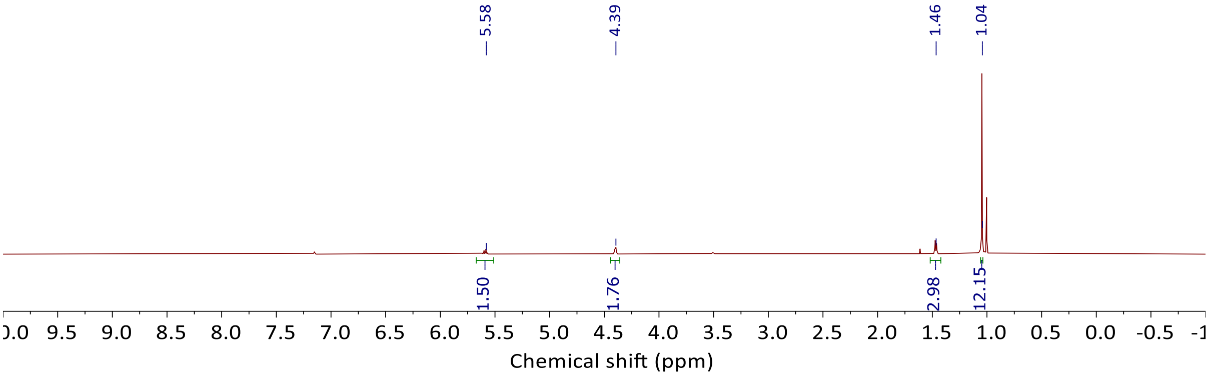

Figure S44. ^{1}H NMR spectrum (C_6D_6) of isolated **11b**.

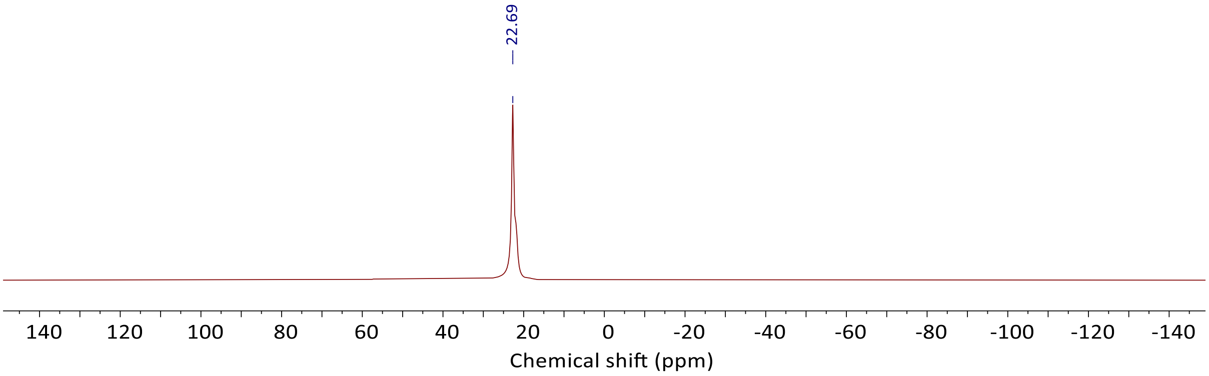

Figure S45. ^{11}B NMR spectrum (C_6D_6) of isolated **11b**.

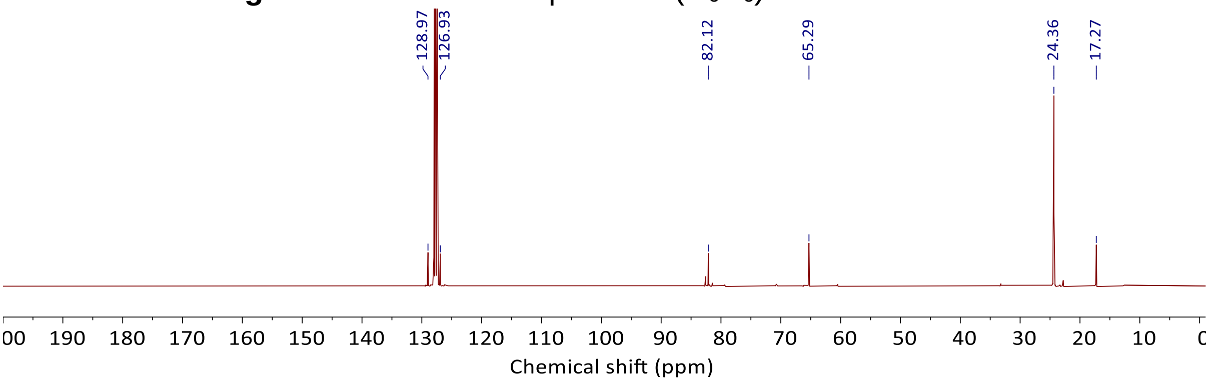

Figure S46. $^{13}C\{^{1}H\}$ NMR spectrum (C_6D_6) of isolated **11b**.

3.2.1.10. 2-[(3-phenylprop-2-en-1-yl)oxy]-4,4,5,5-tetramethyl-1,3,2-dioxaborolane

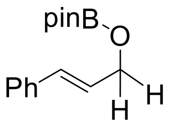

^{1}H NMR (400 MHz, 298 K, C_6D_6): δ = 7.20 – 7.14 (m, 2H, *Ar*), 7.07 (t, $^{3}J_{HH}$ = 7.3 Hz, 2H, *Ar*), 7.02 (d, $^{3}J_{HH}$ = 7.2 Hz, 1H, *Ar*), 6.60 (d, $^{3}J_{HH}$ = 15.9 Hz, 1H, PhC*H*=CH–CH$_2$O), 6.17 (dt, $^{3}J_{HH}$ = 15.9, 5.3 Hz, 1H, PhCH=C*H*–CH$_2$O), 4.53 (dd, $^{3}J_{HH}$ = 5.3, $^{4}J_{HH}$ = 1.7 Hz, 2H, O–C*H$_2$*), 1.06 (s, 12H, *OBpin*) ppm.

^{11}B NMR (128 MHz, 298 K, C_6D_6): δ = 22.8 (s) ppm.

^{13}C{^{1}H} NMR (101 MHz, 298 K, C_6D_6): δ = 136.99 (s, *Ar*), 130.54 (s, *Ar*), 128.40 (s, *Ar*), 127.32 (s, *Ar*), 127.16 (s, Ph*C*H=CH–CH$_2$O), 126.48 (s, PhCH=*C*H–CH$_2$O), 82.33 (s, *OBpin*), 65.16 (s, O–*C*H$_2$), 24.36 (s, *OBpin*) ppm.

Mass spectrometry (APCI): $C_{15}H_{21}B_1O_3$+Na ([M+Na]$^+$): calcd: 283.1481; found: 283.1570.

NMR conv.: >99%

Isolated Yield: 91%

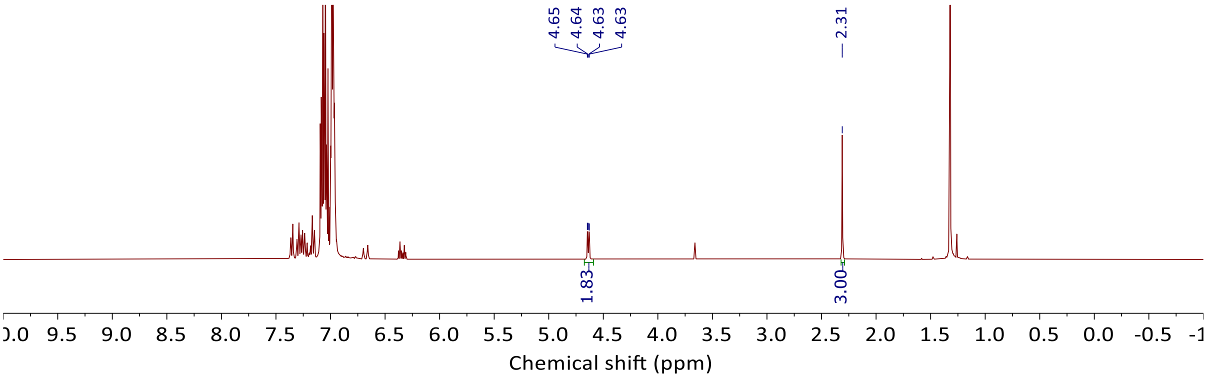

Figure S47. ^{1}H NMR spectrum (*o*DFB) of crude **12b**.

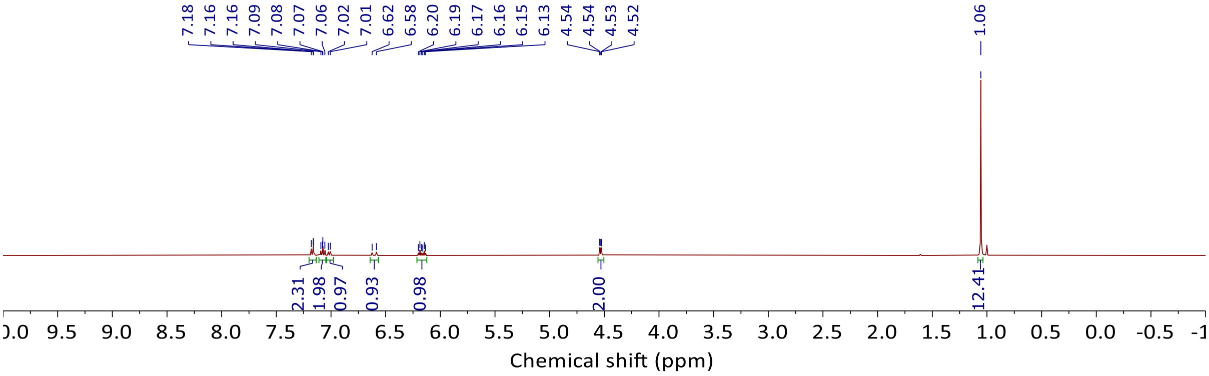

Figure S48. ^{1}H NMR spectrum (C_6D_6) of isolated **12b**.

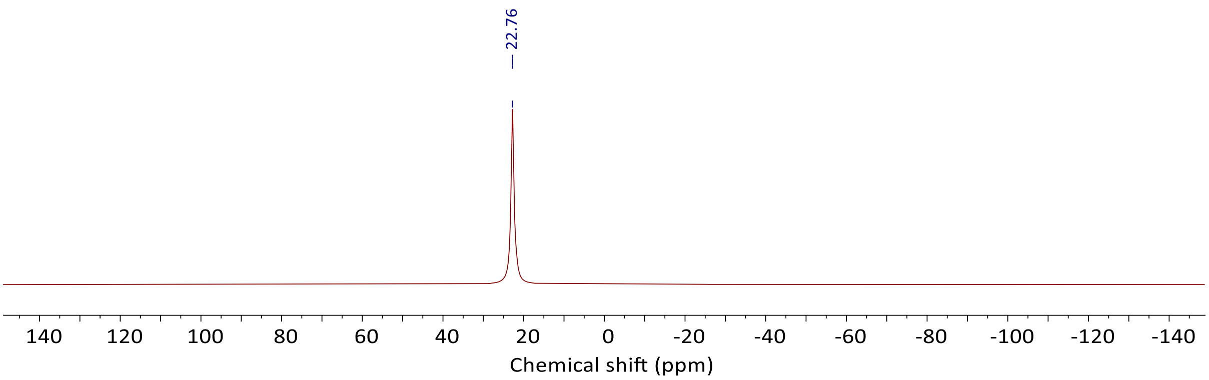

Figure S49. ^{11}B NMR spectrum (C_6D_6) of isolated **12b**.

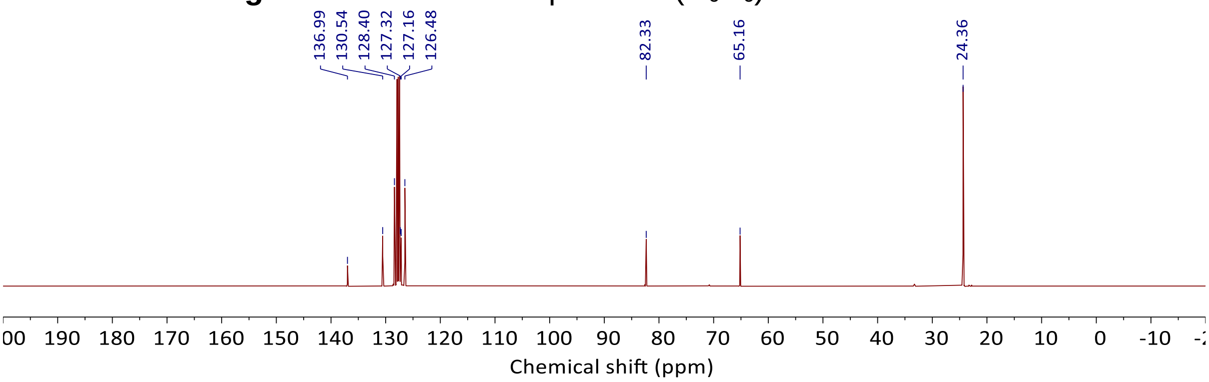

Figure S50. ^{13}C{^{1}H} NMR spectrum (C_6D_6) of isolated **12b**.

3.2.1.11. 2-ethoxy-4,4,5,5-tetramethyl-1,3,2-dioxaborolane

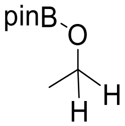

^{1}H NMR (400 MHz, 298 K, Reaction mixture): δ = 3.96 (q, $^{3}J_{HH}$ = 7.0 Hz, 2H, CH_3–C*H*$_2$–O), 1.26 (s, 12H, *OBpin*), 1.25 (t, $^{3}J_{HH}$ = 7.1 Hz, 3H, C*H*$_3$–CH_2–O).

^{11}B NMR (128 MHz, 298 K, Reaction mixture): δ = 21.7 (s) ppm.

NMR conv.: 76%

Note: Compound was not observed by mass spectrometry (GCMS, ESI or APCI), possibly due to fragmentation and the low molecular mass of the compound.

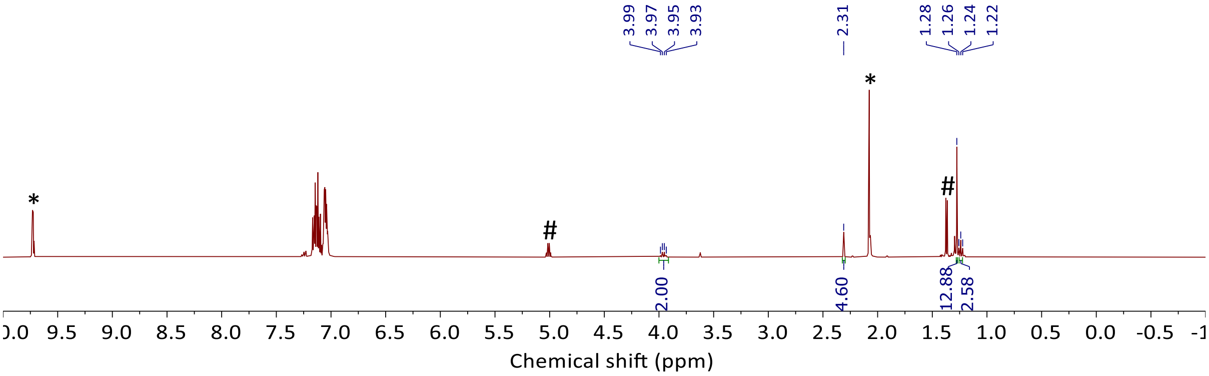

Figure S51. ^{1}H NMR spectrum (*o*DFB) of crude **13b**. Excess acetaldehyde marked by *. Paraldehyde marked by #.

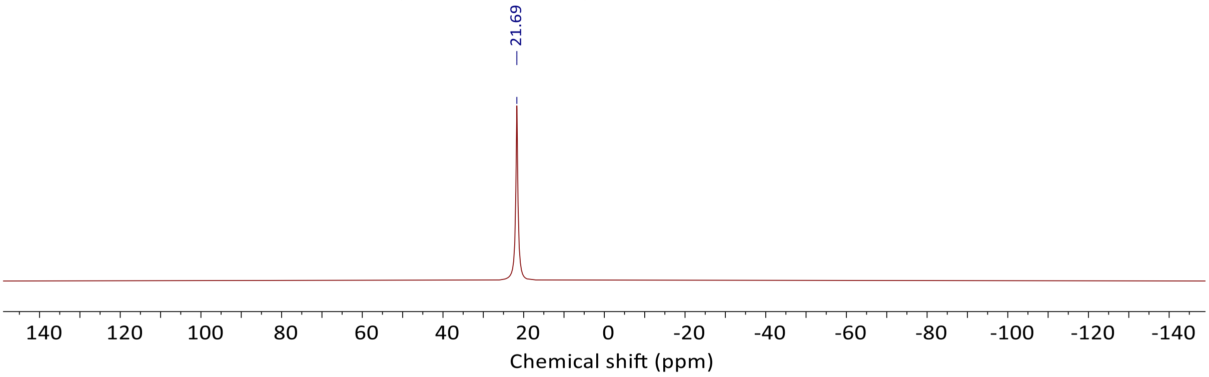

Figure S52. ^{11}B NMR spectrum (*o*DFB) of crude **13b**.

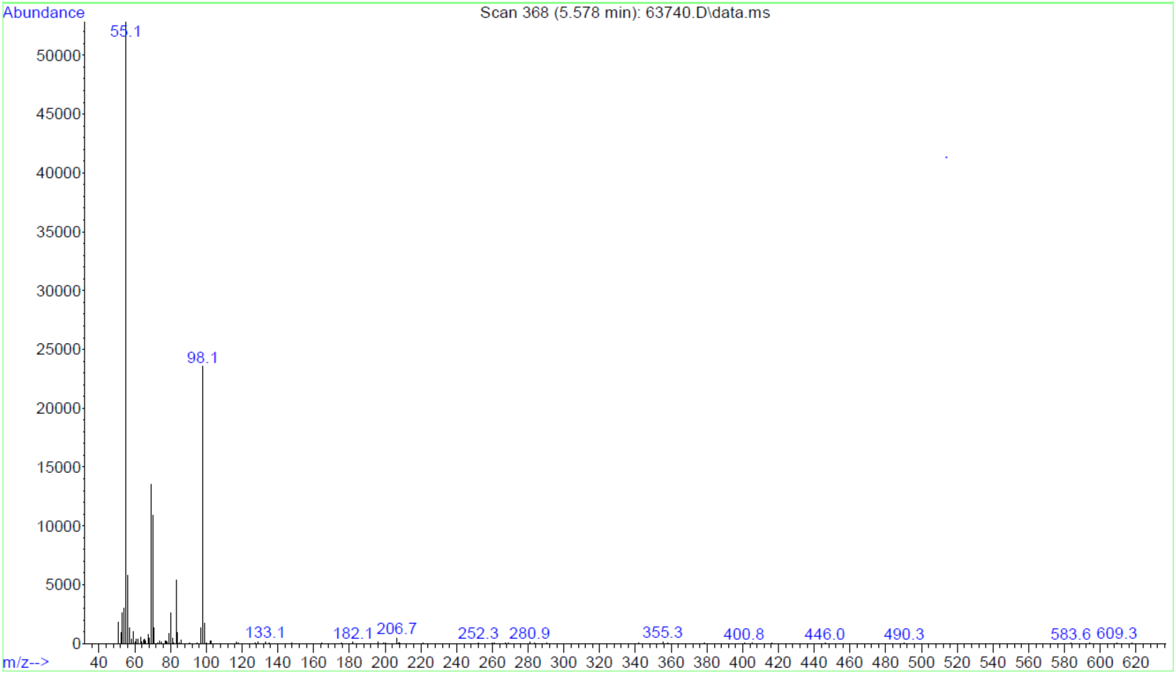

Figure S53. Mass spectrum (GC-MS) of crude **13b**.

3.2.2. Characterization Data Hydroboration of Ketones

3.2.2.1. 2-[(benzhydryl)oxy]-4,4,5,5-tetramethyl-1,3,2-dioxaborolane

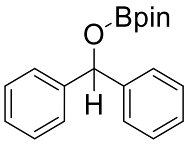

^{1}H NMR (400 MHz, 298 K, C_6D_6): δ = 7.45 - 7.41 (m, 4H, *Ar*), 7.11 - 7.05 (m, 4H, *Ar*), 7.03 - 6.96 (m, 2H, *Ar*), 6.42 (s, 1H, O–C*H*), 0.97 (s, 12H, *OBpin*) ppm.

^{11}B NMR (128 MHz, 298 K, C_6D_6): δ = 22.8 (s) ppm.

^{13}C{^{1}H} NMR (101 MHz, 298 K, C_6D_6): δ = 143.53 (s, *Ar*), 128.22 (s, *Ar*), 127.19 (s, *Ar*), 126.60 (s, *Ar*), 82.48 (s, *OBpin*), 78.17 (s, O–CH), 24.24 (s, *OBpin*) ppm.

Mass spectrometry (APCI): $C_{19}H_{23}B_1O_3$+Na ([M+Na]$^+$): calcd: 333.1632; found: 333.1640.

NMR conv.: 99%

Isolated Yield: 96%

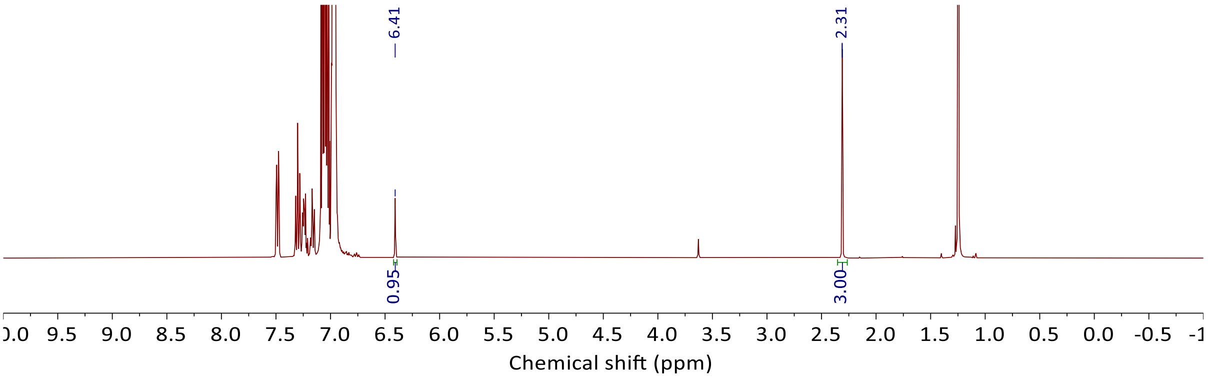

Figure S54. ^{1}H NMR spectrum (*o*DFB) of crude **3b**.

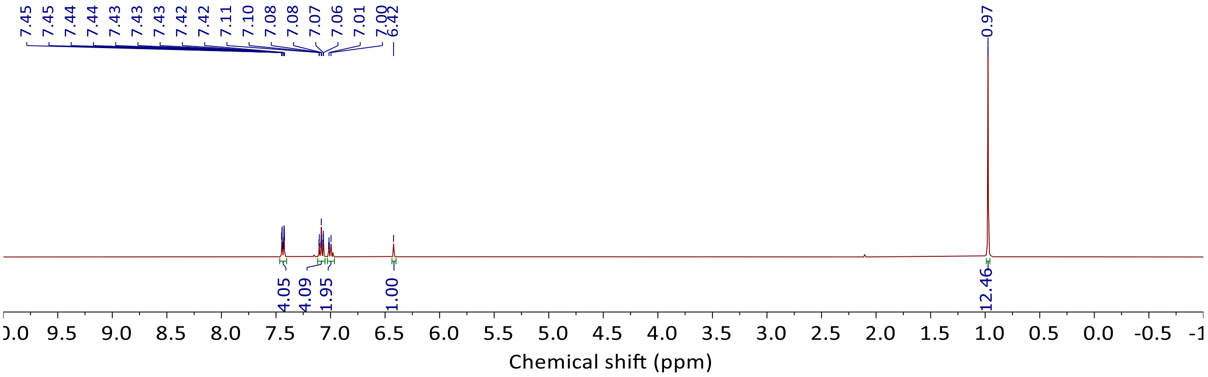

Figure S55. ^{1}H NMR spectrum (C_6D_6) of isolated **3b**.

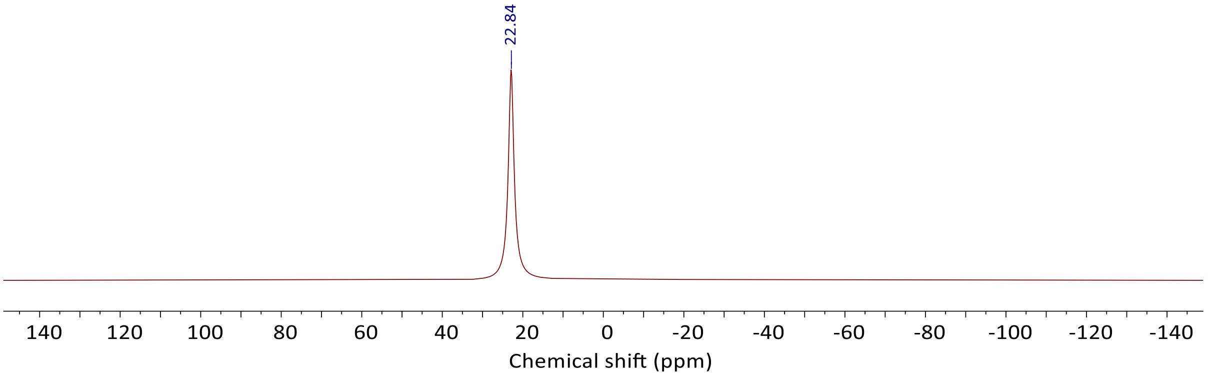

Figure S56. ^{11}B NMR spectrum (C_6D_6) of isolated **3b**.

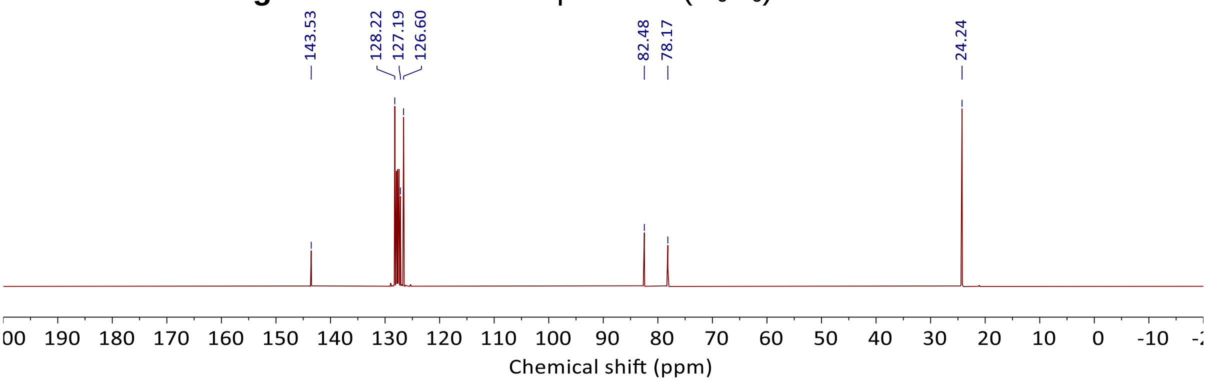

Figure S57. ^{13}C{^{1}H} NMR spectrum (C_6D_6) of isolated **3b**.

3.2.2.2. 2-(2-pyridylmethoxy)-4,4,5,5-tetramethyl-1,3,2-dioxaborolane

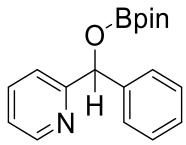

^{1}H NMR (400 MHz, 298 K, C_6D_6): δ = 8.30 (d, $^3J_{HH}$ = 4.6 Hz, 1H, *Ar*), 7.61 (d, $^3J_{HH}$ = 7.0 Hz, 2H, *Ar*), 7.09 (t, $^3J_{HH}$ = 7.5 Hz, 2H, *Ar*), 7.03 – 6.93 (m, 2H, *Ar*), 6.80 (t, $^3J_{HH}$ = 7.7 Hz, 1H, *Ar*), 6.41 – 6.36 (m, 1H, *Ar*), 6.33 (s, 1H, O–C*H*), 1.26 (s, 12H, *OBpin*) ppm.

^{11}B NMR (128 MHz, 298 K, C_6D_6): δ = 17.9 (s) ppm.

^{13}C{^{1}H} NMR (101 MHz, 298 K, C_6D_6): δ = 162.30 (s, *Ar*), 144.29 (s, *Ar*), 142.58 (s, *Ar*), 128.31 (s, *Ar*), 127.54 (s, *Ar*), 126.79 (s, *Ar*), 122.31 (s, *Ar*), 119.94 (s, *Ar*), 81.19 (s, *OBpin*), 78.74 (s, O–CH), 25.26 (s, *OBpin*) ppm.

Mass spectrometry (APCI): $C_{18}H_{22}B_1NO_3$+H ([M+H]$^+$): calcd: 312.1769; found: 312.1765.

NMR conv.: 99%

Isolated Yield: 95%

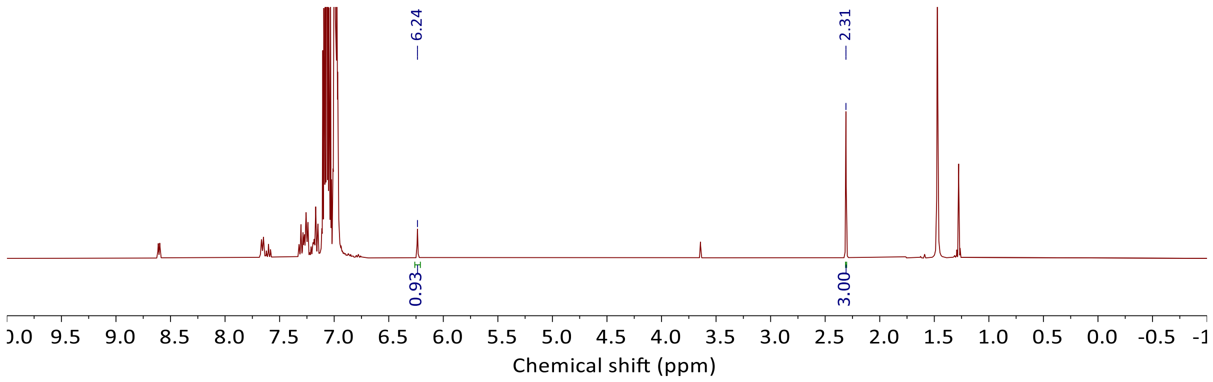

Figure S58. ^{1}H NMR spectrum (*o*DFB) of crude **14b**.

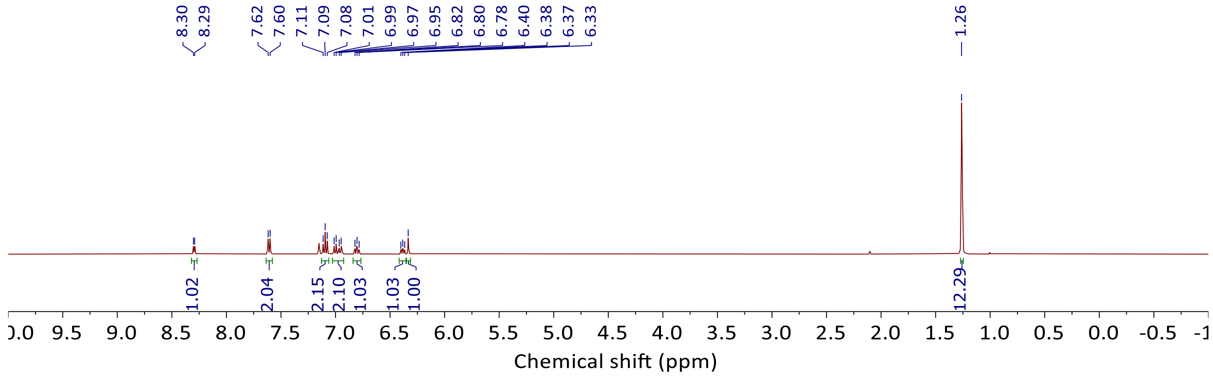

Figure S59. ^{1}H NMR spectrum (C_6D_6) of isolated **14b**.

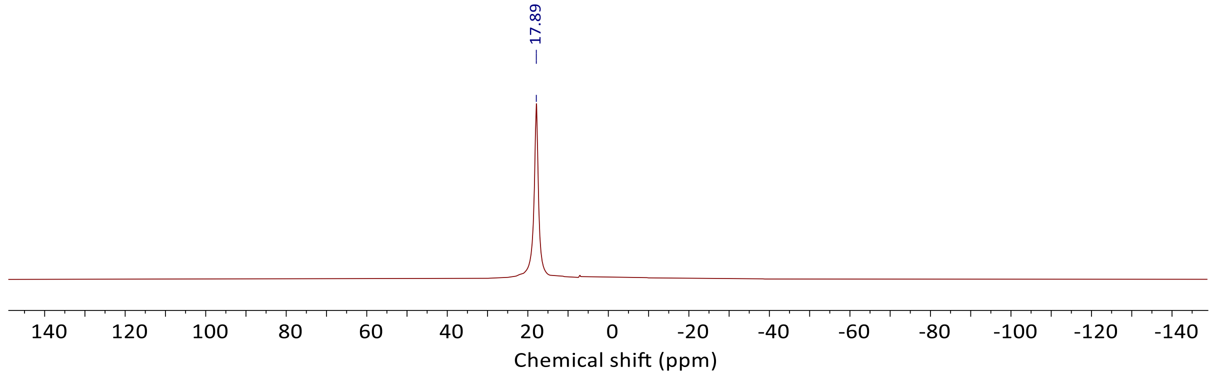

Figure S60. ^{11}B NMR spectrum (C_6D_6) of isolated **14b**.

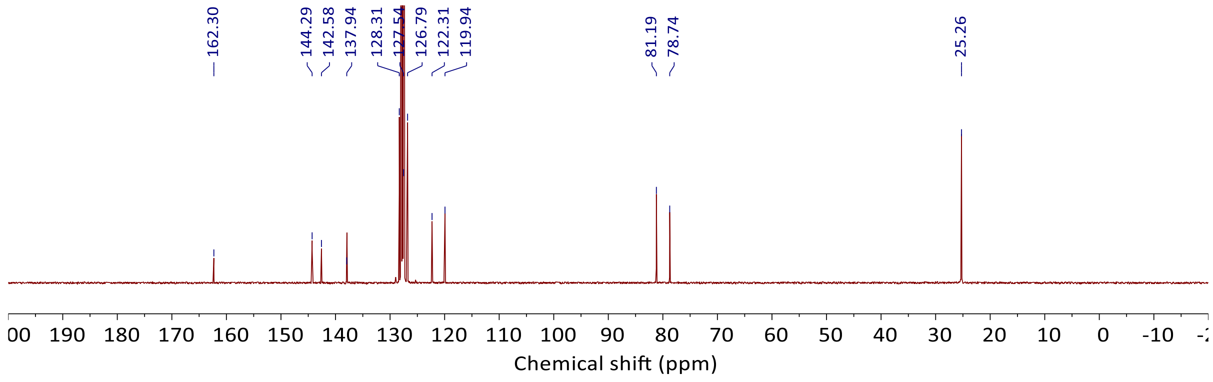

Figure S61. ^{13}C{^{1}H} NMR spectrum (C_6D_6) of isolated **14b**.

3.2.2.3. 2-[(2-pyridyl)oxy]-4,4,5,5-tetramethyl-1,3,2-dioxaborolane

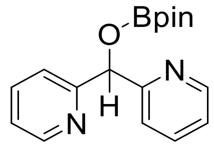

^{1}H NMR (400 MHz, 298 K, C_6D_6): δ = 8.29 (d, $^{3}J_{HH}$ = 4.5 Hz, 2H, *Ar*), 7.91 (d, $^{3}J_{HH}$ = 8.0 Hz, 2H, *Ar*), 6.89 (td, $^{3}J_{HH}$ = 7.8, $^{4}J_{HH}$ = 1.8 Hz, 2H, *Ar*), 6.46 – 6.40 (m, 2H, *Ar*), 6.35 (s, 1H, O–C*H*), 1.55 (s, 12H, *OBpin*) ppm.

^{11}B NMR (128 MHz, 298 K, C_6D_6): δ = 13.0 (s) ppm.

^{13}C{^{1}H} NMR (101 MHz, 298 K, C_6D_6): δ = 161.22 (s, *Ar*), 144.19 (s, *Ar*), 138.13 (s, *Ar*), 122.66 (s, *Ar*), 121.26 (s, *Ar*), 79.82 (s, *OBpin*), 78.33 (s, O–CH), 26.25 (s, *OBpin*) ppm.

Mass spectrometry (APCI): $C_{17}H_{21}B_1N_2O_3$+Na ([M+Na]$^+$): calcd: 335.1538; found: 335.1455.

NMR conv.: 99%

Isolated Yield: 94%

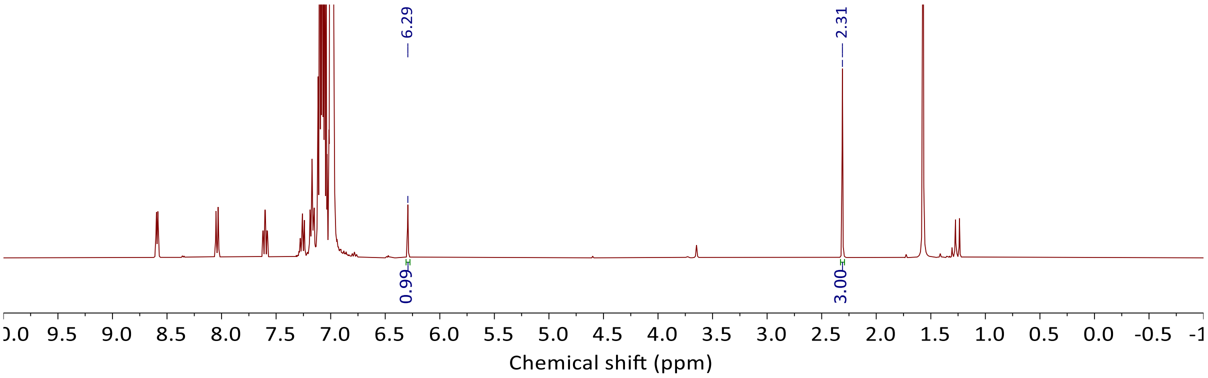

Figure S62. ^{1}H NMR spectrum (*o*DFB) of crude **15b**.

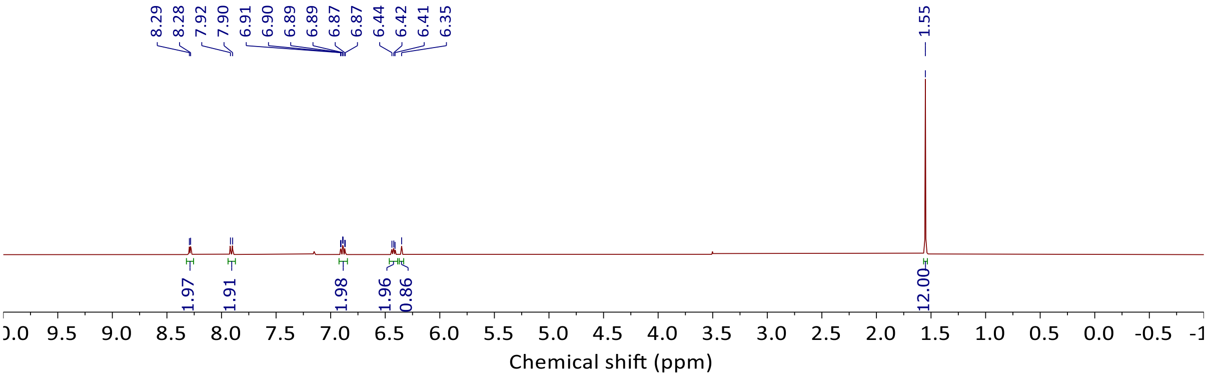

Figure S63. ^{1}H NMR spectrum (C_6D_6) of isolated **15b**.

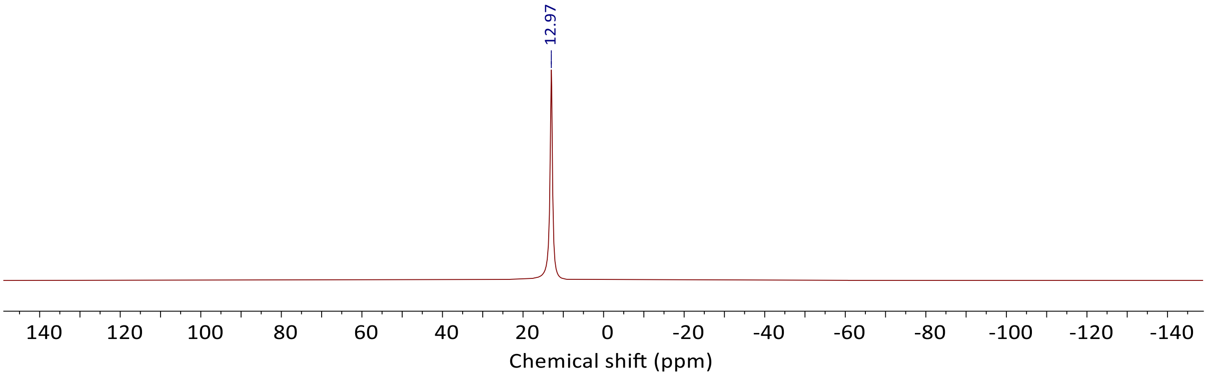

Figure S64. ^{11}B NMR spectrum (C_6D_6) of isolated **15b**.

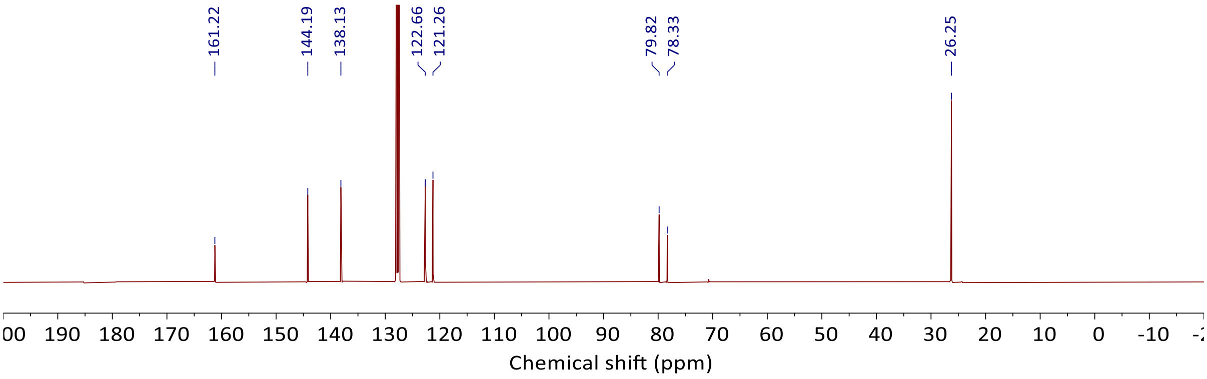

Figure S65. ^{13}C{^{1}H} NMR spectrum (C_6D_6) of isolated **15b**.

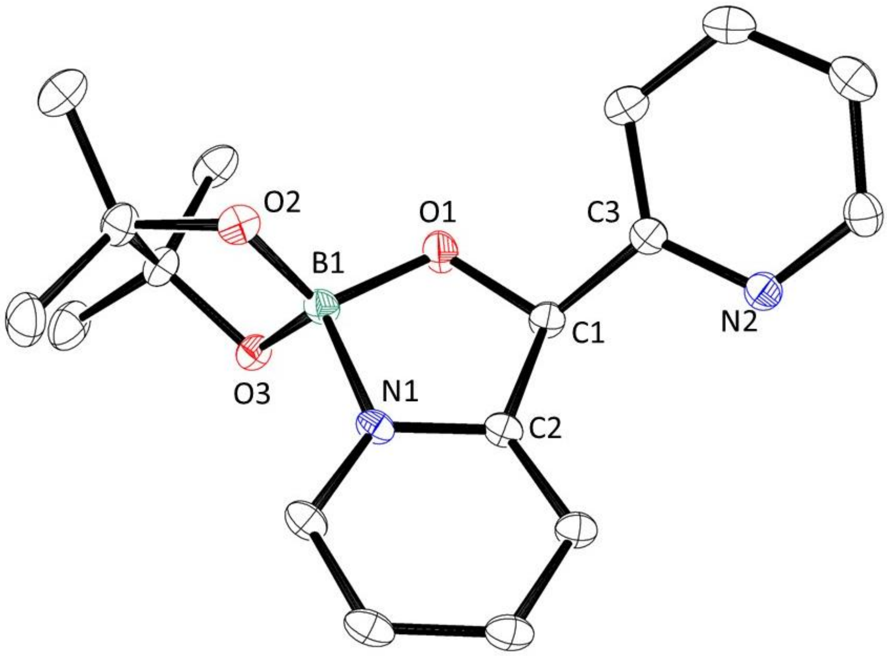

Figure S66. Molecular structure of **15b**. Anisotropic displacement ellipsoids pictured at 50% probability. Hydrogen atoms omitted for clarity. Nitrogen: Blue; Boron: Green; Carbon: White; Oxygen: Red.

3.2.2.4. 2-(1-phenylethoxy)-4,4,5,5-tetramethyl-1,3,2-dioxaborolane

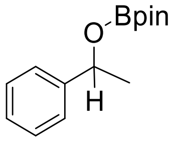

^{1}H NMR (400 MHz, 298 K, C_6D_6): δ = 7.38 - 7.34 (m, 2H, *Ar*), 7.17 - 7.10 (m, 2H, *Ar*), 7.05 (d, $^{3}J_{HH}$ = 7.4 Hz, 1H, *Ar*), 5.41 (q, $^{3}J_{HH}$ = 6.5 Hz, 1H, O–C*H*), 1.45 (d, $^{3}J_{HH}$ = 6.5 Hz, 3H, *CH$_3$*), 1.02 (s, 6H, *OBpin*), 0.99 (s, 6H, *OBpin*) ppm.

^{11}B NMR (128 MHz, 298 K, C_6D_6): δ = 22.6 (s) ppm.

^{13}C{^{1}H} NMR (101 MHz, 298 K, C_6D_6): δ = 145.02 (s, *Ar*), 128.17 (s, *Ar*), 126.98 (s, *Ar*), 125.33 (s, *Ar*), 82.16 (s, *OBpin*), 72.60 (s, O–*C*H), 25.42 (s, *CH$_3$*), 24.31 (s, *OBpin*), 24.23 (s, *OBpin*) ppm.

Mass spectrometry (ESI): $C_{14}H_{21}B_1O_3$+Na ([M+Na]$^+$): calcd: 271.1479; found: 271.1485.

NMR conv.: 99%

Isolated Yield: 96%

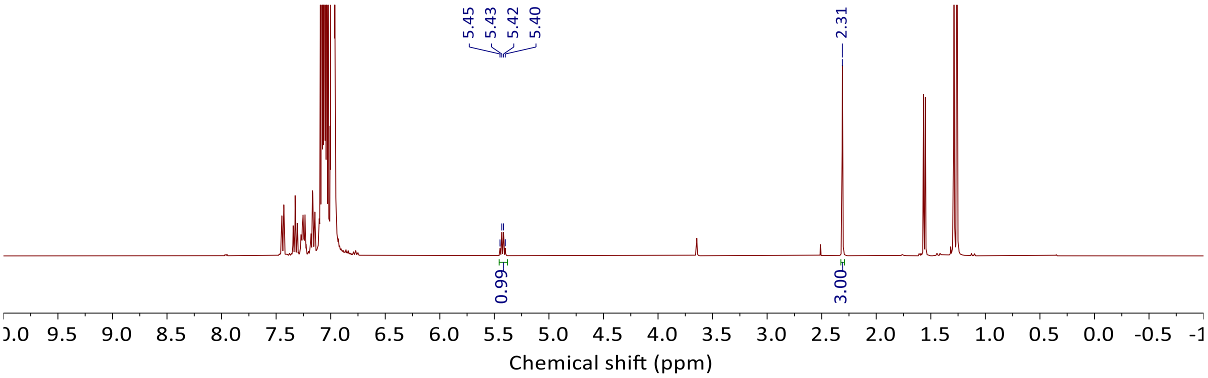

Figure S67. ^{1}H NMR spectrum (*o*DFB) of crude **16b**.

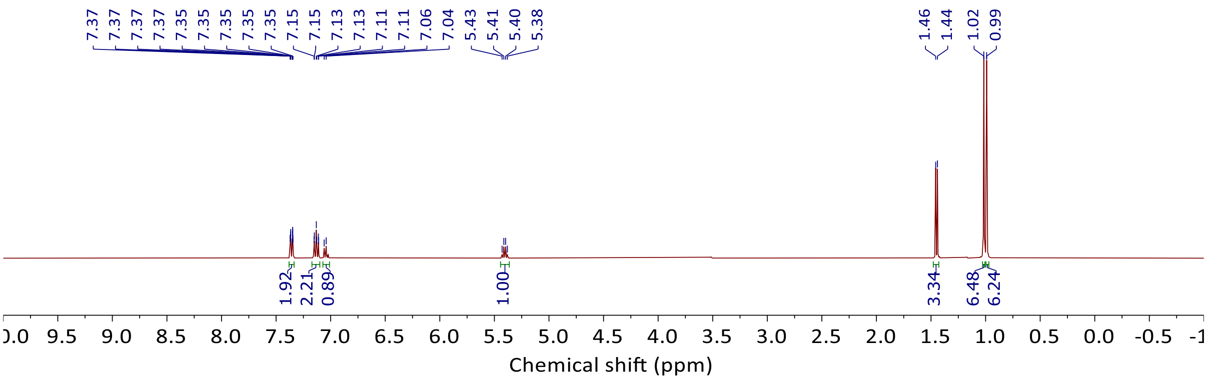

Figure S68. ^{1}H NMR spectrum (C_6D_6) of isolated **16b**.

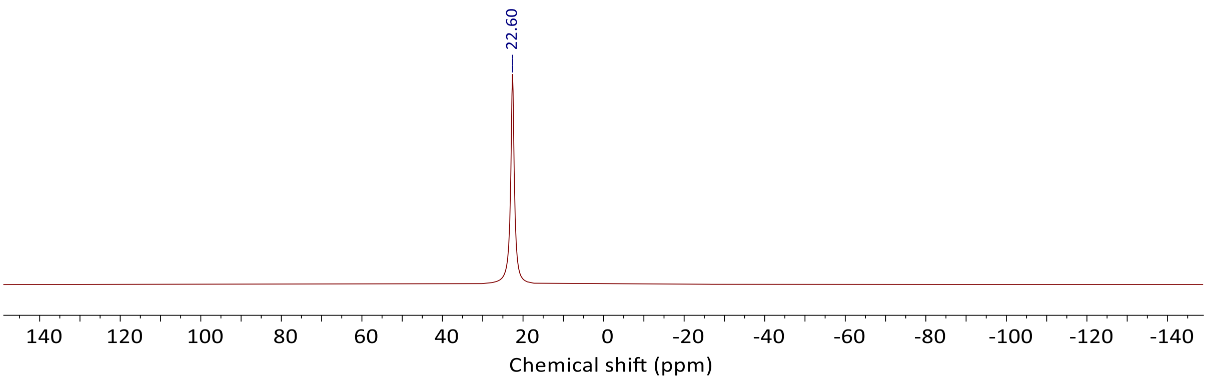

Figure S69. ^{11}B NMR spectrum (C_6D_6) of isolated **16b**.

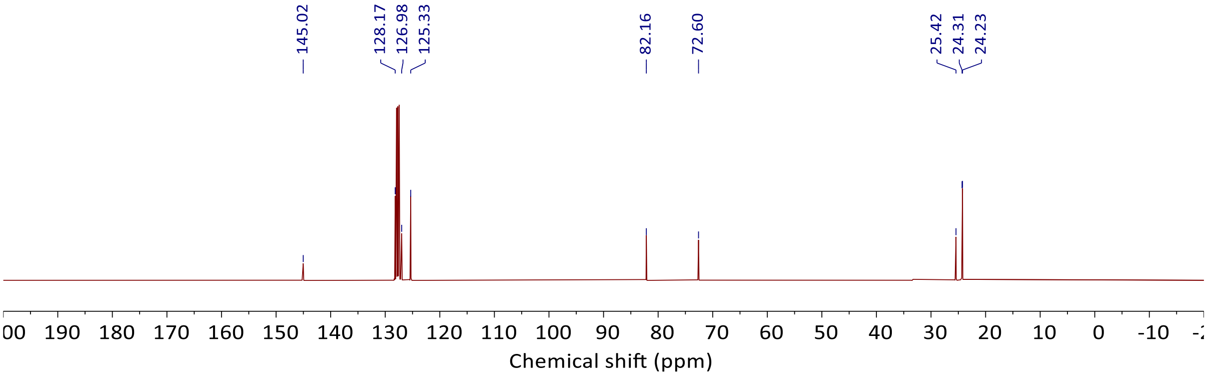

Figure S70. $^{13}C\{^1H\}$ NMR spectrum (C_6D_6) of isolated **16b**.

3.2.2.5. 2-[1-(thiophen-2-yl)ethoxy]-4,4,5,5-tetramethyl-1,3,2-dioxaborolane

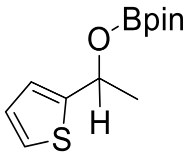

^1H NMR (400 MHz, 298 K, C_6D_6): δ = 6.85 - 6.81 (m, 2H, *Ar*), 6.68 (dd, $^3J_{HH}$ = 5.1, 3.5 Hz, 1H, *Ar*), 5.62 (qd, $^3J_{HH}$ = 6.4, $^4J_{HH}$ = 0.9 Hz, 1H, O–C*H*), 1.51 (d, $^3J_{HH}$ = 6.5 Hz, 3H, *CH_3*), 1.02 (br, 12H, *OBpin*).

^{11}B NMR (128 MHz, 298 K, C_6D_6): δ = 22.6 (s).

$^{13}C\{^1H\}$ NMR (101 MHz, 298 K, C_6D_6): δ = 148.57 (s, *Ar*), 126.25 (s, *Ar*), 123.96 (s, *Ar*), 123.15 (s, *Ar*), 82.36 (s, *OBpin*), 68.69 (s, O–*C*H), 25.01 (s, *C*H_3), 24.35 (s, *OBpin*), 24.22 (s, *OBpin*).

Mass spectrometry (APCI): $C_{12}H_{19}B_1O_3$+Na ($[M+Na]^+$): calcd: 277.1040; found: 277.1048.

NMR conv.: 99%

Isolated Yield: 90%

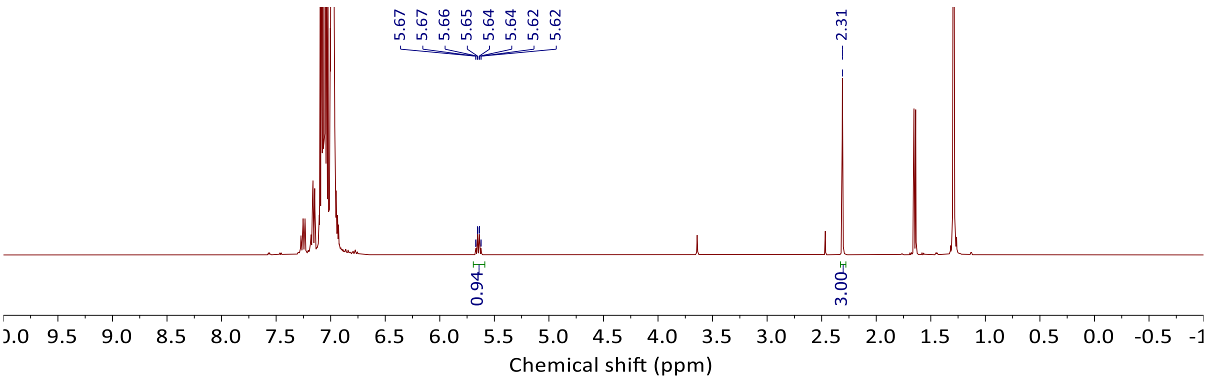

Figure S71. ^{1}H NMR spectrum (*o*DFB) of crude **17b**.

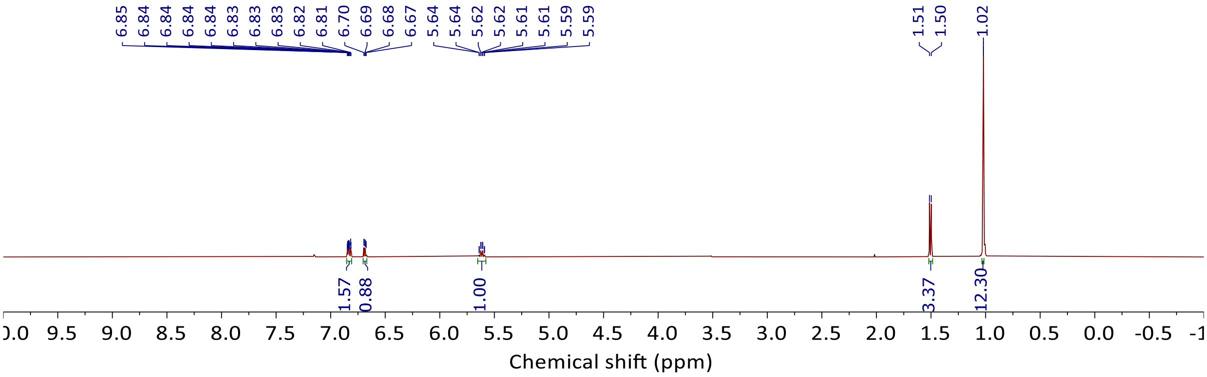

Figure S72. ^{1}H NMR spectrum (C_6D_6) of isolated **17b**.

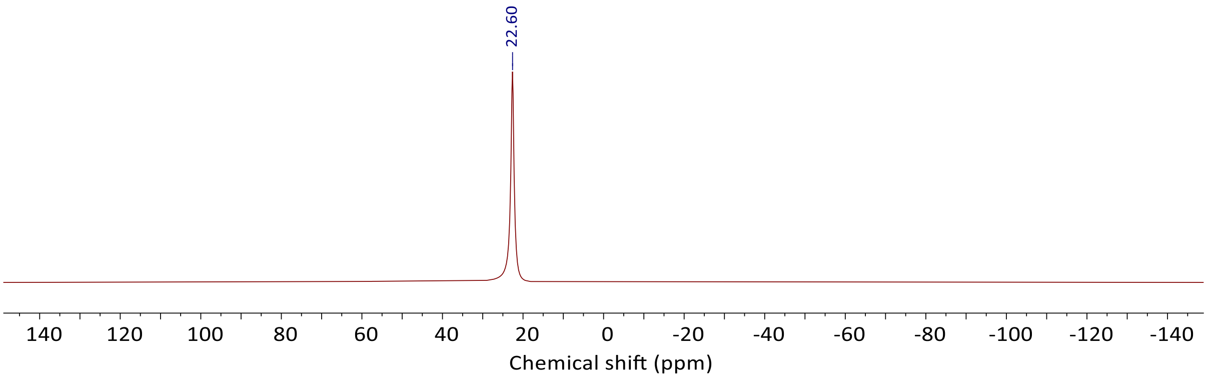

Figure S73. ^{11}B NMR spectrum (C_6D_6) of isolated **17b**.

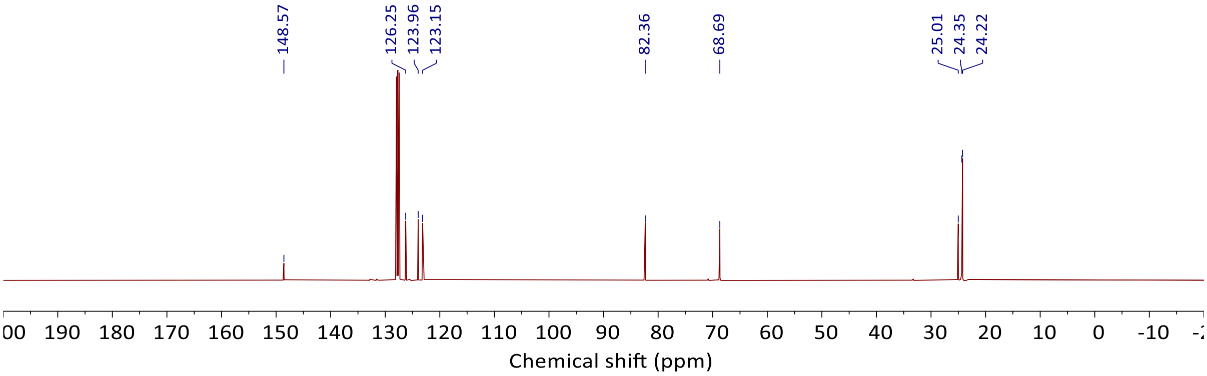

Figure S74. $^{13}C\{^{1}H\}$ NMR spectrum (C_6D_6) of isolated **17b**.

3.2.2.6. 2-(1-pentaflurorophenylethoxy)-4,4,5,5-tetramethyl-1,3,2-dioxaborolane

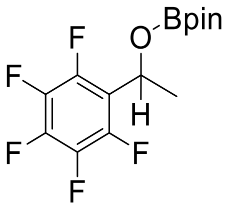

^{1}H NMR (400 MHz, 298 K, C_6D_6): δ = 5.63 (q, $^{3}J_{HH}$ = 6.7 Hz, 1H, O–C*H*), 1.43 (d, $^{3}J_{HH}$ = 6.6 Hz, 3H, C*H3*), 1.00 (s, 6H, *OBpin*), 0.98 (s, 6H, *OBpin*) ppm.

^{11}B NMR (128 MHz, 298 K, C_6D_6): δ = 22.5 (s) ppm.

^{13}C{^{1}H} NMR (101 MHz, 298 K, C_6D_6): δ = 144.69 (d of m, $^{1}J_{CF}$ = 246.5 Hz, *Ar*–F), 140.30 (d of m, $^{1}J_{CF}$ = 252.4 Hz, *Ar*–F), 137.30 (d of m, $^{1}J_{CF}$ = 251.4 Hz, *Ar*–F), 116.94 – 116.41 (m, *Ar*–F), 82.68 (s, *OBpin*), 64.04 (s, O–*C*H), 24.24 (s, *CH3*), 24.17 (s, *OBpin*), 24.10 (s, *OBpin*) ppm.

^{19}F{^{1}H} NMR (376 MHz, 298 K, C_6D_6): δ = –143.5 (dd, $^{3}J_{FF}$ = 23.5, $^{4}J_{FF}$ = 9.0 Hz), –156.36 (tt, $^{3}J_{FF}$ = 23.2, $^{4}J_{FF}$ = 3.0 Hz), –162.83 - –163.01 (m) ppm.

Mass spectrometry (APCI): $C_{14}H_{16}B_1O_3F_5$+K+$C_8H_5O_1F_5$ ([M+K+ $C_8H_5O_1F_5$]$^{+}$): calcd: 589.1010; found: 589.1029.

NMR conv.: 80%

Isolated Yield: 69%

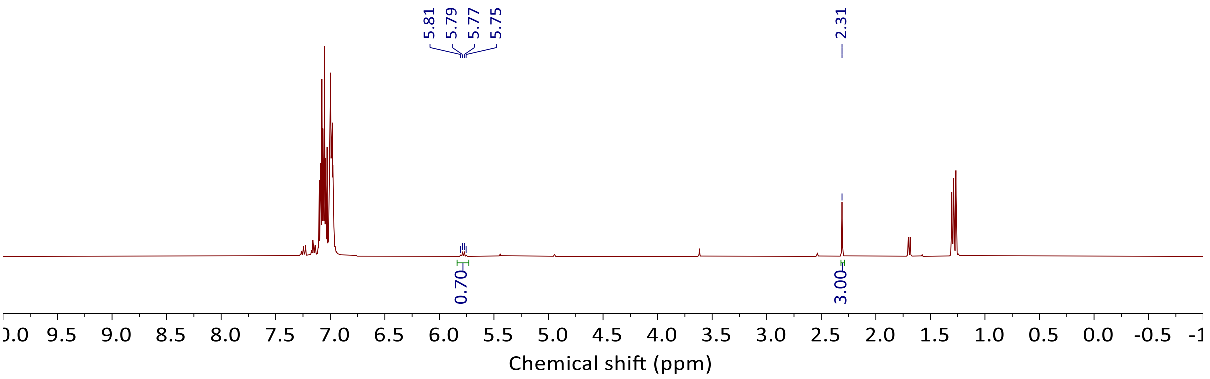

Figure S75. ^{1}H NMR spectrum (*o*DFB) of crude **18b**.

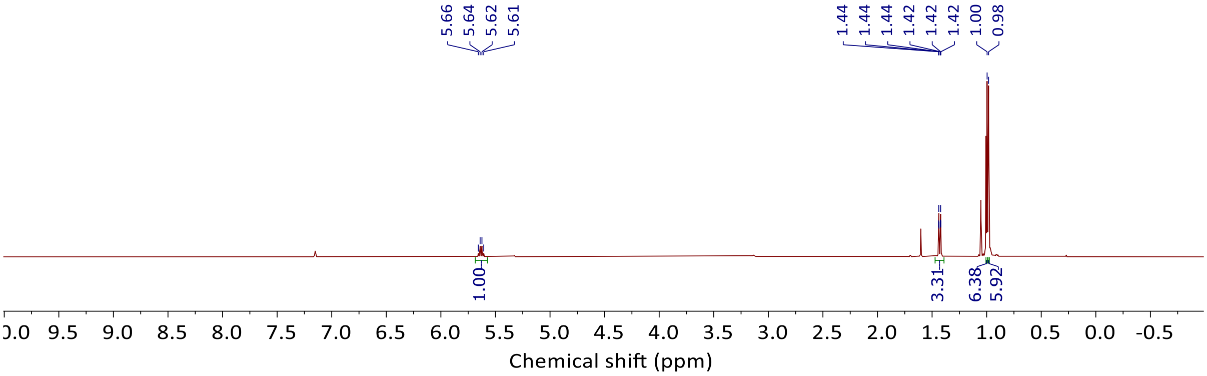

Figure S76. ^{1}H NMR spectrum (C_6D_6) of isolated **18b**.

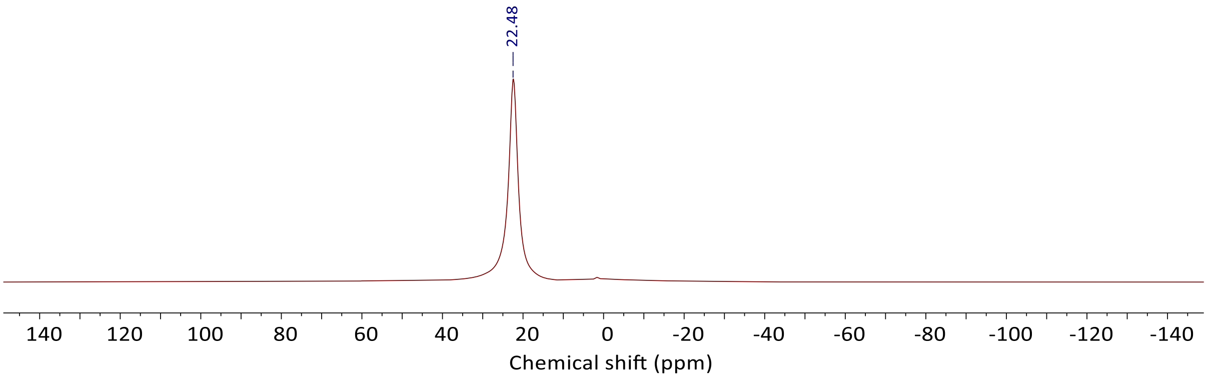

Figure S77. ^{11}B NMR spectrum (C_6D_6) of isolated **18b**.

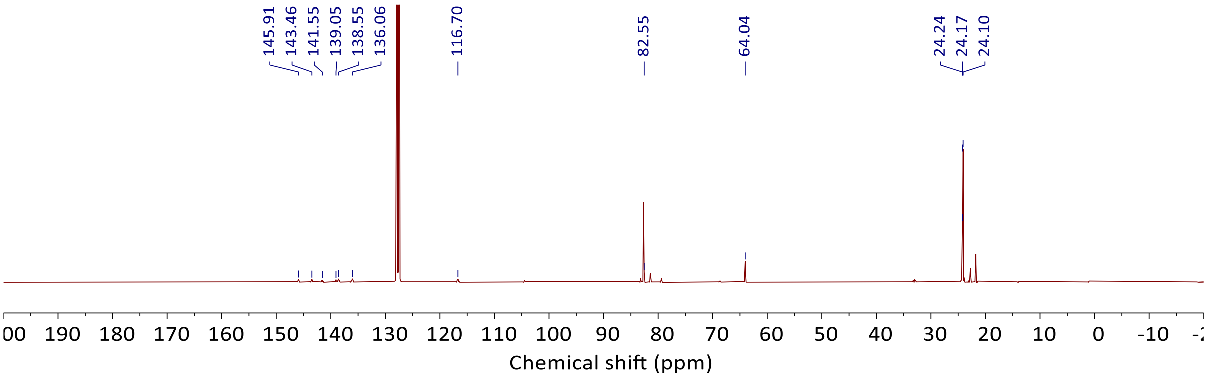

Figure S78. $^{13}C\{^{1}H\}$ NMR spectrum (C_6D_6) of isolated **18b**.

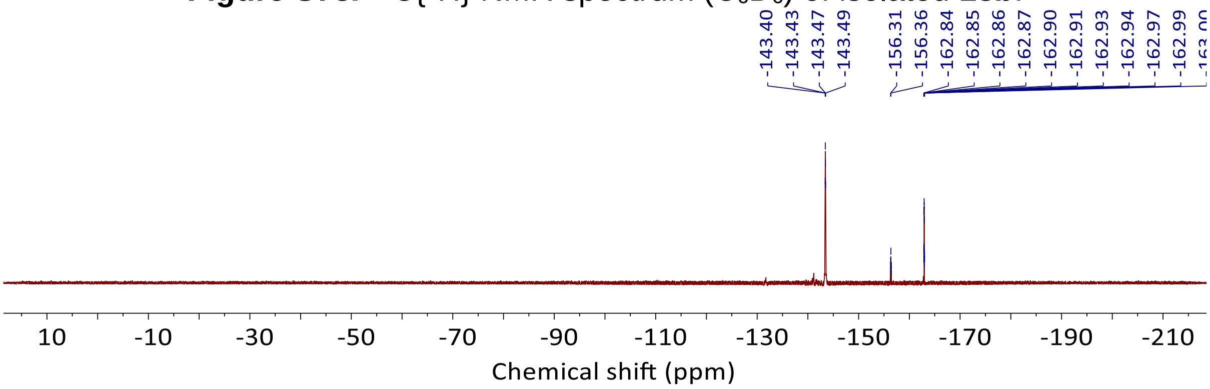

Figure S79. $^{19}F\{^{1}H\}$ NMR spectrum (C_6D_6) of isolated **18b**.

3.2.2.7. 2-(1-ferroceneethoxy)-4,4,5,5-tetramethyl-1,3,2-dioxaborolane

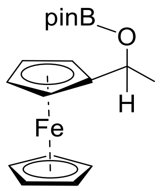

^{1}H NMR (400 MHz, 298 K, C_6D_6): δ = 5.28 (q, $^{3}J_{HH}$ = 6.5 Hz, 1H, O–*C*H), 4.35 – 4.33 (m, 1H, *Cp*), 4.09 – 4.07 (m, 1H, *Cp*), 4.06 (s, 5H, *Cp*), 3.97 – 3.94 (s, 2H, *Cp*), 1.49 (d, $^{3}J_{HH}$ = 6.5 Hz, 3H, C*H*$_3$), 1.09 (s, 6H, *OBpin*), 1.08 (s, 6H, *OBpin*) ppm.

^{11}B NMR (128 MHz, 298 K, C_6D_6): δ = 22.5 (s) ppm.

^{13}C{^{1}H} NMR (101 MHz, 298 K, C_6D_6): δ = 92.13 (s, *Cp*), 82.11 (s, *OBpin*), 68.91 (s, *Cp*), 68.63 (s, *Cp*), 67.58 (two overlapping singlets, *Cp*), 67.16 (s, *Cp*), 65.73 (s, O–*C*H), 24.48 (s, *OBpin*), 24.39 (s, *OBpin*), 23.64 (s, C*H*$_3$).

Mass spectrometry (APCI): $C_{18}H_{25}B_1FeO_3$+Na ($[M+Na]^+$): calcd: 379.1142; found: 379.1120.

NMR conv.: 85%

Isolated Yield: 79%

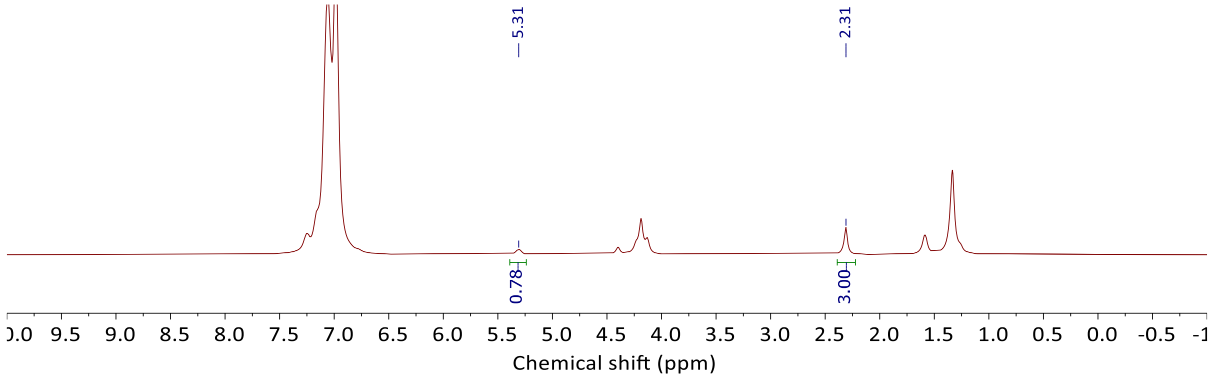

Figure S80. ^{1}H NMR spectrum (*o*DFB) of crude **19b**.

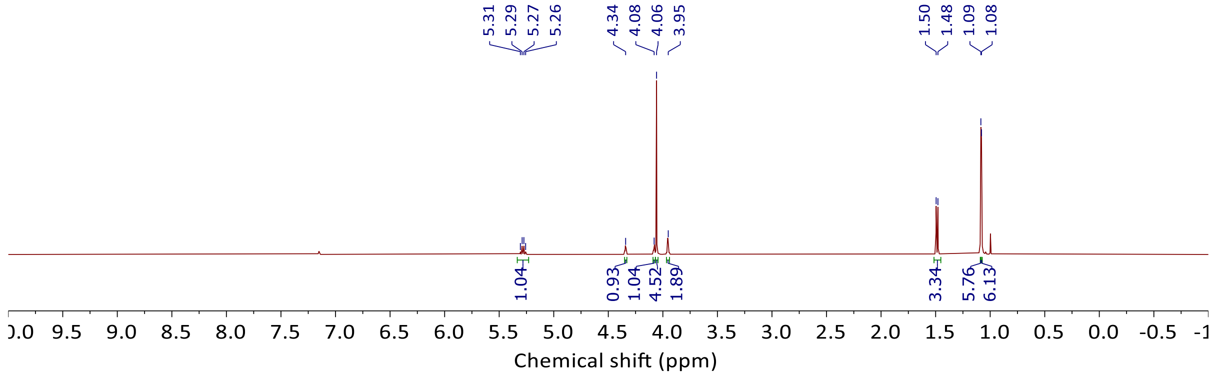

Figure S81. ^{1}H NMR spectrum (C_6D_6) of isolated **19b**.

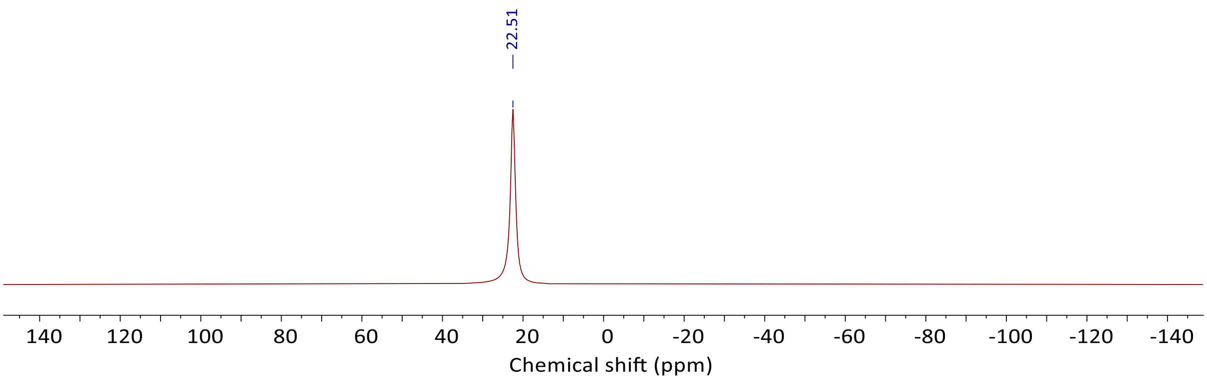

Figure S82. ^{11}B NMR spectrum (C_6D_6) of isolated **19b**.

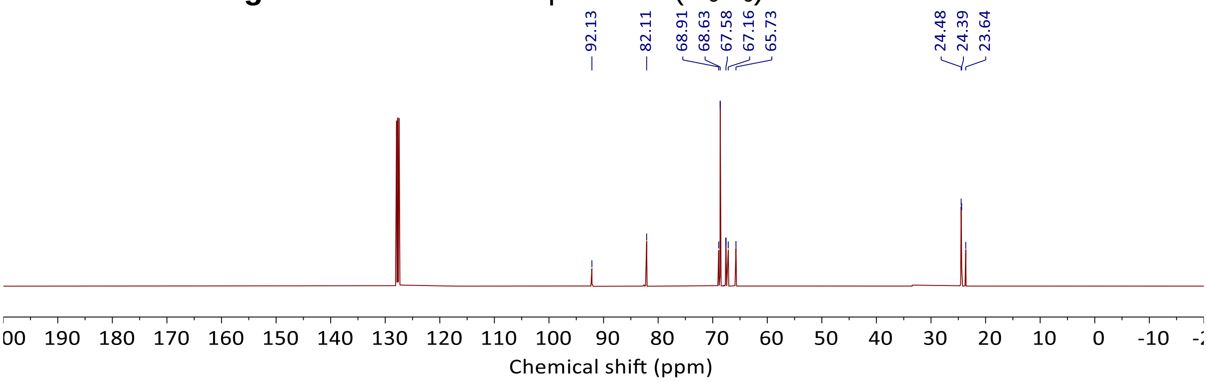

Figure S83. ^{13}C{^{1}H} NMR spectrum (C_6D_6) of isolated **19b**.

3.2.2.8. 2-(adamantan-2-yloxy)-4,4,5,5-tetramethyl-1,3,2-dioxaborolane

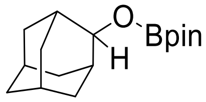

^{1}H NMR (400 MHz, 298 K, C_6D_6): δ = 4.47 (t, $^3J_{HH}$ = 3.5 Hz, 1H, O–*C*H), 2.37 (d, $^3J_{HH}$ = 12.4 Hz, 2H, *Ad*), 2.12 – 2.02 (m, 2H, *Ad*), 1.76 – 1.53 (m, 8H, *Ad*), 1.44 (d, $^3J_{HH}$ = 12.4 Hz, 2H, *Ad*), 1.07 (s, 12H, *OBpin*) ppm.

^{11}B NMR (128 MHz, 298 K, C_6D_6): δ = 22.38 (s) ppm.

^{13}C{^{1}H} NMR (101 MHz, 298 K, C_6D_6): δ = 81.84 (s, *OBpin*), 76.78 (s, O–*C*H), 37.52 (s, *Ad*), 36.29 (s, *Ad*), 34.24 (s, *Ad*), 31.16 (s, *Ad*), 27.58 (s, *Ad*), 27.10 (s, *Ad*), 24.36 (s, *OBpin*) ppm.

Mass spectrometry (APCI): $C_{16}H_{27}B_1O_3$+H ([M+H]$^+$): calcd: 279.2129; found: 279.2125.

NMR conv.: 99%

Isolated Yield: 92%

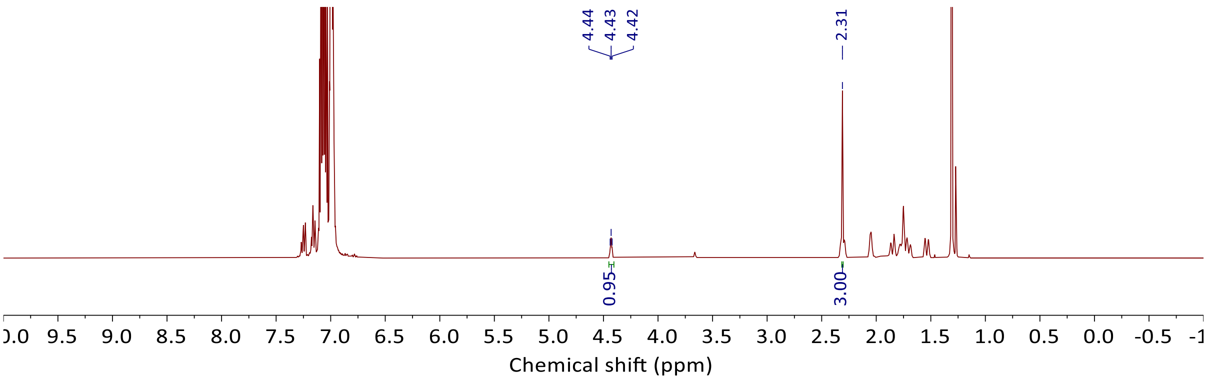

Figure S84. ^{1}H NMR spectrum (*o*DFB) of crude **20b**.

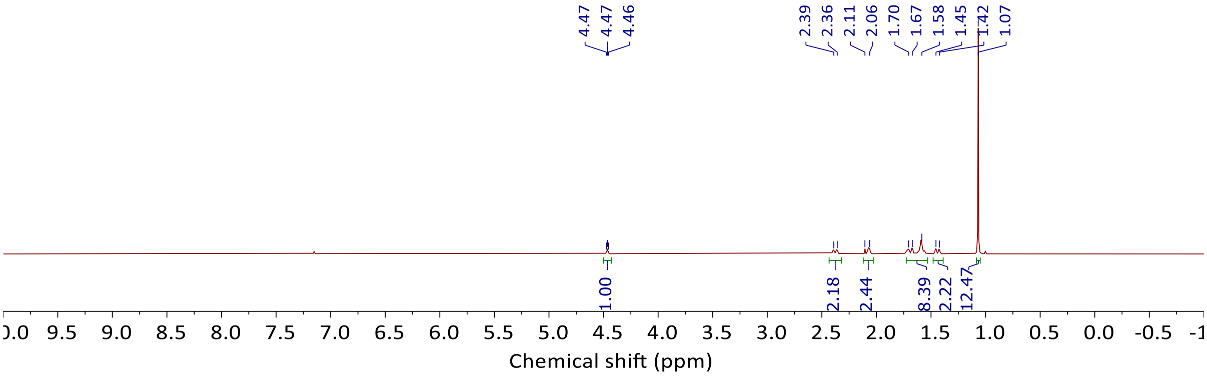

Figure S85. ^{1}H NMR spectrum (C_6D_6) of isolated **20b**.

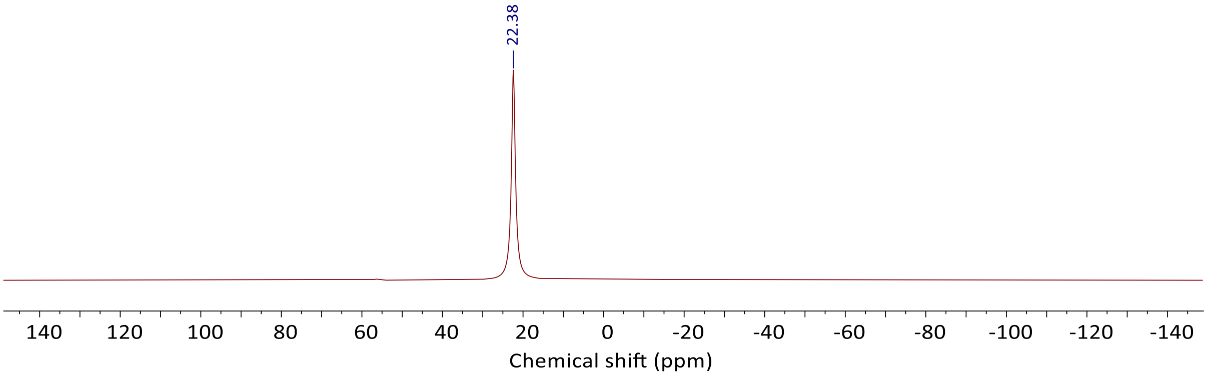

Figure S86. ^{11}B NMR spectrum (C_6D_6) of isolated **20b**.

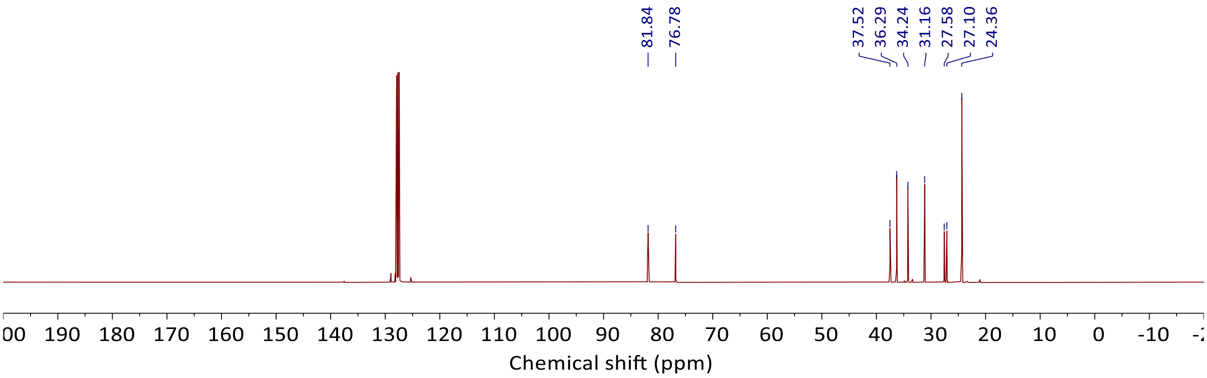

Figure S87. $^{13}C\{^{1}H\}$ NMR spectrum (C_6D_6) of isolated **20b**.

3.2.2.9. 2-(1-cyclobutylethoxy)-4,4,5,5-tetramethyl-1,3,2-dioxaborolane

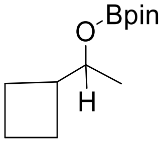

^{1}H NMR (400 MHz, 298 K, C_6D_6): δ = 4.27 (qd, $^{3}J_{HH}$ = 6.3 Hz, $^{3}J_{HH}$ = 6.3 Hz, 1H, O–*C*H), 2.35 – 2.21 (m, 1H), 2.08 – 1.64 (m, 6H, *cyclobutyl*), 1.10 (d, $^{3}J_{HH}$ = 6.2 Hz, 3H, C*H*$_3$), 1.08 (s, 12H, *OBpin*) ppm.

^{11}B NMR (400 MHz, 298 K, C_6D_6): δ = 22.5 (s) ppm.

^{13}C{^{1}H} NMR (101 MHz, 298 K, C_6D_6): δ = 81.84 (s, *OBpin*), 73.89 (s, O–*C*H), 42.15 (s, *cyclobutyl*), 24.36 (s, *cyclobutyl*), 24.28 (s, *CH$_3$*), 24.09 (s, *OBpin*), 24.04 (s, *OBpin*), 19.56 (s, *cyclobutyl*), 17.68 (s, *cyclobutyl*) ppm.

Mass spectrometry (ESI): $C_{12}H_{23}B_1O_3$+K ([M+K]$^+$): calcd: 265.1377; found: 265.1435.

NMR conv.: 99%

Isolated Yield: 91%

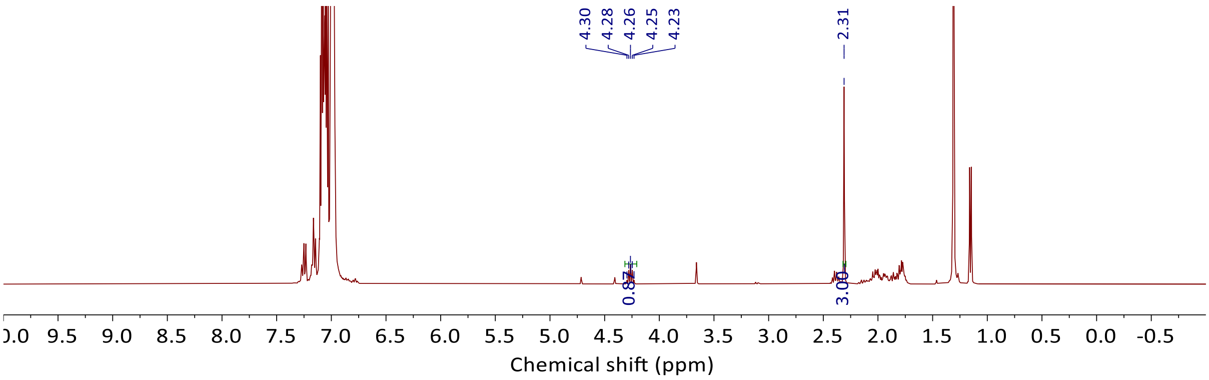

Figure S88. ^{1}H NMR spectrum (*o*DFB) of crude **21b**.

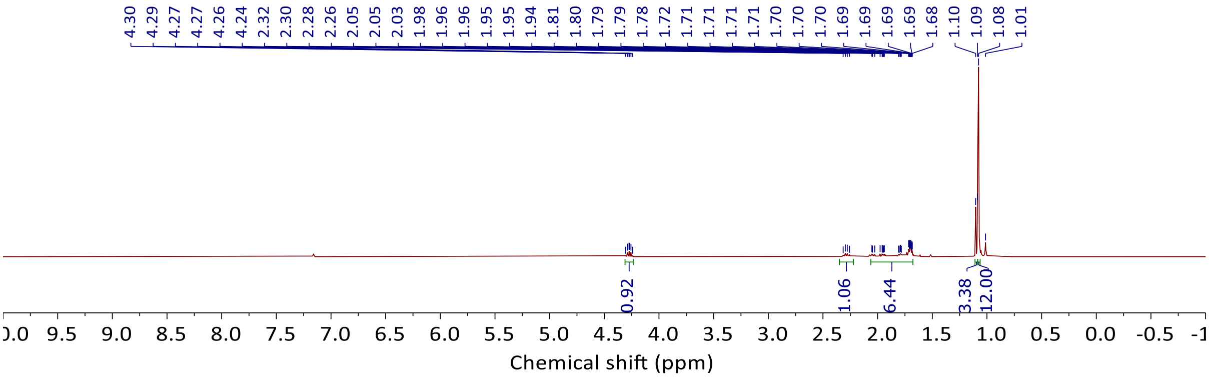

Figure S89. ^{1}H NMR spectrum (C_6D_6) of isolated **21b**.

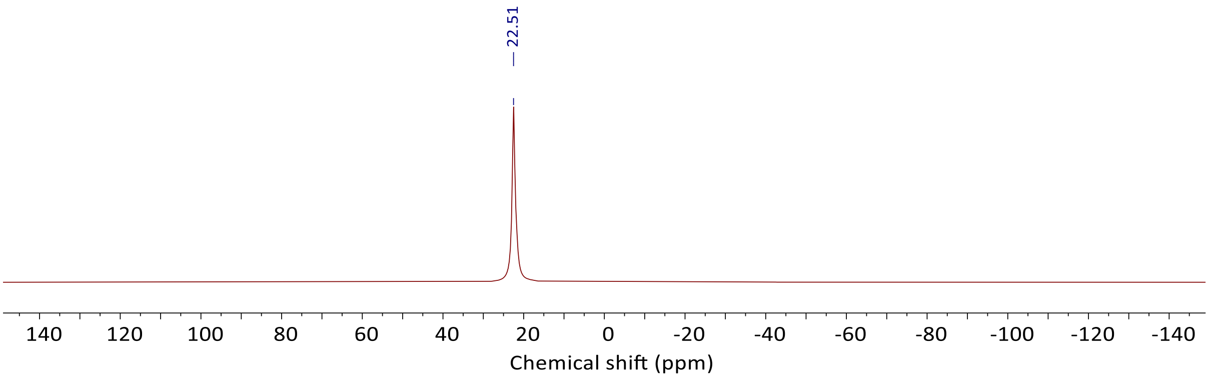

Figure S90. ^{11}B NMR spectrum (C_6D_6) of isolated **21b**.

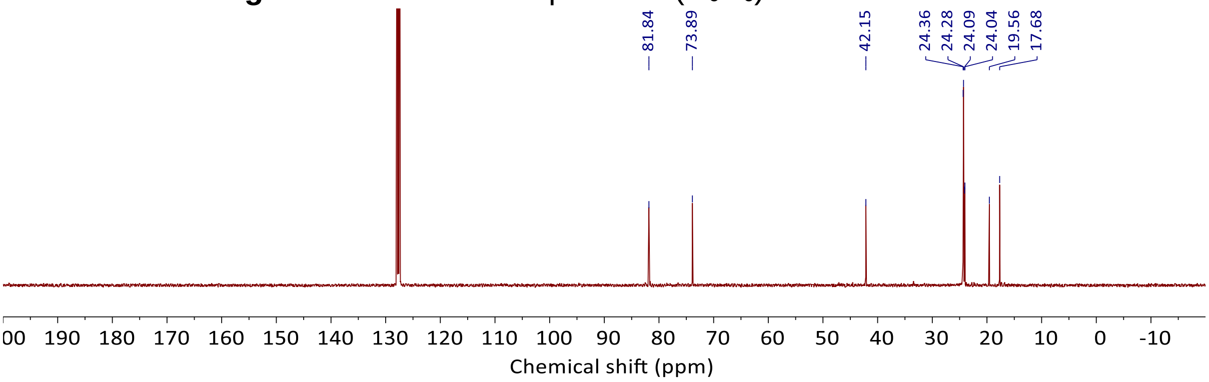

Figure S91. ^{13}C{^{1}H} NMR spectrum (C_6D_6) of isolated **21b**.

3.2.2.10. 2-[(but-3-yn-2-yl)ethoxy]-4,4,5,5-tetramethyl-1,3,2-dioxaborolane

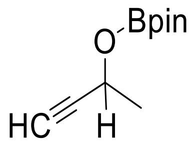

^{1}H NMR (400 MHz, 298 K, Reaction mixture): δ = 5.01 (qd, $^3J_{HH}$ = 6.6, $^4J_{HH}$ = 2.1 Hz, 1H, O–C*H*), 2.43 (d, $^4J_{HH}$ = 2.1 Hz, 1H, C≡C–*H*), 1.52 (d, $^3J_{HH}$ = 6.6 Hz, 3H, C*H*$_3$), 1.27 (s, 6H, *OBpin*), 1.26 (s, 6H, *OBpin*) ppm.

^{11}B NMR (128 MHz, 298 K, Reaction mixture): δ = 22.3 (s) ppm.

Mass spectrometry after hydrolysis (ESI): $C_4H_6O_1$+H ([M+H]$^+$): calcd: 71.0491; found: 71.0496.

NMR conv.: 23%

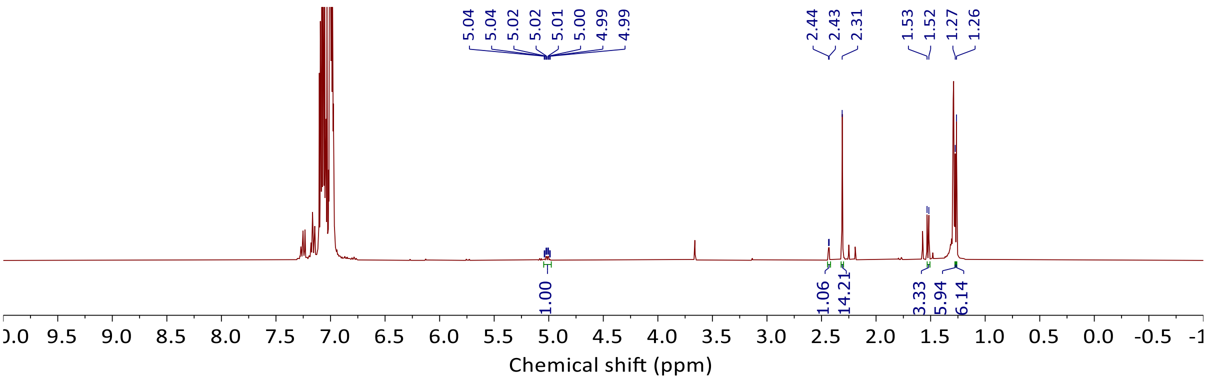

Figure S92. ^{1}H NMR spectrum (*o*DFB) of crude **22b**.

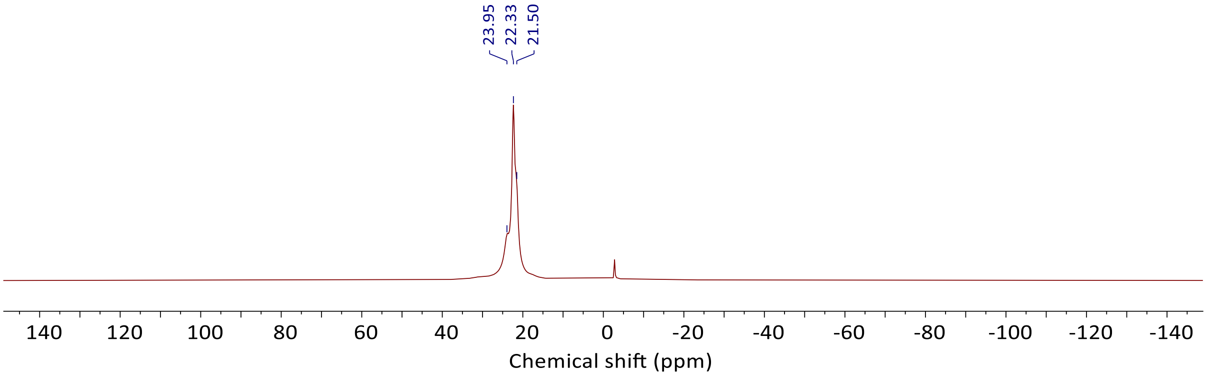

Figure S93. ^{11}B NMR spectrum (*o*DFB) of crude **22b**.

3.2.2.11. 2-(cyclohex-2-en-1-yloxy)-4,4,5,5-tetramethyl-1,3,2-dioxaborolane

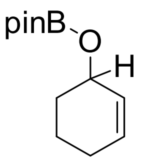

^{1}H NMR (400 MHz, 298 K, Reaction mixture): δ = 5.94 – 5.71 (m, 1H, OCH–C*H*=CH), 5.43 – 5.30 (m, 3H, O–C*H*), 1.30 (s, 12H, *OBpin*) ppm.

^{11}B NMR (128 MHz, 298 K, Reaction mixture): δ = 21.6 (s) ppm.

Mass spectrometry after hydrolysis (ESI): $C_6H_{10}O_1$+H ($[M+H]^+$): calcd: 99.0810; found: 99.0769.

NMR conv.: 33%

Note: Due to the poor yield of the reaction there is significant overlap preventing identification of all resonances corresponding to the hydroborated product.

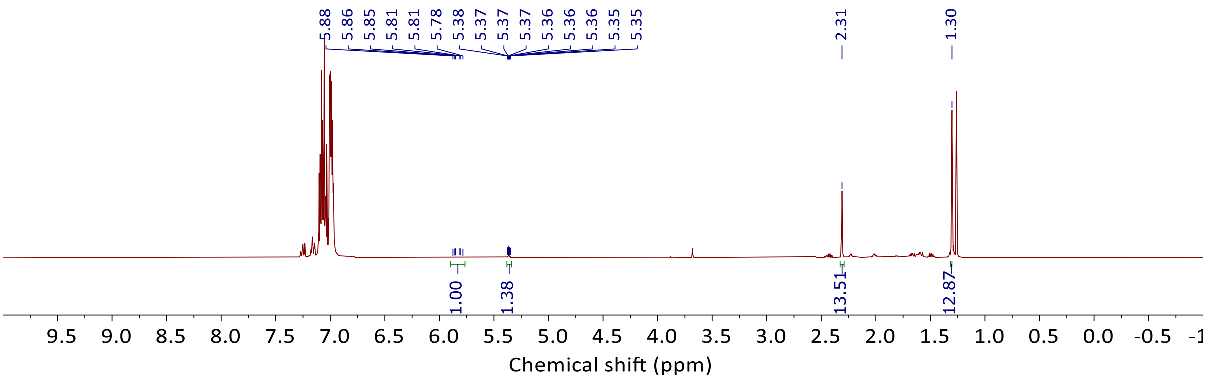

Figure S94. ^{1}H NMR spectrum (*o*DFB) of crude **23b**.

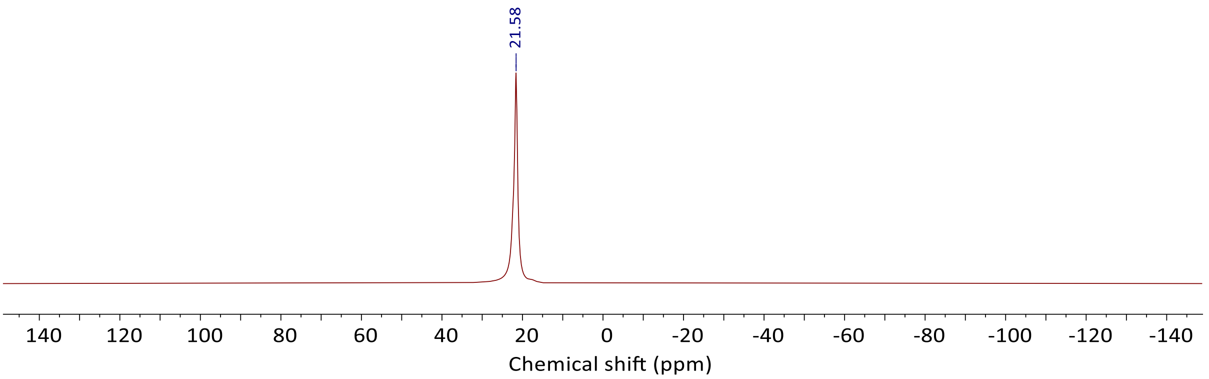

Figure S95. ^{11}B NMR spectrum (*o*DFB) of crude **23b**.

3.3. Chemoselectivity Hydroboration of Aldehydes Versus Ketones

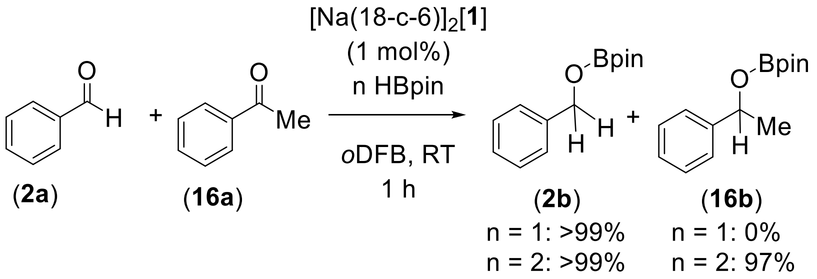

To a J Young NMR tube a solution of [Na(18-c-6)]$_2$[**1**] (2 mg, 2.19 µmol, 0.01 eq.), *o*DFB (0.5 mL), acetophenone (26 µL, 0.219 mmol, 1.0 eq.), benzaldehyde (22 µL, 0.219 mmol, 1.0 eq.), toluene (25 µL, 0.235 mmol) and pinacolborane (28 mg, 32 µL, 0.219 mmol, 1.0 eq.) was added. The reaction was monitored by ^{1}H NMR, ^{11}B NMR and $^{11}B\{^{1}H\}$ NMR. Crude NMR conv. was determined by integration of the ^{1}H NMR spectrum using the toluene as internal standard (^{1}H δ = 2.31 ppm). After complete consumption of pinacolborane another equivalent of pinacolborane (28 mg, 32 µL, 0.219 mmol, 1.0 eq.) was added. Again, the reaction was monitored by ^{1}H NMR, ^{11}B

NMR and $^{11}B\{^{1}H\}$ NMR. NMR conv. was determined by integration of the crude ^{1}H NMR spectrum using the toluene as internal standard. Addition of 1.0 eq. pinacolborane resulted in exclusive hydroboration of **2a** and addition of a second eq. pinacolborane resulted in hydroboration of the **16a**.

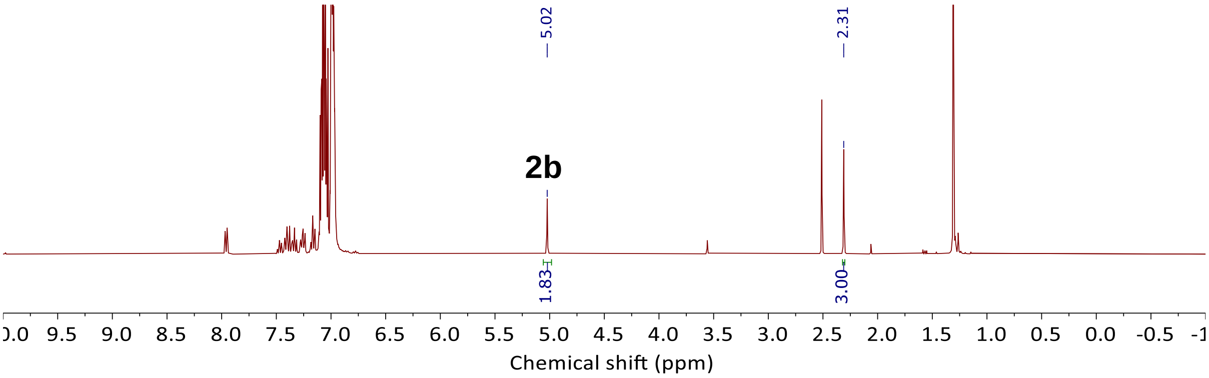

Figure S96. ^{1}H NMR spectrum (*o*DFB) of reaction mixture after addition of 1.0 eq. HBpin.

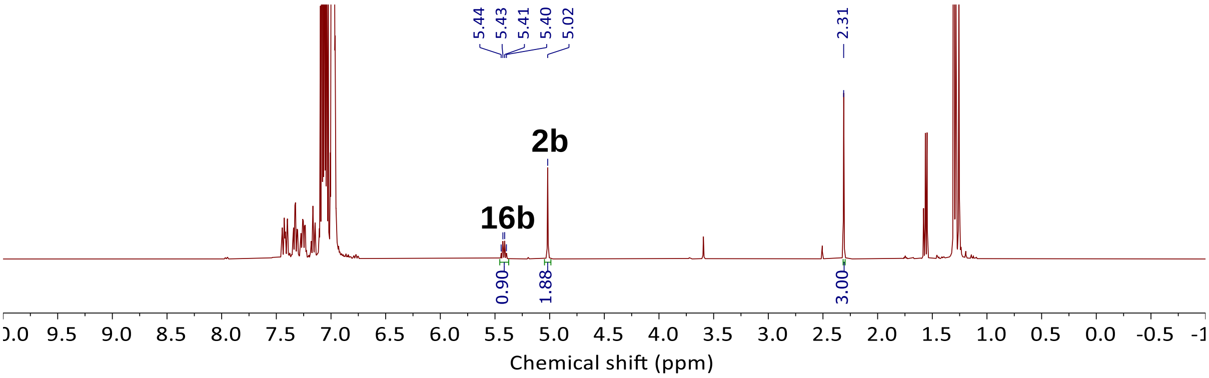

Figure S97. ^{1}H NMR spectrum (*o*DFB) of reaction mixture after addition of 2.0 eq. HBpin.

4. Catalytic Hydroboration of Carbodiimides and Isocyanates

4.1. General Procedure for Hydroboration of Carbodiimides and Isocyanates

To a J Young NMR tube a solution of $[Na(18\text{-}c\text{-}6)]_2$[**1**] (2 mg, 2.19 µmol, 0.01 eq.), pinacolborane (84 mg, 96 µL, 0.66 mmol, 3.0 eq.), *o*DFB (0.5 mL), heteroallene (0.22 mmol, 1.0 eq.) and toluene (25 µL, 0.24 mmol) was added. The reaction was allowed to proceed for 1 - 7 days. The reaction was monitored by ^{1}H NMR, ^{11}B NMR and $^{11}B\{^{1}H\}$ NMR. NMR conv. was determined by integration of the crude ^{1}H NMR spectrum using the toluene as internal standard (^{1}H δ = 2.31 ppm). Products were not isolated. Resonances arising from new C–H bonds have been marked below and were used to calculate yields. NMR assignment for **24b**, **25b**, **26b**, **26d**, **27b**, and **27d** match literature values.[5-7] NMR assignment for **26c**, and **27c** match closely related analogues compounds reported in literature.[8]

4.1.1. Hydroboration of Diisopropyl Carbodiimide

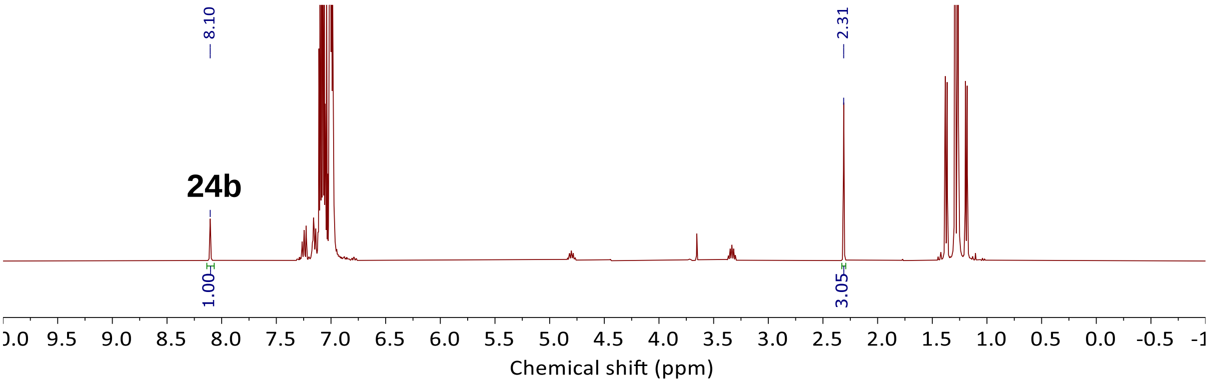

Figure S98. ^{1}H NMR spectrum (*o*DFB) of hydroboration of **24a**.

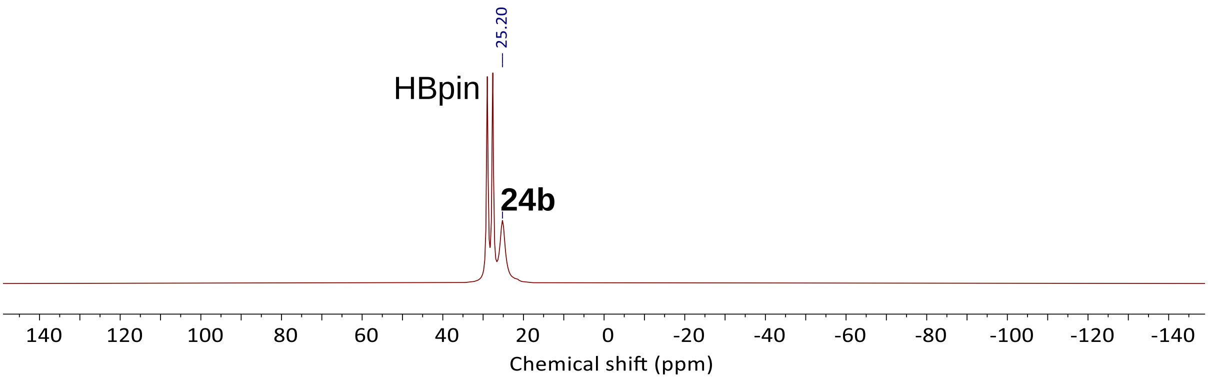

Figure S99. ^{11}B NMR spectrum (*o*DFB) of hydroboration of **24a**.

4.1.2. Hydroboration of Dicyclohexyl Carbodiimide

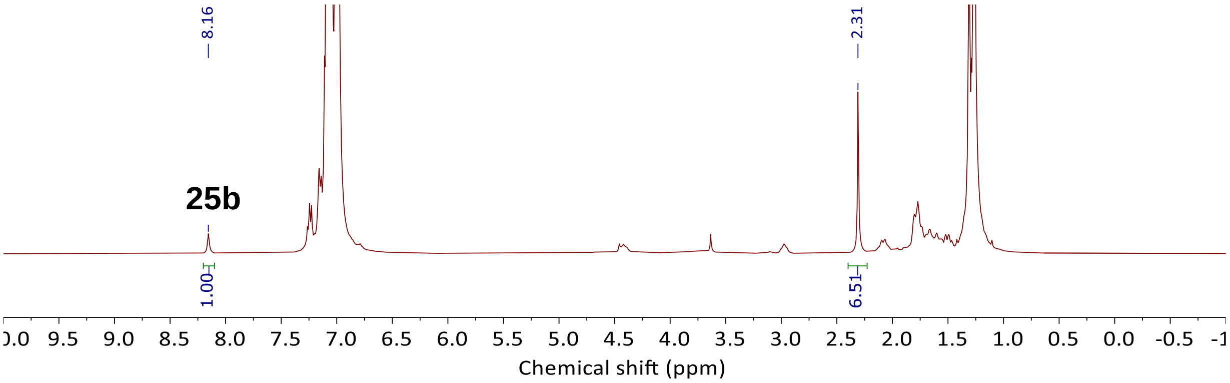

Figure S100. ^{1}H NMR spectrum (*o*DFB) of hydroboration of **25a**.

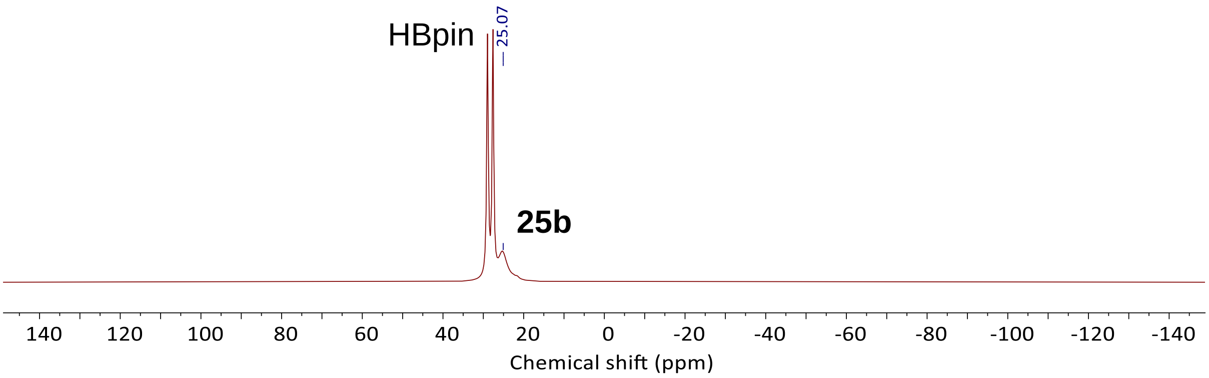

Figure S101. ^{11}B NMR spectrum (*o*DFB) of hydroboration of **25a**.

4.1.3. Hydroboration of Phenyl Isocyanate

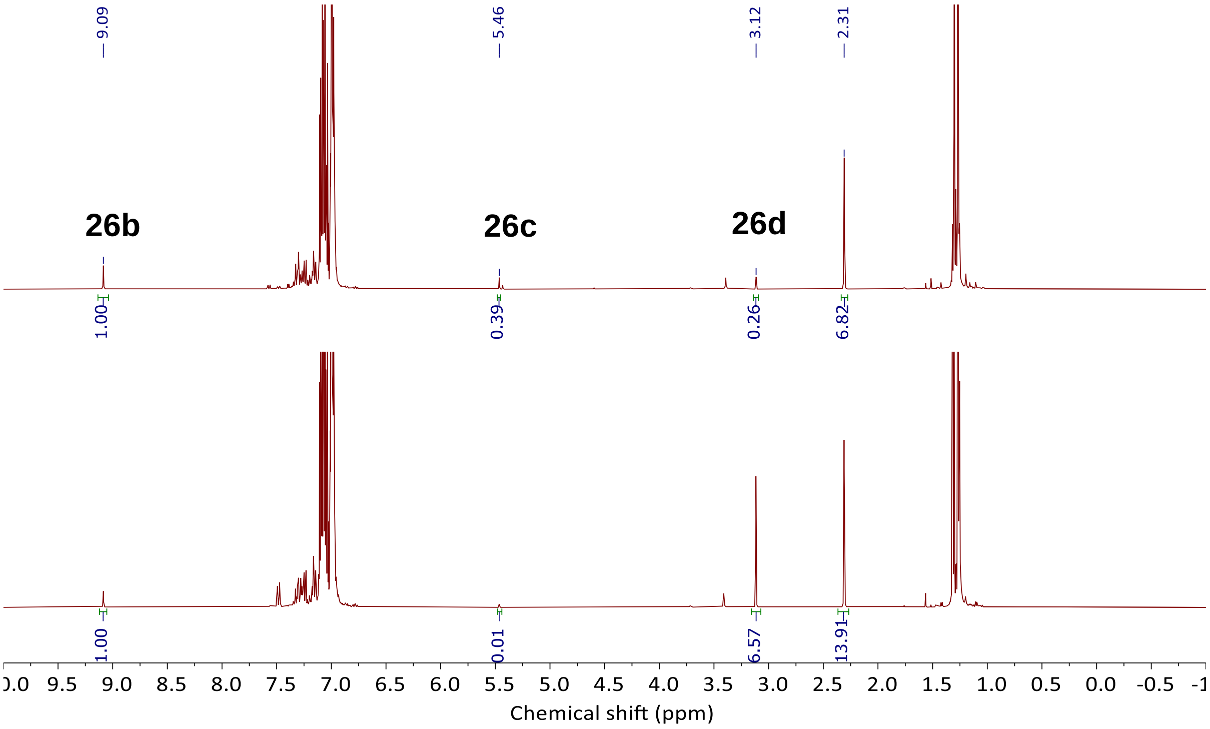

Figure S102. ^{1}H NMR spectrum (*o*DFB) of hydroboration of **26a**. Top spectrum recorded after 1 day; Bottom spectrum recoded after 7 days.

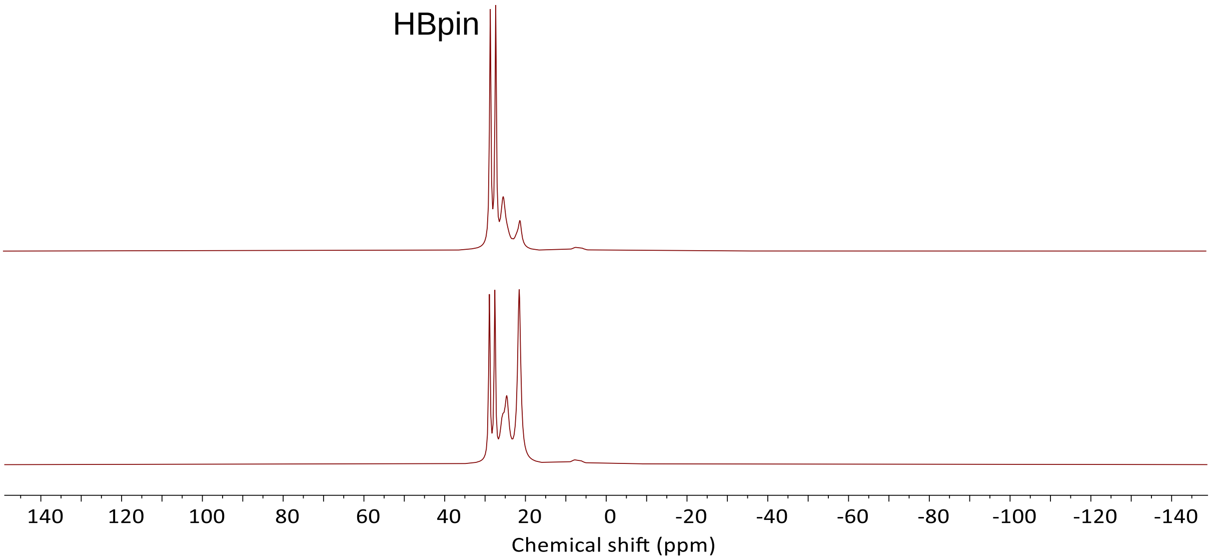

Figure S103. ^{11}B NMR spectrum (*o*DFB) of hydroboration of **26a**. Top spectrum recorded after 1 day; Bottom spectrum recoded after 7 days.

4.1.4. Hydroboration of Cyclohexyl Isocyanate

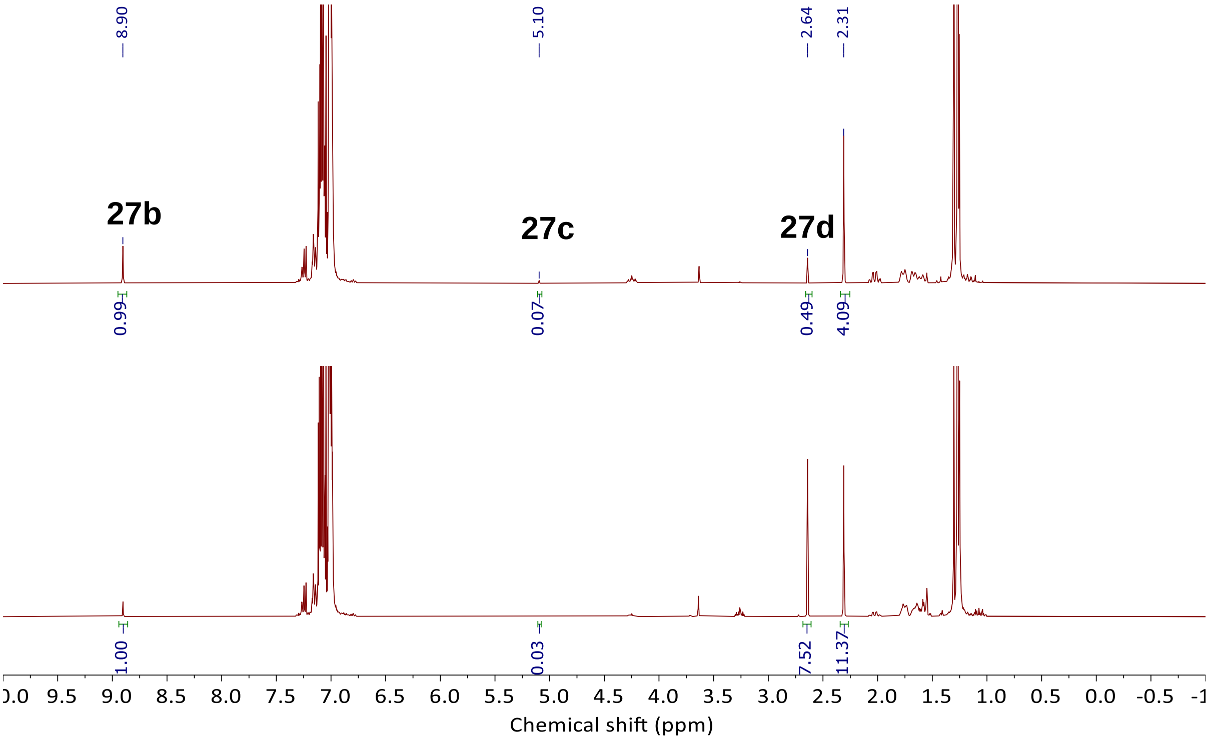

Figure S104. ^{1}H NMR spectrum (*o*DFB) of hydroboration of **27a**. Top spectrum recorded after 1 day; Bottom spectrum recoded after 7 days.

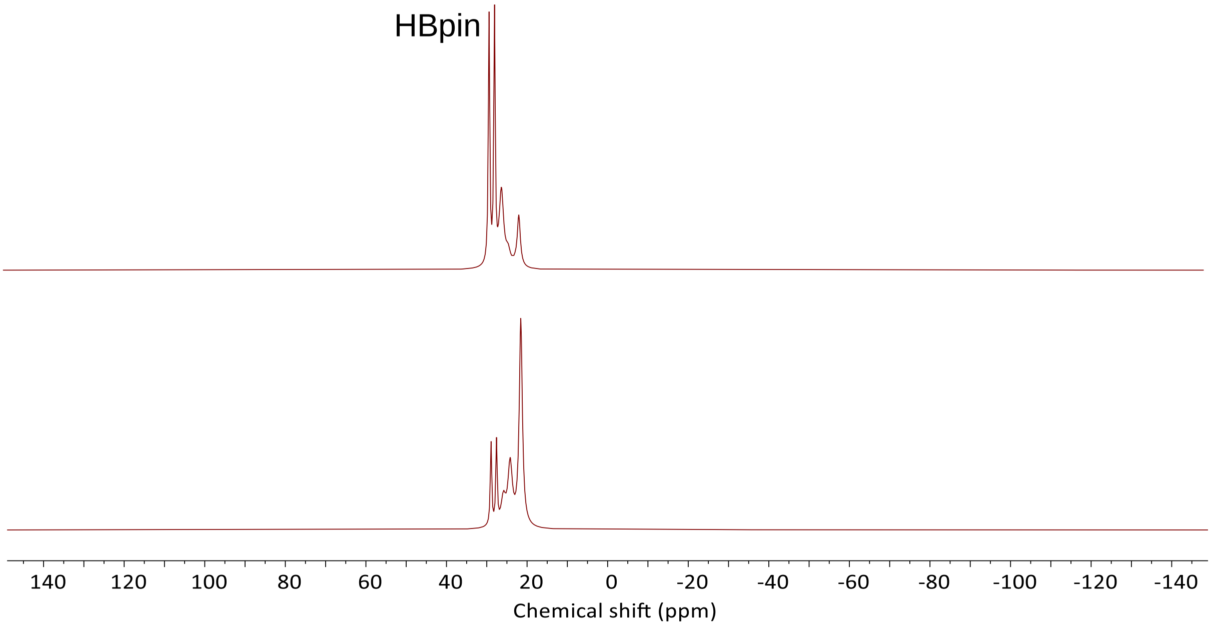

Figure S105. ^{11}B NMR spectrum (*o*DFB) of hydroboration of **27a**. Top spectrum recorded after 1 day; Bottom spectrum recoded after 7 days.

5. Catalytic Hydroboration of Carbon Dioxide

5.1. Solvent and Reductant Screening

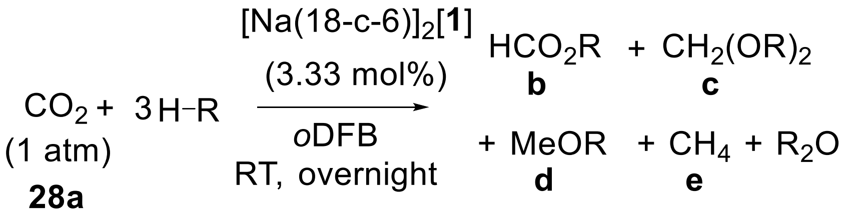

R = $SiEt_3$, $SiPh_3$, Bpin, Bcat, BBN, $BH_2(SMe_2)$

To a J Young NMR tube a solution of $[Na(18\text{-}c\text{-}6)]_2$[**1**] (9.0 mg, 9.9 µmol, 0.033 eq.), solvent (0.6 mL) and reductant (0.30 mmol, 1.0 eq. ‘H-R’) was added. The reaction mixture was degassed and the headspace was refilled with CO_2 (1 atm). The reaction was monitored by ^1H, ^{11}B and $^{11}B\{^1H\}$ NMR. After 18 hours, toluene (25 µL, 0.24 mmol) was added as an internal standard. NMR conv. was determined by integration of the crude ^1H NMR spectrum using the toluene as internal standard (^1H δ = 2.31 ppm).

Table S5. Solvent and reductant screening hydroboration CO_2

Entry	Borane	Solvent	HCO_2R Conv. (%)[a]	$CH_2(OR)_2$ Conv. (%)[a]	MeOR Conv. (%)[a]	CH_4 Conv. (%)[a, b]	Overall Conv. (%)[a]
1	HBpin	THF	24	33	5	5	67
2	HBpin	*o*DFB	3	1	31	10	45
3	HBpin	Toluene	0	0	0	0	0
4	HBcat	THF	0	0	2	0	2
5	HBcat	*o*DFB	0	0	0	0	0
6	HBcat	Toluene	0	0	0	0	0
7	$BH_3{\cdot}SMe_2$	THF	0	0	0	0	0
8	$BH_3{\cdot}SMe_2$	*o*DFB	0	0	0	0	0
9	$BH_3{\cdot}SMe_2$	Toluene	0	0	0	0	0
10	HBBN dimer	THF	0	5	52	0	57
11	HBBN dimer	*o*DFB	11	0	84	0	95
12	HBBN dimer	Toluene	0	0	0	0	0
13	Et_3SiH	*o*DFB	0	0	0	0	0
14	Ph_3SiH	*o*DFB	0	0	0	0	0

[a] Determined by ^1H NMR spectroscopy, based on C–H bond formation. [b] Due to the gaseous nature of CH_4 a higher conversion is expected than reported.

5.2. Catalyst Loading

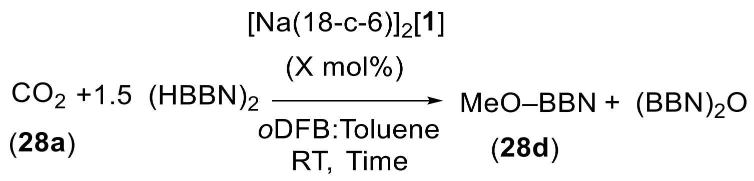

To a J Young NMR tube a solution of [Na(18-c-6)]$_2$[**1**] (specified loading), C_6Me_6 (6.5 mg, 0.04 mmol), *o*DFB:toluene mixture (0.6 mL, 2:1) (0.6 mL) and HBBN dimer (0.5 eq., 36 mg, 0.15 mmol) was added. The reaction mixture was degassed and the headspace was refilled with CO_2 (1 atm). The reaction was monitored by ^1H, ^{11}B and $^{11}B\{^1H\}$ NMR. The NMR conv. was calculated by integration of the crude ^1H NMR spectrum using the C_6Me_6 as an internal standard (^1H δ = 2.20 ppm). NMR spectra of lowest loading is presented below. All HBBN dimer was fully consumed. NMR assignment for **28d** match literature values.[9, 10]

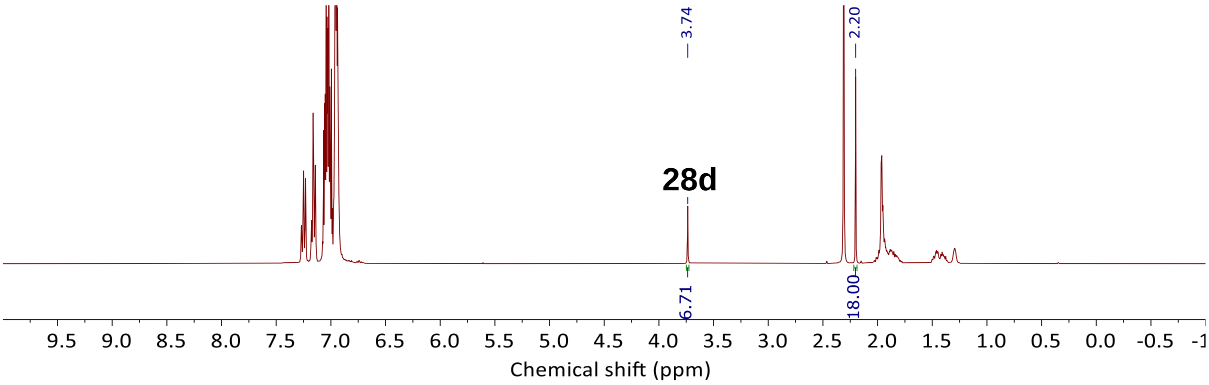

Figure S106. ^1H NMR spectrum (reaction mixture) of crude **28d**.

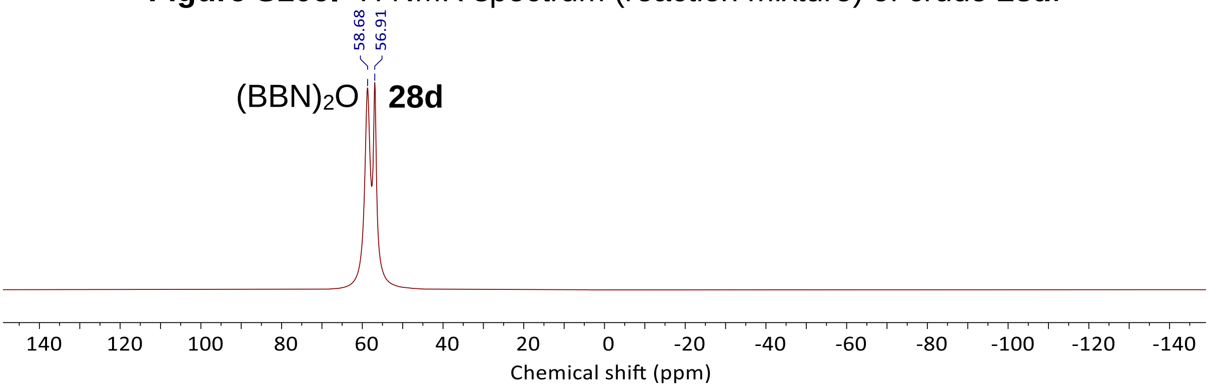

Figure S107. ^{11}B NMR spectrum (reaction mixture) of crude **28d**.

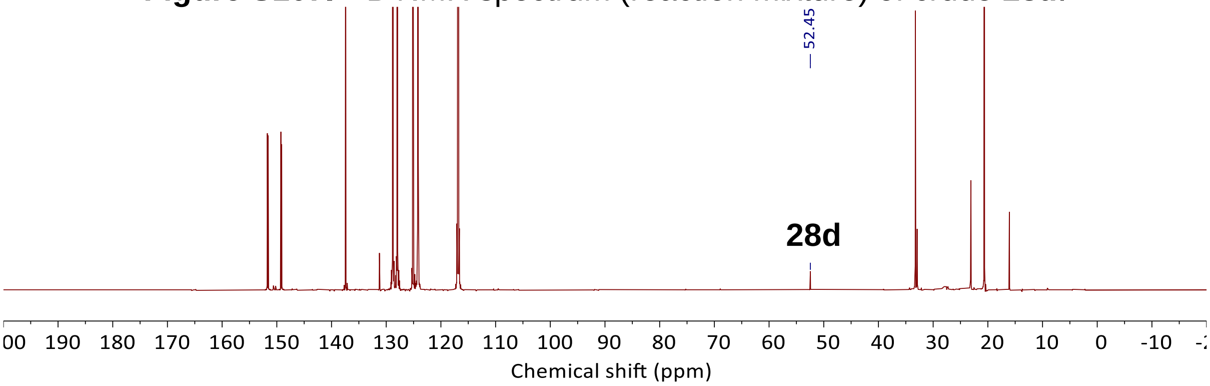

Figure S108. $^{13}C\{^1H\}$ NMR spectrum (reaction mixture) of crude **28d**.

5.3. Catalyst Control Reactions

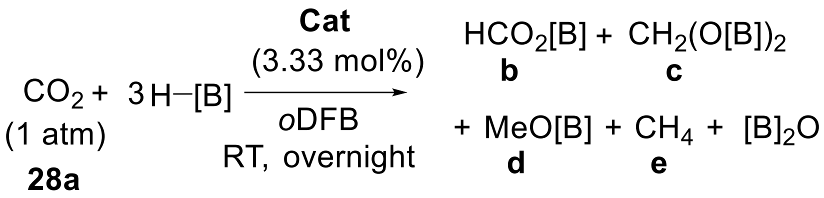

Same general procedure as above in Section 5.3.

Table S6. Catalyst Control Reactions

Entry	Catalyst	Solvent	HCOO[B] Conv. (%)[a]	[B]OCH$_2$O[B] Conv. (%)[a]	MeO[B] Conv. (%)[a]	CH_4 Conv. (%)[a]	Overall Conv. (%)[a]
1	-	*o*DFB	0	0	0	0	0
2	[Na(DME)]$_3$P$_7$	*o*DFB	0	0	0	0	0
3	[Na(DME)]$_3$P$_7$	*o*DFB/Pyr	0	0	0	0	0
4	K_3P_7	*o*DFB	0	0	0	0	0
5	K_3P_7 + 18-c-6	*o*DFB	0	0	0	0	0
6	$(Me_3Si)_3P_7$[b]	*o*DFB	0	0	0	0	0

[a] Determined by ^{1}H NMR spectroscopy, based on C–H bond formation. [b] Synthesised according to literature procedure.[1]

5.4. Isotopic Labelled Studies

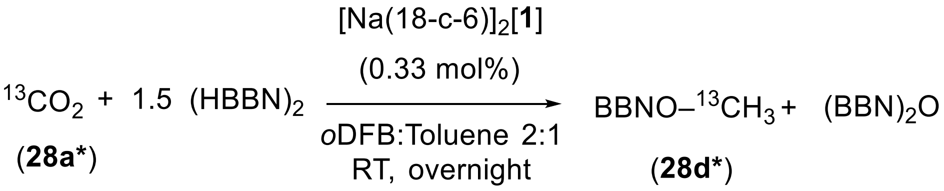

To a J Young NMR tube a solution of $[Na(18\text{-}c\text{-}6)]_2$[**1**] (0.9 mg, 0.99 µmol, 0.0033 eq.) in *o*DFB:toluene (0.6 mL, 2:1), C_6Me_6 (6.2 mg, 0.038 mmol) and HBBN dimer (0.5 eq., 36 mg, 0.15 mmol) was added. The reaction mixture was degassed and the headspace was refilled with ^{13}C labelled CO_2 (1 atm). The reaction was monitored by ^{1}H, ^{11}B and $^{11}B\{^{1}H\}$ NMR. The NMR conv. was calculated by integration of the crude ^{1}H NMR spectrum using the C_6Me_6 as an internal standard (^{1}H δ = 2.20 ppm). NMR spectra of lowest loading is presented below. All HBBN dimer was fully consumed.

^{1}H NMR (400 MHz, 298 K, Reaction mixture): δ = 3.71 (d, $^{1}J_{HC}$ = 142.7 Hz, 3H, $O^{13}CH_3$), 2.03 – 1.26 (m, 42H, MeOBBN and $(BBN)_2O$) ppm.
^{11}B NMR (128 MHz, 298 K, Reaction mixture): δ = 58.8 (s, $(BBN)_2O$), 56.9 (s, MeOBBN) ppm.
^{13}C{^{1}H} NMR (101 MHz, 298 K, Reaction mixture): δ = 52.07 (s, $O^{13}CH_3$) ppm.
NMR conv.: 99%

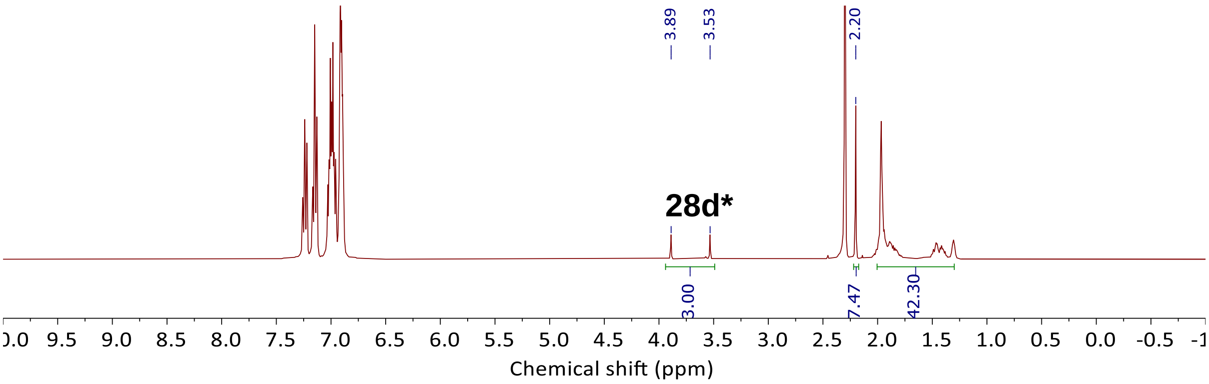

Figure S109. ^{1}H NMR spectrum (reaction mixture) of crude **28d***.

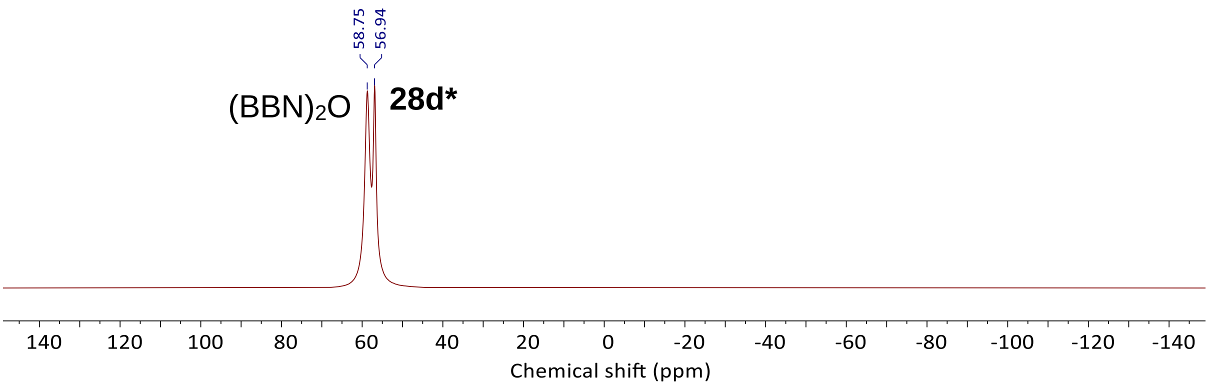

Figure S110. ^{11}B NMR spectrum (reaction mixture) of crude **28d***.

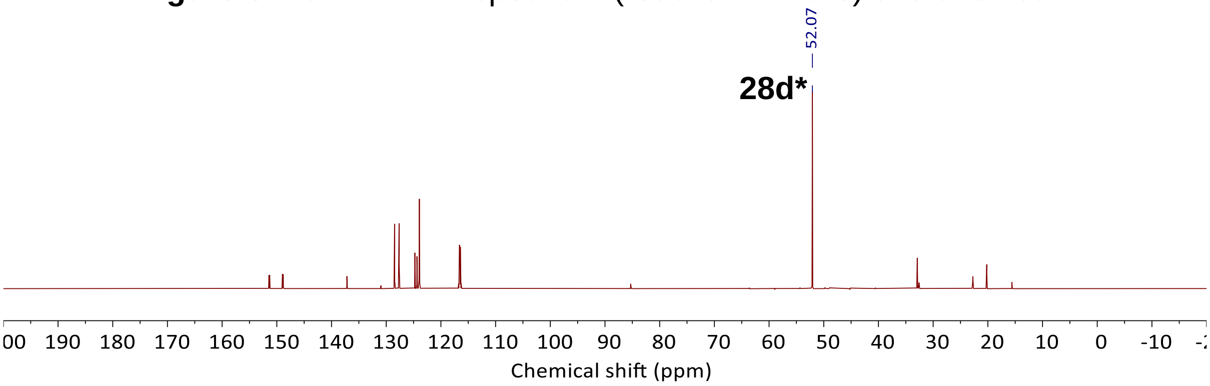

Figure S111. $^{13}C\{^1H\}$ NMR spectrum (reaction mixture) of crude **28d***.

5.5. Tracked Catalytic Hydroboration of CO_2

5.5.1. Tracked Reaction at 25 °C

To a J Young NMR tube a solution of $[Na(18\text{-}c\text{-}6)]_2$[**1**] (0.9 mg, 0.99 µmol, 0.0033 eq.) in *o*DFB:toluene (0.6 mL, 2:1), C_6Me_6 (6.2 mg, 0.038 mmol) and HBBN dimer (0.5 eq., 36 mg, 0.15 mmol) was added. The reaction mixture was degassed and the headspace was refilled with CO_2 (1 atm). The NMR tube loaded into the NMR spectrometer within 1 min. The NMR spectrometer was set to 25 °C. The reaction was monitored by ^1H NMR. The NMR conv. was calculated by integration of the crude ^1H NMR spectrum using the C_6Me_6 as an internal standard (^1H δ = 2.20 ppm). TON: 300, TOF h^{-1}: 30. All HBBN dimer was fully consumed. NMR assignment for **28c** match literature values.[9, 10]

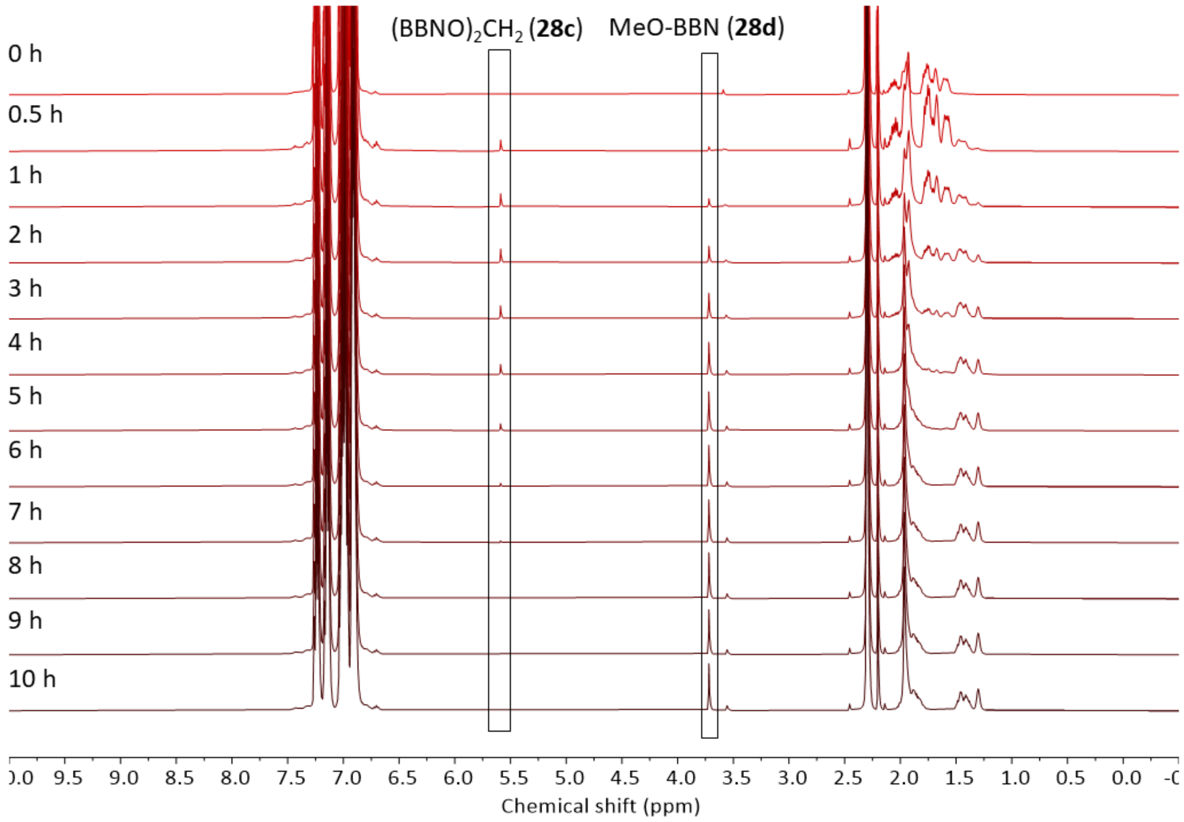

Figure S112. Stacked ^{1}H NMR spectrum (reaction mixture) of tracked hydroboration of CO_2 at 25 °C.

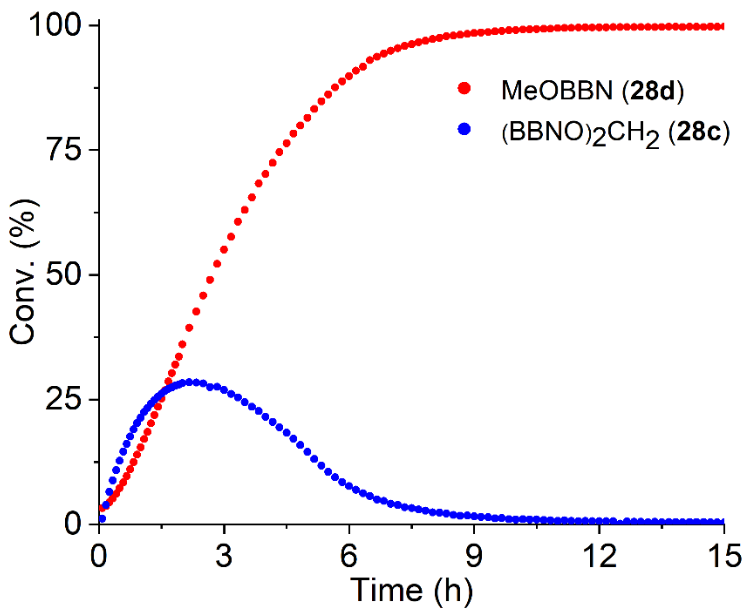

Figure S113. Tracked conversion hydroboration of CO_2 at 25 °C.

5.5.2. Tracked Reaction at 50 °C

Same procedure as shown above in section 5.4.1. except the NMR spectrometer was pre-set to 50 °C. All HBBN dimer was fully consumed. The NMR conv. was calculated by integration of the crude ^{1}H NMR spectrum using the C_6Me_6 (10 mg, 0.062 mmol) as an internal standard (^{1}H δ = 2.20 ppm). TON: 300, TOF h^{-1}: 300.

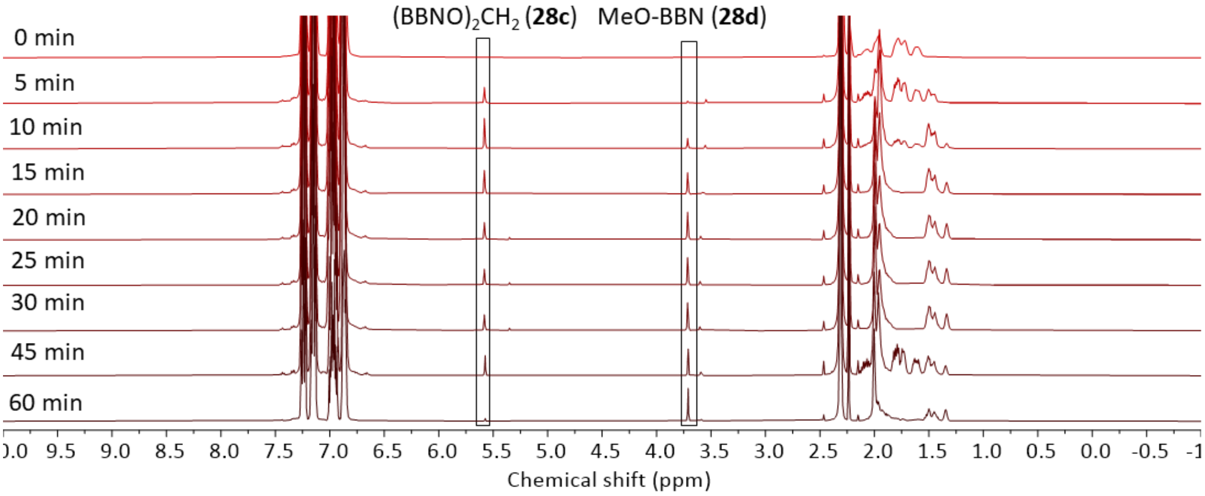

Figure S114. Stacked ^{1}H NMR spectrum (reaction mixture) of tracked hydroboration of CO_2 at 50 °C.

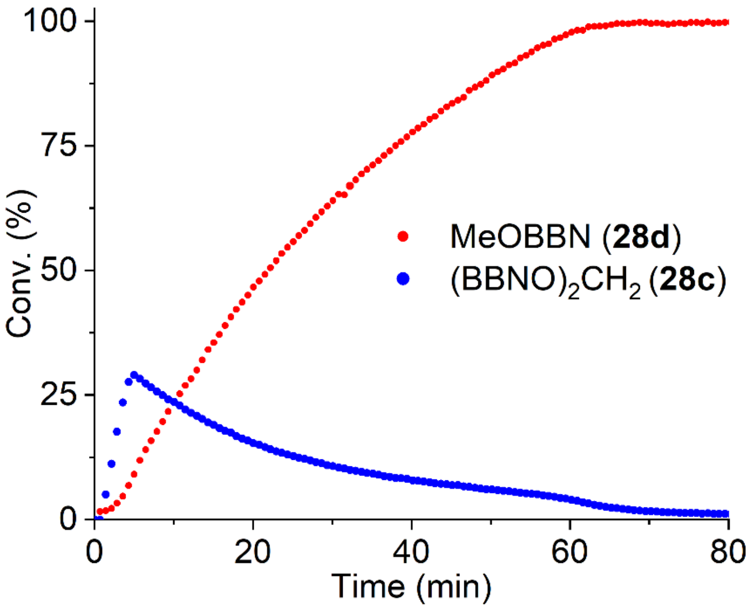

Figure S115. Tracked conversion hydroboration of CO_2 at 50 °C.

5.6. Catalyst Recycling

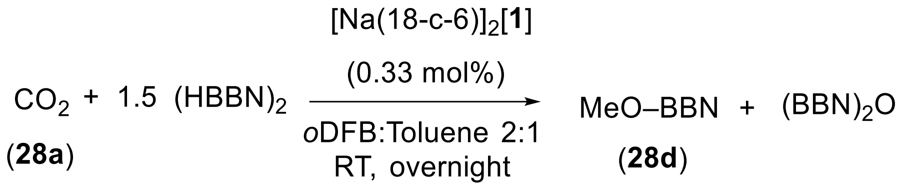

To a J Young NMR tube a solution of $[Na(18\text{-}c\text{-}6)]_2$[**1**] (0.9 mg, 0.99 µmol, 0.0033) in *o*DFB:toluene (0.6 mL, 2:1), C_6Me_6 (6.2 mg, 0.038 mmol) and HBBN dimer (36 mg, 0.15 mmol, 0.5 eq.) was added. The reaction mixture was degassed and the headspace was refilled with CO_2 (1 atm). The reaction was monitored by ^{1}H, ^{11}B and ^{11}B{^{1}H} NMR. The NMR conv. was calculated by integration of the crude ^{1}H NMR spectrum using the C_6Me_6 as an internal standard (^{1}H δ = 2.20 ppm). The tube was reloaded with HBBN dimer and subsequently degassed and the headspace refilled with CO_2 (1 atm). This process was repeated 7 times. No catalyst decomposition was observed by ^{1}H and ^{11}B NMR spectroscopy. Effective TON: 2067.

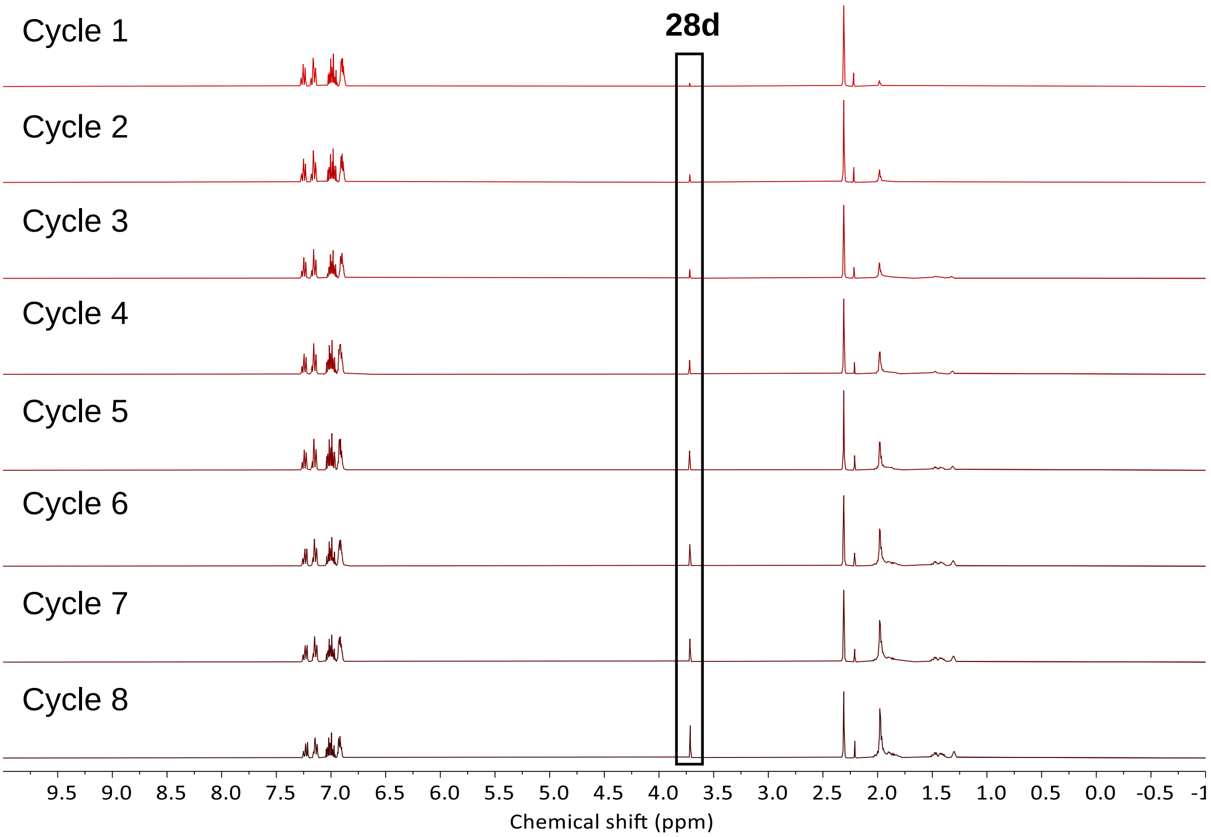

Figure S116. Stacked ^{1}H NMR spectrum (reaction mixture) living catalysis.

5.7. Hydrolysis Methoxyborane (28d) to Methanol

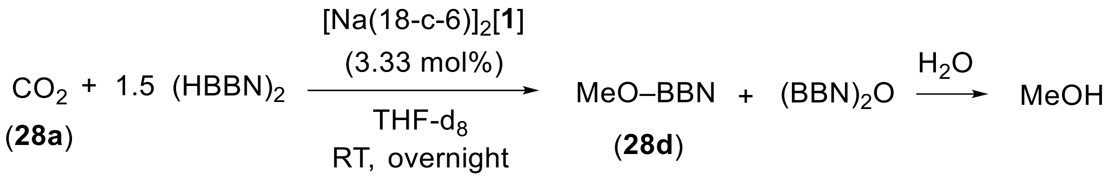

The reduction of CO_2 was performed as described above in section 5.2. After reaction completion was observed by ^{1}H, ^{11}B and ^{13}C{^{1}H} NMR spectroscopy, 50 µL H_2O was added. The reaction mixture was agitated for 30 min and then investigated by ^{1}H, ^{11}B and ^{13}C{^{1}H} NMR spectroscopy.

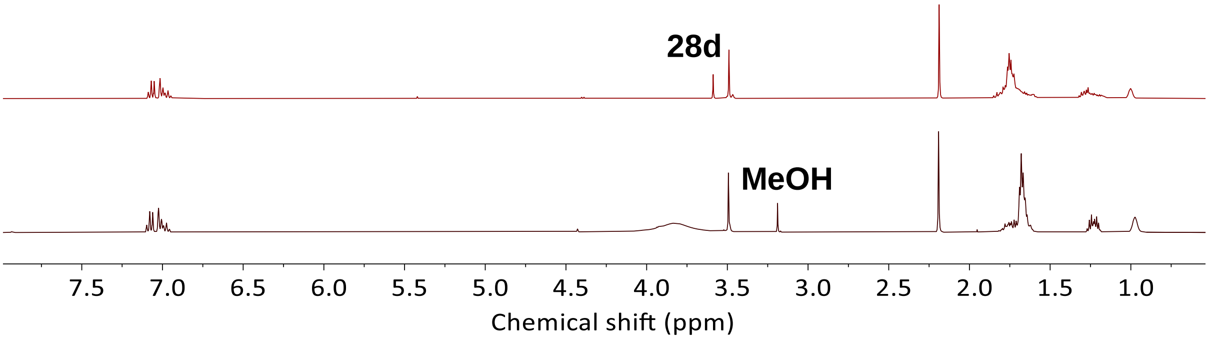

Figure S117. ^{1}H NMR spectrum (reaction mixture) hydrolysis of **28d**. Top: before addition of H_2O. Bottom: after addition of H_2O.

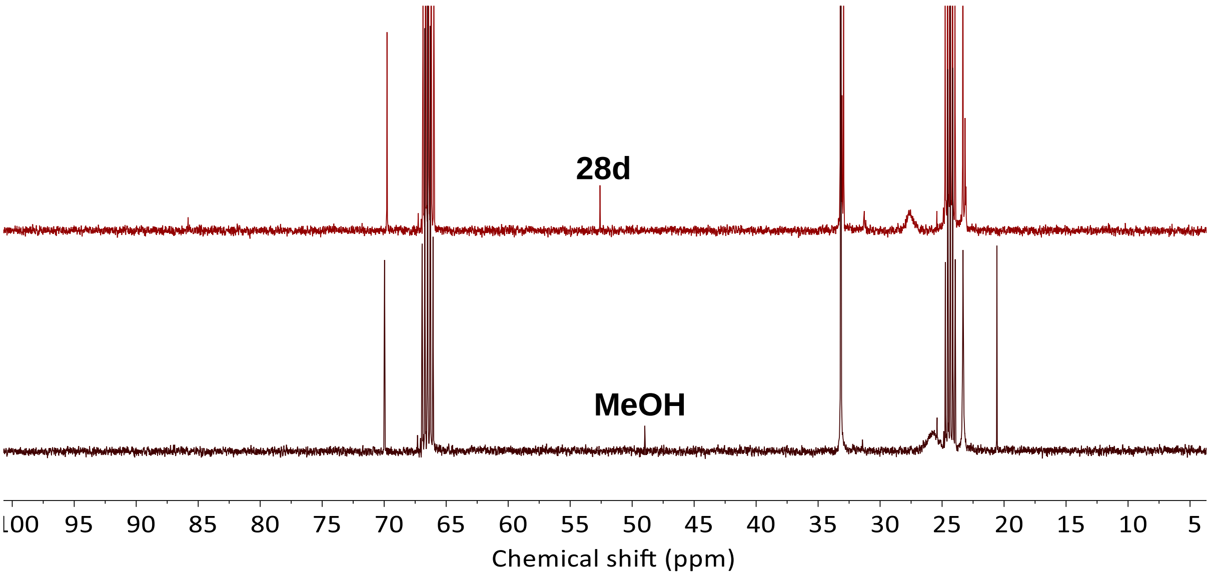

Figure S118. ^{13}C{^{1}H} NMR spectrum (reaction mixture) hydrolysis of **28d**. Top: before addition of H_2O. Bottom: after addition of H_2O.

5.8. Comparison to Literature Metal-free Catalysts for CO_2 Hydroboration

Table S7. Comparison of TON and TOF for Metal-free Catalysts Reported for CO_2 Hydroboration and [Na(18-c-6)]$_2$[**1**].

$$CO_2 + H{-}[B] \xrightarrow{\textbf{Cat}} MeO[B]$$

Catalysts [ref]	H–[B]	Solvent	Pressure CO_2 (atm)	T (°C)	Cat loading (mol%)	TON	TOF h^{-1}
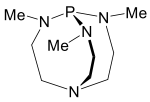 [10]	HBBN dimer	THF	1	20	0.01	6043	32
				70	0.5	100	287
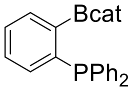 [11]	$BH_3{\cdot}SMe_2$	C_6D_6	2	70	0.33	2950	737
					0.1	853	853
$^tBu_3P$ [12]	BBN	C_6H_5Br	3	60	0.02	5556	176
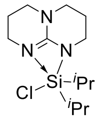 [13]	HBpin	THF	1	90	2.5	32	2
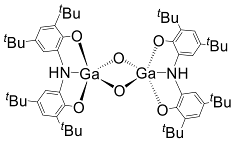 [14]	HBpin	C_6D_6	2	RT	1.0	99	2.6
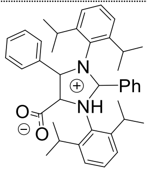 [15]	HBBN dimer	C_6D_6	1	RT	0.1	300	50
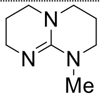 [16]	HBBN dimer	C_6D_6	1	RT	0.1	648	33
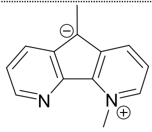 [17]	$BH_3{\cdot}SMe_2$	$CDCl_3$	1.5	RT	1	298	43
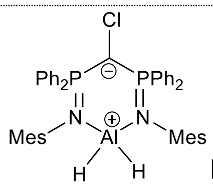 [18]	$BH_3{\cdot}SMe_2$	C_6D_6	1	110	1	293	293
					10	30	356
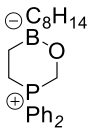 [19]	HBBN dimer	C_6D_6	1	60	0.1	341	49
						177	177
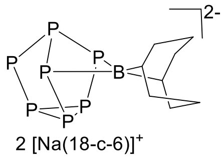 [this work]	HBBN dimer	*o*DFB: Tol	1	RT	0.01	9800	20
				50	0.33	300	300

Note: the table above is not an exhaustive list; catalysts that hydroborate CO_2 were selected based on their high performance or because they were studied under similar conditions as those used in the present study of [**1**]$^{2-}$.

6. Experimental Mechanistic Investigations

6.1. Addition H–[B] to [Na(18-c-6)]$_2$[1]

6.1.1. Addition (HBBN)$_2$ to [Na(18-c-6)]$_2$[1]

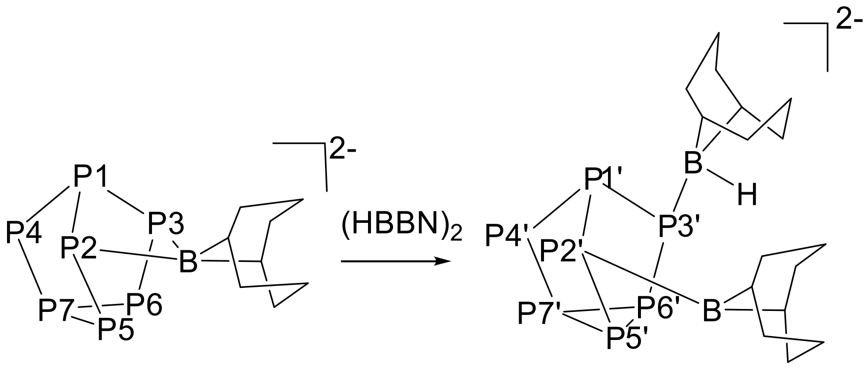

To a J Young NMR tube a solution of [Na(18-c-6)]$_2$[**1**] (50 mg, 0.055 mmol, 1 eq.) in *o*DFB and HBBN dimer (6.5 mg, 0.027 mmol, 1 eq.) was added. The reaction was monitored by ^{11}B, ^{11}B{^{1}H} and ^{31}P NMR.

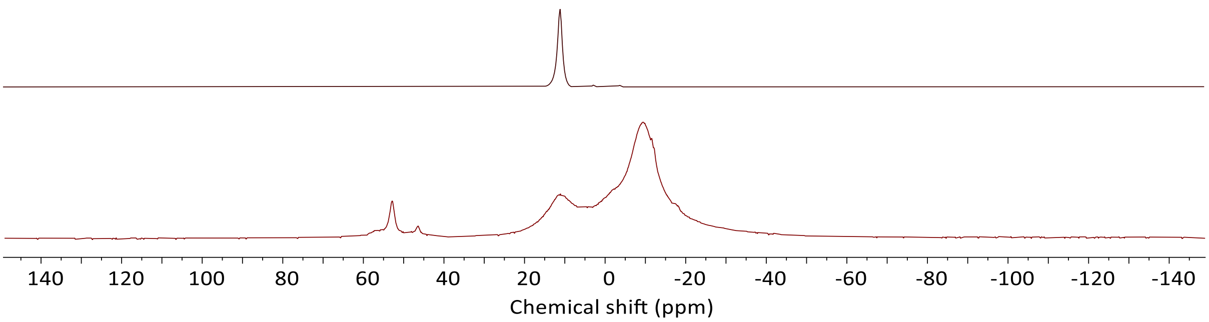

Figure S119. Coordination of BBN to [**1**]$^{2-}$. Top: ^{11}B NMR spectrum (*o*DFB) of [Na(18-c-6)]$_2$[**1**]. Bottom: ^{11}B NMR spectrum (*o*DFB) of [Na(18-c-6)]$_2$[**1**] + (BBN)$_2$.

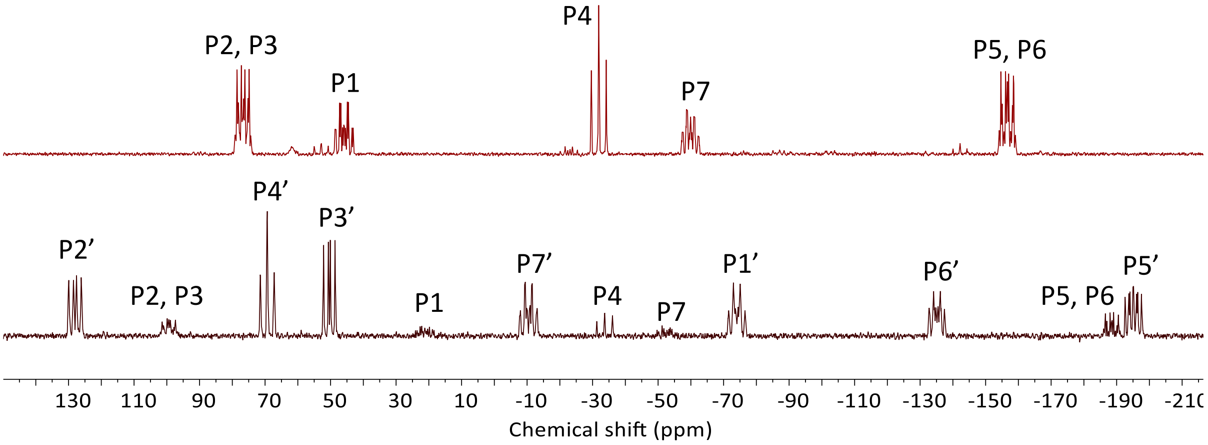

Figure S120. Coordination of BBN to [**1**]$^{2–}$. Top: ^{31}P NMR spectrum (*o*DFB) of [Na(18-c-6)]$_2$[**1**]. Bottom: ^{31}P NMR spectrum (*o*DFB) of [Na(18-c-6)]$_2$[**1**] + HBBN dimer.

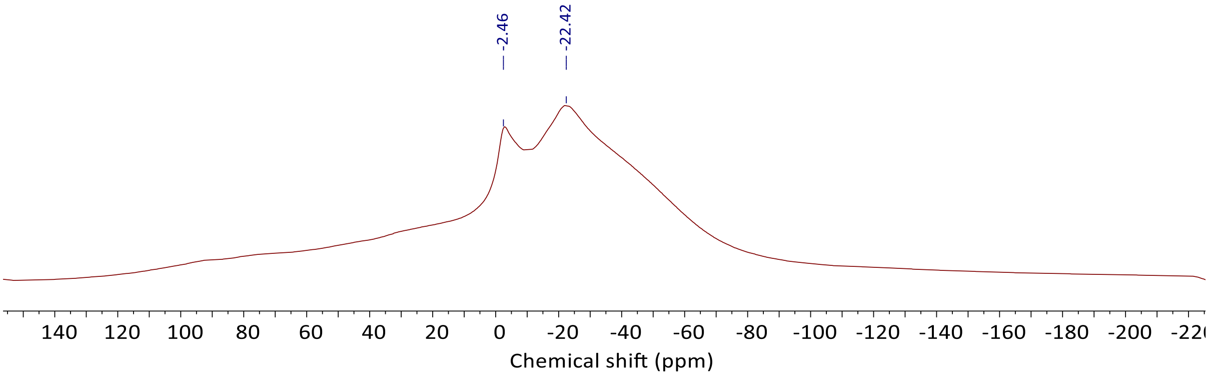

Figure S121. Coordination of BBN to [**1**]$^{2–}$. ^{11}B SS NMR of [Na(18-c-6)]$_2$[**1**]+ HBBN dimer . MAS 12 KHz. Note: Sample started melting in the rotor.

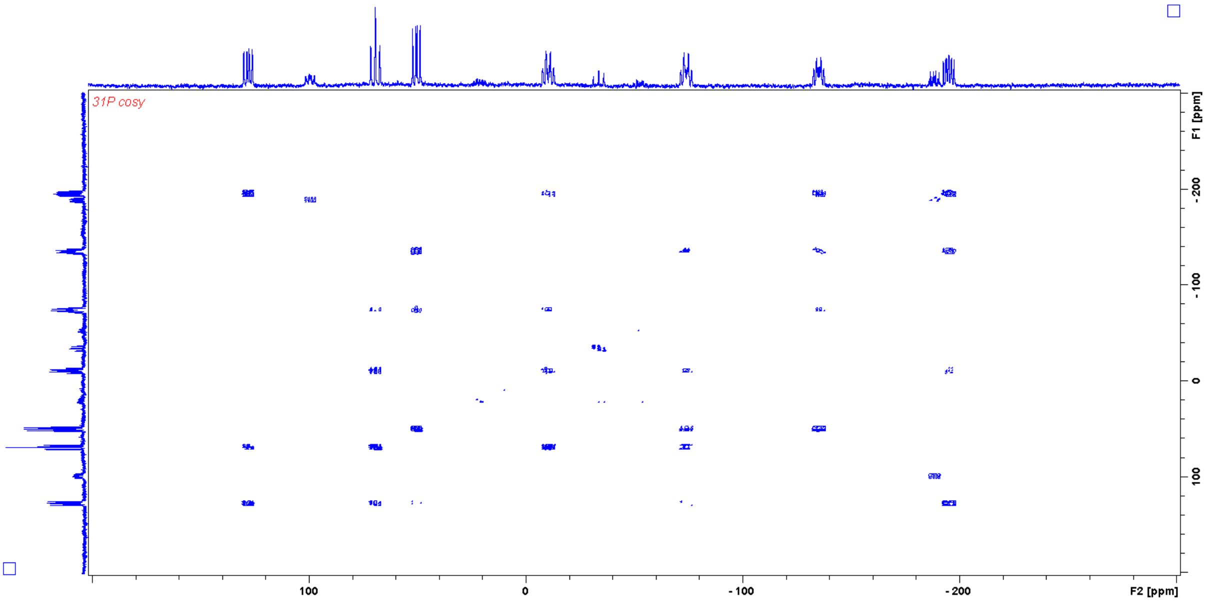

Figure S122. ^{31}P COSY NMR spectrum (*o*DFB) of $[Na(18\text{-}c\text{-}6)]_2$[**1**] + HBBN dimer.

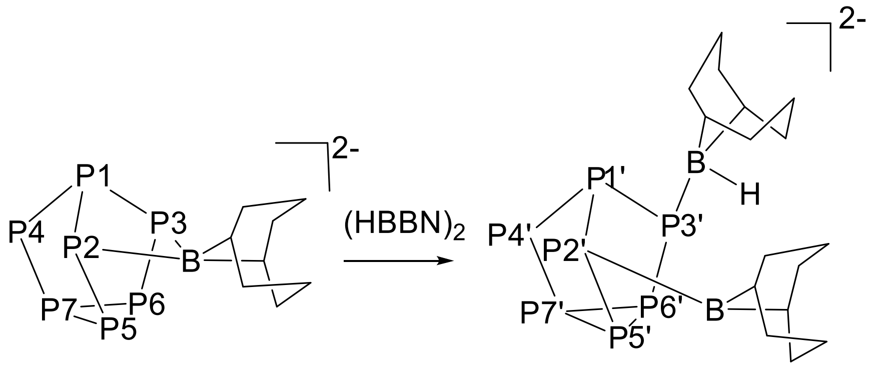

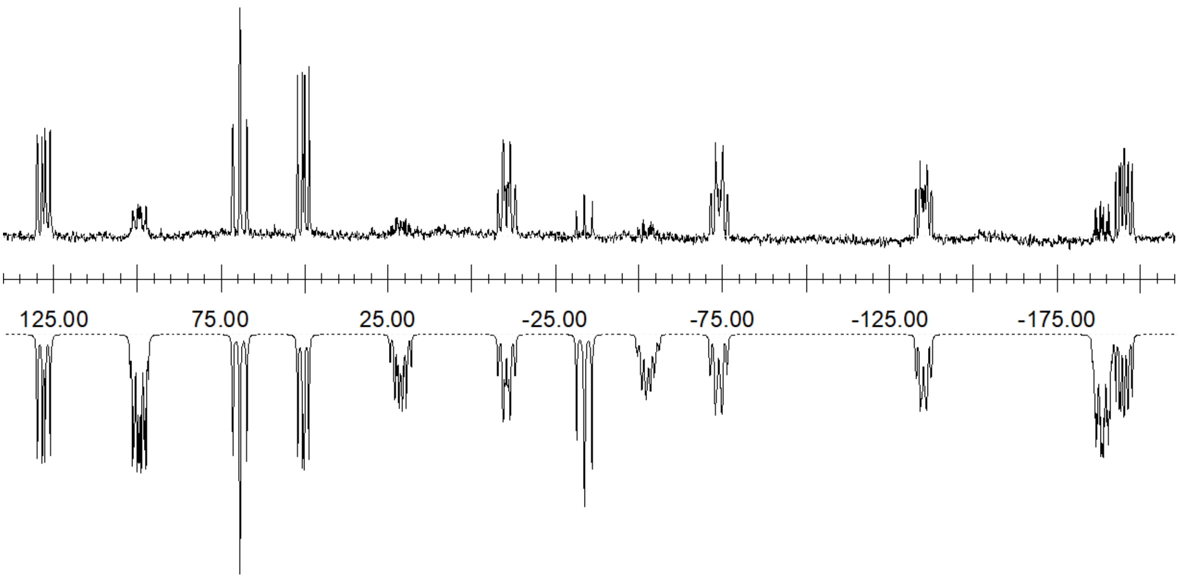

Figure S123. ^{31}P NMR spectrum (*o*DFB) of $[Na(18\text{-}c\text{-}6)]_2$[**1**] + HBBN dimer.Top: Experimental NMR spectra; bottom: simulated NMR spectrum.

Table S8. Simulated NMR spectroscopic values

Nucleus	Chemical shift (ppm)	Nucleus	Chemical shift (ppm)
P1	–52.69	P1’	–73.87
P2, P3	99.39	P2’	127.95
P4	–33.73	P3’	50.5
P5	21.22	P4’	69.29
P6, P7	–188.55	P5’	–195.07
		P6’	–135.14
		P7’	–10.43

J coupling	coupling constant (Hz)	*J* coupling	coupling constant (Hz)
^1JP1–P2	231	^1JP1’–P2’	252
^1JP1–P3	231	^1JP1’–P3’	232
^1JP1–P4	392	^1JP1’–P4’	335
^2JP1–P5	55.	^2JP1’–P5’	37
^2JP1–P6	53.	^2JP1’–P6’	38
^2JP1–P7	72.	^2JP1’–P7’	0
^1JP4–P5	350	^1JP2’–P5’	378
^1JP2–P6	425	^1JP3’–P6’	305
^1JP3–P7	425	^1JP4’–P7’	342
^1JP5–P6	235	^1JP5’–P6’	183
^1JP5–P7	243	^1JP5’–P7’	240
^1JP6–P7	192	^1JP6’–P7’	236
Final residual	1.63e+06	Final residual	8.25e+05

6.1.2. Addition HBpin to [Na(18-c-6)]$_2$[1]

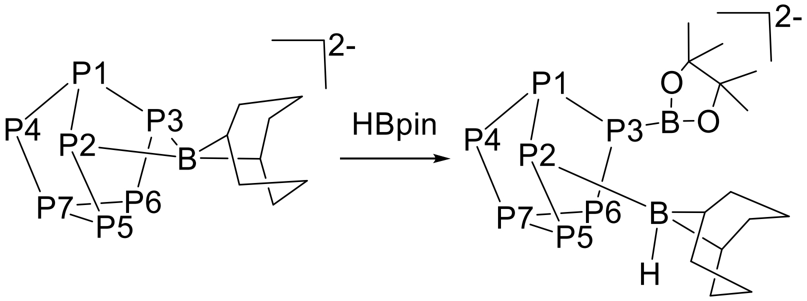

To a J Young NMR tube a solution of [Na(18-c-6)]$_2$[**1**] (25 mg, 0.027 mmol, 1.0 eq.) in *o*DFB and HBpin (3.9 µL, 0.027 mmol, 1.0 eq.) was added. The reaction was monitored by ^{11}B, ^{11}B{^{1}H} and ^{31}P NMR. ^{11}B{^{1}H} and ^{31}P NMR.

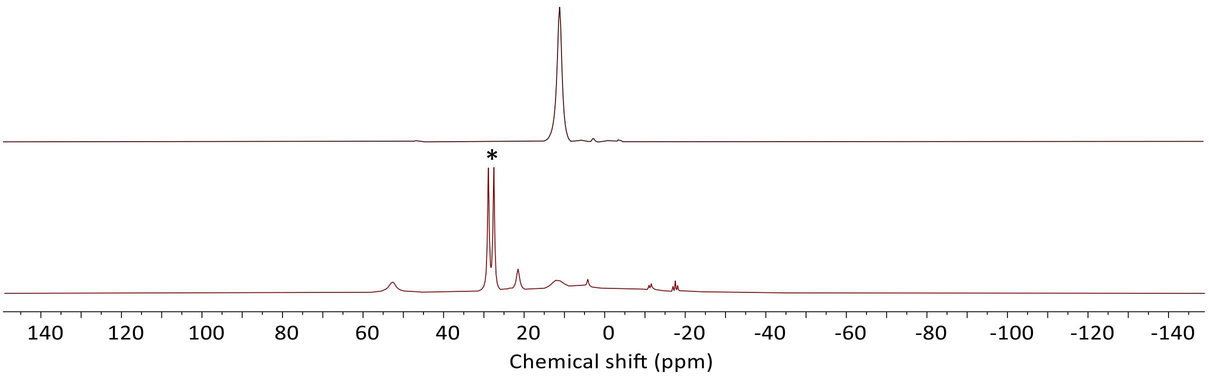

Figure S124. Coordination of HBpin to [**1**]$^{2-}$. Top: ^{11}B NMR spectrum (*o*DFB) of [Na(18-c-6)]$_2$[**1**]. Bottom: ^{11}B NMR spectrum (*o*DFB) of [Na(18-c-6)]$_2$[**1**] + HBpin. HBpin marked by *.

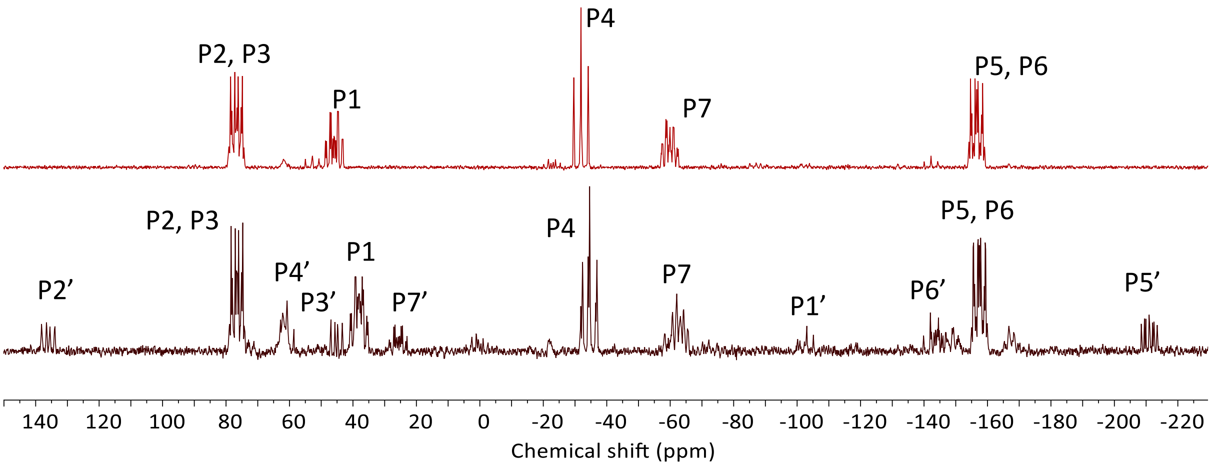

Figure S125. Coordination of HBpin to [**1**]$^{2-}$. Top: ^{31}P NMR spectrum (*o*DFB) of [Na(18-c-6)]$_2$[**1**]. Bottom: ^{31}P NMR spectrum (*o*DFB) of [Na(18-c-6)]$_2$[**1**] + HBpin.

6.2. Addition of Carbonyls to [Na(18-c-6)]$_2$[1]

6.2.1. Addition Benzaldehyde to [Na(18-c-6)]$_2$[1]

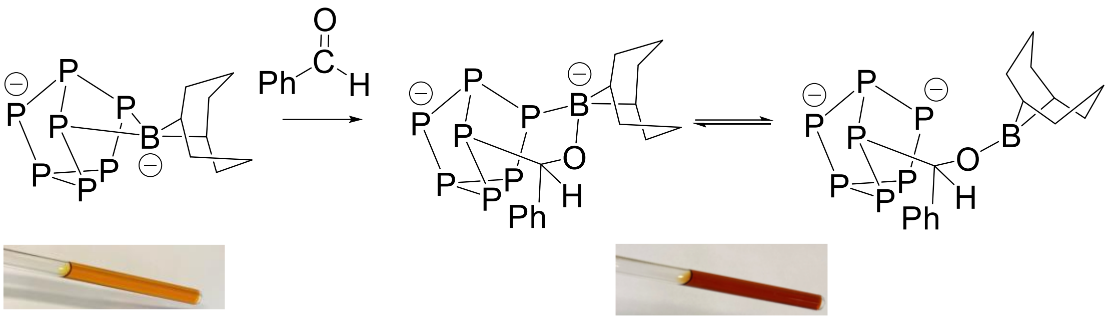

To a J Young NMR tube a solution of [Na(18-c-6)]$_2$[**1**] (25 mg, 0.027 mmol) in *o*DFB and benzaldehyde (2.8 µL, 0.027 mmol) was added. The reaction was monitored by ^{11}B, ^{11}B{^{1}H} and ^{31}P NMR. Color change observed from orange to red.

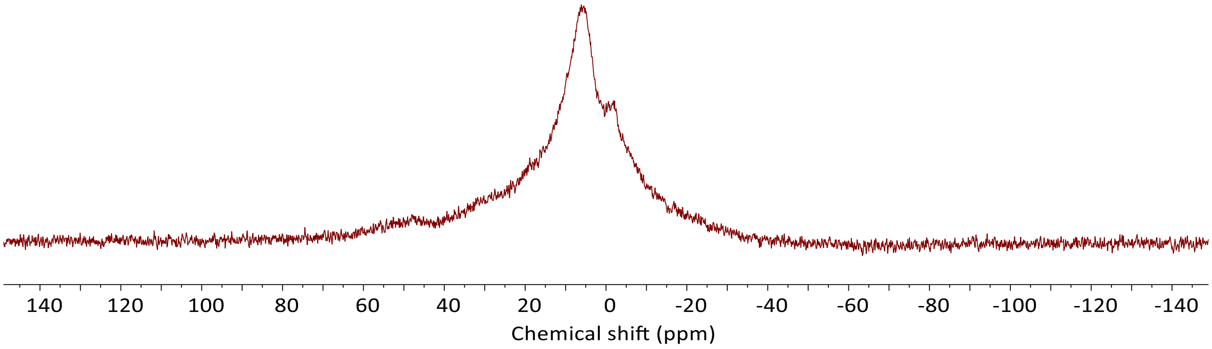

Figure S126. ^{11}B NMR spectrum (*o*DFB) of [Na(18-c-6)]$_2$[**1**] + benzaldehyde.

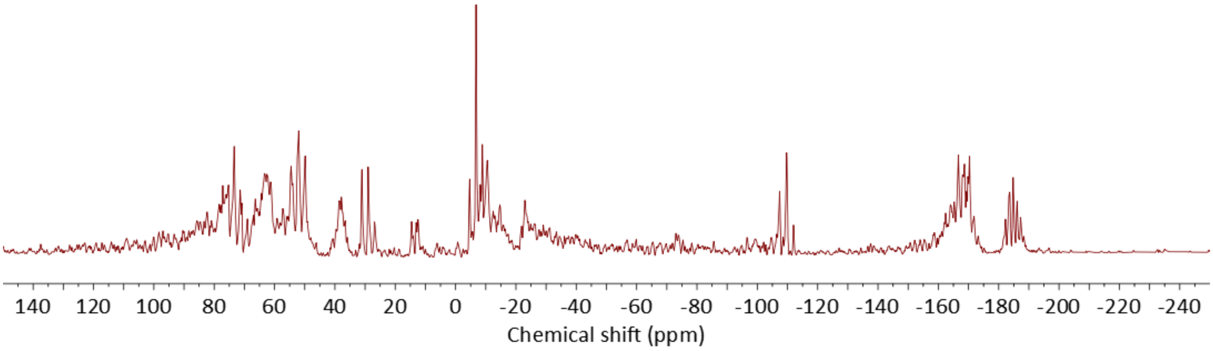

Figure S127. ^{31}P NMR spectrum (*o*DFB) of [Na(18-c-6)]$_2$[**1**] + benzaldehyde. Note: No unreacted [Na(18-c-6)]$_2$[**1**] observed.

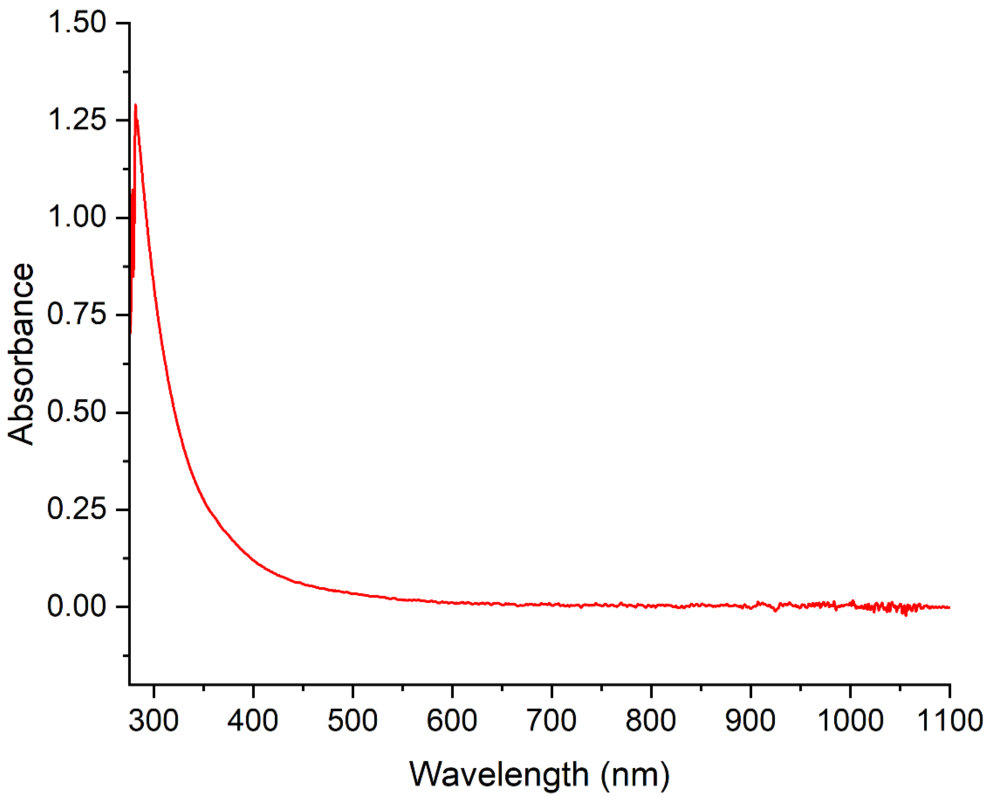

Figure S128. Uv-Vis spectrum of [**1**]$^{2-}$ + benzaldehyde (0.11 mM in *o*DFB).

6.2.2. Addition Acetophenone to [Na(18-c-6)]$_2$[1]

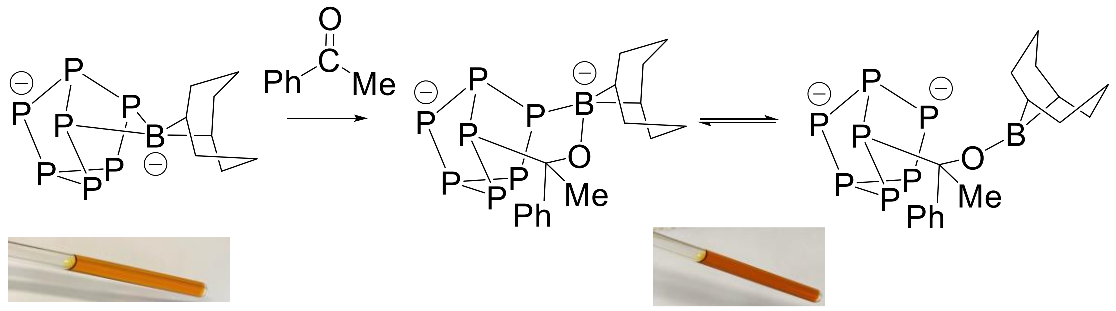

To a J Young NMR tube a solution of [Na(18-c-6)]$_2$[**1**] (25 mg, 0.027 mmol) in *o*DFB and acetophenone (3.0 µL, 0.027 mmol) was added. The reaction was monitored by ^{11}B, ^{11}B{^{1}H} and ^{31}P NMR. Color change observed from orange to red.

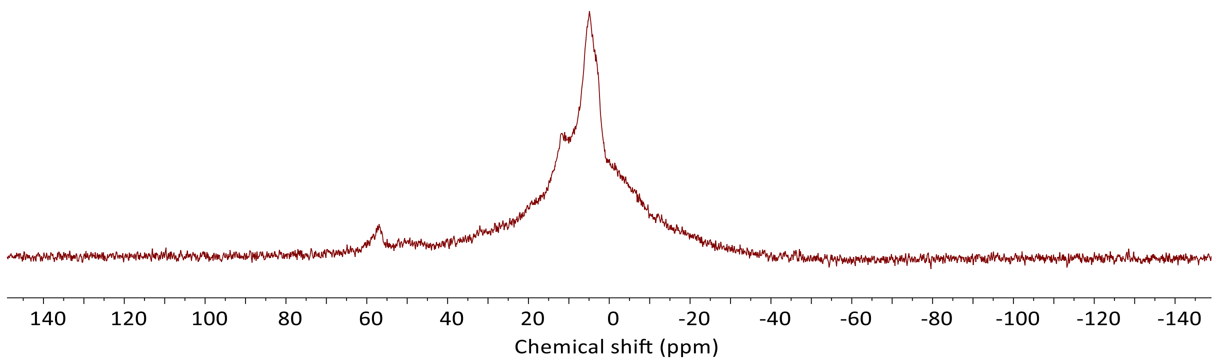

Figure S129. ^{11}B NMR spectrum (*o*DFB) of $[Na(18\text{-}c\text{-}6)]_2$[**1**] + acetophenone.

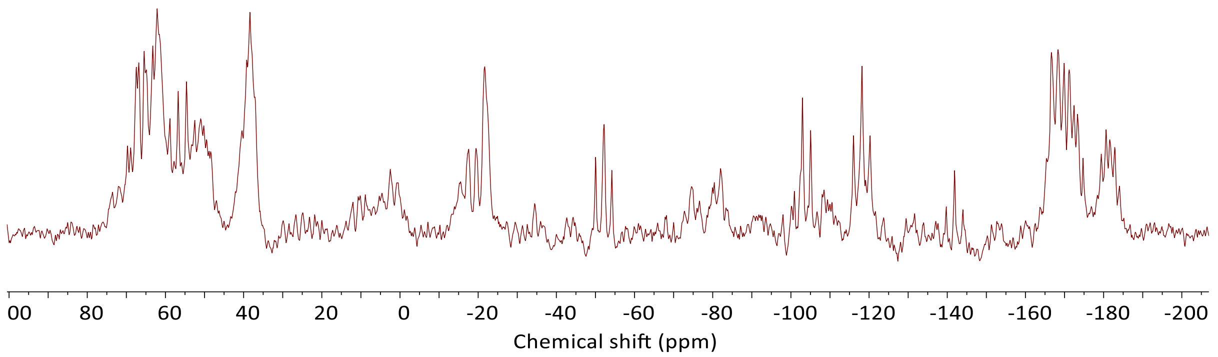

Figure S130. ^{31}P NMR spectrum (*o*DFB) of $[Na(18\text{-}c\text{-}6)]_2$[**1**] + acetophenone. Note: No unreacted $[Na(18\text{-}c\text{-}6)]_2$[**1**] observed.

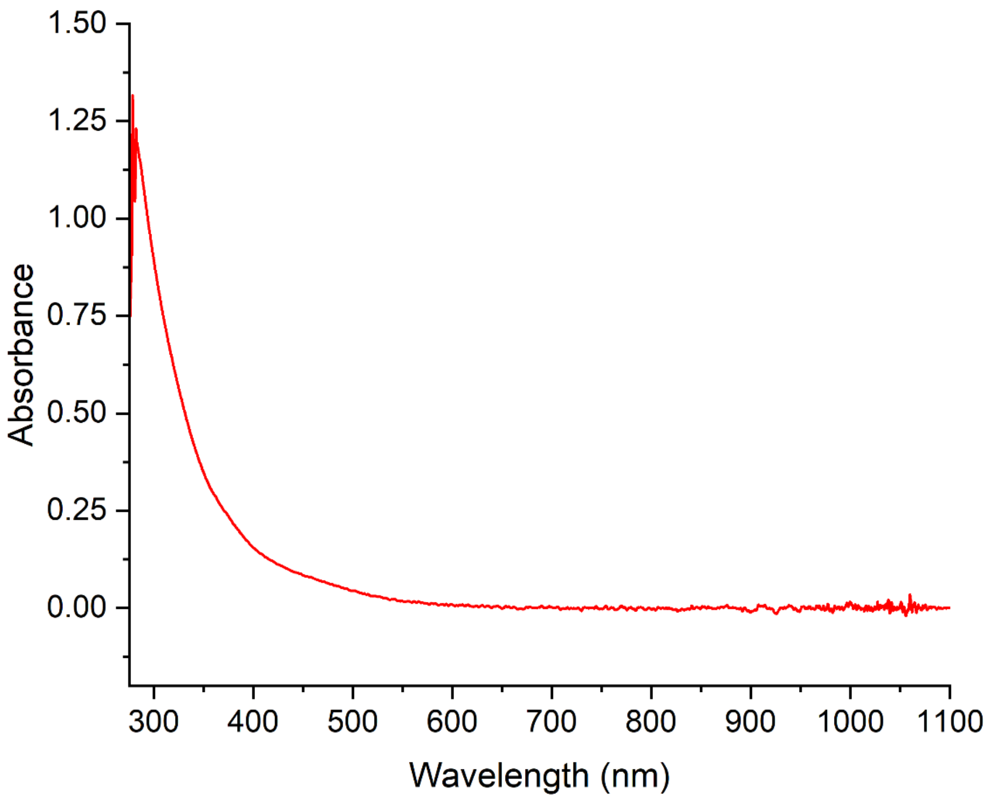

Figure S131. Uv-Vis spectrum of [**1**]$^{2-}$ + acetophenone (0.11 mM in *o*DFB).

6.3. Addition of Heteroallenes to [Na(18-c-6)]$_2$[1]

6.3.1. Addition phenyl isocyanate to [Na(18-c-6)]$_2$[1]

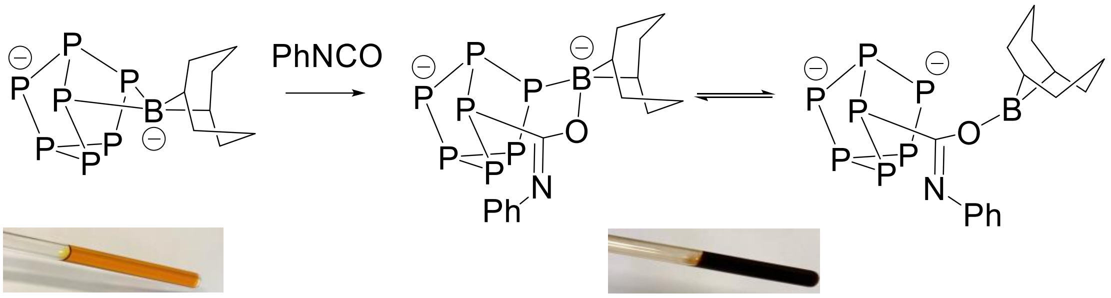

To a J Young NMR tube a solution of [Na(18-c-6)]$_2$[**1**] (25 mg, 0.027 mmol) in *o*DFB and phenyl isocyanate (3.0 µL, 0.027 mmol) was added. The reaction was monitored by ^{11}B, ^{11}B{^{1}H} and ^{31}P NMR. Color change observed from orange to dark red.

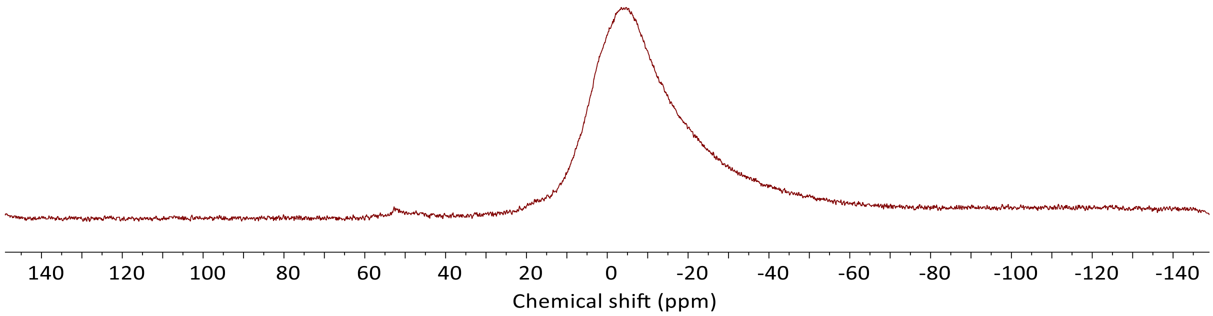

Figure S132. ^{11}B NMR spectrum (*o*DFB) of $[Na(18\text{-}c\text{-}6)]_2$[**1**] + phenyl isocyanate.

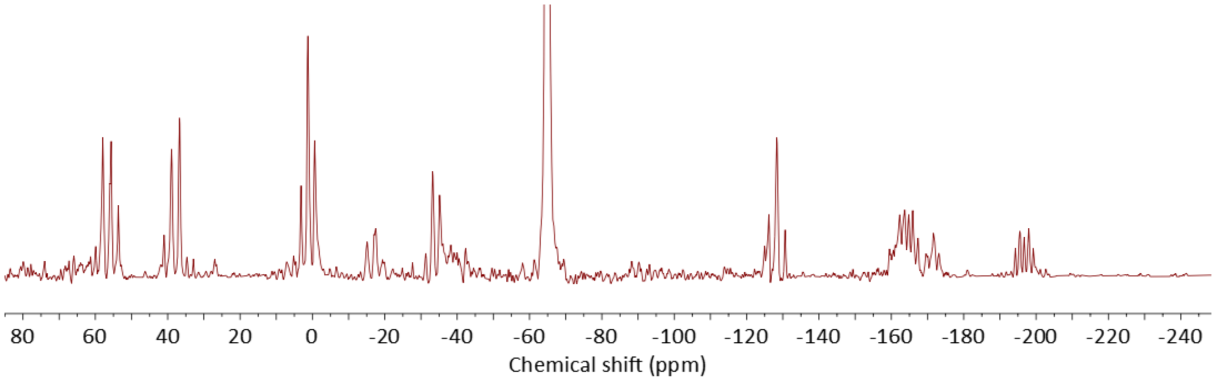

Figure S133. ^{31}P NMR spectrum (*o*DFB) of $[Na(18\text{-}c\text{-}6)]_2$[**1**] + phenyl isocyanate.
Note: No unreacted $[Na(18\text{-}c\text{-}6)]_2$[**1**] observed.

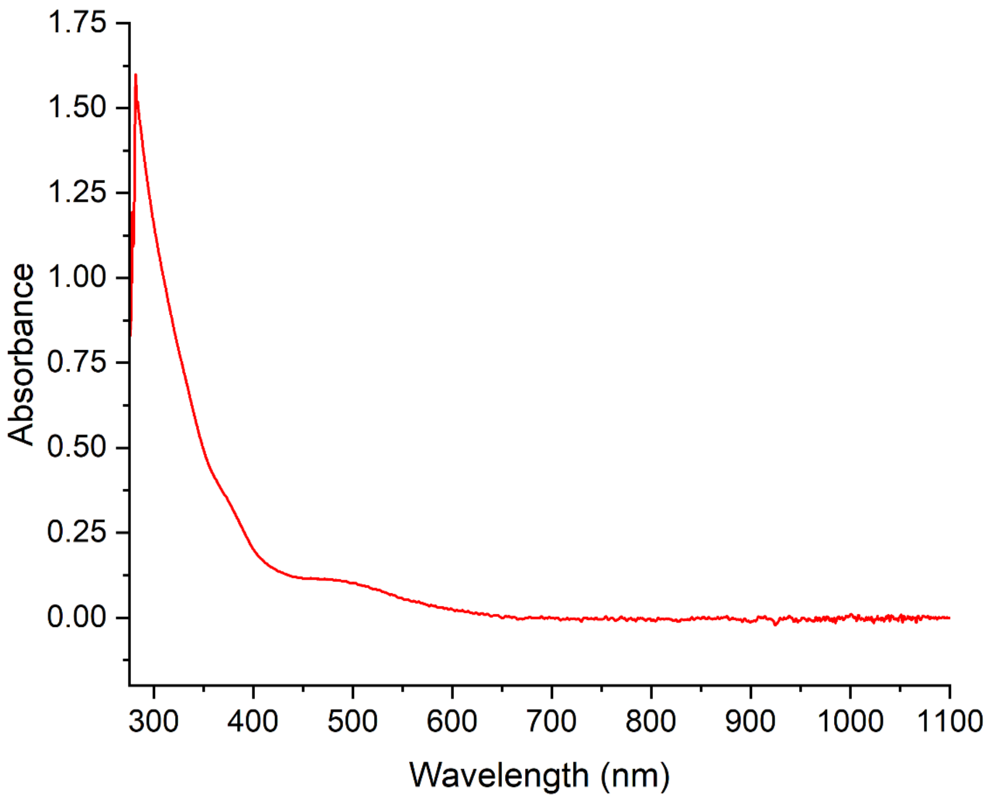

Figure S134. Uv-Vis spectrum of [**1**]$^{2-}$ + phenyl isocyanate (0.11 mM in *o*DFB).

6.3.2. Addition CO_2 to [Na(18-c-6)]$_2$[1]

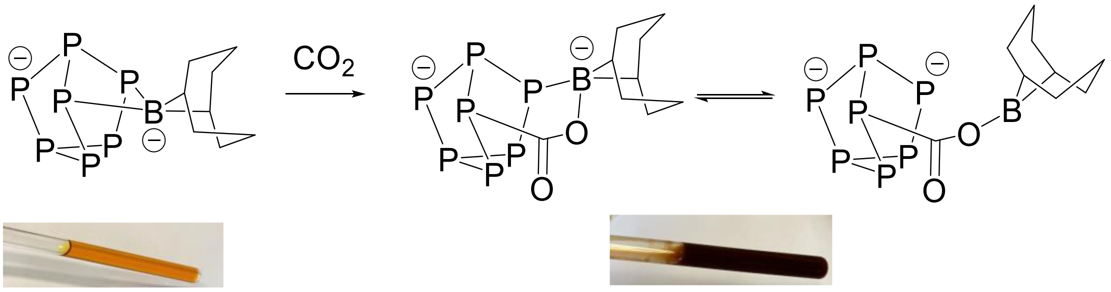

To a J Young NMR tube a solution of [Na(18-c-6)]$_2$[**1**] (25 mg, 0.027 mmol) in *o*DFB was added. The mixture was degassed and refilled with CO_2 (1 atm). The reaction was monitored by ^{11}B, ^{11}B{^{1}H}, ^{13}C{^{1}H} and ^{31}P NMR. Color change observed from orange to dark red.

Note: Applying a vacuum to the product does not result in any observable reversibility.

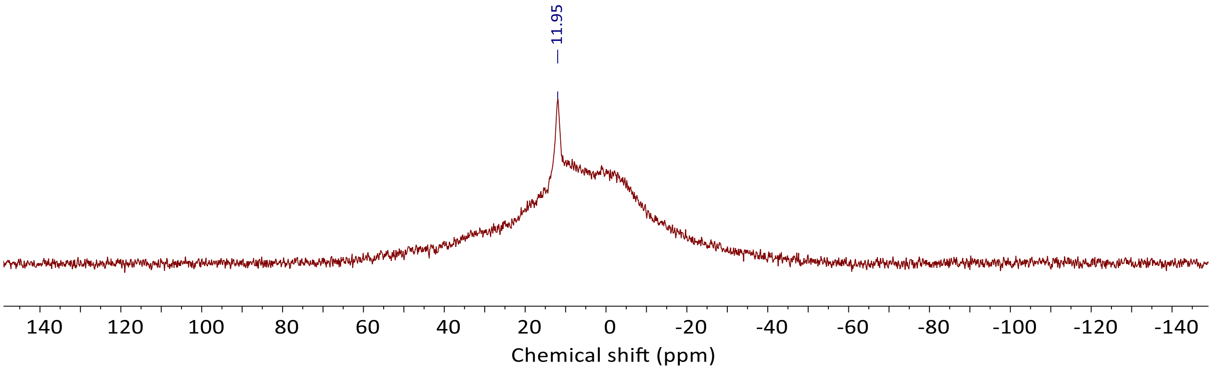

Figure S135. ^{11}B NMR spectrum (*o*DFB) of [Na(18-c-6)]$_2$[**1**] + CO_2.

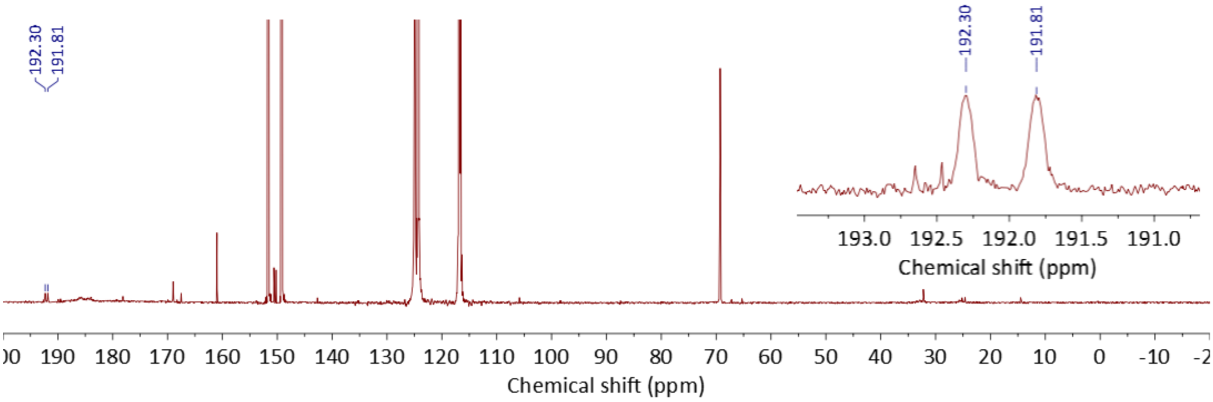

Figure S136. $^{13}C\{^1H\}$ NMR spectrum (*o*DFB) of [Na(18-c-6)]$_2$[**1**] + CO_2.

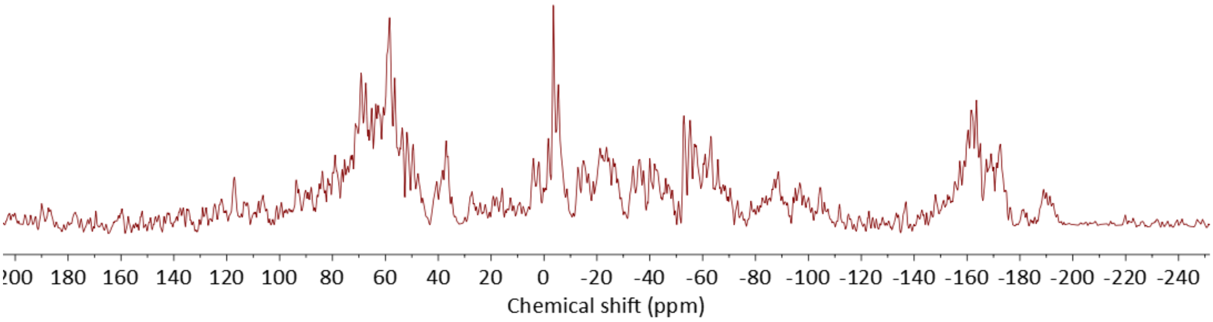

Figure S137. ^{31}P NMR spectrum (*o*DFB) of [Na(18-c-6)]$_2$[**1**] + CO_2. Note: No unreacted [Na(18-c-6)]$_2$[**1**] observed.

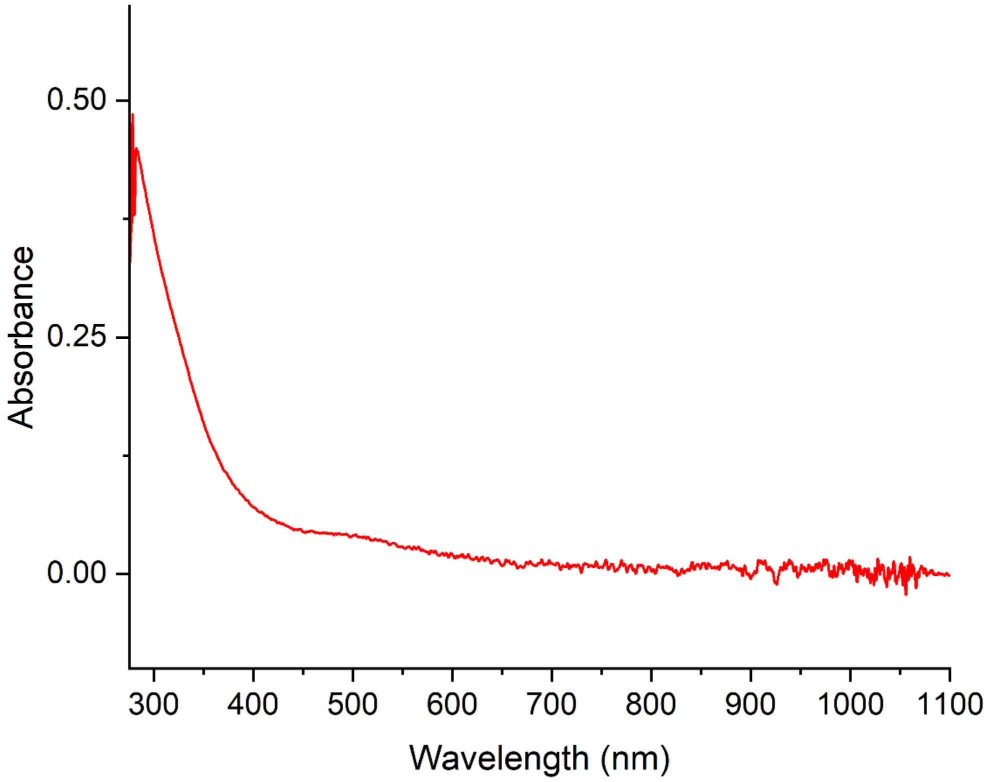

Figure S138. Uv-Vis spectrum of [**1**]$^{2-}$ + CO_2 (0.11 mM in *o*DFB).

6.4. Selected NMR Hydroboration Aldehydes/Ketones

Following the general hydroboration procedure from section 3.2., during the reactions the resonances observed marked below are believed to be arising from an **I4** in the mechanism shown in the manuscript as Figure 4. Noteworthy is the exact same coupling constant observed to the adjacent C*H*=CHR moiety for the hydroboration product, Ha, Hb and Hc in Figure S139 and S140.

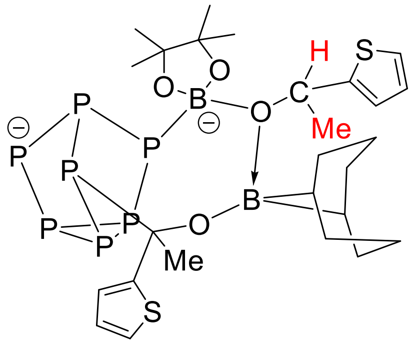

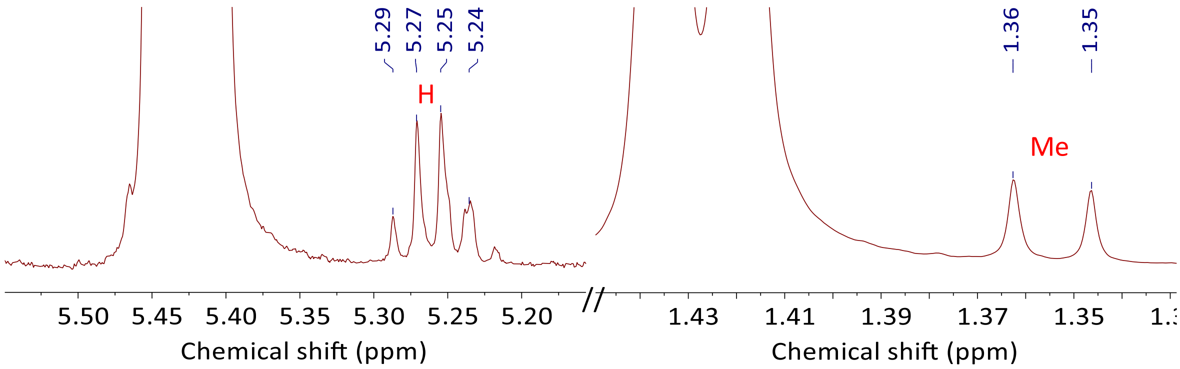

Figure S139. ^{1}H NMR spectrum after 30 min (reaction mixture) of crude **17b**.

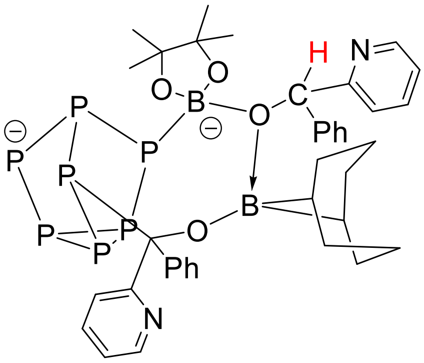

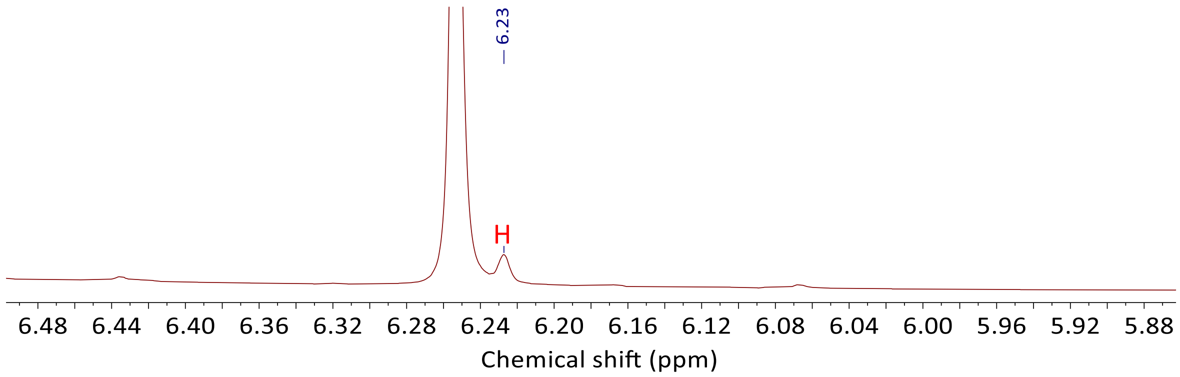

Figure S140. ^{1}H NMR spectrum after 30 min (reaction mixture) of crude **17b**.

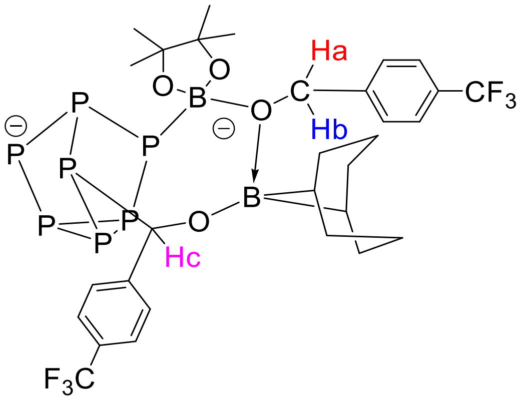

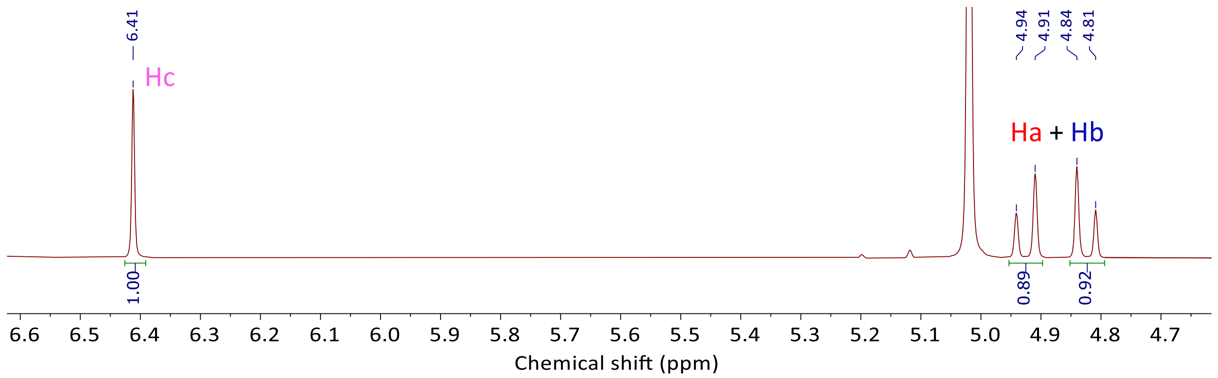

Figure S141. ^{1}H NMR spectrum after 30 min (reaction mixture) of crude **7b**.

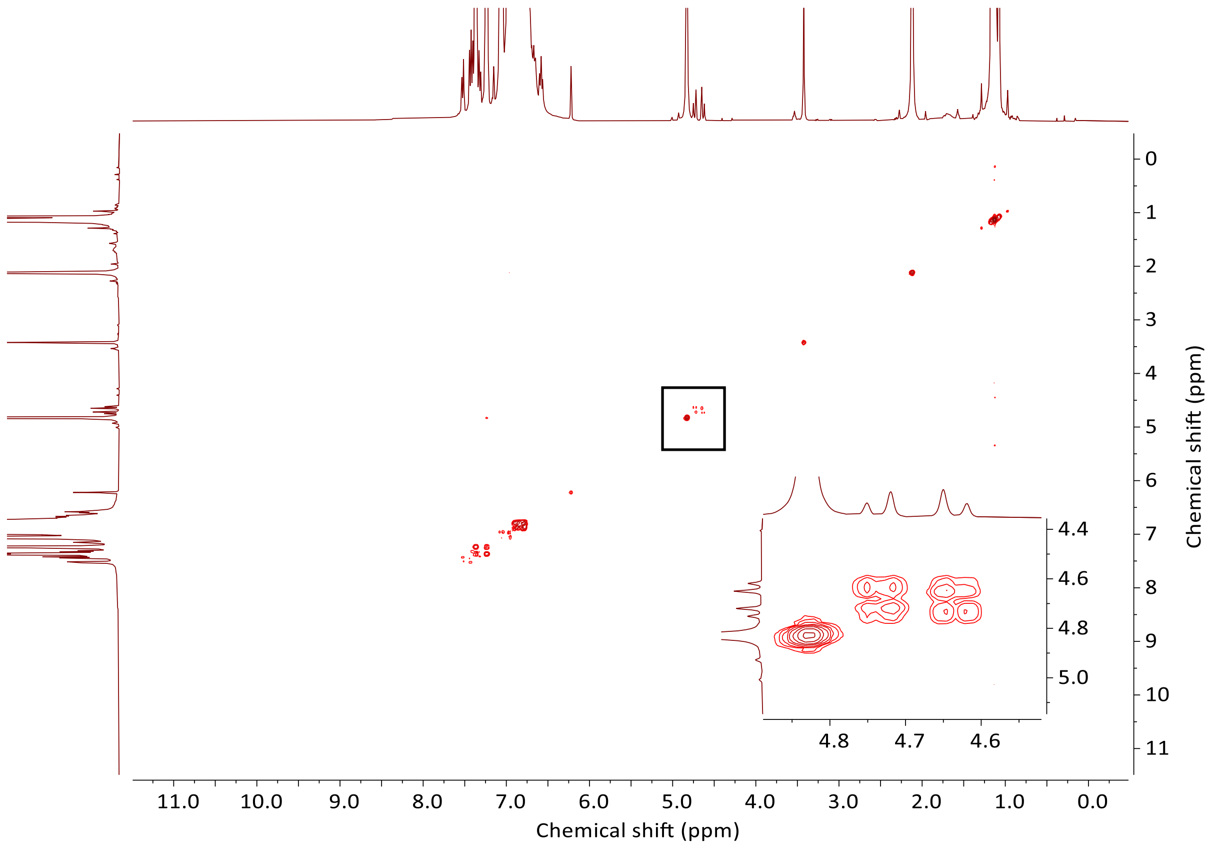

Figure S142. ^{1}H COSY NMR spectrum after 120 min (reaction mixture) of crude **7b**. The highlighted resonances in the box are believed to be arising from **I4**.

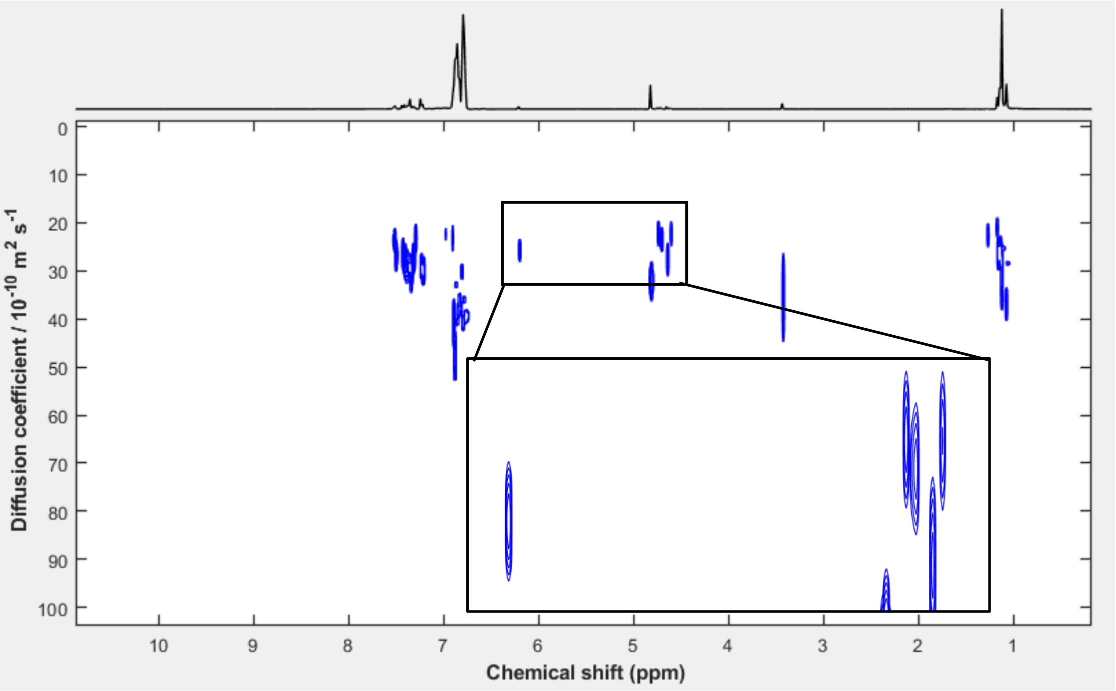

Figure S143. ^{1}H DOSY NMR spectrum after 60 min (reaction mixture) of crude **7b**. The highlighted resonances in the box are believed to be arising from **I4**.

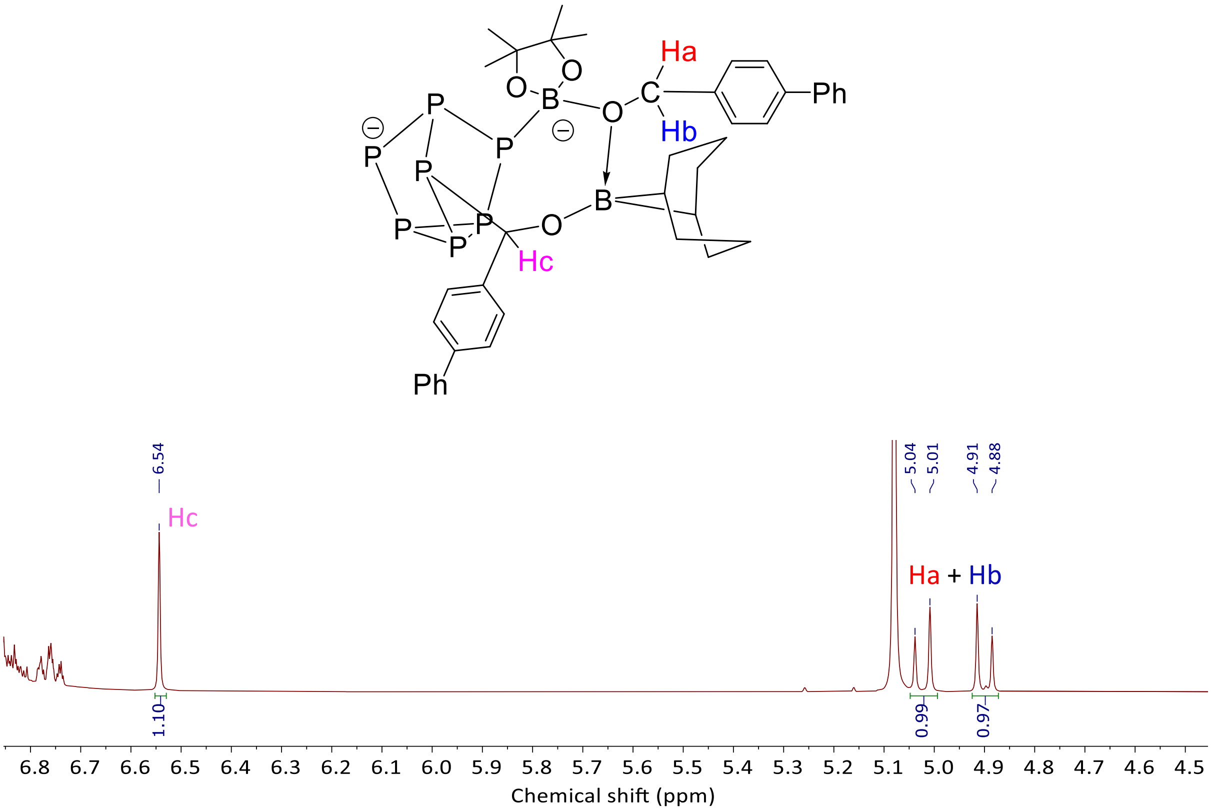

Figure S144. ^{1}H NMR spectrum after 30 min (reaction mixture) of crude **5b**.

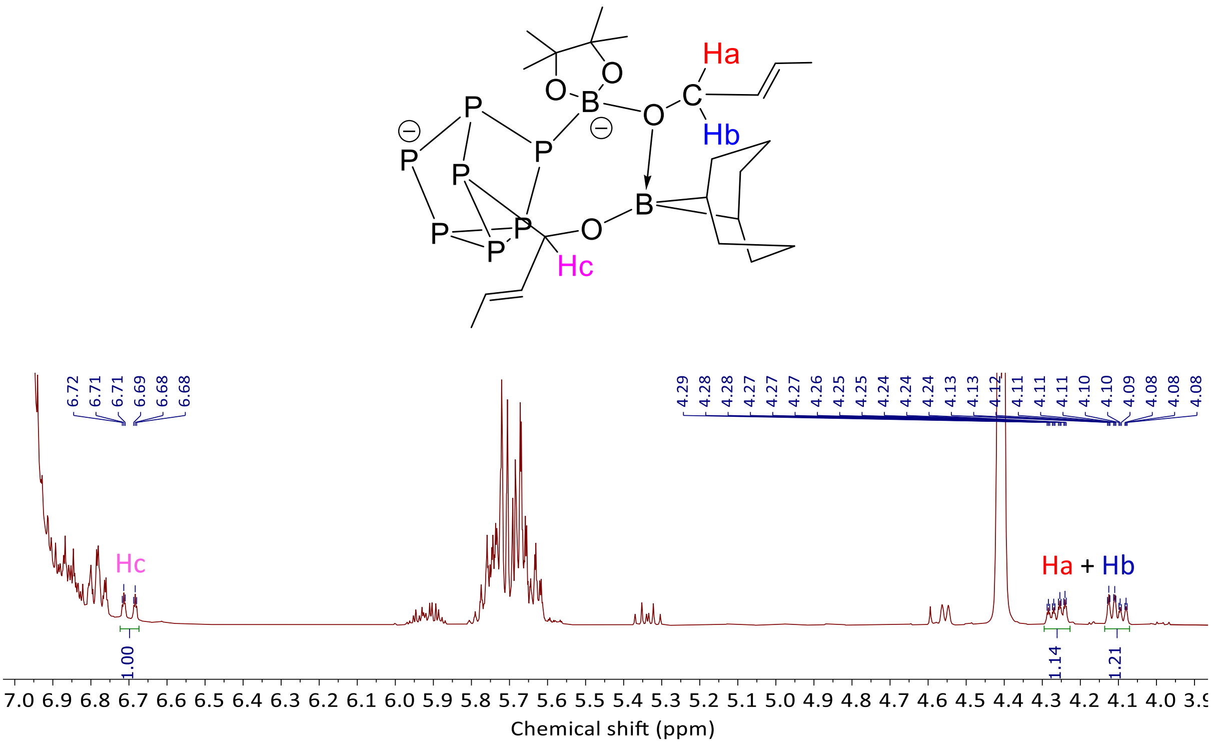

Figure S145. ^{1}H NMR spectrum after 30 min (reaction mixture) of crude **11b**.

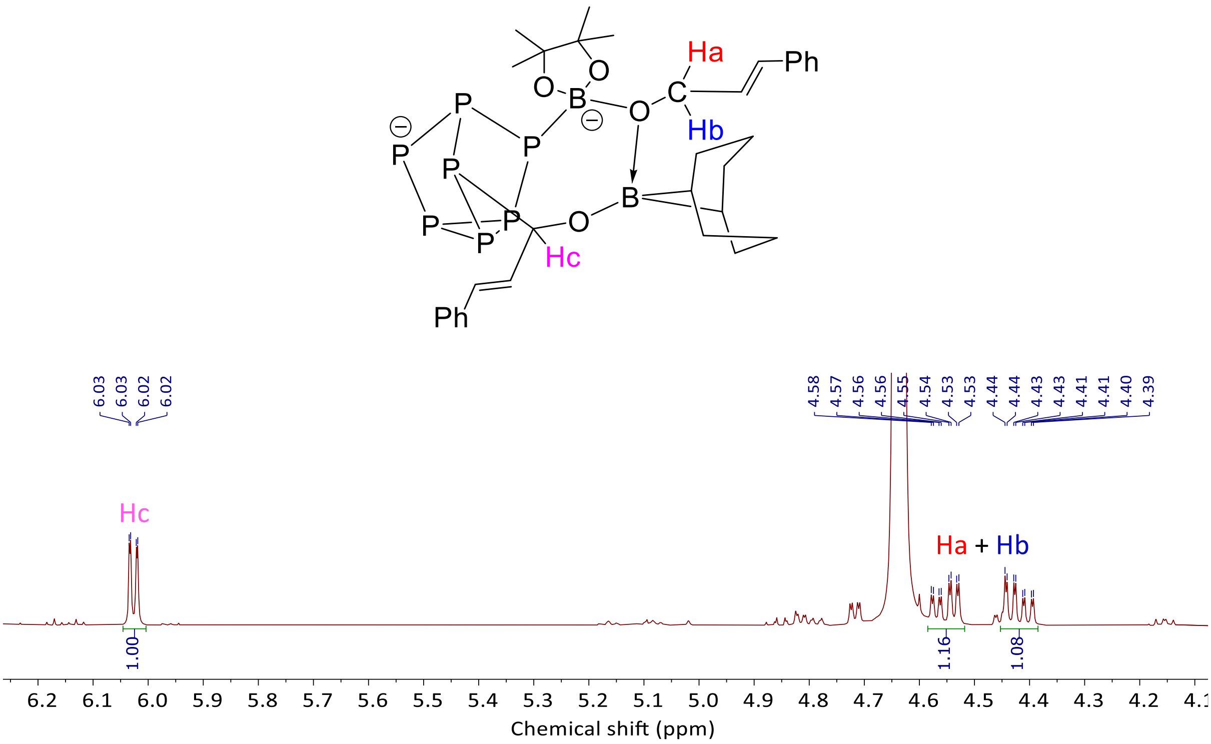

Figure S146. ^{1}H NMR spectrum after 30 min (reaction mixture) of crude **12b**.

6.5. Stoichiometric Hydroboration of Acetophenone

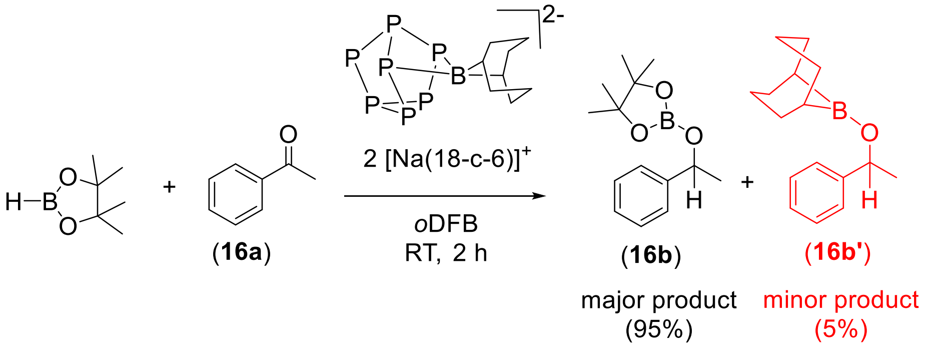

Toa J Young NMR tube a solution of [Na(18-c-6)]$_2$[**1**] (25 mg, 0.027 mmol, 1.0 eq.) in *o*DFB HBpin (4.0 µL, 0.027 mmol, 1.0 eq.), acetophenone (3.2 µL, 0.027 mmol, 1.0 eq.), and toluene (25 µL, 0.24 mmol) was added. The reaction was monitored by ^{1}H, ^{11}B, ^{11}B{^{1}H} and ^{31}P NMR. Using the toluene as an internal standard, the reaction gave an overall conversion of 95% conversion. Product distribution was 95:5 **16b**:**16b'**. Compound **16b'** was independently synthesized using the general ketone hydroboration procedure (section 3) to aid in identifying resonances observed. The ^{31}P NMR spectrum recorded after the reaction shows compound [**1**]$^{2-}$ and some other weak resonances which are presumed to be [(Bpin)P$_7$]$^{2-}$, marked by *.

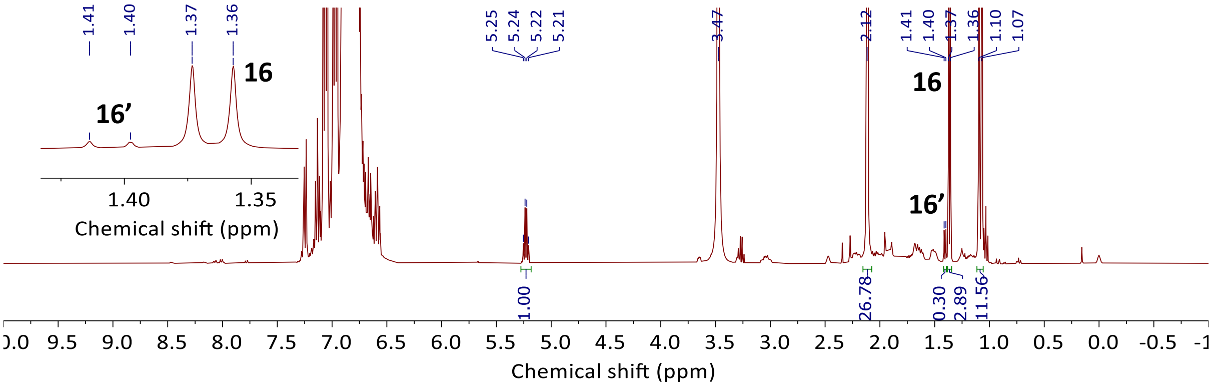

Figure S147. ^{1}H NMR spectrum stoichiometric reduction **16a** (reaction mixture).

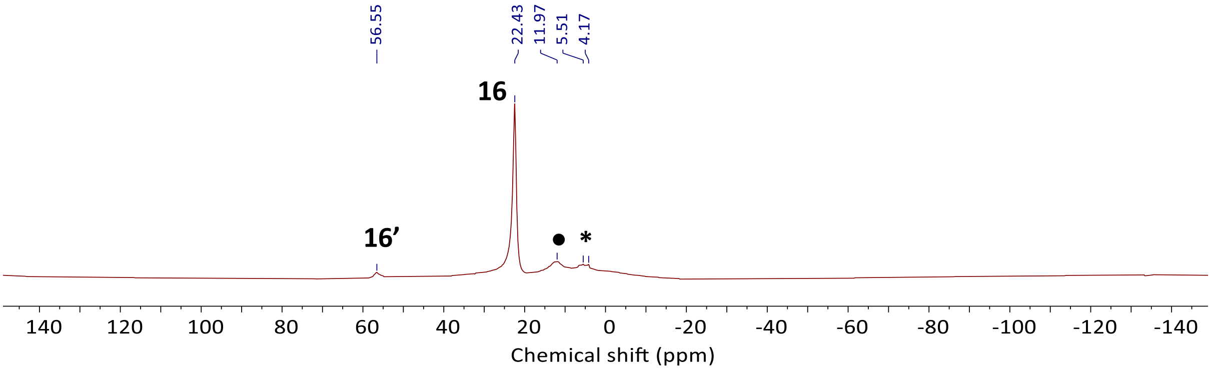

Figure S148. ^{11}B NMR spectrum stoichiometric reduction **16a** (reaction mixture). [**1**]$^{2-}$ marked by •. Potential [(Bpin)P_7]$^{2-}$ marked by *.

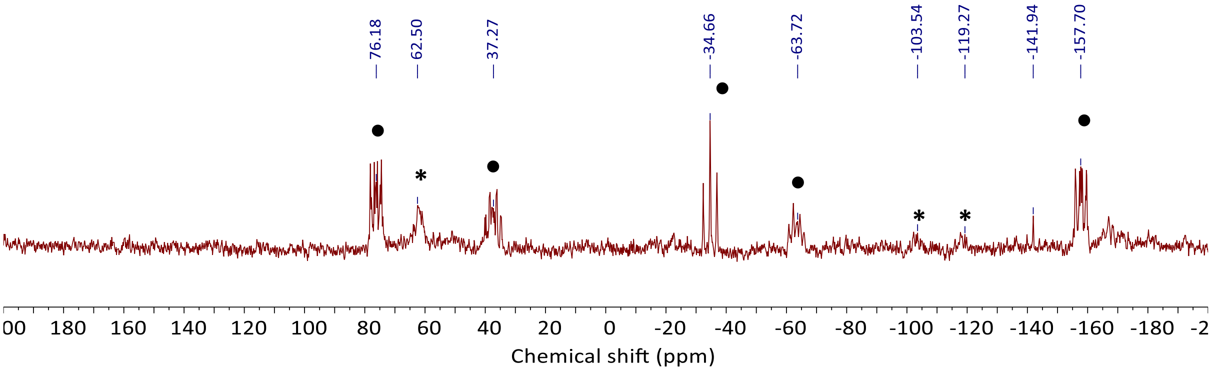

Figure S149. ^{31}P NMR spectrum stoichiometric reduction **16a** (reaction mixture). [**1**]$^{2-}$ marked by •. Potential [(Bpin)P_7]$^{2-}$ marked by *.

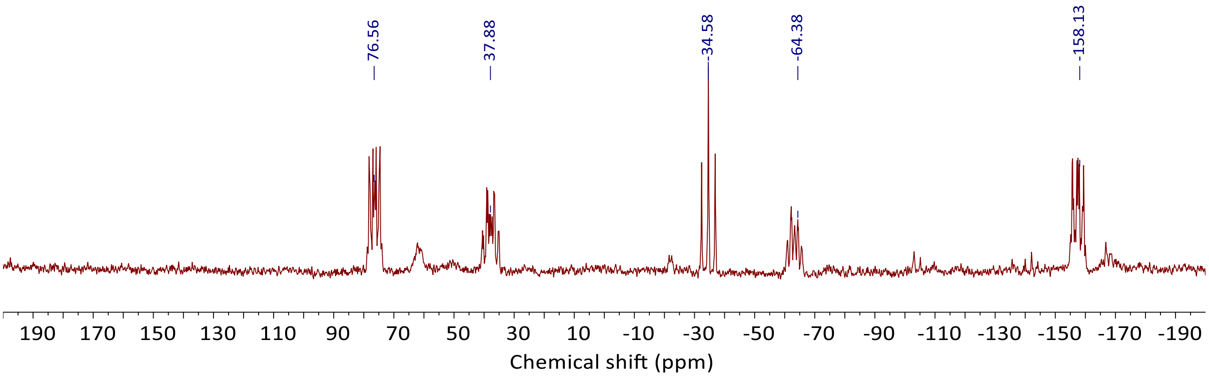

Figure S150. ^{31}P NMR spectrum recovered catalysts after the stoichiometric reduction **16a** (*o*DFB).

6.6. Stoichiometric Hydroboration of CO_2

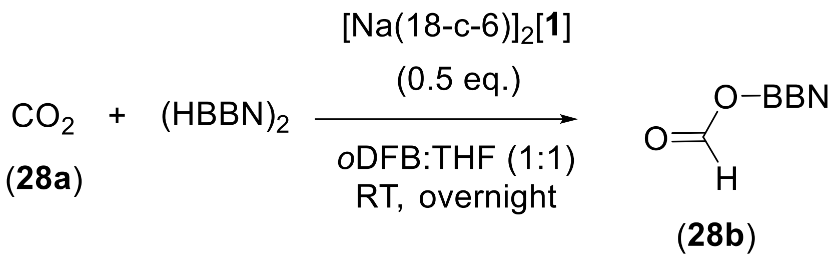

To a J Young ampoule a solution of [Na(18-c-6)]$_2$[**1**] (25 mg, 0.027 mmol, 0.5 eq.) in THF:*o*DFB (0.6 mL, 1:1) and HBBN dimer (3.4 mg, 0.014 mmol, 1.0 eq.) was added. The reaction mixture was degassed and the headspace was refilled with CO_2 (1 atm). The reaction was monitored by ^{1}H, ^{11}B and ^{11}B{^{1}H} NMR. After complete consumption of HBBN dimer was confirmed by ^{11}B NMR spectroscopy the solvent was removed under reduced pressure. The residue was extracted with diethyl ether. From this concentrated diethyl ether solution crystals suitable for single crystal X-ray diffraction analysis were obtained through slow evaporation of solvent. Single crystal X-ray diffraction analysis showed two formyl moieties bounded to a single BBN moiety. This product presumably formed by the work-up. It is reported in literature that the Bpin and Bcat analogues of **28b** are not isolable.[20-22] No spectroscopic changes are observed when the reaction is performed using 2.0 eq. HBBN dimer.

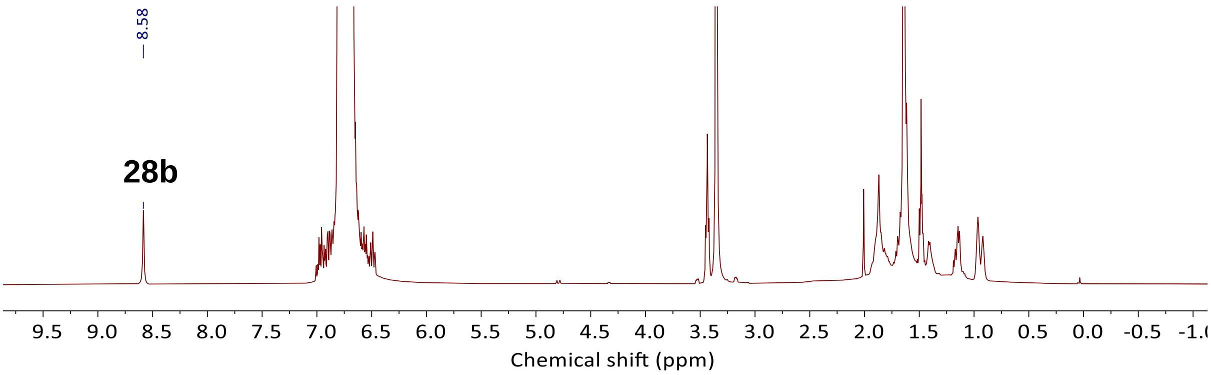

Figure S151. ^{1}H NMR spectrum (*o*DFB:THF) of crude **28b**.

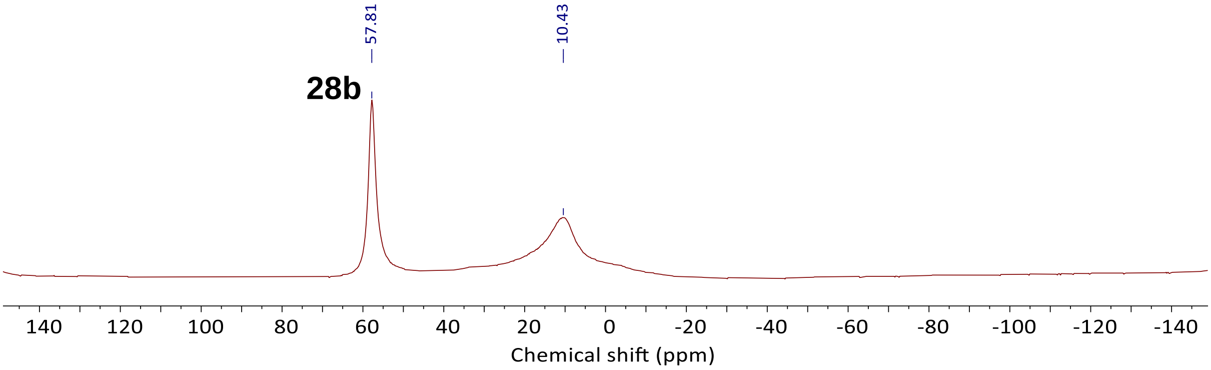

Figure S152. ^{11}B NMR spectrum (*o*DFB:THF) of crude **28b**.

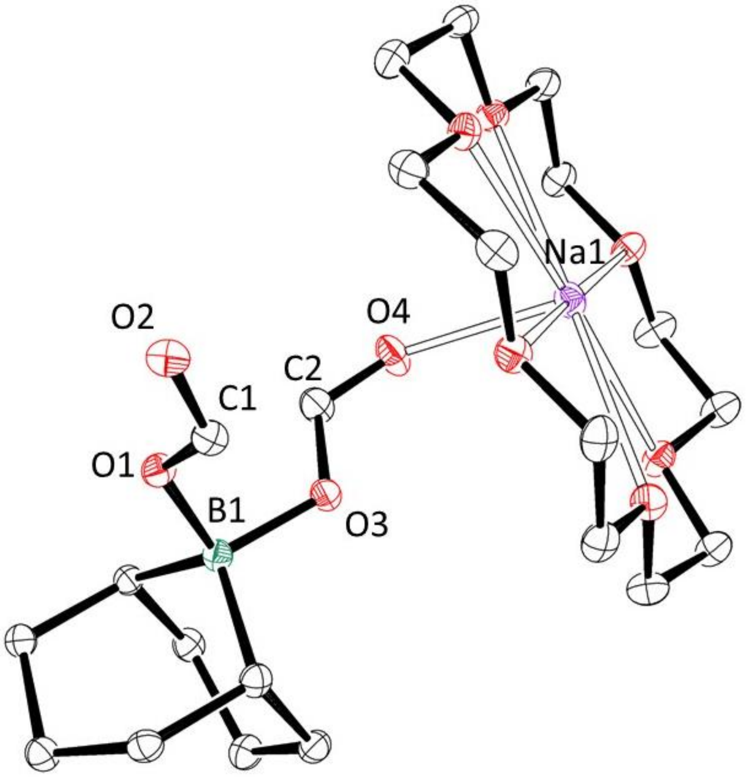

Figure S153. Molecular structure of **28b'**. Anisotropic displacement ellipsoids pictured at 50% probability. Hydrogen atoms and counter cations omitted for clarity. Phosphorus: Orange; Boron: Green; Carbon: White.

6.7. Hydroboration of formyl-BBN (28b)

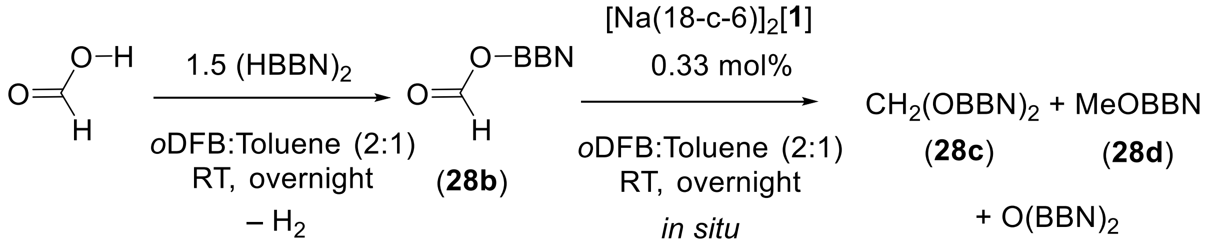

To a J Young ampoule a solution of HBBN dimer (36 mg, 0.15 mmol, 3.0 eq.) in *o*DFB : toluene (0.6 mL, 2:1), formic acid (3.8 µL, 0.10 mmol, 1 eq.) and C_6Me_6 (10.5 mg, 0.065 mmol) was added. Formation of H_2 gas was observed. The reaction was monitored by ^1H, ^{11}B and $^{11}B\{^1H\}$ NMR. The NMR conv. of **28b** (95%) was calculated by integration of the crude ^1H NMR spectrum using the C_6Me_6 as an internal standard (^1H δ = 2.20 ppm). After 18 h, a solution of $[Na(18\text{-}c\text{-}6)]_2$[**1**] (0.9 mg, 0.99 µmol, 0.0033 eq.) in *o*DFB (0.1 mL) was added. The reaction was monitored by ^1H, ^{11}B and $^{11}B\{^1H\}$ NMR. The NMR conv. of **28d** (92%) after 24 h was calculated by integration of the crude ^1H NMR spectrum using the C_6Me_6 as an internal standard (^1H δ = 2.20 ppm).

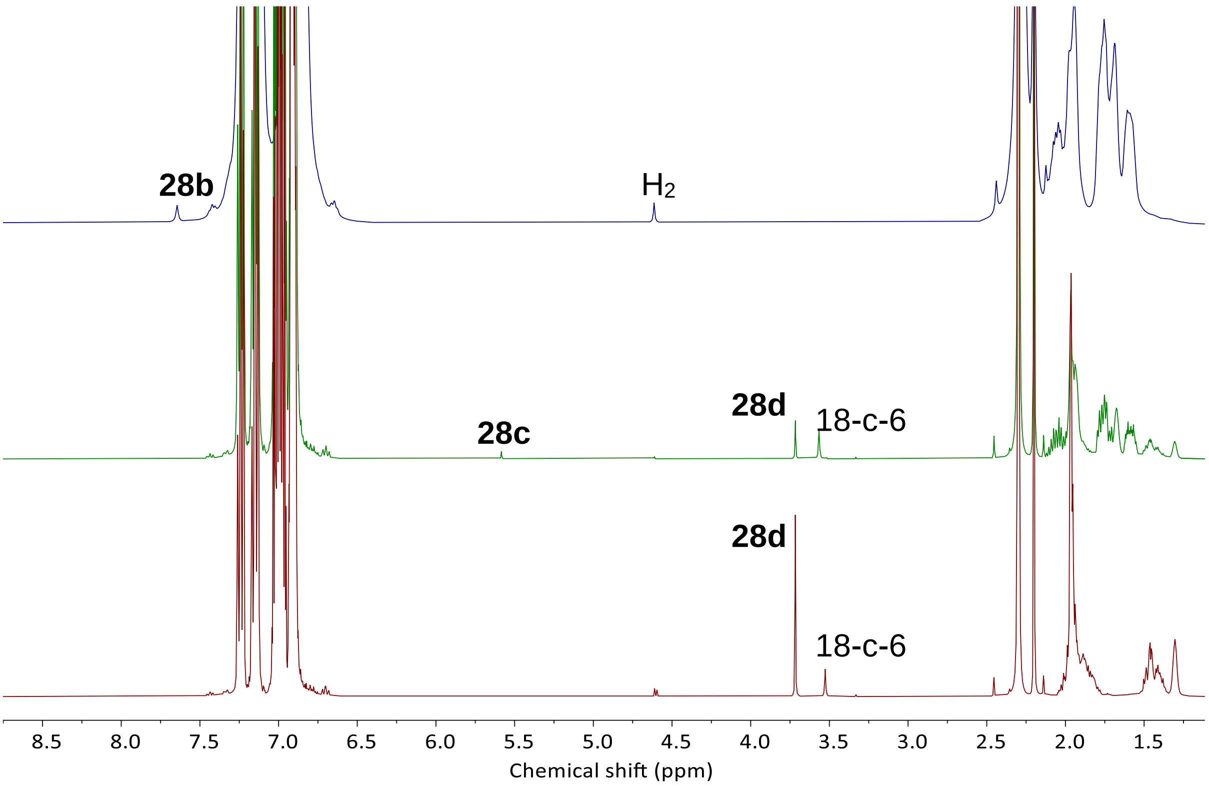

Figure S154. ^1H NMR spectrum (reaction mixture) of hydroboration of **28b**. Top spectrum recorded after 0 h; Middle spectrum recorded after 1 h; Bottom spectrum recoded after 18 h.

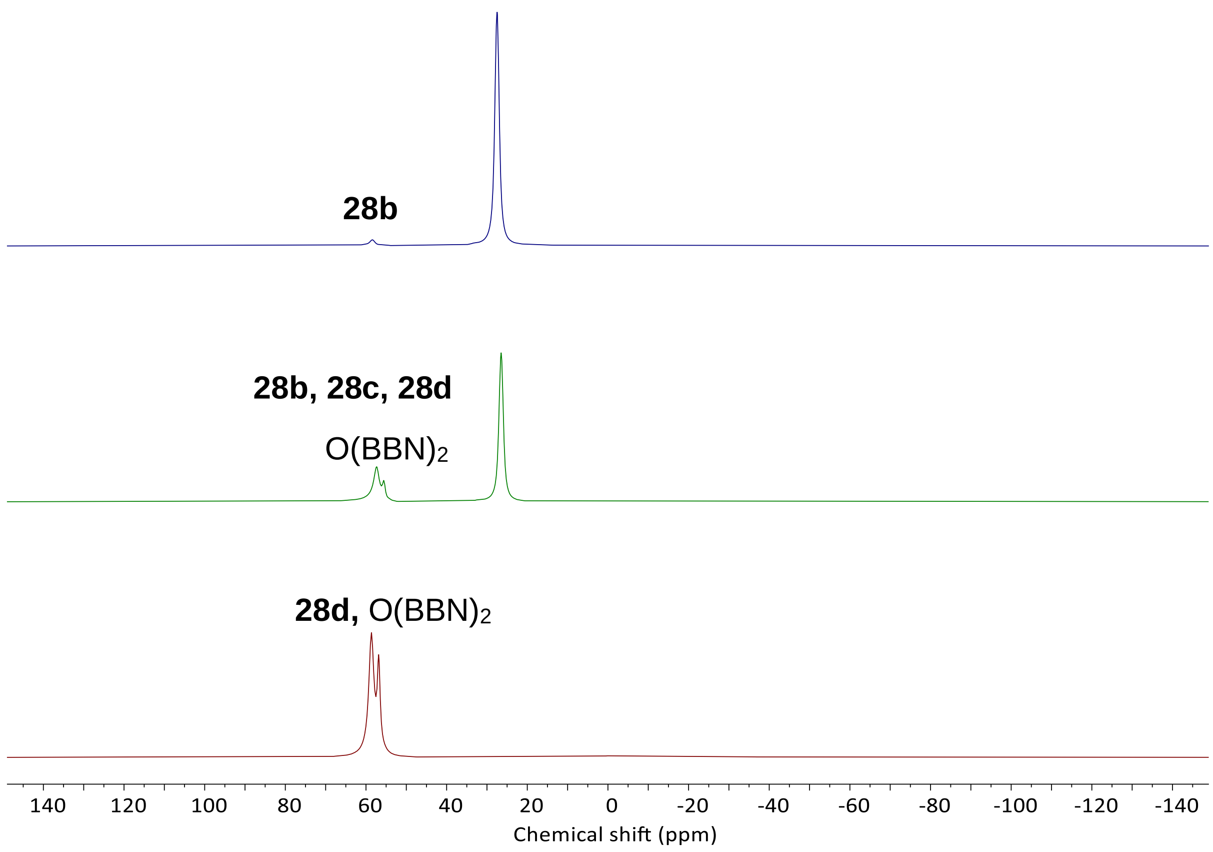

Figure S155. ^{11}B NMR spectrum (reaction mixture) of hydroboration of **28b**. Top spectrum recorded after 0 h; Middle spectrum recorded after 1 h; Bottom spectrum recoded after 18 h.

7. Crystallography Tables

Table S9: Crystallographic data for [Na(18-c-6)]$_2$[1] and [K(18-c-6)]$_2$[1]

Identification code	[Na(18-c-6)]$_2$[**1**]	[K(18-c-6)]$_2$[**1**]
Empirical formula	$C_{40}H_{78}BNa_2O_{14}P_7$	$C_{44.5}H_{78}BK_2O_{12.5}P_7$
Formula weight	1056.60	1118.86
Temperature/K	100.03(13)	150.01(10)
Crystal system	monoclinic	monoclinic
Space group	$P2_1/c$	$P2_1/c$
a/Å	23.8152(11)	14.6032(3)
b/Å	11.3083(4)	22.7779(4)
c/Å	20.3330(8)	17.5915(3)
α/°	90	90
β/°	103.202(4)	90.880(2)
γ/°	90	90
Volume/Å^3	5331.2(4)	5850.77(19)
Z	4	4
ρ_{calc}g/cm^3	1.316	1.270
μ/mm^{-1}	2.805	3.682
F(000)	2248.0	2372.0
Crystal size/mm^3	0.191 × 0.091 × 0.011	0.296 × 0.118 × 0.08
Radiation	Cu Kα (λ = 1.54184)	CuKα (λ = 1.54184)
2Θ range for data collection/°	3.81 to 108.48	6.052 to 154.09
Index ranges	-25 ≤ h ≤ 25, -11 ≤ k ≤ 11, -17 ≤ l ≤ 21	-18 ≤ h ≤ 16, -26 ≤ k ≤ 28, -20 ≤ l ≤ 22
Reflections collected	22802	27127
Independent reflections	6494 [R_{int} = 0.0868, R_{sigma} = 0.0753]	12139 [R_{int} = 0.0462, R_{sigma} = 0.0706]
Data/restraints/parameters	6494/2302/786	12139/0/648
Goodness-of-fit on F^2	1.017	1.040
Final R indexes [I>=2σ (I)]	R_1 = 0.0610, wR_2 = 0.1540	R_1 = 0.0532, wR_2 = 0.1340
Final R indexes [all data]	R_1 = 0.1132, wR_2 = 0.1820	R_1 = 0.0807, wR_2 = 0.1505
Largest diff. peak/hole / e Å^{-3}	0.65/-0.34	0.76/-0.37
CCDC	2195885	2195883

Table S10: Crystallographic data for 15b and 28b’

Identification code	**15b**	**28b’**
Empirical formula	$C_{17}H_{21}BN_2O_3$	$C_{22}H_{40}BNaO_{10}$
Formula weight	312.17	498.34
Temperature/K	100.00(10)	99.98(11)
Crystal system	triclinic	orthorhombic
Space group	P-1	$P2_12_12_1$
a/Å	7.7127(7)	7.7202(2)
b/Å	10.9084(12)	14.2608(5)
c/Å	10.9940(9)	23.9540(9)
α/°	64.075(10)	90
β/°	82.217(7)	90
γ/°	77.994(9)	90
Volume/Å^3	812.64(15)	2637.24(15)
Z	2	4
ρ_{calc}g/cm^3	1.276	1.255
μ/mm^{-1}	0.699	0.944
F(000)	332.0	1072.0
Crystal size/mm^3	0.193 × 0.065 × 0.045	0.05 × 0.02 × 0.01
Radiation	Cu Kα (λ = 1.54184)	Cu Kα (λ = 1.54184)
2Θ range for data collection/°	8.956 to 152.324	7.214 to 151.866
Index ranges	-9 ≤ h ≤ 9, -13 ≤ k ≤ 13, -13 ≤ l ≤ 9	-9 ≤ h ≤ 9, -13 ≤ k ≤ 17, -30 ≤ l ≤ 28
Reflections collected	9007	15130
Independent reflections	3233 [R_{int} = 0.0428, R_{sigma} = 0.0534]	4929 [R_{int} = 0.0362, R_{sigma} = 0.0428]
Data/restraints/parameters	3233/0/212	4929/0/307
Goodness-of-fit on F^2	1.042	1.052
Final R indexes [I>=2σ (I)]	R_1 = 0.0431, wR_2 = 0.1131	R_1 = 0.0369, wR_2 = 0.0823
Final R indexes [all data]	R_1 = 0.0515, wR_2 = 0.1196	R_1 = 0.0476, wR_2 = 0.0871
Largest diff. peak/hole / e Å^{-3}	0.21/-0.20	0.17/-0.22
CCDC	2195882	2195884

8. Density Functional Theory

8.1. Computational methods

All density functional theory calculations were performed using the Gaussian 16 (G16) suite of programmes.[23] The wB97XD functional,[24] which has been used with some success in related applications,[25] was used throughout, along with the def2-TZVP basis set on all atoms.[26] A superfine integration grid was used, and the influence of the solvent was modelled using the SMD model[27] with parameters appropriate to fluorobenzene (a convenient model for *o*DFB). Free energies were computed using the unscaled vibrational frequencies. It is well established that this protocol provides an over-estimate of the entropic contributions, so we report the energies (ΔE) throughout the manuscript, with free energies (ΔG) given in parenthesis.

8.2. Total energies (E and G) and optimized cartesian coordinates (Å) for all stationary points reported in the text.

H_2CO *E* =-114.515537 au *G* =-114.510254 au

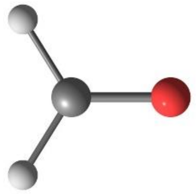

Figure S156. Optimized structure of **H_2CO**.

6	0.526804000	-0.000017000	-0.000017000
8	-0.672846000	0.000050000	0.000004000
1	1.110625000	-0.938489000	0.000035000
1	1.111323000	0.938191000	0.000035000

PhCHO *E* = -345.592415722 au *G* = -345.512225 au

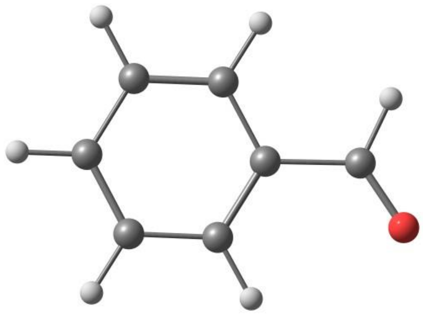

Figure S157. Optimized structure of **PhCHO**.

6	1.983765000	0.460523000	-0.000003000
8	2.838902000	-0.390954000	0.000007000
1	2.259220000	1.533184000	-0.000051000
6	0.530171000	0.199560000	0.000000000
6	0.033032000	-1.102914000	-0.000002000
6	-0.347904000	1.279036000	0.000005000
6	-1.332567000	-1.317746000	-0.000001000
6	-1.717035000	1.062492000	0.000001000
6	-2.206794000	-0.235377000	-0.000002000
1	0.726360000	-1.934775000	-0.000005000
1	0.046402000	2.289349000	0.000006000
1	-1.722595000	-2.327763000	-0.000004000
1	-2.400502000	1.902074000	0.000004000
1	-3.276112000	-0.407886000	0.000003000

H[B] = HB(OCH2CH2O) *E* =-254.623618704 au *G* =-254.572125 au

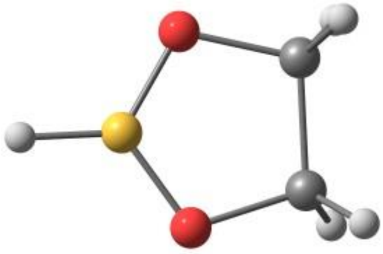

Figure S158. Optimized structure of **H[B]**.

1	2.414189000	-0.000336000	-0.000016000
5	1.224861000	-0.000155000	-0.000012000
8	0.483758000	1.141397000	0.040354000
8	0.483453000	-1.141523000	-0.040334000
6	-0.898576000	0.767633000	-0.044312000
6	-0.898781000	-0.767402000	0.044305000
1	-1.300079000	1.129145000	-0.992176000
1	-1.445625000	1.235132000	0.773923000
1	-1.445931000	-1.234752000	-0.773946000
1	-1.300416000	-1.128799000	0.992159000

MeO[B] *E* =-369.219595601 au *G* =-369.137732 au

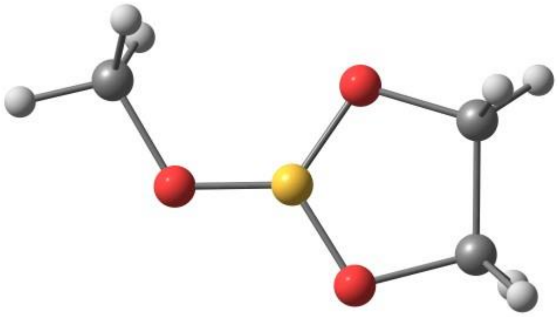

Figure S159. Optimized structure of **MeO[B]**.

6	2.584735000	0.308617000	0.015922000
8	1.555277000	-0.667058000	-0.017945000
1	2.546262000	0.891351000	0.939408000
1	2.514351000	0.988746000	-0.836233000
5	0.261020000	-0.282911000	-0.009407000
8	-0.171308000	1.019788000	0.065584000
8	-0.760004000	-1.197921000	-0.076640000
6	-1.592069000	0.998367000	-0.086993000
6	-1.979431000	-0.476410000	0.098678000
1	-1.845322000	1.365894000	-1.084057000
1	-2.049375000	1.649613000	0.657351000
1	-2.712281000	-0.811748000	-0.634848000
1	-2.366879000	-0.672731000	1.101300000
1	3.537005000	-0.218492000	-0.029520000

MeO-9-BBN *E* =-453.365076795 au *G* = -453.146394 au

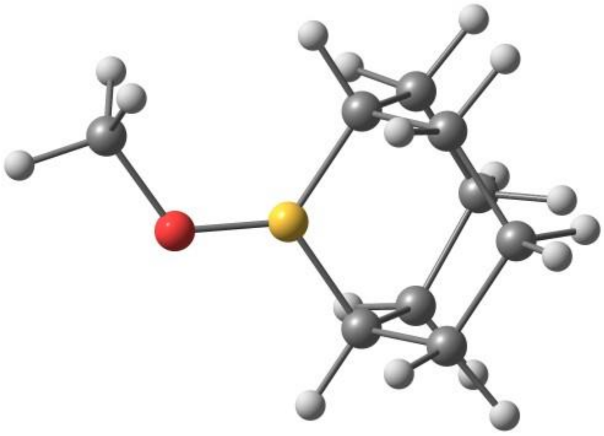

Figure S160. Optimized structure of **MeO-9-BBN**.

6	-0.349981000	-0.001022000	-1.379401000
1	-0.013146000	-0.001659000	-2.420328000
6	-1.147997000	-1.302763000	-1.138707000
1	-2.045686000	-1.313699000	-1.766743000
1	-0.527196000	-2.138600000	-1.481152000
6	-1.552252000	-1.562222000	0.319262000
1	-1.875673000	-2.602759000	0.413255000
1	-2.426385000	-0.962526000	0.569626000
6	-0.438716000	-1.301720000	1.343232000
1	-0.868370000	-1.312973000	2.350928000
1	0.269867000	-2.137308000	1.305741000
6	0.367346000	0.001498000	1.131763000
1	1.183351000	0.002562000	1.859713000

6	-0.439781000	1.304489000	1.340573000
1	-0.868721000	1.317855000	2.348547000
1	0.267970000	2.140680000	1.300496000
6	-1.554277000	1.561266000	0.316759000
1	-1.879284000	2.601474000	0.408890000
1	-2.427306000	0.960690000	0.568857000
6	-1.150377000	1.299565000	-1.140913000
1	-0.531357000	2.135896000	-1.485351000
1	-2.048422000	1.307530000	-1.768489000
5	0.852921000	0.000316000	-0.366983000
8	2.134354000	-0.000018000	-0.812622000
6	3.237959000	0.000700000	0.078829000
1	3.235913000	-0.888569000	0.713397000
1	4.147533000	0.000189000	-0.521796000
1	3.235934000	0.891030000	0.711922000

1 E = -2728.01768724 au G = -2727.836340 au

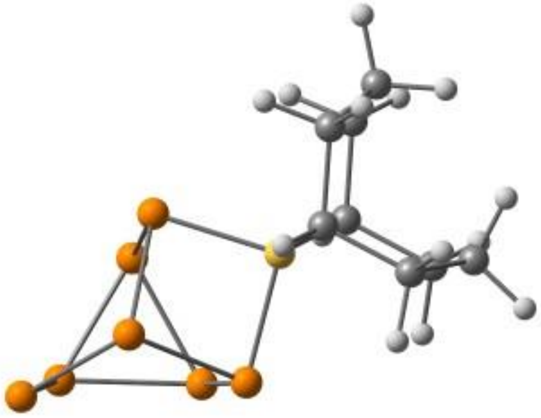

Figure S161. Optimized structure of [**1**]$^{2-}$

15	3.351642000	0.815324000	0.000064000
15	1.539444000	1.449172000	1.122458000
15	1.539231000	1.449577000	-1.122060000
15	0.419321000	-0.392667000	-1.498413000
15	1.353350000	-1.717249000	-0.000214000
15	0.419787000	-0.393171000	1.498676000
15	3.450650000	-1.330421000	-0.000410000
6	-2.113002000	-1.343515000	-0.000202000
1	-1.661846000	-2.343079000	-0.000412000
6	-2.953081000	-1.245408000	1.287229000
1	-3.749984000	-2.001590000	1.281753000
1	-2.299358000	-1.498956000	2.128185000
6	-3.585455000	0.127115000	1.560546000
1	-3.914479000	0.163686000	2.604657000
1	-4.494584000	0.232214000	0.966494000
6	-2.658786000	1.320839000	1.289076000
1	-3.261114000	2.239497000	1.290076000
1	-1.961451000	1.416357000	2.128955000
6	-1.822370000	1.227637000	0.000361000
1	-1.152109000	2.095097000	0.000621000
6	-2.658473000	1.321464000	-1.288557000
1	-3.260902000	2.240036000	-1.289221000
1	-1.960886000	1.417376000	-2.128164000

6	-3.585033000	0.127750000	-1.560827000
1	-3.913647000	0.164770000	-2.605059000
1	-4.494384000	0.232763000	-0.967096000
6	-2.952857000	-1.244871000	-1.287769000
1	-2.298968000	-1.498296000	-2.128640000
1	-3.749776000	-2.001041000	-1.282664000
5	-0.990708000	-0.162674000	-0.000027000

1b *E* = -2727.99318489 au *G* = -2727.811888 au (conformer with only one P bound to B)

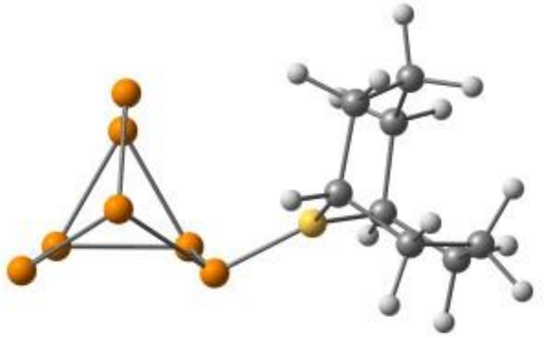

Figure S162. Optimized structure of the unbridged isomer of [**1**]$^{2-}$, [**1'**]$^{2-}$

15	3.382851000	-0.829675000	-0.630773000
15	1.286636000	-1.576676000	-0.873692000
15	2.164795000	-1.319000000	1.188467000
15	1.451899000	0.538675000	1.968768000
15	1.433056000	1.684271000	0.123420000
15	0.282745000	0.330378000	-1.184483000
15	3.355084000	1.311823000	-0.820047000
6	-2.325862000	1.304188000	0.084096000
1	-1.785811000	2.254878000	0.104644000
6	-3.550077000	1.485478000	-0.838689000
1	-4.190032000	2.286053000	-0.449306000
1	-3.186756000	1.831559000	-1.813249000
6	-4.391725000	0.223143000	-1.047441000
1	-5.094771000	0.396915000	-1.867972000
1	-5.007556000	0.047710000	-0.165484000
6	-3.562992000	-1.028204000	-1.363710000
1	-4.202107000	-1.915568000	-1.281744000
1	-3.248097000	-0.975706000	-2.411939000
6	-2.288359000	-1.221766000	-0.514819000
1	-1.734969000	-2.060062000	-0.945898000
6	-2.535481000	-1.558884000	0.975091000
1	-3.106876000	-2.491110000	1.052297000
1	-1.560074000	-1.754976000	1.434533000
6	-3.240800000	-0.460318000	1.778669000
1	-3.157232000	-0.698977000	2.843038000
1	-4.308317000	-0.474070000	1.556407000
6	-2.670329000	0.946599000	1.549309000
1	-1.741336000	1.033811000	2.119237000
1	-3.363155000	1.691698000	1.958048000
5	-1.455030000	0.117897000	-0.488510000

P7-[B] *E* = -2643.84640418 au *G* = -2643.803645 au

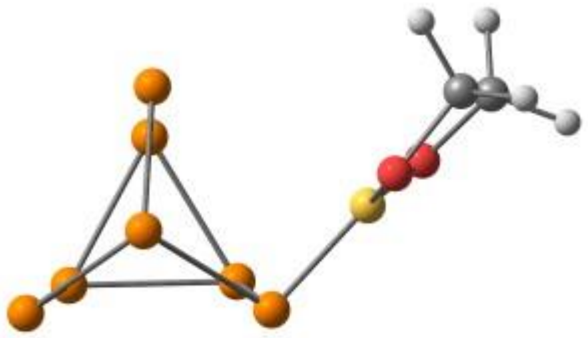

Figure S163. Optimized structure of **P_7-[B].**

15	7.350864000	9.097614000	4.330148000
15	5.260659000	9.773230000	4.884015000
15	5.553837000	8.219486000	3.248849000
15	5.198928000	6.297524000	4.124812000
15	6.061810000	6.658290000	6.096104000
15	4.939012000	8.532480000	6.669181000
15	7.980243000	7.622603000	5.758437000
6	1.077582000	7.719669000	5.433019000
6	1.409363000	6.473145000	6.253546000
8	2.295552000	8.460468000	5.381903000
8	2.660950000	6.762526000	6.869235000
5	3.191433000	7.866499000	6.239103000
1	0.296680000	8.340646000	5.901086000
1	1.520070000	5.576330000	5.621151000
1	0.657415000	6.247477000	7.022994000
1	0.745018000	7.483371000	4.411864000

I1 *E* = -2982.64783594 au *G* = -2982.394835 au

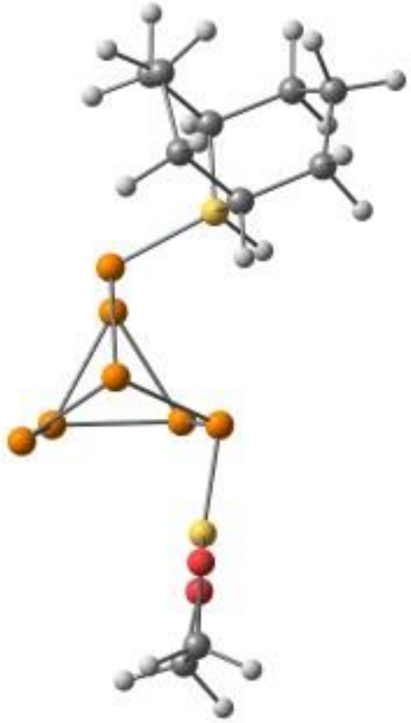

Figure S164. Optimized structure of **I1.**

15	2.005507000	2.488087000	-0.008994000
15	1.576405000	1.107084000	1.702150000
15	-0.061956000	2.286902000	0.768459000
15	-1.018941000	0.998027000	-0.706990000
15	0.694007000	-0.217971000	-1.297620000
15	1.303554000	-0.836766000	0.738802000
15	2.146297000	1.290546000	-1.794758000
6	-2.698124000	-1.513990000	-0.348461000
1	-1.818477000	-2.118491000	-0.603896000
6	-3.532385000	-2.357596000	0.633272000
1	-3.879231000	-3.285652000	0.155902000
1	-2.863081000	-2.663021000	1.445207000
6	-4.748477000	-1.647659000	1.249335000
1	-5.093629000	-2.222813000	2.115899000
1	-5.578008000	-1.670451000	0.540580000
6	-4.495482000	-0.196075000	1.684279000
1	-5.466487000	0.273294000	1.899857000
1	-3.944358000	-0.208509000	2.631267000
6	-3.683084000	0.651973000	0.689172000
1	-3.514890000	1.623707000	1.173075000
6	-4.430847000	0.938595000	-0.624228000
1	-5.413567000	1.388273000	-0.421616000
1	-3.859529000	1.695906000	-1.171386000
6	-4.634933000	-0.271190000	-1.550641000
1	-4.902410000	0.090398000	-2.549645000
1	-5.498698000	-0.845977000	-1.211512000
6	-3.421587000	-1.206170000	-1.669438000
1	-2.693847000	-0.745203000	-2.347351000
1	-3.746910000	-2.133410000	-2.162344000
5	-2.278576000	-0.138729000	0.420921000
6	5.402849000	-1.305689000	0.435183000
6	4.898512000	-2.458754000	-0.442765000
8	4.223991000	-0.755843000	1.025568000
8	3.484524000	-2.272571000	-0.512039000
5	3.139411000	-1.277239000	0.367893000
1	5.895241000	-0.527679000	-0.151944000
1	5.103803000	-3.436883000	-0.000782000
1	5.316415000	-2.430449000	-1.449528000
1	6.079581000	-1.639255000	1.222641000
1	-1.705958000	-0.375950000	1.483306000

I2 *E*= -2842.58616167 au *G* = -2842.373685 au

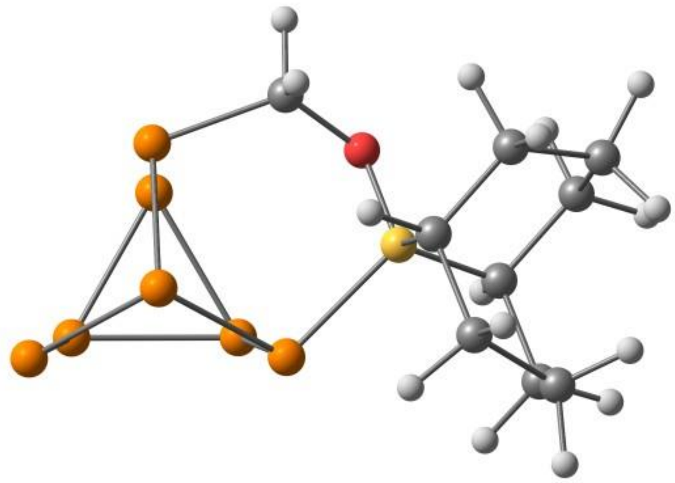

Figure S165. Optimized structure of **I2.**

15	3.501672000	-0.569405000	0.906525000
15	1.432195000	-0.646514000	1.700607000
15	2.349177000	1.311746000	1.179540000
15	1.684801000	1.661961000	-0.881983000
15	1.450205000	-0.388022000	-1.617058000
15	0.227667000	-1.220535000	-0.016596000
15	3.430088000	-1.107749000	-1.192880000
6	-2.275456000	0.061370000	-1.237413000
1	-1.699619000	0.285440000	-2.145222000
6	-2.834632000	-1.358796000	-1.421643000
1	-3.510965000	-1.395309000	-2.287188000
1	-1.996484000	-2.018536000	-1.668636000
6	-3.571270000	-1.946360000	-0.207419000
1	-3.667413000	-3.028914000	-0.344012000
1	-4.594404000	-1.566857000	-0.187656000
6	-2.901711000	-1.675523000	1.149179000
1	-3.620112000	-1.918512000	1.944616000
1	-2.069905000	-2.377282000	1.268506000
6	-2.348572000	-0.254903000	1.337147000
1	-1.809186000	-0.243878000	2.294074000
6	-3.443722000	0.824048000	1.436751000
1	-4.159977000	0.576906000	2.233271000
1	-2.955920000	1.755832000	1.737007000
6	-4.228474000	1.082780000	0.141152000
1	-4.777768000	2.025692000	0.241637000
1	-4.994537000	0.315095000	0.021165000
6	-3.367361000	1.143415000	-1.130093000
1	-2.873196000	2.119997000	-1.162190000
1	-4.033037000	1.108608000	-2.004096000
5	-1.358810000	0.162573000	0.113817000
6	-0.095371000	2.196732000	-0.559493000
8	-0.823347000	1.506413000	0.384600000
1	-0.030296000	3.247125000	-0.252347000
1	-0.564008000	2.191619000	-1.556120000

I3 *E*= -3097.22934912 au *G* = -3096.938901 au

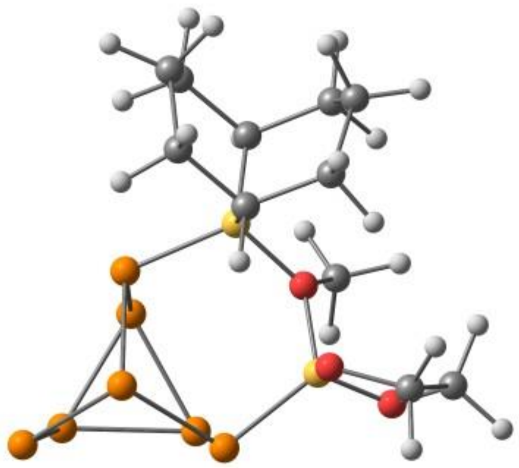

Figure S166. Optimized structure of **I3**.

15	-3.816448000	-0.931382000	0.621960000
15	-2.615723000	0.800434000	1.335078000
15	-1.817616000	-1.276340000	1.534195000
15	-0.566226000	-1.584470000	-0.219405000
15	-1.582895000	-0.304837000	-1.665212000
15	-1.742664000	1.553778000	-0.519331000
15	-3.602246000	-1.039059000	-1.522178000
6	2.105323000	-0.536907000	-1.166431000
1	1.595751000	0.033430000	-1.944962000
6	3.399419000	0.231593000	-0.843568000
1	4.027580000	0.308679000	-1.741513000
1	3.111020000	1.252839000	-0.583178000
6	4.243397000	-0.347332000	0.297788000
1	4.991684000	0.393394000	0.600815000
1	4.814082000	-1.202946000	-0.064988000
6	3.426643000	-0.760161000	1.529245000
1	4.060371000	-1.372398000	2.184530000
1	3.192759000	0.141849000	2.101615000
6	2.111229000	-1.505034000	1.225374000
1	1.613286000	-1.674339000	2.187575000
6	2.356013000	-2.915099000	0.651812000
1	3.015855000	-3.475704000	1.326993000
1	1.400858000	-3.446663000	0.651378000
6	2.945979000	-2.969819000	-0.764774000
1	2.792656000	-3.975082000	-1.170772000
1	4.028262000	-2.842730000	-0.710478000
6	2.359252000	-1.941752000	-1.742710000
1	1.406753000	-2.325340000	-2.120996000
1	3.022048000	-1.871880000	-2.615534000
5	1.199756000	-0.641181000	0.180467000
6	1.507811000	3.491504000	-1.249503000
6	1.436165000	3.871480000	0.225556000
8	0.910947000	2.218883000	-1.312077000
8	0.289052000	3.198326000	0.690045000
5	0.185316000	1.990386000	-0.093228000
1	2.535727000	3.449688000	-1.624000000
1	1.331614000	4.947122000	0.387606000
1	2.334051000	3.526740000	0.760691000
1	0.944267000	4.203678000	-1.867140000
8	0.888383000	0.819508000	0.738130000

6	0.782828000	1.016010000	2.146792000
1	1.578913000	1.679680000	2.489271000
1	-0.174438000	1.469119000	2.395899000
1	0.857400000	0.062387000	2.658117000

I4 *E* =-3211.78592097 au *G* =-3211.468015 au

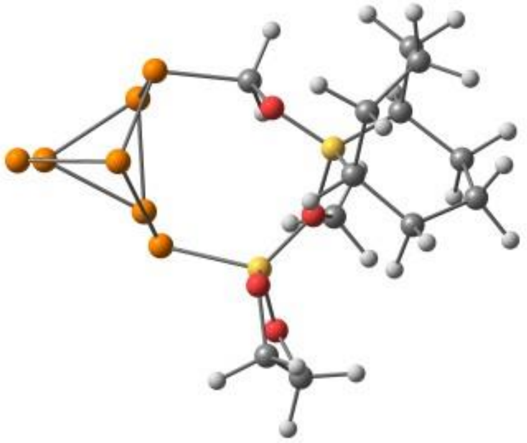

Figure S167. Optimized structure of **I4**.

15	-4.528077000	-0.257640000	0.471048000
15	-2.788175000	0.715920000	1.471791000
15	-2.954034000	-1.515636000	1.396751000
15	-1.802614000	-2.078690000	-0.380398000
15	-1.990785000	-0.271653000	-1.620690000
15	-1.638135000	1.374231000	-0.247150000
15	-4.149680000	-0.238189000	-1.662663000
6	2.886401000	-0.133092000	-1.166938000
1	2.312427000	0.413211000	-1.916041000
6	3.947882000	0.826093000	-0.614220000
1	4.621487000	1.164842000	-1.413346000
1	3.423206000	1.718127000	-0.257262000
6	4.797776000	0.261199000	0.530882000
1	5.346824000	1.080109000	1.008581000
1	5.563552000	-0.398336000	0.120528000
6	3.995344000	-0.487154000	1.607097000
1	4.695849000	-1.059610000	2.230249000
1	3.550171000	0.258339000	2.272463000
6	2.870327000	-1.402232000	1.089349000
1	2.329476000	-1.774872000	1.968423000
6	3.388945000	-2.664351000	0.369988000
1	4.039039000	-3.242253000	1.040312000
1	2.524843000	-3.301417000	0.154509000
6	4.139574000	-2.407346000	-0.942272000
1	4.246019000	-3.355380000	-1.480570000
1	5.157853000	-2.085811000	-0.718252000
6	3.460379000	-1.383034000	-1.862365000
1	2.626398000	-1.877945000	-2.368783000
1	4.170156000	-1.092426000	-2.649001000
5	1.915821000	-0.636319000	0.015593000
6	1.151658000	3.480369000	-1.248105000
6	1.252769000	3.892241000	0.215624000

8	0.911577000	2.095781000	-1.200805000
8	0.398513000	2.991500000	0.879934000
5	0.362644000	1.778232000	0.084394000
1	2.067622000	3.683049000	-1.811251000
1	0.926980000	4.920786000	0.392761000
1	2.286432000	3.789893000	0.578804000
1	0.317394000	3.997195000	-1.742129000
8	1.186893000	0.689858000	0.778010000
6	1.008883000	0.715044000	2.189693000
1	1.589376000	1.526320000	2.630588000
1	-0.044321000	0.872360000	2.433611000
1	1.322561000	-0.231181000	2.616843000
8	0.812443000	-1.405207000	-0.513239000
6	-0.096044000	-1.927519000	0.398029000
1	-0.187997000	-1.303346000	1.290137000
1	0.196059000	-2.933139000	0.736809000

I5 *E* =-3351.85418639 au *G* = -3351.493889 au

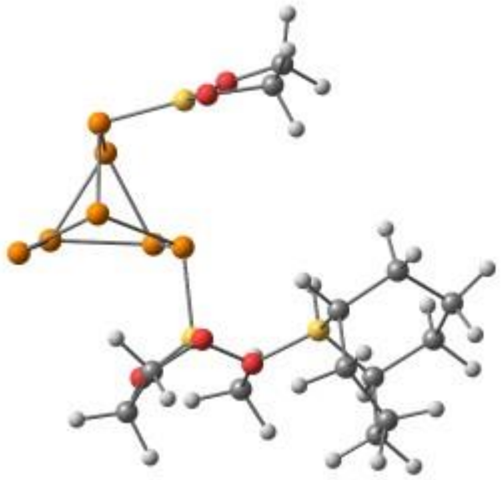

Figure S168. Optimized structure of **I5**.

15	3.440553000	-1.692418000	1.481276000
15	1.622943000	-0.467902000	1.893003000
15	3.592034000	0.529862000	1.620741000
15	3.656611000	1.031238000	-0.525079000
15	2.366182000	-0.542601000	-1.368963000
15	0.687335000	-0.091101000	-0.030354000
15	3.427885000	-2.244060000	-0.591769000
6	-3.016933000	0.789843000	-0.705883000
1	-2.131546000	0.880603000	-1.343015000
6	-4.023920000	-0.085224000	-1.472913000
1	-4.340384000	0.413262000	-2.400417000
1	-3.482086000	-0.983266000	-1.777820000
6	-5.279856000	-0.509730000	-0.697221000
1	-5.759819000	-1.339932000	-1.227520000
1	-6.013817000	0.297987000	-0.701904000
6	-5.007714000	-0.934226000	0.750739000
1	-5.968115000	-1.041058000	1.274210000
1	-4.557980000	-1.934675000	0.736296000
6	-4.058683000	-0.006438000	1.524295000
1	-3.930734000	-0.441112000	2.523120000

6	-4.608851000	1.413033000	1.762743000
1	-5.591702000	1.370389000	2.252267000
1	-3.935524000	1.902734000	2.475129000
6	-4.719338000	2.309667000	0.520051000
1	-4.842908000	3.348748000	0.844843000
1	-5.631883000	2.067456000	-0.026612000
6	-3.518833000	2.218837000	-0.431528000
1	-2.687641000	2.786461000	0.002172000
1	-3.779408000	2.724621000	-1.371404000
5	-2.649096000	0.151083000	0.732278000
6	-0.806890000	-2.571505000	-2.425726000
6	-0.666434000	-3.626679000	-1.335437000
8	-1.161912000	-1.400492000	-1.730501000
8	-0.268163000	-2.892850000	-0.202913000
5	-0.715882000	-1.539672000	-0.370160000
1	-1.577280000	-2.820233000	-3.161888000
1	0.085665000	-4.383628000	-1.572809000
1	-1.625997000	-4.132396000	-1.152277000
1	0.145364000	-2.427947000	-2.953831000
8	-1.909854000	-1.266716000	0.609920000
6	-1.787583000	-1.897294000	1.882593000
1	-1.066505000	-2.706224000	1.813700000
1	-1.461939000	-1.171743000	2.632157000
1	-2.754848000	-2.306359000	2.174266000
1	-1.851813000	0.844828000	1.364510000
6	0.871508000	3.909005000	-1.436063000
6	1.242601000	4.357205000	-0.019836000
8	1.853942000	2.925024000	-1.765704000
8	2.099207000	3.321869000	0.470440000
5	2.420259000	2.509817000	-0.585767000
1	0.909071000	4.720322000	-2.163278000
1	0.373725000	4.444667000	0.631896000
1	1.792953000	5.301766000	-0.013176000
1	-0.114604000	3.439696000	-1.468035000

I6 *E* =-3466.42638344 au *G* = -3466.027844 au

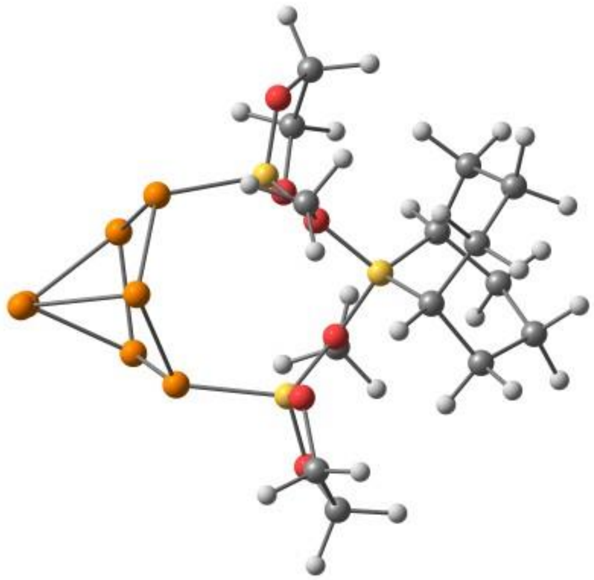

Figure S169. Optimized structure of **I6**.

15	-4.641400000	-0.105259000	0.614243000
15	-2.832028000	0.639401000	1.699056000
15	-2.953137000	-1.485231000	1.022088000
15	-1.982003000	-1.564830000	-0.919505000
15	-2.314389000	0.492455000	-1.608235000
15	-1.857047000	1.750441000	0.109672000
15	-4.468562000	0.536302000	-1.444849000
6	3.005982000	0.824626000	-0.939044000
1	2.483070000	1.500546000	-1.612884000
6	3.758969000	1.714679000	0.068573000
1	4.391114000	2.433646000	-0.467217000
1	3.025942000	2.304337000	0.623993000
6	4.629009000	0.939737000	1.060424000
1	4.970232000	1.619152000	1.848264000
1	5.534825000	0.607056000	0.551925000
6	3.916961000	-0.258512000	1.710432000
1	4.674292000	-0.942707000	2.114542000
1	3.364418000	0.108790000	2.577744000
6	2.935227000	-1.046697000	0.816707000
1	2.367309000	-1.703892000	1.475503000
6	3.660647000	-1.968182000	-0.183771000
1	4.226522000	-2.735695000	0.358683000
1	2.906013000	-2.500839000	-0.769499000
6	4.607333000	-1.242828000	-1.139221000
1	4.935164000	-1.938627000	-1.918407000
1	5.512737000	-0.964129000	-0.598588000
6	3.981150000	-0.006047000	-1.801905000
1	3.453406000	-0.332926000	-2.699443000
1	4.786241000	0.647095000	-2.162710000
5	1.973830000	-0.074132000	-0.057965000
6	0.809274000	3.860468000	-0.965920000
6	0.992656000	4.199394000	0.516326000
8	0.589500000	2.471726000	-0.999964000
8	0.369529000	3.145881000	1.211070000
5	0.160626000	2.046666000	0.294769000
1	1.691090000	4.113246000	-1.563076000
1	0.525377000	5.152911000	0.780000000
1	2.056276000	4.260190000	0.781769000
1	-0.055808000	4.388056000	-1.387077000
8	1.014989000	0.832898000	0.792229000
6	0.853183000	0.617012000	2.195160000
1	1.492387000	1.303045000	2.750777000
1	-0.185375000	0.807124000	2.462647000
1	1.078271000	-0.415520000	2.427730000
8	0.935316000	-0.900222000	-0.895835000
6	0.932557000	-0.828750000	-2.318259000
1	1.113785000	0.195596000	-2.631213000
1	-0.043521000	-1.135167000	-2.687503000
1	1.684073000	-1.501953000	-2.731906000
6	0.239019000	-3.416452000	1.447878000
6	0.762825000	-4.092205000	0.182396000
8	0.094889000	-2.065020000	1.091906000
8	0.292226000	-3.281009000	-0.868244000
5	-0.050528000	-1.980905000	-0.327088000
1	0.927827000	-3.511408000	2.293035000
1	0.385690000	-5.112383000	0.067531000
1	1.859601000	-4.131985000	0.184520000

1	-0.732438000	-3.832388000	1.747307000

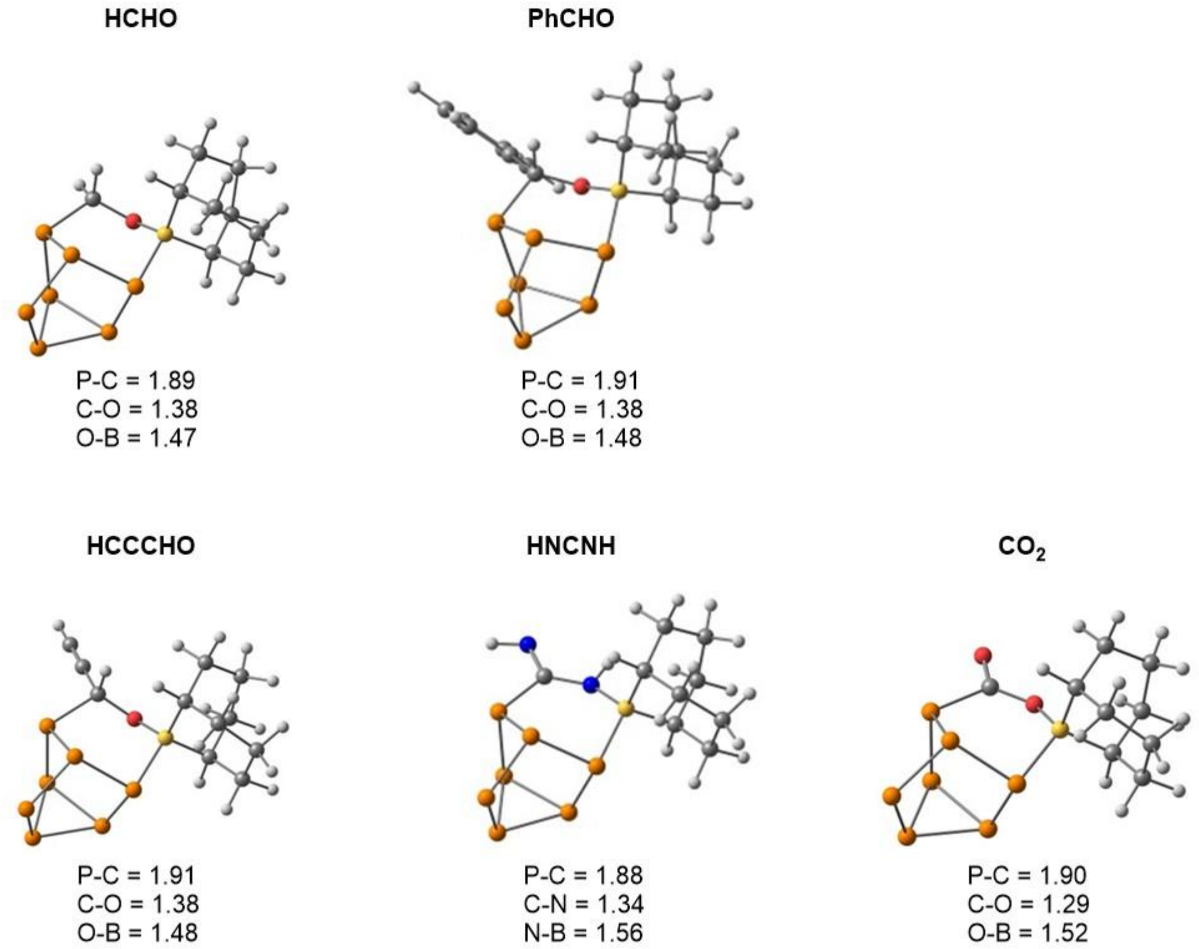

Figure S170 Optimized structures of analogues of **I2** with different substrates (HCHO, PhCHO, HC≡CCHO, HNCNH and CO_2. All bond lengths are given in Å.

Optimized coordinates of **I2** with PhCHO as substrate.

E = -3073.65076473 au *G* = -3073.362917 au

15	3.668877000	0.958850000	0.951425000
15	2.059565000	-0.351657000	1.729564000
15	1.607204000	1.758501000	1.185886000
15	0.927046000	1.628625000	-0.896227000
15	1.974730000	-0.165113000	-1.592689000
15	1.480437000	-1.553159000	0.010430000
15	3.976065000	0.464345000	-1.137826000
6	-1.254245000	-2.052365000	-1.268652000
1	-0.907692000	-1.544586000	-2.178451000
6	-0.842236000	-3.525798000	-1.418318000

1	-1.336805000	-3.974142000	-2.291237000
1	0.230602000	-3.551928000	-1.635417000
6	-1.114262000	-4.419080000	-0.197519000
1	-0.537777000	-5.344384000	-0.303069000
1	-2.160520000	-4.728947000	-0.204929000
6	-0.783034000	-3.780661000	1.161054000
1	-1.242670000	-4.389926000	1.951686000
1	0.297989000	-3.847765000	1.319002000
6	-1.189433000	-2.306871000	1.312129000
1	-0.786478000	-1.959080000	2.272673000
6	-2.713404000	-2.089723000	1.369863000
1	-3.160814000	-2.693778000	2.171733000
1	-2.883822000	-1.042901000	1.636979000
6	-3.464284000	-2.381551000	0.062175000
1	-4.470906000	-1.953965000	0.128345000
1	-3.613613000	-3.457751000	-0.039111000
6	-2.779332000	-1.840472000	-1.202104000
1	-2.970618000	-0.763606000	-1.258665000
1	-3.268651000	-2.282901000	-2.081098000
5	-0.610329000	-1.405052000	0.087491000
6	-0.852387000	0.976461000	-0.622123000
8	-0.991229000	-0.001946000	0.331473000
1	-1.123486000	0.617035000	-1.625843000
6	-1.745026000	2.154388000	-0.303300000
6	-2.228196000	2.352564000	0.985352000
6	-2.093488000	3.069167000	-1.295214000
6	-3.035403000	3.444033000	1.277438000
6	-2.901654000	4.156924000	-1.006779000
6	-3.375984000	4.351004000	0.284756000
1	-1.963852000	1.638277000	1.753514000
1	-1.723952000	2.924625000	-2.304972000
1	-3.399321000	3.585310000	2.288584000
1	-3.163819000	4.855516000	-1.792772000
1	-4.006946000	5.201737000	0.512820000

Optimized coordinates of **I2** with HC≡CCHO as substrate.

E = -2918.72904575 au *G* = -2918.509684 au

15	3.506355000	-0.819982000	0.903300000
15	1.439286000	-0.924552000	1.695934000
15	2.339968000	1.048992000	1.206146000
15	1.705232000	1.410511000	-0.863968000
15	1.451575000	-0.631047000	-1.619808000
15	0.238783000	-1.488529000	-0.027645000
15	3.438393000	-1.331288000	-1.204421000
6	-2.266736000	-0.208607000	-1.235654000
1	-1.694271000	0.019730000	-2.144696000
6	-2.824636000	-1.628836000	-1.427263000
1	-3.501262000	-1.659858000	-2.292389000
1	-1.986125000	-2.286205000	-1.678997000
6	-3.559856000	-2.224131000	-0.215965000
1	-3.655278000	-3.305748000	-0.359447000
1	-4.582990000	-1.845256000	-0.192866000
6	-2.889046000	-1.961461000	1.141503000
1	-3.606393000	-2.208110000	1.936384000
1	-2.057052000	-2.663717000	1.256442000
6	-2.336017000	-0.541566000	1.336780000

1	-1.795543000	-0.535676000	2.292756000
6	-3.430045000	0.538086000	1.443429000
1	-4.143831000	0.286857000	2.240480000
1	-2.941563000	1.468301000	1.747065000
6	-4.217964000	0.803458000	0.151376000
1	-4.766624000	1.745698000	0.258222000
1	-4.983777000	0.036081000	0.028395000
6	-3.359446000	0.871921000	-1.120832000
1	-2.869467000	1.850785000	-1.149819000
1	-4.025505000	0.839348000	-1.994228000
5	-1.348537000	-0.123885000	0.113995000
6	-0.111094000	1.928640000	-0.554670000
8	-0.810112000	1.224622000	0.400212000
1	-0.555084000	1.829025000	-1.556556000
6	-0.114376000	3.355302000	-0.218690000
6	-0.108916000	4.522572000	0.062787000
1	-0.096735000	5.557360000	0.317719000

Optimized coordinates of **I2** with HNCNH as substrate.

E = -2876.86557064 au *G* = -2876.646999 au

15	3.480293000	-0.892550000	0.816559000
15	1.440243000	-0.854609000	1.685776000
15	2.502202000	1.050706000	1.240763000
15	1.774062000	1.567743000	-0.771952000
15	1.368221000	-0.407617000	-1.626042000
15	0.161876000	-1.248086000	-0.032574000
15	3.310786000	-1.266669000	-1.313853000
6	-2.314260000	-0.090315000	-1.256865000
1	-1.744982000	0.128595000	-2.169999000
6	-2.827803000	-1.533996000	-1.387911000
1	-3.519231000	-1.619216000	-2.237651000
1	-1.974167000	-2.173499000	-1.632093000
6	-3.522823000	-2.105919000	-0.140735000
1	-3.566842000	-3.196514000	-0.229793000
1	-4.562927000	-1.775984000	-0.125154000
6	-2.860639000	-1.746816000	1.199373000
1	-3.568718000	-1.979961000	2.006518000
1	-2.003050000	-2.409140000	1.350953000
6	-2.370079000	-0.295066000	1.324624000
1	-1.839615000	-0.219842000	2.283905000
6	-3.537404000	0.710987000	1.385784000
1	-4.247164000	0.418745000	2.171158000
1	-3.147098000	1.683193000	1.706929000
6	-4.316510000	0.899160000	0.073027000
1	-4.904217000	1.821146000	0.138189000
1	-5.048217000	0.096488000	-0.026659000
6	-3.449683000	0.948680000	-1.194964000
1	-3.001897000	1.944527000	-1.277675000
1	-4.109371000	0.844983000	-2.067208000
5	-1.374992000	0.090015000	0.079947000
6	0.146338000	2.300434000	-0.193580000
7	-0.873951000	1.552643000	0.249942000
7	0.088059000	3.597984000	-0.142323000
1	-1.589775000	2.175566000	0.604434000
1	0.955412000	3.999789000	-0.481581000

Optimized coordinates of **I2** with CO_2 as substrate.

E = -2916.64233921 au *G* = -2916.450160 au

15	-3.517041000	-0.738763000	-0.834390000
15	-1.488460000	-0.736497000	-1.733528000
15	-2.454150000	1.183308000	-1.143193000
15	-1.678690000	1.531219000	0.890907000
15	-1.355165000	-0.519111000	1.597123000
15	-0.206103000	-1.305746000	-0.065077000
15	-3.342204000	-1.256237000	1.262023000
6	2.254689000	-0.131702000	1.242179000
1	1.658479000	0.055103000	2.144801000
6	2.809970000	-1.560683000	1.361252000
1	3.473800000	-1.640307000	2.232567000
1	1.969991000	-2.233498000	1.562276000
6	3.565068000	-2.077315000	0.125982000
1	3.661939000	-3.165430000	0.201545000
1	4.586549000	-1.694283000	0.142479000
6	2.914750000	-1.731710000	-1.223370000
1	3.645241000	-1.925753000	-2.020231000
1	2.087933000	-2.427911000	-1.396224000
6	2.361762000	-0.301678000	-1.342625000
1	1.837011000	-0.241242000	-2.305330000
6	3.460677000	0.777866000	-1.369911000
1	4.185336000	0.563873000	-2.167359000
1	2.985724000	1.727053000	-1.631618000
6	4.227092000	0.965599000	-0.051263000
1	4.771968000	1.914977000	-0.091125000
1	4.994979000	0.195212000	0.034737000
6	3.349827000	0.951777000	1.210097000
1	2.856232000	1.923897000	1.299866000
1	4.002855000	0.856625000	2.088307000
5	1.353006000	0.015546000	-0.107768000
6	-0.026861000	2.206508000	0.235344000
8	0.898414000	1.459557000	-0.266960000
8	0.056873000	3.421616000	0.230718000

The optimized structures (in all cases confirmed to be local minima by the absence of imaginary frequencies) confirm that the geometry of the adduct **I2** is largely independent of the identity of the substrate. In all cases there is a significant elongation of the C=O or C=N bond upon binding, and a short P–C bond of around 1.90 Å.

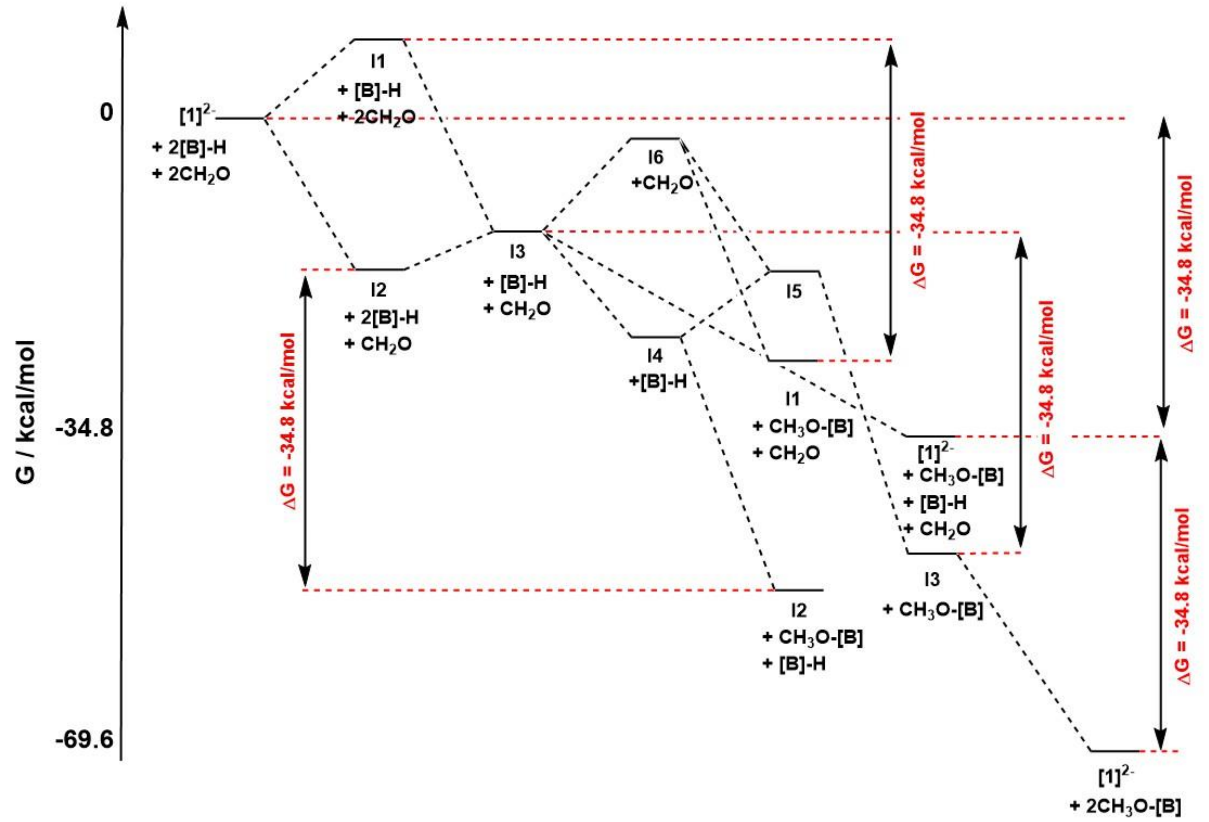

Figure S171 Alternative representation of the energetics of the catalytic reaction in the form of a free-energy surface. Note that the overall reaction (top to bottom) corresponds to the consumption of two moles of reactant and the formation of two moles of product. Also note that several of the intermediates (**I1**-**I3**) appear multiple times in the figure, in combination with different amounts of reactants and products. The vertical arrows, all of which correspond to the free energy of the overall reaction (CH_2O + [B]H → CH_3O[B]), are shown to highlight the many different possible cycles.

9. References

1. Cicač-Hudi, M.; Bender, J.; Schlindwein, S. H.; Bispinghoff, M.; Nieger, M.; Grützmacher, H.; Gudat, D., Direct Access to Inversely Polarized Phosphaalkenes from Elemental Phosphorus or Polyphosphides. *Eur. J. Inorg. Chem.* **2016,** *2016* (5), 649–658.
2. Turbervill, R. S. P.; Goicoechea, J. M., Studies on the Reactivity of Group 15 Zintl Ions with Carbodiimides: Synthesis and Characterization of a Heptaphosphaguanidine Dianion. *Chem. Commun.* **2012,** *48* (10), 1470–1472.
3. Sheldrick, G. M., *Acta. Cryst.* **2015,** *A71*, 3–8.
4. Dolomanov, O. V.; Bourhis, L. J.; Gildea, R. J.; Howard, J. A. K.; Puschmann, H., *J. Appl. Cryst.* **2009,** *42*, 339–341.
5. Khononov, M.; Fridman, N.; Tamm, M.; Eisen, M. S., Hydroboration of Aldehydes, Ketones, and Carbodiimides Promoted by Mono(imidazolin-2-iminato) Hafnium Complexes. *Eur. J. Org. Chem.* **2020,** *2020* (21), 3153–3160.
6. Du, Z.; Behera, B.; Kumar, A.; Ding, Y., Super Hydride Catalyzed Ester and Isocyanate Hydroboration. *J. Organomet. Chem.* **2021,** *950*, 121982.
7. Gudun, K. A.; Tussupbayev, S.; Slamova, A.; Khalimon, A. Y., Hydroboration of Isocyanates: Cobalt-Catalyzed vs. Catalyst-Free Approaches. *Org. Biomol. Chem.* **2022,** *20* (34), 6821–6830.
8. Sahoo, R. K.; Sarkar, N.; Nembenna, S., Zinc Hydride Catalyzed Chemoselective Hydroboration of Isocyanates: Amide Bond Formation and C=O Bond Cleavage. *Angew. Chem. Int. Ed.* **2021,** *60* (21), 11991–12000.
9. Chandra Sau, S.; Bhattacharjee, R.; Hota, P. K.; Vardhanapu, P. K.; Vijaykumar, G.; Govindarajan, R.; Datta, A.; Mandal, S. K., Transforming Atmospheric CO_2 into Alternative Fuels: A Metal-Free Approach Under Ambient Conditions. *Chem. Sci.* **2019,** *10* (6), 1879–1884.
10. Blondiaux, E.; Pouessel, J.; Cantat, T., Carbon Dioxide Reduction to Methylamines under Metal-Free Conditions. *Angew. Chem. Int. Ed.* **2014,** *53* (45), 12186–12190.
11. Courtemanche, M.-A.; Légaré, M.-A.; Maron, L.; Fontaine, F.-G., A Highly Active Phosphine–Borane Organocatalyst for the Reduction of CO_2 to Methanol Using Hydroboranes. *J. Am. Chem. Soc.* **2013,** *135* (25), 9326–9329.
12. Wang, T.; Stephan, D. W., Phosphine Catalyzed reduction of CO_2 with boranes. *Chem. Commun.* **2014,** *50* (53), 7007–7010.
13. von Wolff, N.; Lefèvre, G.; Berthet, J. C.; Thuéry, P.; Cantat, T., Implications of CO_2 Activation by Frustrated Lewis Pairs in the Catalytic Hydroboration of CO_2: A View Using N/Si^+ Frustrated Lewis Pairs. *ACS Catal.* **2016,** *6* (7), 4526–4535.
14. Liu, L.; Lo, S.-K.; Smith, C.; Goicoechea, J. M., Pincer-Supported Gallium Complexes for the Catalytic Hydroboration of Aldehydes, Ketones and Carbon Dioxide. *Chem. Eur. J.* **2021,** *27* (69), 17379–17385.
15. Sau, S. C.; Bhattacharjee, R.; Vardhanapu, P. K.; Vijaykumar, G.; Datta, A.; Mandal, S. K., Metal-Free Reduction of CO_2 to Methoxyborane under Ambient Conditions through Borondiformate Formation. *Angew. Chem. Int. Ed.* **2016,** *55* (48), 15147–15151.
16. Das Neves Gomes, C.; Blondiaux, E.; Thuéry, P.; Cantat, T., Metal-Free Reduction of CO_2 with Hydroboranes: Two Efficient Pathways at Play for the Reduction of CO_2 to Methanol. *Chem. Eur. J.* **2014,** *20* (23), 7098–7106.

17. Yang, Y.; Xu, M.; Song, D., Organocatalysts with carbon-centered activity for CO_2 reduction with boranes. *Chem. Commun.* **2015,** *51* (56), 11293–11296.
18. Chia, C.-C.; Teo, Y.-C.; Cham, N.; Ho, S. Y.-F.; Ng, Z.-H.; Toh, H.-M.; Mézailles, N.; So, C.-W., Aluminum-Hydride-Catalyzed Hydroboration of Carbon Dioxide. *Inorg. Chem.* **2021,** *60* (7), 4569–4577.
19. Ramos, A.; Antiñolo, A.; Carrillo-Hermosilla, F.; Fernández-Galán, R., $Ph_2PCH_2CH_2B(C_8H_{14})$ and Its Formaldehyde Adduct as Catalysts for the Reduction of CO_2 with Hydroboranes. *Inorg. Chem.* **2020,** *59* (14), 9998–10012.
20. Bontemps, S.; Sabo-Etienne, S., Trapping Formaldehyde in the Homogeneous Catalytic Reduction of Carbon Dioxide. *Angew. Chem. Int. Ed.* **2013,** *52* (39), 10253–10255.
21. Chakraborty, S.; Zhang, J.; Krause, J. A.; Guan, H., An Efficient Nickel Catalyst for the Reduction of Carbon Dioxide with a Borane. *J. Am. Chem. Soc.* **2010,** *132* (26), 8872–8873.
22. Shintani, R.; Nozaki, K., Copper-Catalyzed Hydroboration of Carbon Dioxide. *Organometallics* **2013,** *32* (8), 2459–2462.
23. Gaussian 09, R. A., M. J. Frisch, G. W. Trucks, H. B. Schlegel, G. E. Scuseria, M. A. Robb, J. R. Cheeseman, G. Scalmani, V. Barone, G. A. Petersson, H. Nakatsuji, X. Li, M. Caricato, A. Marenich, J. Bloino, B. G. Janesko, R. Gomperts, B. Mennucci, H. P. Hratchian, J. V. Ortiz, A. F. Izmaylov, J. L. Sonnenberg, D. Williams-Young, F. Ding, F. Lipparini, F. Egidi, J. Goings, B. Peng, A. Petrone, T. Henderson, D. Ranasinghe, V. G. Zakrzewski, J. Gao, N. Rega, G. Zheng, W. Liang, M. Hada, M. Ehara, K. Toyota, R. Fukuda, J. Hasegawa, M. Ishida, T. Nakajima, Y. Honda, O. Kitao, H. Nakai, T. Vreven, K. Throssell, J. A. Montgomery, Jr., J. E. Peralta, F. Ogliaro, M. Bearpark, J. J. Heyd, E. Brothers, K. N. Kudin, V. N. Staroverov, T. Keith, R. Kobayashi, J. Normand, K. Raghavachari, A. Rendell, J. C. Burant, S. S. Iyengar, J. Tomasi, M. Cossi, J. M. Millam, M. Klene, C. Adamo, R. Cammi, J. W. Ochterski, R. L. Martin, K. Morokuma, O. Farkas, J. B. Foresman, and D. J. Fox, Gaussian, Inc., Wallingford CT, 2016.
24. Chai, J.-D.; Head-Gordon, M., Long-range Corrected Hybrid Density Functionals with Damped Atom–Atom Dispersion Corrections. *Phys. Chem. Chem. Phys.* **2008,** *10* (44), 6615–6620.
25. Weigend, F.; Ahlrichs, R., Balanced Basis Sets of Split Valence, Triple Zeta Valence and Quadruple Zeta Valence Quality for H to Rn: Design and Assessment of Accuracy. *Physical Chemistry Chemical Physics* **2005,** *7* (18), 3297–3305.
26. Courtemanche, M.-A.; Légaré, M.-A.; Maron, L.; Fontaine, F.-G., Reducing CO_2 to Methanol Using Frustrated Lewis Pairs: On the Mechanism of Phosphine–Borane-Mediated Hydroboration of CO_2. *J. Am. Chem. Soc.* **2014,** *136* (30), 10708–10717.
27. Marenich, A. V.; Cramer, C. J.; Truhlar, D. G., Universal Solvation Model Based on Solute Electron Density and on a Continuum Model of the Solvent Defined by the Bulk Dielectric Constant and Atomic Surface Tensions. *J. Phys. Chem. B* **2009,** *113* (18), 6378–6396.
